# Site‐Selective Peptide and Protein Functionalization with Cyclopropenium Cations

**DOI:** 10.1002/anie.202518939

**Published:** 2025-11-04

**Authors:** Adriana Faraone, Matteo Balletti, Aliénor Jeandin, Hang‐Fei Tu, Viktoria A. Ikonnikova, Laura S. Sojka, Marcos G. Suero

**Affiliations:** ^1^ Institute of Chemical Research of Catalonia (ICIQ‐CERCA) The Barcelona Institute of Science and Technology Av. Països Catalans 16 Tarragona 43007 Spain; ^2^ ICREA Pg. Lluis Companys 23 Barcelona 08010 Spain

**Keywords:** Bioconjugation, Cyclopropene, Cyclopropenium cation, Peptide, Protein

## Abstract

In the realm of organic chemistry, carbocations play a pivotal role as highly reactive intermediates in the synthesis of complex molecules. While cyclase enzymes construct terpenoid natural products through carbocation intermediates, the use of these electrophilic reactive species for peptide and protein bioconjugation in aqueous media remains unexplored. Herein, we disclose the discovery and development of a new chemical modification of peptides and proteins with aromatic cyclopropenium cations, selective at cysteine residues. The bioconjugation is fast, operationally simple, and occurs at low concentration in aqueous media, allowing for the installation of a tetrasubstituted cyclopropene ring with excellent site selectivity. Moreover, the cyclopropenylation is preferential to internal cysteines, thus complementing current methodologies for selective terminal cysteine bioconjugation. These studies further showcased the bioconjugates' utility as radical traps in a thiol–ene process, enabling the formation of cyclopropane‐linked conjugates.

## Graphical abstract

A cysteine‐selective bioconjugation using aromatic cyclopropenium cations is reported. The reaction proceeds rapidly under aqueous conditions, enabling site‐selective installation of tetrasubstituted cyclopropene rings on peptides and proteins. The method shows preference for internal cysteines and provides conjugates that serve as efficient radical traps in thiol–ene chemistry, forming stable cyclopropane‐linked products.

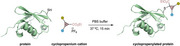

## Introduction

New site‐selective and site‐specific chemical modifications of peptides and proteins at endogenous amino acid residues are of the highest interest for pharmaceutical and biotechnology industries as well as chemical biology.^[^ ^1^ ^]^ Peptides and protein bioconjugates are gaining increasing attention for their potential as therapeutics in the form of antibody–drug conjugates,^[^ ^2^, ^3^, ^4^ ^]^ peptidomimetics,^[^ ^5^ ^]^ PEGylated proteins,^[^ ^6^, ^7^ ^]^ or stapled peptides.^[^ ^8^, ^9^, ^10^ ^]^

Bioconjugation reactions are ideally operationally simple, fast, and selective while maintaining compatibility with physiological conditions.^[^ ^11^, ^12^, ^13^, ^14^ ^]^ Despite the wealth of bioconjugation reactions developed for lysine^[^ ^15^ ^]^ and cysteine residues,^[^ ^16^, ^17^, ^18^ ^]^ and to a lesser extent, for histidine,^[^ ^19^, ^20^, ^21^ ^]^ tryptophan,^[^ ^22^, ^23^, ^24^, ^25^, ^26^ ^]^ methionine,^[^ ^27^, ^28^, ^29^ ^]^ phenylalanine,^[^ ^30^ ^]^ serine,^[^ ^31^ ^]^ or tyrosine,^[^ ^32^, ^33^, ^34^, ^35^ ^]^ the use of carbocations as electrophilic species remains unexplored. This can be explained by the poor stability of carbocations in water.^[^ ^36^, ^37^, ^38^, ^39^, ^40^, ^41^, ^42^ ^]^

**Figure 1:**
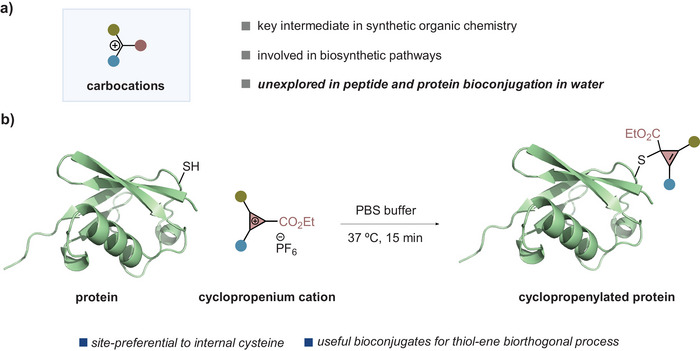
Carbocations for peptide and protein bioconjugation.

Carbocations are key reactive species in synthetic organic chemistry^[^ ^43^, ^44^, ^45^, ^46^ ^]^ and are involved in the biosynthesis of a broad range of natural products (Figure 1a).^[^ ^47^, ^48^ ^]^ Their stability usually increases with the *p*‐character of the hypovalent carbon atom, substitution with electron‐donor functionalities,^[^ ^49^ ^]^ non‐nucleophilic counterions,^[^ ^50^, ^51^, ^52^ ^]^ or stabilizing solvents.^[^ ^36^, ^43^, ^44^, ^45^, ^46^, ^53^, ^54^ ^]^ Among carbocations, cyclopropenium cations (CPCs) benefit from a particularly high stability due to their aromatic character. CPCs were reported for the first time in 1957 by Breslow,^[^ ^55^, ^56^, ^57^ ^]^ are generally stable salts and a range of derivatives substituted with aryl, alkyl, and heteroatoms can be prepared using multistep synthetic sequences.

In 2022, our group disclosed the first catalytic synthesis of CPCs by a [2 + 1] cycloaddition between alkynes and Rh(II)‐carbynoids as cationic monovalent carbon (:^+^C–R) transfer species.^[^ ^58^ ^]^ The process was able to generate for the first time CPCs substituted with ester functionalities, which enhance the CPC electrophilicity and impart regioselectivity in the nucleophilic attack to the alpha carbonyl position. A broad range of nucleophiles reacted with the CPCs, thus providing a novel disconnection approach to cyclopropene rings. These CPCs were also used as three‐carbon building blocks in a regioselective late‐stage aryl C─H bond cyclopropenylation of densely functionalized drug molecules and natural products.^[^ ^59^ ^]^ Considering the broad reactivity of our CPCs, we wondered whether a chemical modification of peptides and proteins could be attainable. We speculated that the major problems that could hamper this novel bioconjugation were i) site selectivity: diverse nucleophilic amino acid side chains are present in these biomolecules, and ii) low conversion: undesired water attack to the CPC could happen in a highly diluted aqueous media. If successful, however, such bioconjugation method would enable the selective installation of a cyclopropene ring, a functionality that has proven to be useful for in vitro and in vivo bioorthogonal reactions, such as Inverse Electron‐Demand Diels–Alder (IEDDA) reactions or [3 + 2] cycloadditions.^[^ ^60^, ^61^, ^62^, ^63^, ^64^, ^65^, ^66^, ^67^, ^68^ ^]^

Our strategy would remarkably differ from known protocols for the installation of cyclopropene rings in peptides and proteins, which rely on technically challenging and time‐consuming metabolic engineering or poorly selective covalent modifications with designed cyclopropene reagents.^[^ ^60^, ^69^, ^70^ ^]^ Furthermore, cyclopropenes have been used in bioconjugation of cysteines as Michael acceptors,^[^ ^71^ ^]^ leading to structurally different products with the loss of the cyclopropene motif.

Herein, we disclose the discovery and development of a site‐selective bioconjugation at cysteine residues in peptides and proteins with CPCs (Figure 1b). The chemical modification enabled the introduction of a tetrasubstituted cyclopropene, which proved to be useful as bioorthogonal handle in a thiol–ene process for the generation of a small library of cyclopropanated bioconjugates.

## Results and Discussion

### Reaction Optimization

We initiated this project by testing at 22 °C during 15 min, N‐ and C‐protected amino acids—cysteine, tyrosine, tryptophan, serine, methionine, phenylalanine, and histidine—whose side chains could potentially react with CPC **2a**. By using phosphate saline buffer (PBS) and 2,2,2‐trifluoroethanol (TFE) to guarantee solubilization of protected amino acids, we were pleased to find that only cysteine led to the formation of **3** in 91% yield (Table 1a). The addition of the sulfur nucleophile occurred with excellent regioselectivity at the cyclopropenium carbon atom substituted with the ester group. This is in line with our previous observation that nucleophilic attack on the CPC happens at the same carbon atom and was attributed to an orbital control.^[^ ^58^ ^]^ Nevertheless, using amino acids with other nucleophilic group (Table 1a) did not afford the corresponding cyclopropenylated derivatives (as assessed by UHPLC‐MS and ^1^H NMR analysis). However, we observed degradation to a complex mixture of unidentified products in the case of tryptophan.

**Table 1:** Discovery and optimization of a Cys‐selective bioconjugation with cyclopropenium reagents.^a)^

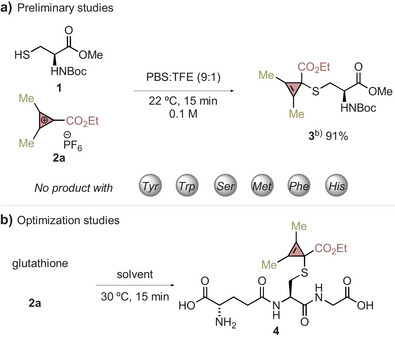				
Entry	**2a** (equiv)	Solvent	Concentration (mM)	% Yield **4** ^c)^
1	3	PBS:TFE	100	99^d)^
2	3	PBS:TFE	10	21^e)^
3	3	PBS:TFE	1	0^f)^
4	50	PBS:TFE	1	5
5	100	PBS:TFE	1	53
6	100	PBS	1	65
7	100	PBS	1	75^g)^
8	20	PBS	5	99^g)^, ^h)^

PBS = phosphate saline buffer, 10 mM concentration. TFE = 2,2,2‐trifluoroethanol. All reactions were carried out in 15 min and no improved yields were observed after this time because of full degradation of **2a**.

^a)^ All reactions were run on 0.1 µmol scale unless otherwise stated.

^b)^ Yield of isolated product, reaction run on 0.1 mmol scale.

^c)^ Analytical yields were calculated on UHPLC‐MS using sulisobenzone as an internal standard.

^d)^ Reaction run on a 20 µmol scale.

^e)^ Reaction run on a 10 µmol scale.

^f)^ Reaction run on a 1 µmol scale.

^g)^ Temperature 37 °C.

^h)^ Reaction run on a 0.5 µmol scale.

Having established the selective reactivity of our CPC with cysteine, we aimed to develop a site‐selective functionalization of peptides and proteins. For the optimization, we selected the commercially available tripeptide glutathione (GSH). Under these reaction conditions, we observed the quantitative functionalization of GSH to the cyclopropenylated tripeptide **4** (Table 1b, entry 1). We anticipated that performing this bioconjugation reaction on more complex peptides and proteins would require higher dilution conditions. Lowering the GSH concentration to 10 mM resulted in a dramatic drop in the reaction efficiency (entry 2), and further dilution to a 1 mM concentration led to a complete shutdown of the reaction because of degradation of **2a** (entry 3). After this, we were glad to see that by increasing the equivalents of **2a**, **4** could be obtained in moderate yields (entries 4 and 5). Further improvement of the efficiency was observed by performing the bioconjugation without TFE (entry 6) and at 37 °C (entry 7). Finally, increasing the concentration of GSH to 5 mM allowed us to reduce the equivalents of **2a**, leading again to the quantitative formation of **4** (Table 1b, entry 8). The reaction could be scaled up to 0.5 mmol, which allowed for the isolation of **4** in 82% yield by reverse‐phase chromatography, and its structure was confirmed by NMR and HRMS. We observed that **4** was stable to highly acidic and alkaline pH over 3 weeks at room temperature and could be stored in solution under common laboratory light exposure without noticeable decomposition (see Supporting Information, section 12), showcasing the remarkably high stability of the cyclopropenylated bioconjugate **4**. Control experiments carried out under the optimized reaction conditions with alternative aromatic cations, such as diphenylcyclopropenium or tropylium, did not afford the expected bioconjugated products as judged by UHPLC‐MS and ^1^H NMR analysis, which may be due to a lower electrophilicity (see Supporting Information, Section 9).

### Peptide Scope

**Figure 2:**
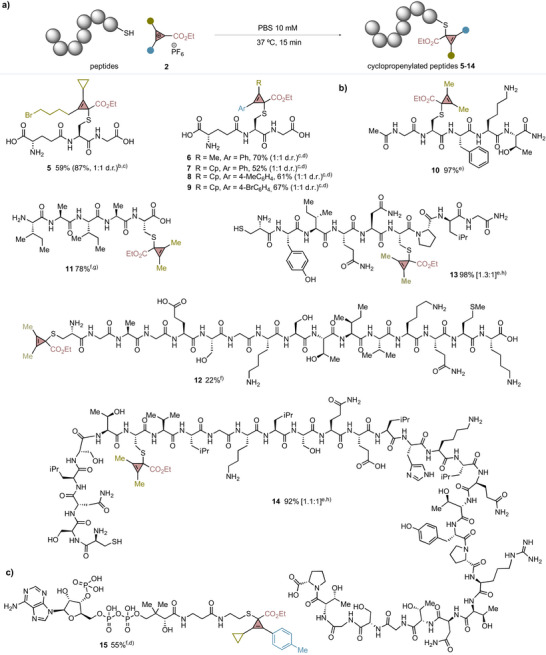
Scope of peptides. ^a)^All the reactions were run on a 0.1 µmol scale, using 10 mM PBS buffer. Conversions were determined by relative integration based on UHPLC‐MS unless otherwise stated. ^b)^30% of 2,2,2‐trifluoroethanol used as co‐solvent. ^c)^Yield of isolated product as trifluoroacetic acid salt after purification by reverse‐phase chromatography. ^d)^20% of MeCN used as co‐solvent. ^e)^Reaction run using 100 equiv of CPC. ^f)^Reaction run using 20 equiv of CPC. ^g)^10% of dimethyl formamide used as co‐solvent. ^h)^Reduction with 1.2 or 2 equiv of TCEP prior to CPC addition. See Supporting Information for details. For substrates bearing two different cysteine residues, both mono‐ and bis‐cyclopropenylated products were detected, and the values in brackets denote their relative ratio.

We next turned our attention to evaluating the scope of CPCs using GSH as a benchmark peptide (Figure 2a). We observed that the bioconjugation reaction tolerated CPCs decorated with different substituents, such as alkyl or aryl bromides, which could serve as linchpins for further manipulations, delivering cyclopropenylated peptides **5**–**9** in good yields and as mixtures of diastereomers (52%–87%, d.r. = 1:1).^[^ ^72^ ^]^ Due to the lower aqueous solubility of phenyl‐substituted CPCs, a small volume of co‐solvent such as acetonitrile (MeCN) was necessary to solubilize the reagent and increase the yield of product while using a small excess of CPC. After this, we demonstrated that the commercial pentapeptide Ac‐GCFKT‐NH_2_ could be efficiently and selectively cyclopropenylated at the internal cysteine in the presence of lysine (Figure 2b, **10**, 97%). 2D‐NMR‐ROESY experiment carried out with **10** unequivocally proved the site‐selectivity at the cysteine residue instead of serine or lysine (see Supporting Information, section 6, Figure S3).

We then examined the impact of cysteine positioning at the N‐ or C‐terminus on the efficiency of the reaction. We were surprised to observe that peptide H‐IAIAC‐OH with a C‐terminal cysteine delivered **11** in good conversion, while fragment of the alpha subunit of the guanosine‐5′‐triphosphate (GTP)‐binding protein, a peptide with a N‐terminal cysteine, provided a notably lower conversion for **12** (22%). This relative discrepancy led us to anticipate that site‐specificity could be achieved in peptides containing both a N‐terminal and an internal cysteine. With this hypothesis in mind, we reduced the disulfide bridge of oxytocin with tris(2‐carboxyethyl)phosphine (TCEP). The resulting linear peptide was then treated with CPC **2a**, which afforded the peptide cyclopropenylated at the internal cysteine as the major product (**13**, 59%). While dual modification of both cysteines was detected (13*, 39%), the lack of selective labeling at the N‐terminus indicates preference for internal cysteine alkylation.

**Figure 3:**
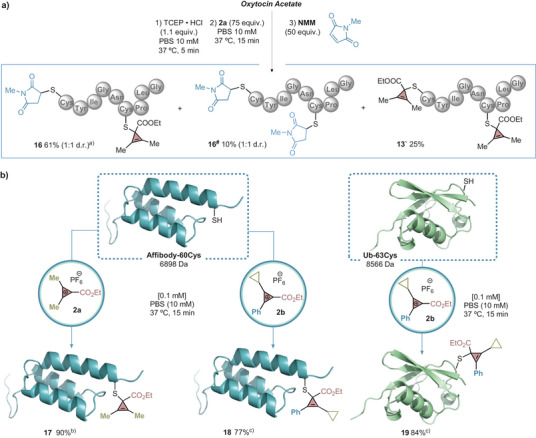
Orthogonal functionalization of oxytocin and bioconjugation of proteins. ^a)^Reaction also produced 25% of CPC double alkylation product and 10% of NMM double alkylation product. ^b)^1000 equiv of CPC used. ^c)^500 equiv of CPC used. Conversions were determined by relative integration based on UHPLC–MS. See Supporting Information for details.

Control experiments using 2‐formylbenzeneboronic acid as a N‐terminal selective cysteine bioconjugation reagent developed by Gois,^[^ ^73^ ^]^ confirmed the preferential internal site‐selectivity of our bioconjugation reagent (see Supporting Information, section 10). At present, the origin of such selectivity is not fully understood and we believe that electrostatic repulsion between the cyclopropenium cation and the protonated N‐terminus of the peptide could be responsible for this observation. The reduced open form of the peptide hormone salmon calcitonin followed a similar trend delivering the product in a 1.1:1 mixture of mono‐ and bis‐alkylated in 92% conversion (**14**, 48% + 14* 44%). As anticipated by the preliminary studies, our bioconjugation reaction failed to functionalize tryptophan‐containing peptides, such as H‐HCKFWW‐OH or octreotide acetate. In these cases, complex crude mixtures were obtained, containing degradation products of the starting material. Finally, we demonstrated that the protocol was amenable for the cyclopropenylation of other biologically relevant molecules such as coenzyme A, delivering the corresponding bioconjugated product **15** in 55% conversion (Figure 2c). Given the preferential internal selectivity observed for substrates bearing two nonequivalent cysteine residues, as in compound **13**, we explored a sequential and orthogonal functionalization of oxytocin. After reduction of the disulfide bridge, oxytocin was first reacted with CPC **2a** to functionalize the internal cysteine, consistent with previous observations (product **13**, Figure 2a), while the terminal cysteine remained available for further modification. The resulting 1.3:1 mixture of mono‐ and bis‐cyclopropenylated species (**13** + **13′**) was subsequently subjected to a thiol–Michael reaction with *N*‐methylmaleimide (NMM),^[^ ^74^ ^]^ affording product **16** in 61% conversion, along with the bis‐cyclopropenylated (**13′**, 25%) and NMM double‐functionalized (**16^#^**, 10%) derivatives (Figure 3a). Although accompanied by side products, this sequence provides a proof of concept for divergent cysteine‐selective functionalization in oxytocin and, in principle, in other multicysteine peptides.

### Protein Scope

Having explored the potential of our bioconjugation method for the installation of a cyclopropene moiety on peptides, we next investigated the reactivity of our CPC reagents **2a** with small proteins (Figure 3b). We first selected the affibody‐60Cys, an antibody mimetic protein derived from the Z domain of *Staphylococcus aureus* protein A.^[^ ^75^, ^76^ ^]^ The original 58 amino acid affibody was engineered to introduce a pentaglycine tag at the N‐terminal position and the mutation Q55C.^[^ ^77^ ^]^ The resulting 63 amino acid affibody‐60Cys was treated with CPC reagent **2a** in PBS buffer at 100 micromolar concentration, resulting in the efficient incorporation of the cyclopropene moiety at cysteine (**17**, 90%). While MALDI‐TOF analysis confirmed the mass of the modified protein, tryptic digestion followed by tandem mass spectrometry failed to detect the desired modification in the sequence. Various MS/MS parameters, including different collision energies and voltages, were tested, but in all cases we observed the loss of the CPC fragment in the gas phase during analysis. The loss of the CPC during MS/MS is a significant limitation, as it weakens site‐specificity assignments. However, to corroborate the site‐selectivity in a complex system such as a protein, a control experiment carried out with affibody‐60Glu (cysteine has been mutated with a glutamine amino acid) showed no conversion to a CPC‐modified protein confirming that bioconjugation takes place exclusively at the cysteine residue (see Supporting Information, section 7). Likewise, employing the CPC reagent **2b** under similar conditions, bioconjugate **18** was formed in 77% conversion. Next, we investigated engineered ubiquitin‐63Cys, composed of 77 amino acids and modified with a surface‐exposed cysteine at residue 63.^[^ ^78^ ^]^ While CPC **2a** failed to afford the expected bioconjugation product, we observed that **2b** led to 84% conversion of the bioconjugated protein **19**.^[^ ^79^ ^]^ MALDI‐TOF analysis confirmed in all cases the formation of the desired bioconjugated proteins, while circular dichroism corroborated that the bioconjugation did not alter the protein secondary structure (see Supporting Information, section 7). Nonetheless, the reaction failed to deliver the expected product in the case of β‐lactoglobulin, probably due to the presence of tryptophan residues in positions 19 and 61 of the sequence.

### Synthetic Applications

**Figure 4:**
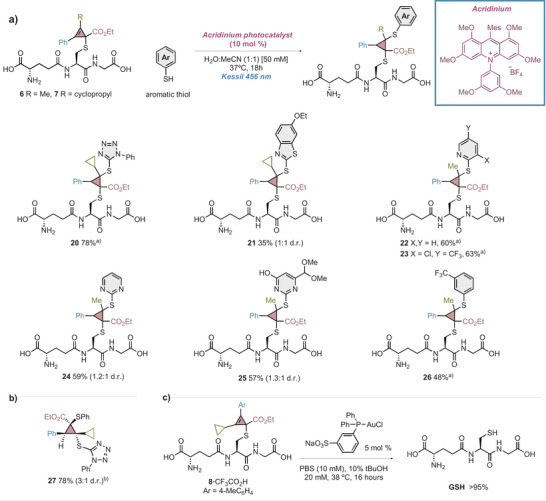
Photocatalytic thiol‐ene reaction and decaging. ^a)^Diastereomers inseparable by UHPLC‐MS; d.r. not determined. ^b)^Yield and d.r. refer to isolated product through flash column chromatography.

Next, we aimed to illustrate the potential of our site‐selective cysteine bioconjugation by identifying a reaction in aqueous media that could functionalize the tetrasubstituted cyclopropene rings. We discovered that cyclopropenylated GSH derivatives bearing a phenyl substituent could undergo a photoredox‐catalyzed thiol–ene coupling with (hetero)aromatic thiols under mild reaction conditions using an acridinium photocatalyst (Figure 4a).^[^ ^80^, ^81^ ^]^ The reaction tolerated five‐ and six‐membered ring heterocycles (**20**–**25**) as well as substituted benzene rings (**26**). The reaction generated an inseparable mixture of diastereoisomers at the stereogenic center formed with the aromatic thiol. GOESY experiments carried out with compound **27** suggested that the major diastereomer features the phenyl ring and ester group in *trans* disposition (Figure 4b). Moreover, Stern–Volmer fluorescent quenching studies confirmed thiol quenching of the photocatalyst excited state, generating a thiyl radical via radical cation formation and deprotonation (see Supporting Information, section 8). Under the optimized conditions, aliphatic thiols failed to deliver the desired bioconjugates.

It is worth highlighting that despite the relevance of cyclopropanes in drug design, bioorthogonal reactions that involve the creation of a cyclopropane ring in peptides or proteins are largely unexplored and limited to the IEDDA developed by Prescher and coworkers.^[^ ^60^, ^61^, ^62^, ^63^, ^64^, ^65^, ^66^, ^67^, ^68^, ^69^, ^70^ ^]^ Additionally, this reaction manifold is limited to uncongested cyclopropenes and fails when more sterically encumbered substrates are employed. Therefore, the thiol–ene reactivity presented therein offers an orthogonal strategy for cyclopropene functionalization in biological contexts, significantly expanding their utility as bioorthogonal reporters. This methodology showcases the possibility of an additional approach^[^ ^82^ ^]^ to peptide–drug conjugates, which are of significant interest in medicinal chemistry and drug discovery.^[^ ^83^ ^]^ Finally, we found suitable reaction conditions for decaging cyclopropenylated peptides in aqueous media.^[^ ^84^, ^85^, ^86^, ^87^ ^]^ Such process involved the use of a water‐soluble Au(I) catalyst and *tert*‐butanol as cosolvent and enabled >95% conversion of **8** into GSH in (Figure 4c) showing the reversibility of our bioconjugation protocol.^[^ ^75^, ^76^ ^]^

## Conclusion

We have developed the first peptide and protein bioconjugation with a carbocation species in aqueous media. Key to the process was the use of cyclopropenium cations that preferentially reacted with internal cysteine residues. Our bioconjugation complements well‐known selective cysteine bioconjugations that use maleimides,^[^ ^74^ ^]^ iodoacetamides,^[^ ^12^ ^]^ palladium‐based reagents,^[^ ^77^ ^]^ or hypervalent iodine compounds^[^ ^35^ ^]^ since they are unable to install a tetrasubstituted cyclopropene ring. Moreover, it provides highly stable bioconjugates, avoiding the use of transition metals or potentially explosive reagents. The potential of the cyclopropene as a versatile bioorthogonal handle was exemplified through the discovery and development of a thiol–ene reaction with diverse aromatic thiols that led to cyclopropane‐linked bioconjugates. It is worth highlighting that although alternative cysteine‐selective cyclopropylations exist,^[^ ^71^ ^]^ our biorthogonal process is featured by its modularity and scope using readily available or commercial aromatic thiols. Moreover, while photo‐mediated thiol–ene reactions typically use an external olefin to trap cysteine‐derived thiyl radicals, our protocol achieves the opposite by using an external thiol, offering a complementary approach to most traditional thiol–ene click strategies.

## Conflict of Interests

The authors declare no conflict of interest.

## Supporting information



Supporting Information

Supporting Information

## Data Availability

The data that support the findings of this study are available in the Supporting Information of this article.

## References

[1] F. Li , R. I. Mahato , Mol. Pharmaceutics 2017, 14, 1321–1324, 10.1021/acs.molpharmaceut.7b00263. PMC5793904 28457140

[2] A. Beck , L. Goetsch , C. Dumontet , C. Corvaïa , Nat. Rev. Drug Discov. 2017, 16, 315–337, 10.1038/nrd.2016.268. 28303026

[3] Z. Fu , S. Li , S. Han , C. Shi , Y. Zhang , Signal Transduct. Target. Ther. 2022, 7, 93, 10.1038/s41392-022-00947-7. 35318309 PMC8941077

[4] C. do Pazo , K. Nawaz , R. M. Webster , Nat. Rev. Drug Discov. 2021, 20, 583–584, 10.1038/d41573-021-00054-2. 33762691

[5] P. Ryan , B. Patel , V. Makwana , H. R. Jadhav , M. Kiefel , A. Davey , T. A. Reekie , S. Rudrawar , M. Kassiou , ACS Chem. Neurosci. 2018, 9, 1530–1551, 10.1021/acschemneuro.8b00185. 29782794

[6] F. M. Veronese , G. Pasut , Drug Discov. Today 2005, 10, 1451–1458, 10.1016/S1359-6446(05)03575-0. 16243265

[7] K. Knop , R. Hoogenboom , D. Fischer , U. S. Schubert , Angew. Chem. Int. Ed. 2010, 49, 6288–6308, 10.1002/anie.200902672. 20648499

[8] M. T. J. Bluntzer , J. O'Connell , T. S. Baker , J. Michel , A. N. Hulme , Pept. Sci. 2021, 113, e24191, 10.1002/pep2.24191.

[9] Y. H. Lau , P. de Andrade , Y. Wu , D. R. Spring , Chem. Soc. Rev. 2015, 44, 91–102, 10.1039/C4CS00246F. 25199043

[10] A. A. Vinogradov , Y. Yin , H. Suga , J. Am. Chem. Soc. 2019, 141, 4167–4181, 10.1021/jacs.8b13178. 30768253

[11] C. D. Spicer , B. G. Davis , Nat. Commun. 2014, 5, 4740, 10.1038/ncomms5740. 25190082

[12] O. Boutureira , G. J. L. Bernardes , Chem. Rev. 2015, 115, 2174–2195, 10.1021/cr500399p. 25700113

[13] E. A. Hoyt , P. M. S. D. Cal , B. L. Oliveira , G. J. L. Bernardes , Nat. Rev. Chem. 2019, 3, 147–171, 10.1038/s41570-019-0079-1.

[14] J. N. deGruyter , L. R. Malins , P. S. Baran , Biochemistry 2017, 56, 3863–3873, 10.1021/acs.biochem.7b00536. 28653834 PMC5792174

[15] M. Haque , N. Forte , J. R. Baker , Chem. Commun. 2021, 57, 10689–10702, 10.1039/D1CC03976H. PMC8516052 34570125

[16] P. Ochtrop , C. P. R. Hackenberger , Curr. Opin. Chem. Biol. 2020, 58, 28–36, 10.1016/j.cbpa.2020.04.017. 32645576

[17] J. You , J. Zhang , J. Wang , M. Jin , Bioconjug. Chem. 2021, 32, 1525–1534, 10.1021/acs.bioconjchem.1c00213. 34105345

[18] F. Chen , J. Gao , Chem. Eur. J. 2022, 28, e202201843, 10.1002/chem.202201843. 35970770 PMC9701160

[19] S. Jia , D. He , C. J. Chang , J. Am. Chem. Soc. 2019, 141, 7294–7301, 10.1021/jacs.8b11912. 31017395 PMC6996876

[20] K. Peciak , E. Laurine , R. Tommasi , J. Choi , S. Brocchini , Chem. Sci. 2019, 10, 427–439, 10.1039/C8SC03355B. 30809337 PMC6354831

[21] C. Wan , Y. Wang , C. Lian , Q. Chang , Y. An , J. Chen , J. Sun , Z. Hou , D. Yang , X. Guo , F. Yin , R. Wang , Z. Li , Chem. Sci. 2022, 13, 8289–8296, 10.1039/D2SC02353A. 35919717 PMC9297702

[22] M. Nuruzzaman , B. M. Colella , C. P. Uzoewulu , A. E. Meo , E. J. Gross , S. Ishizawa , S. Sana , H. Zhang , M. E. Hoff , B. T. W. Medlock , E. C. Joyner , S. Sato , E. A. Ison , Z. Li , J. Ohata , J. Am. Chem. Soc. 2024, 146, 6773–6783, 10.1021/jacs.3c13447. 38421958

[23] Y. Seki , T. Ishiyama , D. Sasaki , J. Abe , Y. Sohma , K. Oisaki , M. Kanai , J. Am. Chem. Soc. 2016, 138, 10798–10801, 10.1021/jacs.6b06692. 27534812

[24] S. J. Tower , W. J. Hetcher , T. E. Myers , N. J. Kuehl , M. T. Taylor , J. Am. Chem. Soc. 2020, 142, 9112–9118, 10.1021/jacs.0c03039. 32348670 PMC7292481

[25] C. R. Hoopes , F. J. Garcia , A. M. Sarkar , N. J. Kuehl , D. T. Barkan , N. L. Collins , G. E. Meister , T. R. Bramhall , C.‐H. Hsu , M. D. Jones , M. Schirle , M. T. Taylor , J. Am. Chem. Soc. 2022, 144, 6227–6236, 10.1021/jacs.1c10536. 35364811 PMC10124759

[26] M. Imiołek , G. Karunanithy , W.‐L. Ng , A. J. Baldwin , V. Gouverneur , B. G. Davis , J. Am. Chem. Soc. 2018, 140, 1568–1571, 10.1021/jacs.7b10230. 29301396 PMC5806083

[27] S. Lin , X. Yang , S. Jia , A. M. Weeks , M. Hornsby , P. S. Lee , R. V. Nichiporuk , A. T. Iavarone , J. A. Wells , F. D. Toste , C. J. Chang , Science 2017, 355, 597–602, 10.1126/science.aal3316. 28183972 PMC5827972

[28] M. T. Taylor , J. E. Nelson , M. G. Suero , M. J. Gaunt , Nature 2018, 562, 563–568, 10.1038/s41586-018-0608-y. 30323287 PMC6203954

[29] J. Kim , B. X. Li , R. Y.‐C. Huang , J. X. Qiao , W. R. Ewing , D. W. C. MacMillan , J. Am. Chem. Soc. 2020, 142, 21260–21266, 10.1021/jacs.0c09926. 33290649 PMC8647115

[30] Y. Weng , C.‐J. Su , H. Jiang , C.‐W. Chiang , Sci. Rep. 2022, 12, 18994, 10.1038/s41598-022-23481-6. 36348051 PMC9643349

[31] J. C. Vantourout , S. R. Adusumalli , K. W. Knouse , D. T. Flood , A. Ramirez , N. M. Padial , A. Istrate , K. Maziarz , J. N. deGruyter , R. R. Merchant , J. X. Qiao , M. A. Schmidt , M. J. Deery , M. D. Eastgate , P. E. Dawson , G. J. L. Bernardes , P. S. Baran , J. Am. Chem. Soc. 2020, 142, 17236–17242, 10.1021/jacs.0c05595. 32965106 PMC8350984

[32] H. Ban , J. Gavrilyuk , C. F. Barbas , J. Am. Chem. Soc. 2010, 132, 1523–1525, 10.1021/ja909062q. 20067259

[33] K. Maruyama , T. Ishiyama , Y. Seki , K. Sakai , T. Togo , K. Oisaki , M. Kanai , J. Am. Chem. Soc. 2021, 143, 19844–19855, 10.1021/jacs.1c09066. 34787412

[34] B. X. Li , D. K. Kim , S. Bloom , R. Y.‐C. Huang , J. X. Qiao , W. R. Ewing , D. G. Oblinsky , G. D. Scholes , D. W. C. MacMillan , Nat. Chem. 2021, 13, 902–908, 10.1038/s41557-021-00733-y. 34183819

[35] N. Declas , J. R. J. Maynard , L. Menin , N. Gasilova , S. Götze , J. L. Sprague , P. Stallforth , S. Matile , J. Waser , Chem. Sci. 2022, 13, 12808–12817, 10.1039/D2SC04558C. 36519034 PMC9645396

[36] Despite the intrinsic fleeting nature of these intermediates, it is well known that allylic/benzylic carbocations are sufficiently stable to survive and react in aqueous media with nucleophiles: J. P. Richard , T. L. Amyes , M. M. Toteva , Acc. Chem. Res. 2001, 34, 981–988, 10.1021/ar0000556. 11747416

[37] M. Hofmann , N. Hampel , T. Kanzian , H. Mayr , Angew. Chem. Int. Ed. 2004, 43, 5402–5405, 10.1002/anie.200460812. 15468080

[38] S. Minegishi , R. Loos , S. Kobayashi , H. Mayr , J. Am. Chem. Soc. 2005, 127, 2641–2649, 10.1021/ja045562n. 15725021

[39] M. Westermaier , H. Mayr , Org. Lett. 2006, 8, 4791–4794, 10.1021/ol0618555. 17020304

[40] P. G. Cozzi , L. Zoli , Green Chem. 2007, 9, 1292, 10.1039/b711523g.

[41] S. Shirakawa , S. Kobayashi , Org. Lett. 2007, 9, 311–314, 10.1021/ol062813j. 17217292

[42] P. G. Cozzi , L. Zoli , Angew. Chem. Int. Ed. 2008, 120, 4230–4234, 10.1002/ange.200800622.

[43] P. J. Stang , In Carbocation Chemistry (Eds: G. A. Olah , G. K. S. Prakash ), Wiley, Hoboken, NJ 2004, pp. 1–120.

[44] G. A. Olah , J. Org. Chem. 2001, 66, 5943–5957, 10.1021/jo010438x. 11529717

[45] For an excellent historical account of the development of carbocation chemistry, see: C. D. Nenitzescu , in Carbonium Ions (Eds: G. A. Olah , P. v. R. Schleyer ), Wiley, New York 1968, pp. 1.

[46] R. R. Naredla , D. A. Klumpp , Chem. Rev. 2013, 113, 6905–6948, 10.1021/cr4001385. 23819438

[47] R. A. Yoder , J. N. Johnston , Chem. Rev. 2005, 105, 4730–4756, 10.1021/cr040623l. 16351060 PMC2575671

[48] D. J. Tantillo , Nat. Prod. Rep. 2011, 28, 1035, 10.1039/c1np00006c. 21541432

[49] E. V. Anslyn , D. A. Dougherty , Modern Physical Organic Chemistry, University Science Books, Sausalito, CA 2006.

[50] A. C. Reed , Acc. Chem. Res. 1998, 31, 133–139, 10.1021/ar970230r.

[51] A. C. Reed , Acc. Chem. Res. 2010, 43, 121–128, 10.1021/ar900159e. 19736934 PMC2808449

[52] T. Kato , A. C. Reed , Angew. Chem. Int. Ed. 2004, 43, 2908–2911, 10.1002/anie.200453931. 15170300

[53] I. Colomer , A. Chamberlain , M. Haughey , T. J. Donhoe , Nat. Rev. Chem. 2017, 1, 0088, 10.1038/s41570-017-0088.

[54] H. F. Motiwala , A. M. Armaly , J. G. Cacioppo , T. C. Coombs , K. R. K. Koehn , V. M. Norwood , J. Aubé , Chem. Rev. 2022, 122, 12544–12747, 10.1021/acs.chemrev.1c00749. 35848353

[55] R. Breslow , J. Am. Chem. Soc. 1957, 79, 5318, 10.1021/ja01576a067.

[56] R. Breslow , C. Yuan , J. Am. Chem. Soc. 1958, 80, 5991–5994, 10.1021/ja01555a026.

[57] K. Komatsu , T. Kitagawa , Chem. Rev. 2003, 103, 1371–1428, 10.1021/cr010011q. 12683786

[58] H.‐F. Tu , A. Jeandin , M. G. Suero , J. Am. Chem. Soc. 2022, 144, 16737–16743, 10.1021/jacs.2c07769. 36074785 PMC9501905

[59] H.‐F. Tu , A. Jeandin , C. Bon , C. Brocklehurst , F. Lima , M. G. Suero , Angew. Chem. Int. Ed. 2023, 62, e202308379, 10.1002/anie.202308379. 37459194

[60] D. M. Patterson , L. A. Nazarova , B. Xie , D. N. Kamber , J. A. Prescher , J. Am. Chem. Soc. 2012, 134, 18638–18643, 10.1021/ja3060436. 23072583

[61] Z. Yu , Y. Pan , Z. Wang , J. Wang , Q. Lin , Angew. Chem. Int. Ed. 2012, 51, 10600–10604, 10.1002/anie.201205352. PMC3517012 22997015

[62] D. N. Kamber , L. A. Nazarova , Y. Liang , S. A. Lopez , D. M. Patterson , H. W. Shih , K. N. Houk , J. A. Prescher , J. Am. Chem. Soc. 2013, 135, 13680–13683, 10.1021/ja407737d. 24000889

[63] A.‐C. Knall , C. Slugovc , Chem. Soc. Rev. 2013, 42, 5131, 10.1039/c3cs60049a. 23563107

[64] D. M. Patterson , L. A. Nazarova , J. A. Prescher , ACS Chem. Biol. 2014, 9, 592–605, 10.1021/cb400828a. 24437719

[65] J. M. J. M. Ravasco , C. M. Monteiro , A. F. Trindade , Org. Chem. Front. 2017, 4, 1167–1198, 10.1039/C7QO00054E.

[66] B. L. Oliveira , Z. Guo , G. J. L. Bernardes , Chem. Soc. Rev. 2017, 46, 4895–4950, 10.1039/C7CS00184C. 28660957

[67] H. Wu , N. K. Devaraj , Acc. Chem. Res. 2018, 51, 1249–1259, 10.1021/acs.accounts.8b00062. 29638113 PMC6225996

[68] D. Schauenburg , T. Weil , J. Am. Chem. Soc. 2025, 147, 8049–8062, 10.1021/jacs.4c15986. 40017419 PMC11912343

[69] A. Sachdeva , K. Wang , T. Elliott , J. W. Chin , J. Am. Chem. Soc. 2014, 136, 7785–7788, 10.1021/ja4129789. 24857040 PMC4333588

[70] C. P. Ramil , M. Dong , P. An , T. M. Lewandowski , Z. Yu , L. J. Miller , Q. Lin , J. Am. Chem. Soc. 2017, 139, 13376–13386, 10.1021/jacs.7b05674. 28876923 PMC5753752

[71] N. J. Smith , K. Rohlfing , L. A. Sawicki , P. M. Kharkar , S. J. Boyd , A. M. Kloxin , J. M. Fox , Org. Biomol. Chem. 2018, 16, 2164–2169, 10.1039/C8OB00166A. 29521395 PMC5877462

[72] Cyclopropenium cations were synthesized from the corresponding alkyne with a Rh‐catalyzed cyclopropenylation reaction and can be decorated with a different range of functional groups (see Ref. 58). These reagents are not air‐sensitive but need to be stored at −25 °C to avoid degradation.

[73] H. Faustino , M. J. S. A. Silva , L. F. Veiros , G. J. L. Bernardes , P. M. P. Gois , Chem. Sci. 2016, 7, 5052–5058, 10.1039/C6SC01520D. 30155155 PMC6018717

[74] J. M. J. M. Ravasco , H. Faustino , A. Trindade , P. M. P. Gois , Chem. Eur. J. 2019, 25, 43–59, 10.1002/chem.201803174. 30095185

[75] M. Högbom , M. Eklund , P. Å. Nygren , P. Nordlund , Proc. Natl. Acad. Sci. USA 2003, 100, 3191–3196, 10.1073/pnas.0436100100. 12604795 PMC404300

[76] R. N. Gilbreth , S. Koide , Curr. Opin. Struct. Biol. 2012, 22, 413–420, 10.1016/j.sbi.2012.06.001. 22749196 PMC3423532

[77] E. V. Vinogradova , C. Zhang , A. M. Spokoyny , B. L. Pentelute , S. L. Buchwald , Nature 2015, 526, 687–691, 10.1038/nature15739. 26511579 PMC4809359

[78] B. Lee , S. Sun , E. Jiménez‐Moreno , A. A. Neves , G. J. L. Bernardes , Bioorg. Med. Chem. 2018, 26, 3060–3064, 10.1016/j.bmc.2018.02.028. 29482952

[79] At present, we do not fully understand the exact reasons why reagent **2a** is not suitable for the bioconjugation of ubiquitin‐63Cys. However, cyclopropyl‐substituted CPC **2b** led to good conversion and that can be due to a higher stability of **2c**. It is well‐known the stabilization properties of cyclopropyl rings of cyclopropenium cations by hyperconjugation of the cyclopropyl C─C bond with the contiguous 2p orbital of the CPC. For a reference see: R. A. Moss , S. Shilan , K.‐J. Karsten , J. A. Potenza , H. J. Schugar , R. C. Munjal , J. Am. Chem. Soc. 1986, 108, 134–140, 10.1021/ja00261a022.

[80] C. E. Hoyle , C. N. Bowman , Angew. Chem. Int. Ed. 2010, 49, 1540–1573, 10.1002/anie.200903924. 20166107

[81] A. Joshi‐Pangu , F. Lévesque , H. G. Roth , S. F. Oliver , L. Campeau , D. Nicewicz , D. A. DiRocco , J. Org. Chem. 2016, 81, 7244–7249, 10.1021/acs.joc.6b01240. 27454776

[82] J. Yu , X. Yang , Y. Sun , Z. Yin , Angew. Chem. Int. Ed. 2018, 57, 11598–11602, 10.1002/anie.201804801. 29984549

[83] T. T. Dean , J. Jelú‐Reyes , A. C. Allen , T. W. Moore , J. Med. Chem. 2024, 67, 1641–1661, 10.1021/acs.jmedchem.3c01835. 38277480 PMC10922862

[84] F. Miege , C. Meyer , J. Cossy , Beilstein J. Org. Chem. 2011, 7, 717–734, 10.3762/bjoc.7.82. 21804867 PMC3135226

[85] C. Vidal , M. Tomás‐Gamasa , P. Destito , F. López , J. L. Mascareñas , Nat. Commun. 2018, 9, 1913, 10.1038/s41467-018-04314-5. 29765051 PMC5954130

[86] R. Vicente , Chem. Rev. 2021, 121, 162–226, 10.1021/acs.chemrev.0c00151. 32639746

[87] R. Vicente , Synthesis 2016, 48, 2343–2360, 10.1055/s-0035-1561644.

