# Supplementary material for: Site‐Selective Peptide and Protein Functionalization with Cyclopropenium Cations
Source: Angew Chem Int Ed Engl. 2025 Nov 4;64(52):e202518939. doi: 10.1002/anie.202518939 (PMC12723463; doi:10.1002/anie.202518939)
Supplement: Supplementary file 1 — Supporting Information [file ANIE-64-e202518939-s002.pdf]

Site-Selective Peptide and Protein Functionalization with Cyclopropenium Cations

Adriana Faraone[a]+, Matteo Balletti[a]+, Aliénor Jeandin[a], Hang-Fei Tu[a], Viktoria A. Ikonnikova[a], Laura S. Sojka[a] and Marcos G. Suero[a][b]*

[a] Institute of Chemical Research of Catalonia (ICIQ-CERCA), The Barcelona Institute of Science and Technology, Av. Països Catalans 16, 43007 Tarragona, Spain.

[b] ICREA, Pg Lluis Companys 23, 08010 Barcelona, Spain.

[+] Both authors contributed equally to this work.

*Corresponding author. Email: mgsuero@iciq.es

Supporting Information

Table of Contents

1. GENERAL INFORMATIONS 3

2. UHPLC-MS ANALYSIS 4

3. REVERSE PHASE COLUMN CHROMATOGRAPHY 5

4. SOLID PHASE PEPTIDE SYNTHESIS 5

5. SYNTHESIS OF CYCLOPROPENIUM CATIONS (CPCS) 6

6. CYCLOPROPENYLATION OF PEPTIDES 10

7. CYCLOPROPENYLATION OF PROTEINS 50

8. THIOL-ENE REACTION 68

9. UNSUCCESSFUL REACTIVITIES 82

10. OXYTOCIN SELECTIVITY 84

11. DECAGING OF BIOCONJUGATE & BIO-ORTHOGONAL FUNCTIONALIZATION WITH NMM ... 86

12. STABILITY OF BIOCONJUGATES 89

13. REFERENCES 93

14. COPIES OF NMR SPECTRA 94

1. General Informations

All reagents were used as purchased and used with no further purification. Ethyl diazoacetate, (≥13 wt. % dichloromethane) was purchased from Aldrich (Ref. E22201) and used without further purification. Glutathione (GHS) was purchased from Sigma-Aldrich and used without further purification. Commercially available peptides were purchased from Sigma, TargetMol, APExBIO, Bachem, TCI or their customized synthesis requested to ProteoGenix. Proteins were purchased from Center for Genomic Regulation (C/ del Dr. Aiguader, 88, Ciutat Vella, 08003 Barcelona). Anhydrous solvents were dried by passing through an activated alumina column on a PureSolvTM solvent purification system (Innovative Technologies, Inc., MA). Flash column chromatography was performed on silica gel (Aldrich, 230-400 mesh) for normal phase or prepacked C18 silica gel disposable columns (RediSep Gold® C18Aq columns, 5.5 gram) for reverse phase using Teledyne ISCO Combi*Flash* NextGen 300. The synthesis of cyclopropenium cations was carried out under argon atmosphere using standard Schlenk technique. Yields refer to purified compounds unless otherwise noted. Peptides and proteins modifications were carried out in 1.5 mL Eppendorf tubes without oxygen exclusion. The reactions were conducted in an Eppendorf Thermomixer® C equipped with temperature control and the mixing frequency set at 800 rpm. Buffers were not degassed and prepared in milliQ-Bio water. Conversions are reported as LC-MS conversions and as an average of three replicates unless otherwise stated. NMR spectra were recorded at 298 K on Bruker Avance 300, Bruker Avance 400 Ultrashield or Bruker Avance 500 Ultrashield apparatuses. Chemical shifts (δ) are quoted in ppm relative to residual solvent signals, with $CDCl_3$ referenced at δ 7.26 and 77.16 ppm, CD_3NO_2 referenced at δ 4.36 and 61.37 ppm, DMSO-d_6 referenced at δ 2.50 and 39.52 ppm, methanol-d_4 referenced at δ 3.31 and 49.00 ppm and D_2O referenced at 4.79 ppm, respectively. Coupling constants (*J*) are quoted in hertz (Hz). Multiplicity is reported with the following abbreviations: s = singlet, brs = broad singlet, d = doublet, t = triplet, q = quartet, dt = doublet of triplets, td = triplet of doublets, tt = triplet of triplets, sp = septet, m = multiplet, app = apparent. High-resolution mass spectra (HRMS) were obtained from the ICIQ High Resolution Mass Spectrometry Unit on MicroTOF Focus and Maxis Impact (Bruker Daltonics) with electrospray ionization. MALDI-TOF mass spectra were obtained on a Bruker AutoFlex mass spectrometer. Centrifugation of peptides and proteins was performed using Thermo Scientific Sorvall ST 16/16R centrifuge. Crudes were centrifuged at 0ºC and 16000 rpm unless otherwise stated. Lyophilization was performed using LyoQuest 80 Telestar apparatus.

2. UHPLC-MS Analysis

Ultra-high performance liquid chromatography-mass spectrometry (UHPLC-MS) measurements were performed on an Agilent 1290 Infinity II system equipped with a G7167B 1290 multisampler and a G7117B 1290 DAD detector, coupled with an Agilent G1958-65638 Jet Stream ESI source and an InfinityLab LC/MSD XT quadrupole mass analyzer. Separations were obtained on a Poroshell 120 EC-C18 column (2.1 x 50 mm, 1.9-micron), Agilent Zorbax SB-C18 column (2.1 x 50 mm, 1.8-micron), Agilent Zorbax 300 SB-C8 column (2.1 x 50 mm, 1.8-micron) or Agilent AdvanceBio RP-mAb SB-C8 column (2.1 x 50 mm, 3.5-micron), using UHPLC-grade water + 0.1% formic acid (solvent A) and UHPLC-MS grade acetonitrile + 0.1% formic acid (solvent B) as the mobile phase, at flow rates of 0.3-0.5 mL/min (method 1 or 2). Column temperatures were set at 30 °C. Mass spectra of peptides and proteins were acquired with the following parameters: positive Jet Stream Technology Ion Source (AJS) ionization, temperature of drying gas set at 300 °C, quadrupole temperature set at 100 °C, drying gas flow set at 3 l/min, nebulizer gas pressure set at 20 psig, capillary voltage set at 3000 V for peptides, 4000 V for proteins and fragmentor voltage set at 70 V. Condensed or full data acquisitions were stored for peptides or proteins, respectively. The acquired data were analyzed with OpenLab CDS ChemStation Edition software.

Method 1: 95:5 solvent A:B 0-2 minutes isocratic, 95:5 to 5:95 solvent A:B 2-8 minutes gradient, 5:95 solvent A:B 8-10 minutes isocratic, 10 minutes (0.25 mL/min).
Method 2: 95:5 solvent A:B 0-2 minutes isocratic, 95:5 to 5:95 solvent A:B 2-28 minutes gradient, 5:95 solvent A:B 28-30 minutes isocratic, 30 minutes (0.25 mL/min).
Method 3: 100:0 solvent 0-2 minutes isocratic, 100:0 to 5:95 solvent A:B 2-10 minutes gradient, 5:95 solvent A:B 10-12 minutes isocratic, 12 minutes (0.25 mL/min).
Method 4: 95:5 solvent 0-1 minutes isocratic, 95:5 to 75:25 solvent A:B 1-2 minutes gradient, 75:25 solvent A:B 2-8 minutes isocratic, 5:95 solvent A:B 8-10 minutes isocratic, 10 minutes. (0.25 mL/min)
Method 5: 100:0 solvent 0-1.5 minutes isocratic, 95:5 solvent A:B 1-1.5 minutes isocratic, 95:5 to 5:95 solvent A:B 1.5-10 minutes gradient. (0.1 mL/min)

3. Reverse Phase Column Chromatography

Flash column chromatography of isolated products was performed on a Combi*Flash* NextGen 300 system equipped with prepacked C18 silica RediSep Gold® C18Aq disposable columns of 5.5 grams (20-40 µm spherical particle size), using deionized water + 0.1% TFA (solvent A) and UHPLC-grade acetonitrile + 0.1% TFA (solvent B) as the mobile phase, at a flow rate of 18 mL/min (method 1). Fractions were collected based on 214 nm and 254 nm UV detector and ELSD detector.

Method 1: 95:5 solvent A:B 0-2 minutes isocratic; 95:5 to 0:100 solvent A:B 2-16 minutes gradient; 100% solvent B 16-18 minutes isocratic; 20:80 solvent A:B 18-20 minutes isocratic; 20 minutes.

Method 2: 90:10 to 70:30 solvent A:B 0-10 minutes gradient; 70:30 to 40:60 solvent A:B 10-20 minutes gradient; 95:5 to 0:100 solvent A:B 20-25 minutes isocratic, 20:80 solvent A:B 25-30 minutes isocratic, 30 minutes.

4. Solid Phase Peptide Synthesis

Peptides that were not purchased were prepared in house on an AAPPTec Focus XC 1.0 peptide synthesizer system using standard Fmoc-SPPS chemistry and Novabiochem® 2-chlorotrityl chloride resins (0.76-0.79 mmol/g resin, 0.20 mmol scale) coupled with Fmoc-protected C-terminal amino acids for final C-terminal free carboxylic acid. Fmoc deprotection was carried out with 20% v/v piperidine in dimethylformamide for 2 minutes three times. The coupling was carried out pre-activating for 5 minutes the next Fmoc-protected amino acid (6.0 equiv. with respect to the resin loading, from a 0.25 M solution in DMF) with N,N′-diisopropylcarbodiimide (DIC, 6.0 equiv. with respect to the resin loading and 1.0 equiv. with respect to the Fmoc-protected amino acid, from a 0.5 M solution in DMF) and ethyl cyano(hydroxyimino)acetate (Oxyma Pure, 6.0 equiv. with respect to the resin loading and 1.0 equiv. with respect to the Fmoc-protected amino acid, from a 1 M solution in DMF) for 1 hour. The peptide cleavage and deprotection was carried out with 94: 1: 2.5: 2.5 % v/v of trifluoroacetic acid (TFA): triisopropylsilane (TIPS): water: ethane

dithiol (EDT) for 30 minutes. The resin was filtered off and the peptides were precipitated with cold diethyl ether, centrifuged for 1 minute, the supernatant removed and the precipitates washed with cold diethyl ether three times. The crude peptides were resuspended in a 90:10 mixture of water: acetonitrile + 0.1% TFA and dried under vacuum overnight. The crude peptides were used in the next step without further purification.

5. Synthesis of Cyclopropenium Cations (CPCs)

General procedure A for the synthesis of CPCs:

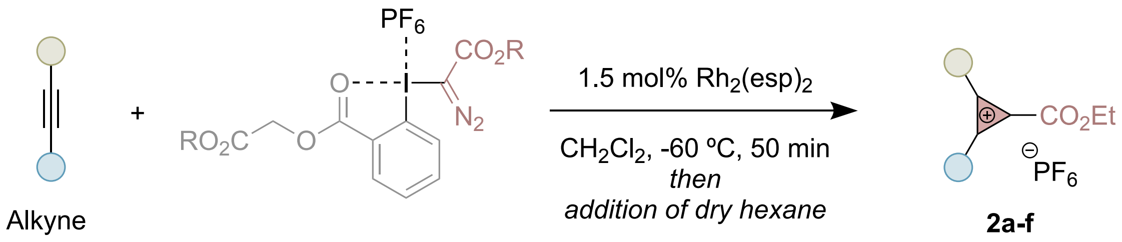

The **CPC**s were synthesized according to a previously reported procedure.[1] To a 100 mL oven-dried flask equipped with a stirring bar was added $Rh_2(esp)_2$ (20 mg, 1.5 mol%). The flask was sealed before being evacuated and backfilled with Argon (three cycles). Degassed anhydrous dichloromethane (5 mL) and the corresponding alkyne (2.6 mmol, 1.3 equiv.) were added and the resulting mixture was cooled to -60 ºC. Then, a solution of the corresponding hypervalent iodine reagent (2.0 mmol, 1.0 equiv.) in degassed anhydrous dichloromethane (11 mL) was added dropwise during 40 minutes using a syringe pump. After the addition, the reaction was kept stirring at -60 ºC until the reaction was complete (monitored by TLC, 10 minutes). Then anhydrous hexane (20 mL) was added to the reaction mixture slowly to induce precipitation of the cation. The solid product was collected by quick filtration on porous frit and washed with dry hexane (8 mL x 3), dried under high vacuum to give the corresponding **CPC**.

*Note: the **CPC**s were stored at –30 ºC under argon.*

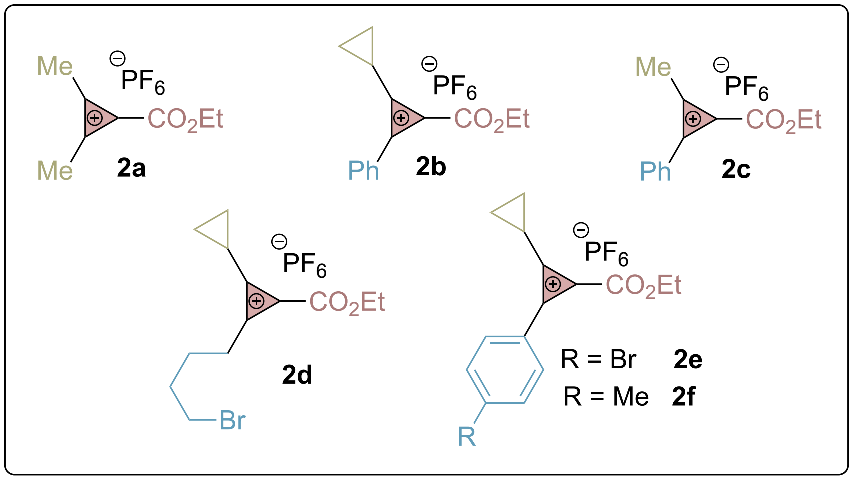

Figure S1: CPCs used in this work.

Yields and characterization data of **CPC** compounds **2a**, **2b** and **2c** were in accordance with the ones reported in the literature.[1] Compounds **2d**, **2e** and **2f** were previously unreported, below follows the characterization of these compounds and the relative yields.

Characterization data of novel CPCs:

1-(ethoxycarbonyl)-2-(4-bromobutyl)-3-cyclopropylcyclopropenium hexafluorophosphate (CPC 2d)

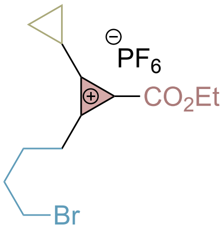

Prepared according to the ***general procedure A*** using (1-diazo-2-ethyloxy-2-oxoethyl)(2-(2-ethyloxy-2-oxoethoxyl)carbonylphenyl)iodonium hexafluorophosphate (808 mg, 1.36 mmol) and (6-bromohex-1-yn-1-yl)cyclopropane alkyne (356 mg, 1.77 mmol). In this case addition of hexane induced the precipitation of the expected compound **2d** as a dark red oil (313 mg, 48% yield, 90% purity).

^{1}H NMR (400 MHz, CD_3NO_2) δ 4.59 (q, J = 7.1 Hz, 2H), 3.59 (t, J = 6.3 Hz, 2H), 3.48 (t, J = 7.0 Hz, 2H), 2.99 – 2.87 (m, 1H), 2.33 – 2.03 (m, 8H), 1.46 (t, J = 7.1 Hz, 3H).

^{13}C NMR (101 MHz, CD_3NO_2) δ 182.3, 173.8, 156.1, 152.5, 65.7, 32.8, 31.3, 26.8, 23.8, 20.6, 12.7, 11.5.

^{19}F NMR (376 MHz, CD_3NO_2) δ -73.6 (d, J = 706.9 Hz).

^{31}P NMR (162 MHz, CD_3NO_2) δ -141.63 (sp, J = 711.4 Hz).

HRMS (ESI) calculated for $C_{13}H_{18}BrO_2^+$ $[M\text{-}PF_6]^+$ m/z: 285.0485, found: 285.0488.

1-(ethoxycarbonyl)-2-(4-bromphenyl)-3-cyclopropylcyclopropenium hexafluorophosphate (CPC 2e)

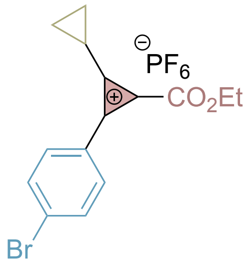

Prepared according to the ***general procedure A*** using (1-diazo-2-ethyloxy-2-oxoethyl)(2-(2-ethyloxy-2-oxoethoxyl)carbonylphenyl)iodonium hexafluorophosphate (592 mg, 1 mmol) and (6-bromohex-1-yn-1-yl)cyclopropane alkyne (372 mg, 1.71 mmol). Filtration of the reaction mixture provided the title compound **2e** as an off-white solid (321 mg, 71% yield).

^{1}H NMR (400 MHz, CD_3NO_2) δ 8.31 (d, J = 8.2 Hz, 2H), 8.08 (d, J = 8.5 Hz, 2H), 4.65 (q, J = 7.1 Hz, 2H), 3.18 – 3.09 (m, 1H), 2.39 – 2.30 (m, 2H), 2.26 – 2.20 (m, 2H), 1.50 (t, J = 7.1 Hz, 1H).

^{13}C NMR (101 MHz, CD_3NO_2) δ 178.5, 163.1, 155.4, 149.9, 140.9, 139.4, 136.9, 120.4, 68.6, 23.6, 15.7, 14.8.

^{19}F NMR (376 MHz, CD_3NO_2) -73.62 (d, J = 707.2 Hz).

^{31}P NMR (162 MHz, CD_3NO_2) δ -141.67 (sp, J = 712.1 Hz).

HRMS (ESI) calculated for $C_{15}H_{14}BrO_2^+$ $[M\text{-}PF_6]^+$ m/z: 305.0177, found: 305.0172.

1-(ethoxycarbonyl)-2-(4-tolyl)-3-cyclopropylcyclopropenium hexafluorophosphate (CPC 2f)

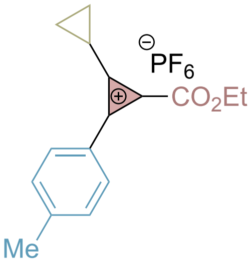

Prepared according to the ***general procedure A*** using (1-diazo-2-ethyloxy-2-oxoethyl)(2-(2-ethyloxy-2-oxoethoxyl)carbonylphenyl)iodonium hexafluorophosphate (592 mg, 1.00 mmol) and (6-bromohex-1-yn-1-yl)cyclopropane alkyne (190 mg, 1.33 mmol). Filtration of the reaction mixture provided the title compound **2f** as a beige solid (185 mg, 76% yield).

^{1}**H NMR** (400 MHz, CD_3NO_2) δ 8.31 (d, J = 8.1 Hz, 2H), 7.73 (d, J = 7.9 Hz, 2H), 4.64 (q, J = 7.1 Hz, 2H), 3.10 (tt, J = 7.7, 4.0 Hz, 1H), 2.64 (s, 3H), 2.30 – 2.28 (m, 2H), 2.25 – 2.15 (m, 2H), 1.51 (t, J = 7.1 Hz, 3H).

^{13}**C NMR** (101 MHz, CD_3NO_2) δ 174.59, 160.01, 154.26, 152.74, 145.76, 137.20, 131.32, 115.70, 65.49, 21.59, 19.88, 12.79, 11.44.

^{19}**F NMR** (376 MHz, CD_3NO_2) δ -73.65 (d, J = 706.9 Hz).

^{31}**P NMR** (162 MHz, CD_3NO_2) δ -141.67 (sp, J = 711.4 Hz).

HRMS (ESI) calculated for $C_{16}H_{17}O_2^+$ $[M-PF_6]^+$ m/z: 241.1228, found: 241.1223

6. Cyclopropenylation of Peptides

Preliminary result: reaction with protected cysteine

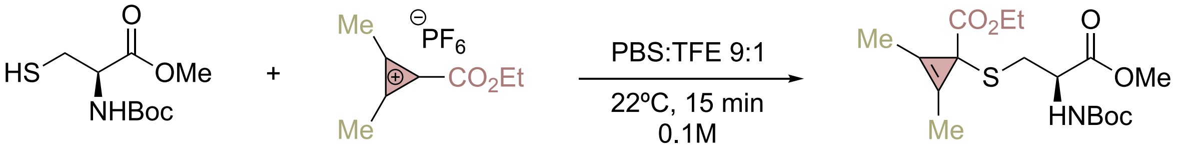

Boc-Cys-OMe (1 equiv.) **CPC 2a** (1.2 equiv.) **3** 91% yield

To a 1.5 mL Eppendorf tube were added Boc-Cys-OMe (24.3 mg, 0.10 mmol), 900 µL of PBS (phosphate buffered saline) 10 mM, 100 µL of trifluoroethanol (TFE) as cosolvent to reach a 100 mM concentration of protected cysteine and the **CPC 2a** (34 mg, 0.12 mmol). The tube was vortexed few seconds and stirred at room temperature for 15 minutes. The aqueous phase was extracted twice with ethyl acetate (2 x 2 mL). The organic phase was dried over anhydrous $MgSO_4$ and filtered. Purification of the crude product by flash column chromatography on silica gel (gradient hexane: EtOAc from 9:1 to 7:3) provided the title compound **3** (34 mg, 91% yield) as a colorless oil.

S-[1-(ethoxycarbonyl)-2,3-dimethylcyclopropenyl]-N-tert-butoxycarbonyl-cysteine methyl ester (3)

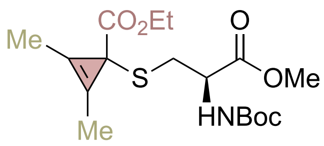

^{1}H NMR (400 MHz, $CDCl_3$) δ 5.66 (d, J = 7.9 Hz, 1H), 4.50 (q, J = 5.9 Hz, 1H), 4.17 (q, J = 7.1 Hz, 2H), 3.76 (s, 3H), 3.16 – 2.93 (m, 2H), 2.10 (s, 6H), 1.46 (s, 9H), 1.28 (t, J = 7.1 Hz, 3H).
^{13}C NMR (101 MHz, $CDCl_3$) δ 173.5, 171.6, 155.4, 109.6, 108.9, 79.9, 77.2, 61.5, 53.5, 52.5, 33.8, 28.3, 14.3, 8.9, 8.6.
HRMS (ESI) calculated for $C_{17}H_{27}NNaO_6S^+$ $[M-Na]^+$ m/z: 396.1451, found: 396.1453.
$[\alpha]_D^{23}$ = -15.1 (c = 0.1, MeCN).

Procedure for the optimization of the reaction using glutathione (GSH):

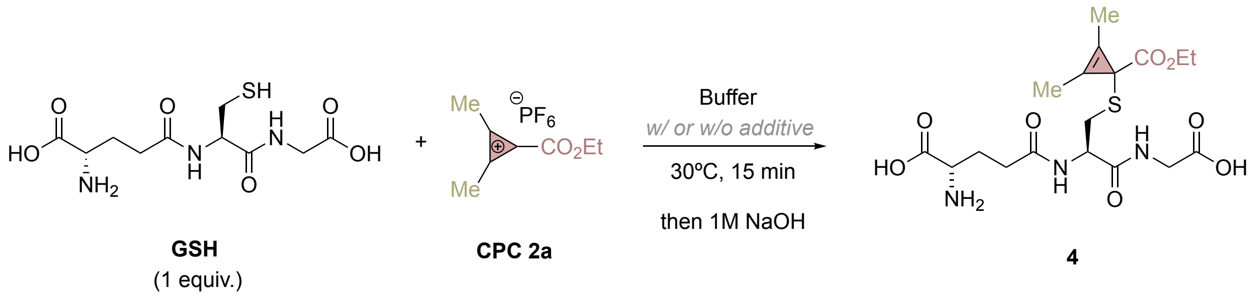

To a 1.5 mL Eppendorf tube were added 90 µL of buffer, 10 µL of glutathione (GSH) stock solution in water and the corresponding **CPC 2a** neat. The tube was vortexed for 10 seconds and stirred in an Eppendorf thermomixer at the selected temperature for the corresponding time. After that, the pH of the mixture was adjusted to neutral (measured with pH strips) with 1 M NaOH solution and the aqueous phase washed twice with 200 µL of ethyl acetate. The organic phase was discarded after 1-3 minutes centrifugation. Sulisobenzone was used as Internal Standard for the UHPLC-MS calibration following the method reported by Kanai et Al.[2] A known amount of sulisobenzone aqueous solution was added to the reaction crude as internal standard and the mixture filtered and analyzed by reverse phase UHPLC-MS. The analytical yields reported for each entry were determined from a calibration curve obtained using previously isolated compound **4** *versus* sulisobenzone as the internal standard (IS).

*Procedure for the isolation of the glutathione bioconjugate **4**:*

S-[1-(ethoxycarbonyl)-2,3-dimethylcyclopropenyl]glutathione (trifluoroacetate salt of 4)

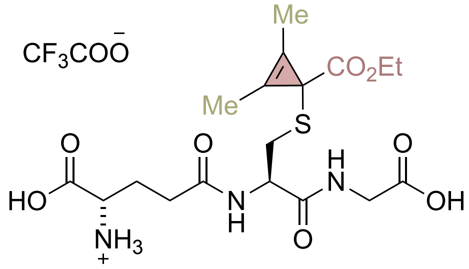

Prepared according to the ***general procedure B*** using GSH (144 mg, 0.47 mmol), milliQ water (4.7 mL for 0.1 M GSH concentration) and **CPC 2a** (280 mg, 0.98 mmol, 2 equiv.). Purification

of the crude mixture by flash column chromatography on C18 silica gel (*method 1* in ***section 3***) provided the title compound **4** as a white solid (171 mg, 82% yield).

^{1}H NMR (300 MHz, D_2O) δ 4.50 (dd, *J* = 8.8, 5.1 Hz, 1H), 4.18 (q, *J* = 7.3 Hz, 2H), 4.10 (t, *J* = 6.6 Hz, 1H), 4.01 (s, 2H), 3.12 (dd, *J* = 13.8, 5.1 Hz, 1H), 2.92 (dd, *J* = 13.8, 8.9 Hz, 1H), 2.61 (td, *J* = 7.5, 3.5 Hz, 2H), 2.33 – 2.17 (m, 2H), 2.10 (t, *J* = 1.3 Hz, 3H), 2.08 (t, *J* = 1.3 Hz, 3H), 1.24 (t, *J* = 7.1 Hz, 3H).

^{13}C NMR (126 MHz, D_2O) δ 176.1, 174.9, 173.9, 171.8, 171.0, 109.0, 108.6, 63.0, 54.1, 53.9, 43.3, 37.5, 32.5, 31.4, 26.1, 13.4, 7.62, 7.55.

^{19}F NMR (282 MHz, D_2O) δ -75.6.

HRMS (ESI) calculated for $C_{18}H_{26}N_3O_8S^-$ $[M\text{-}H\text{-}CF_3COOH]^-$ m/z: 444.1446, found: 444.1448.

$[\alpha]_D^{23}$ = -8.9 (c = 0.1, MeCN).

UHPLC-MS analysis using column Zorbax 300 SB-C8 over 10 minutes (*method 1* in ***section 2***, $R_{t\ product}$ = 3.8 min):

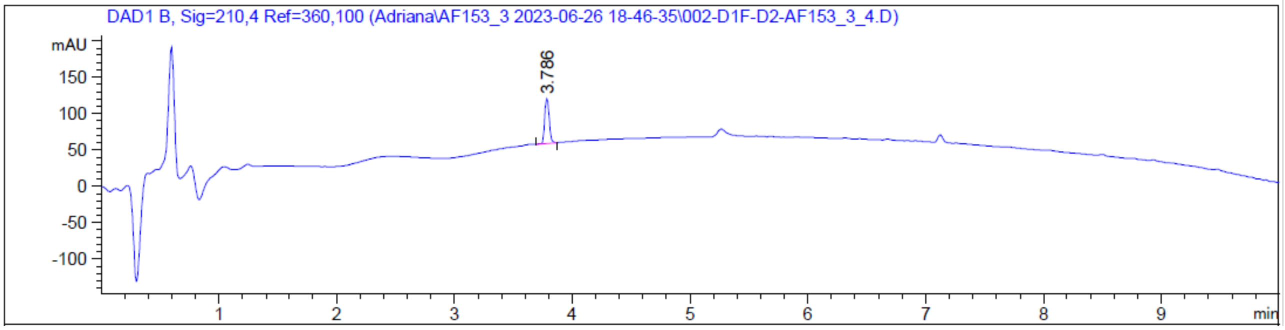

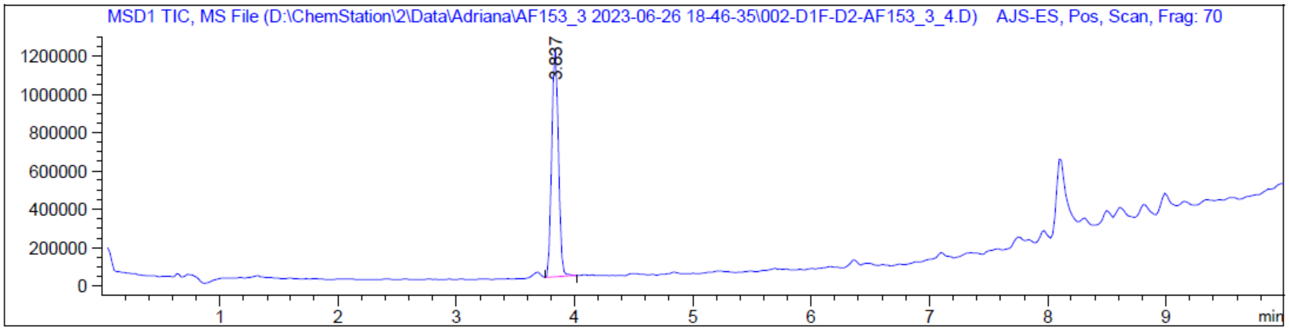

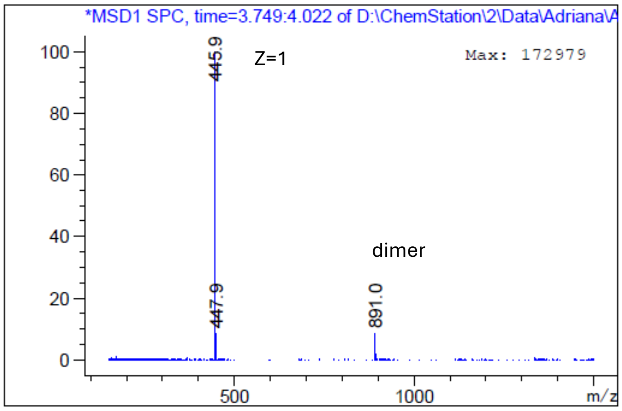

Peak #1 at 3.837 min (3.749 to 4.017 min)

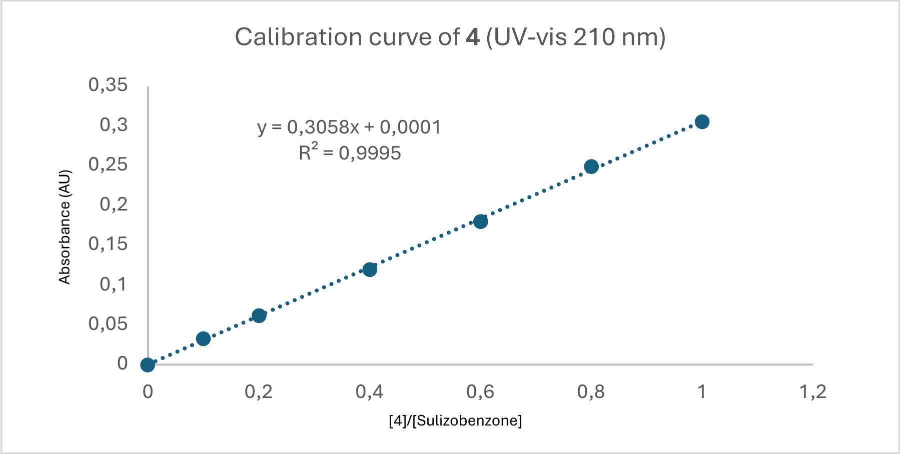

UHPLC calibration curve obtained on column Zorbax SB-C18 over 12 minutes ($R_{t\ product}$ = 6.4 min, *method 3* in ***section 2***).

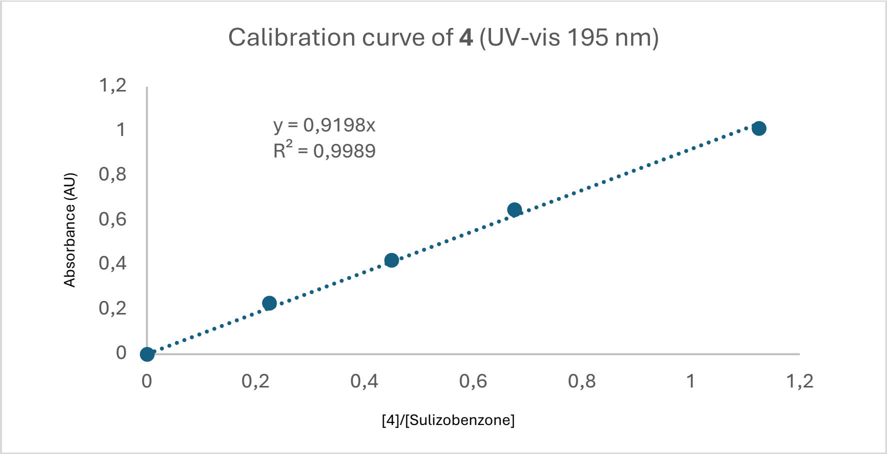

UHPLC calibration curve obtained on column Zorbax 300 SB-C8 over 10 minutes ($R_{t\ product}$ = 3.8 min, *method 1* in ***section 2***).

Selected optimization studies

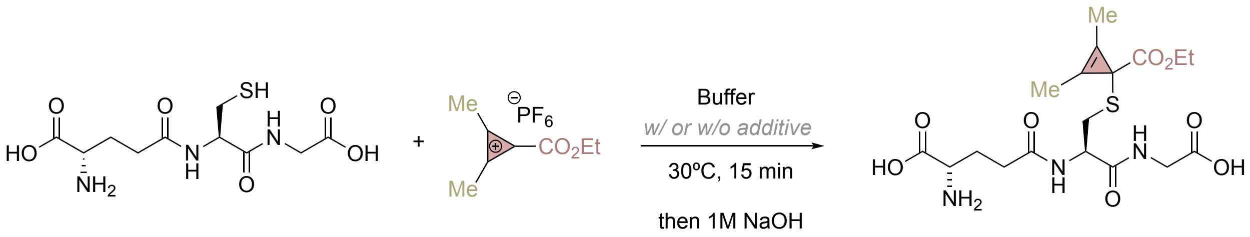

Entry	**2a** equiv.	Buffer	Additive	Concentration/scale	Analytical yield **4** (%)
1	3	PBS 10 mM	10% TFE	100 mM/0.02 mmol	>99[a]
2	3	PBS 10 mM	no	100 mM/0.02 mmol	>99[a]
3	3	no (in water)	no	100 mM/0.02 mmol	>99[a]
4	2	PBS 10 mM	10% TFE	100 mM/0.01 mmol	>99
5	1	PBS 10 mM	10% TFE	100 mM/0.01 mmol	52
6	3	PBS 10 mM	10% TFE	50 mM/0.01 mmol	95[b]
7	3	PBS 10 mM	10% TFE	10 mM/0.01 mmol	21[b]
8	3	PBS 10 mM	10% TFE	1 mM/0.001 mmol	0
9	20	PBS 10 mM	10% TFE	1 mM/0.001 mmol	traces
10	50	PBS 10 mM	10% TFE	1 mM/0.1 µmol	5
11	100	PBS 10 mM	10% TFE	1 mM/0.1 µmol	53
12	100	PBS 10 mM	no	1 mM/0.1 µmol	65
13	100	PBS 100 mM	10% TFE	1 mM/0.1 µmol	18
14	100	HEPES 10 mM	10% TFE	1 mM/0.1 µmol	50
15	100	NH_4OAc 10 mM, pH 6.1	10% TFE	1 mM/0.1 µmol	47
16	100	TRIS 10 mM	10% TFE	1 mM/0.1 µmol	45
17	100	Na_3PO_4 10 mM, pH 9.5	10% TFE	1 mM/0.1 µmol	42

Table S1. Preliminary optimization studies for the selective cyclopropenylation of GSH at the cysteine residue.aNMR yields in D_2O using sulisobenzone as internal standard. bBoth NMR and analytical yields in accordance. PBS = phosphate buffered saline, pH 7.4; TFE = trifluoroethanol; HEPES = 4-(2-hydroxyethyl)-1-piperazineethanesulfonic acid buffer, pH 7.4; TRIS = tris(hydroxymethyl)aminomethane buffer, pH 8.6.

Entry	**2a** equiv.	Temperature/time	Additive	Concentration/scale	Analytical yield **4** (%)
1	100	30 ºC, 15 min	10% HFIP	1 mM/0.1 μmol	55
2	100	30 ºC, 15 min	10% ACN	1 mM/0.1 μmol	41
3	100	30 ºC, 15 min	10% DMF	1 mM/0.1 μmol	53
4	100	30 ºC, 5 min	no	1 mM/0.1 μmol	51
5	100	30 ºC, 30 min	no	1 mM/0.1 μmol	57
6	100	30 ºC, 1 hour	no	1 mM/0.1 μmol	54
7	100	30 ºC, 4 hours	no	1 mM/0.1 μmol	52
8	100	5 ºC, 15 min	no	1 mM/0.1 μmol	58
9	100	10 ºC, 15 min	no	1 mM/0.1 μmol	61
10	100	37 ºC, 15 min	no	1 mM/0.1 μmol	75
11	100	50 ºC, 15 min	no	1 mM/0.1 μmol	69
**12**	**20**	**37 ºC, 15 min**	**no**	**5 mM/0.5 μmol**	**>99**
13	10	37 ºC, 15 min	no	5 mM/0.5 μmol	46
14	20	37 ºC, 15 min	no	5 mM/0.5 μmol	66[a]
15	10	37 ºC, 15 min	no	5 mM/0.5 μmol	15[a]

Table S2. [a]citric acid/citrate buffer 100 mM, pH 3 was used. PBS = phosphate buffered saline, pH 7.4; HFIP = hexafluoroisopropanol.

UHPLC-MS analysis for entry 12 in **Table S2** using column Zorbax SB-C18 over 12 minutes (*method 3* in ***section 2***, $R_{t\ product}$ = 6.4 min, $R_{t\ IS}$ = 6.8 min):

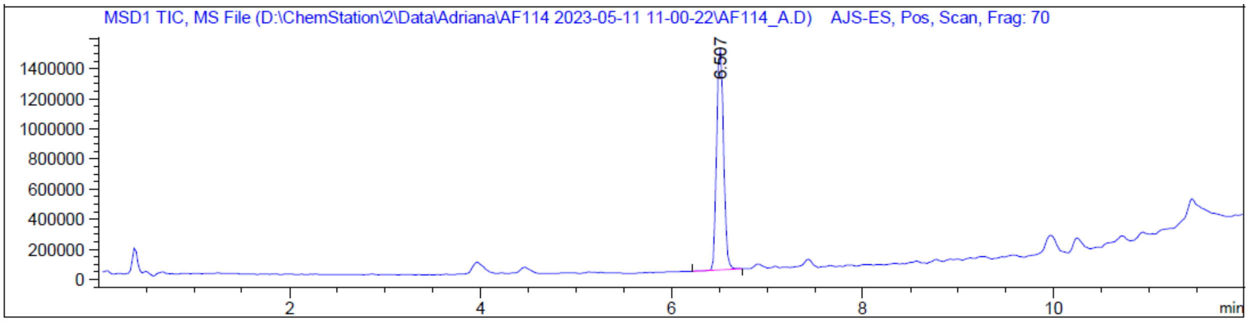

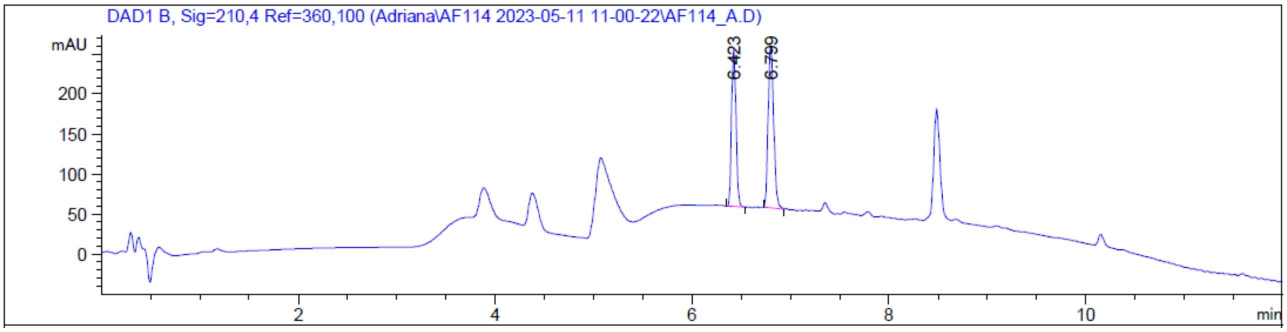

Signal 1: DAD1 B, Sig=210,4 Ref=360,100

Peak #	RetTime [min]	Type	Width [min]	Area [mAU*s]	Height [mAU]	Area %
1	6.423	BB	0.0514	635.39008	197.95140	44.5338
2	6.799	BB	0.0617	791.36945	201.90030	55.4662
Totals :				1426.75952	399.85170	

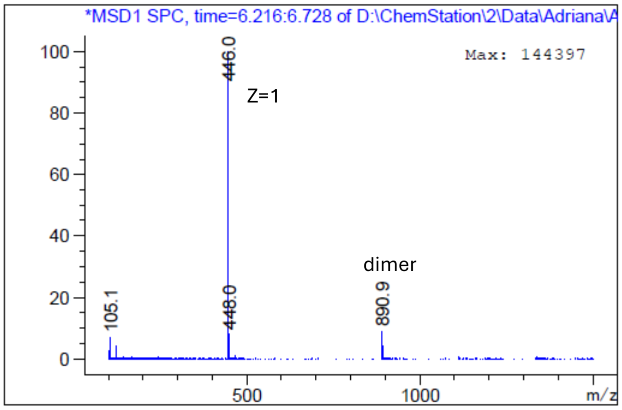

Peak #1 at 6.507 min (6.216 to 6.736 min)

General procedure B: reaction under concentrated conditions for the isolation of glutathione bioconjugates

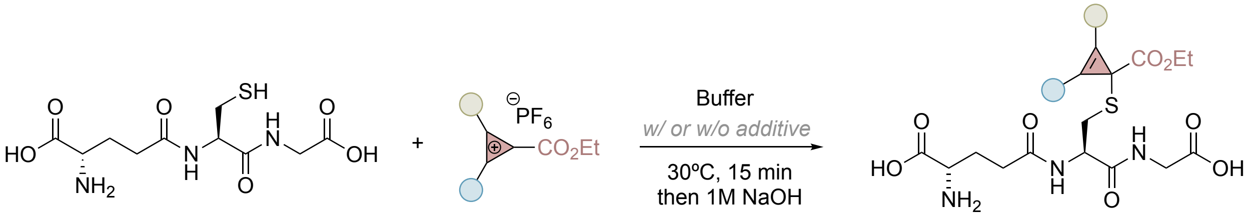

GSH CPC 2a-f 5-9

To a 5 mL Eppendorf tube equipped with a stirring bar were added glutathione (GSH, 1 equiv.), milliQ water or PBS (phosphate buffered saline) 10 mM to reach a 100 mM concentration and the corresponding **CPC** (neat). The tube was vortexed 10 seconds and stirred at room temperature for the corresponding time, until no further conversion was observed from UHPLC-MS analysis. After that, the pH of the mixture was adjusted to neutral (measured with pH strips) with 1 M NaOH solution and the aqueous phase was washed twice with ethyl acetate. The organic phase was

allowed to separate over 1 minute of centrifugation and discarded. The crude mixture from the aqueous phase was dried under vacuum, separated by reverse phase flash column chromatography as detailed in ***section 3***, and dried by lyophilization to afford the trifluoroacetate salt of the title compound.

Characterization data of isolated bioconjugates

S-[1-(Ethoxycarbonyl)-2-(4-bromobutyl)-3-cyclopropylcyclopropenyl]glutathione (trifluoroacetate salt of 5)

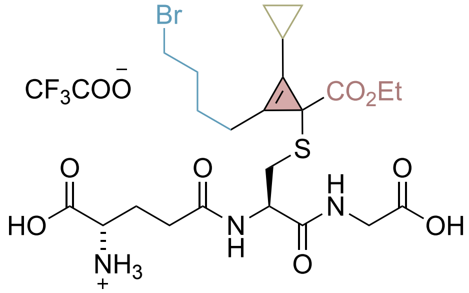

Prepared according to the ***general procedure B*** using GSH (101 mg, 0.33 mmol), PBS buffer 10 mM (2.31 mL) and **CPC 2d** (438 mg, 1.02 mmol, 3 equiv.) dissolved in 990 µL of trifluoroethanol as organic cosolvent (30% of total volume). The aqueous phase was subjected directly to flash column chromatography on C18 silica gel (*method 1* in ***section 3***) without performing organic solvent washes. The purification provided the title compound **5** an off-white solid (114.6 mg, 59% yield, dr 1:1).

^{1}H NMR (400 MHz, D_2O, *diast1+diast2*) δ 4.52 (app td, *J* = 9.1, 5.1 Hz, 1H), 4.26 – 4.15 (m, 2H), 4.06 – 3.95 (m, 3H), 3.55 (t, *J* = 6.5 Hz, 2H), 3.09 (app ddd, *J* = 13.7, 5.2, 2.9 Hz, 1H), 2.92 (app ddd, *J* = 14.1, 8.9, 5.8 Hz, 1H), 2.72 – 2.49 (m, 4H), 2.23 (app q, *J* = 7.4 Hz, 2H), 2.01 – 1.85 (m, 3H), 1.80 – 1.68 (m, 2H), 1.26 (app td, *J* = 7.1, 2.0 Hz, 3H), 1.09 – 0.96 (m, 2H), 0.79 – 0.69 (m, 1H), 0.67 – 0.58 (m, 1H).

^{13}C NMR (101 MHz, D_2O, *diast1+diast2*) δ 175.8, 174.4, 172.9, 172.6, 172.4, 114.5, 114.3, 109.6, 109.4, 63.2, 53.8, 53.7, 52.9, 41.1, 37.3, 37.2, 34.3, 32.7, 32.6, 31.5, 31.0, 25.7, 25.0, 24.9, 22.5, 22.4, 13.5, 6.8, 6.7, 6.4, 4.3, 4.3. (10C missing due to overlap).

^{19}F NMR (376 MHz, D_2O, d1+d2) δ -75.7.

HRMS (ESI) calculated for $C_{23}H_{35}BrN_3O_8S^+$ [M-CF_3COO]$^+$ m/z: 592.1323, found: 592.1350.

UHPLC-MS analysis using column Zorbax 300 SB-C8 over 10 minutes (*method 1* in ***section 2***, $R_{t\ product}$ = 5.6 min):

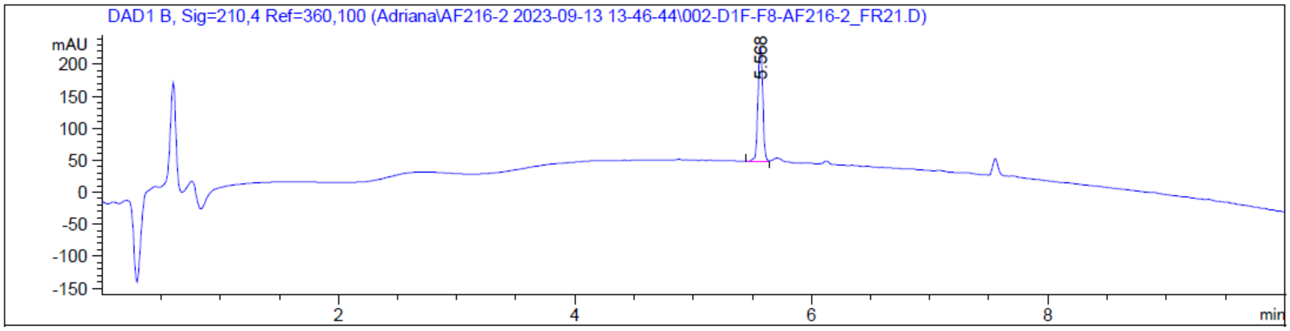

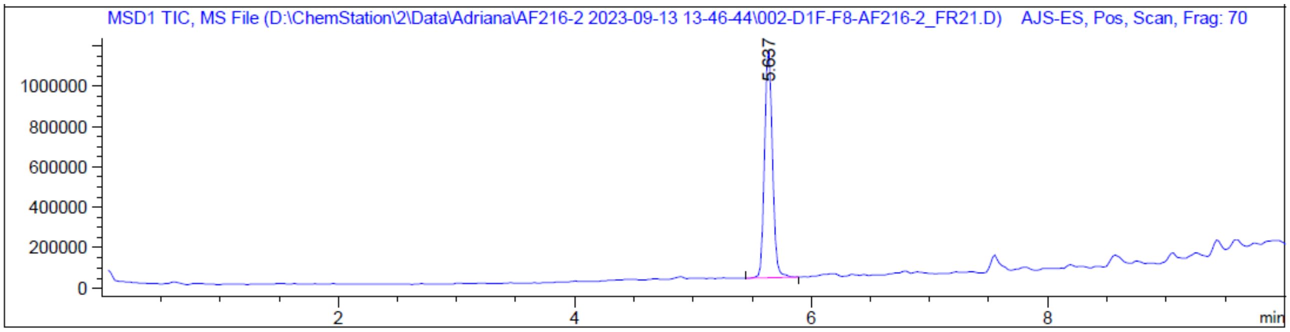

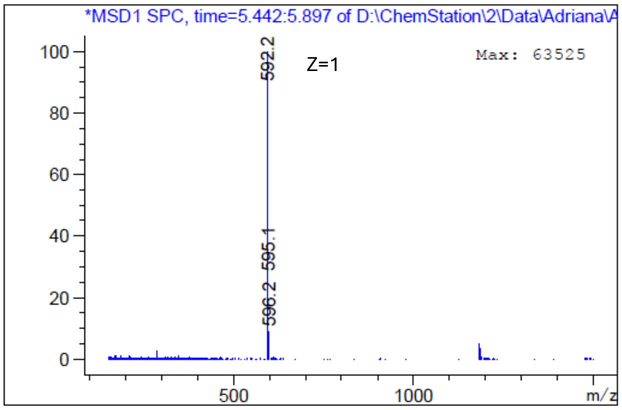

Peak #1 at 5.637 min (5.442 to 5.897 min)

S-[1-(Ethoxycarbonyl)-2-phenyl-3-cyclopropylcyclopropenyl]glutathione (trifluoroacetate salt of 6)

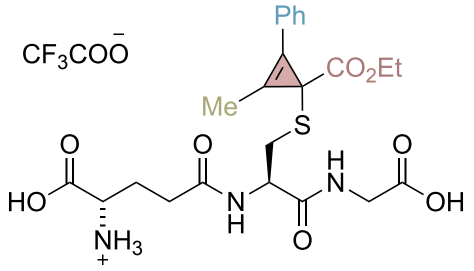

Prepared according to the ***general procedure B*** using GSH (39 mg, 0.126 mmol), PBS buffer 10 mM (882 μL) and **CPC 2c** (87 mg, 0.253 mmol, 2 equiv.) dissolved in 378 μL of MeCN as organic cosolvent (30% of total volume). The aqueous phase was washed with diethyl ether instead of ethyl acetate. Some product was lost during this step. Purification of the crude mixture by flash column chromatography on C18 silica gel (*method 1* in ***section 3***) provided the title compound **6** as an off-white solid (108 mg, 70% yield, dr 1:1).

^{1}H NMR ^{1}H NMR (500 MHz, D_2O, *diast1+diast2*) δ 7.50 – 7.34 (m, 5H), 4.33 (ddd, *J* = 29.7, 8.4, 5.3 Hz, 1H), 4.15 – 4.03 (m, 2H), 3.95 (t, *J* = 6.6 Hz, 1H), 3.87 – 3.79 (m, 2H), 2.98 (ddd, *J* = 18.9, 14.0, 5.3 Hz, 1H), 2.80 (ddd, *J* = 14.0, 8.4, 3.9 Hz, 1H), 2.40 (q, *J* = 7.8 Hz, 2H), 2.30 (d, *J* = 6.0 Hz, 3H), 2.12 – 2.03 (m, 2H), 1.22 – 0.98 (m, 3H).

^{13}C NMR (126 MHz, D_2O, *diast1+diast2*) δ 175.3, 175.2, 174.1, 174.1, 172.8, 172.4, 171.6, 171.6, 130.3, 130.2, 129.3, 129.2, 124.5, 124.4, 117.5, 115.2, 112.2, 111.9, 110.1, 109.8, 63.2, 53.7, 53.7, 52.3, 41.0, 37.4, 37.3, 32.4, 32.0, 30.9, 30.8, 25.5, 25.5, 13.4, 8.7, 8.6. (6C missing due to overlap).

^{19}F NMR (376 MHz, D^2O, *diast1+diast2*) δ -75.6.

HRMS (ESI) calculated for $C_{23}H_{29}N_3NaO_8S^+$ $[M-CF_3COOH+Na]^+$ m/z: 530.1573, found: 530.1631.

UHPLC-MS analysis using column Zorbax 300 SB-C8 over 10 minutes (*method 1* in ***section 2***, $R_{t\ product}$ = 5.1 min):

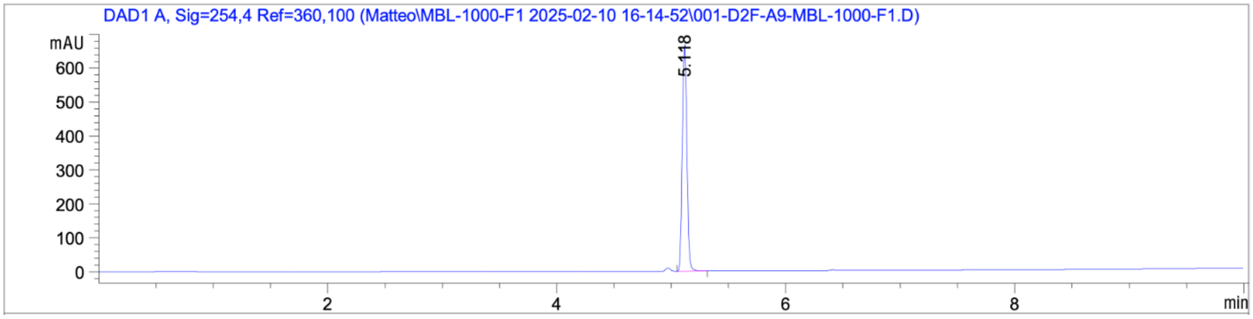

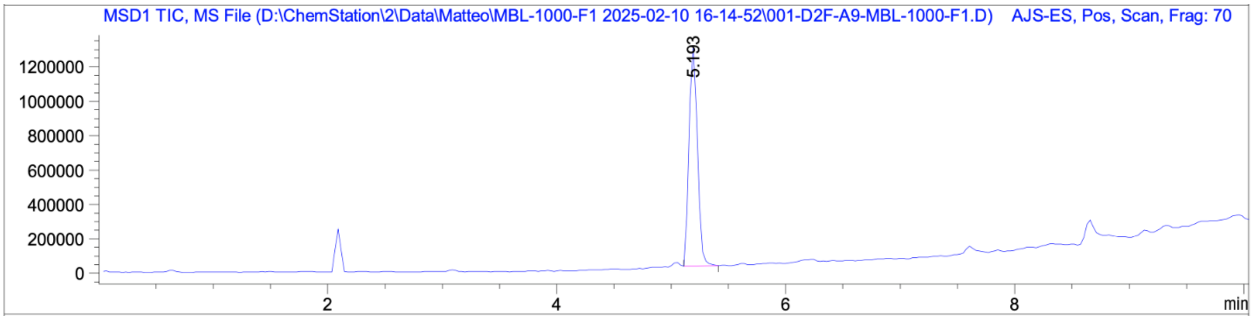

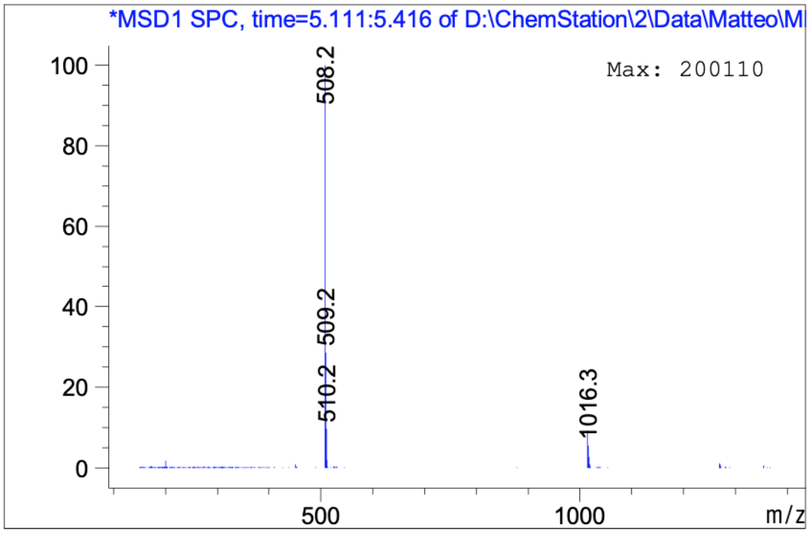

Peak #1 at 5.193 min (5.111 to 5.412 min)

S-[1-(Ethoxycarbonyl)-2-phenyl-3-cyclopropylcyclopropenyl]glutathione (trifluoroacetate salt of 7)

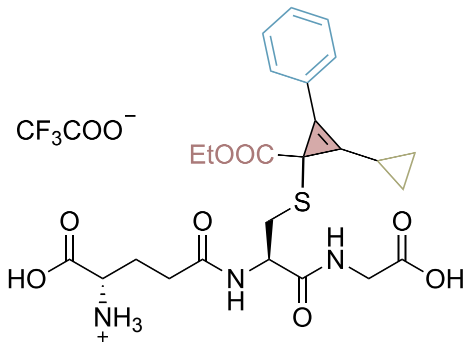

Prepared according to the ***general procedure B*** using GSH (39 mg, 0.126 mmol), PBS buffer 10 mM (882 µL) and **CPC 2b** (94.2 mg, 0.253 mmol, 2 equiv.) dissolved in 378 µL of MeCN as organic cosolvent (30% of total volume). The aqueous phase was washed with diethyl ether instead of ethyl acetate. Some product was lost during this step. Purification of the crude mixture by flash column chromatography on C18 silica gel (*method 1* in ***section 3***) provided the title compound **7** as an off-white solid (42 mg, 52% yield, dr 1:1).

^{1}H NMR (400 MHz, MeOD, *diast1+diast2*) δ 7.53 – 7.35 (m, 10H, d1+d2), 4.54 (dd, *J* = 8.5, 5.5 Hz, 1H), 4.50 (dd, *J* = 8.7, 5.1 Hz, 1H), 4.23 – 4.06 (m, 4H, d1+d2), 3.98 (t, *J* = 6.3 Hz, 2H, d1+d2), 3.90 (d, *J* = 1.1 Hz, 1H), 3.88 (d, *J* = 0.9 Hz, 1H), 3.07 (dd, *J* = 13.3, 5.2 Hz, 1H), 3.01 (dd, *J* = 13.3, 5.5 Hz, 1H), 2.89 (dd, *J* = 13.3, 8.7 Hz, 1H), 2.89 (dd, *J* = 13.3, 8.5 Hz, 1H), 2.60 – 2.47 (m, 4H, d1+d2), 2.27 – 2.08 (m, 6H, d1+d2), 1.18 (t, *J* = 7.1 Hz, 3H, d1+d2), 1.21 – 1.11 (m, 4H, d1+d2), 1.03 – 0.94 (m, 2H, d1+d2), 0.92 – 0.82 (m, 2H, d1+d2).

^{13}C NMR (101 MHz, MeOD, *diast1+diast2*) δ 174.9, 174.8, 174.4, 174.3, 173.01, 172.9, 172.6, 171.8, 130.4, 130.2, 130.1, 130.0, 127.1, 127.0, 119.3, 119.1, 109.6, 109.5, 66.9, 62.91, 62.88, 55.0, 53.7, 41.8, 38.5, 38.5, 34.6, 34.5, 32.4, 27.1, 15.4, 14.6, 8.8, 8.8, 8.5, 8.5, 6.9. (7C missing due to overlap).

^{19}F NMR (376 MHz, MeOD, *diast1+diast2*) δ -77.0.

HRMS (ESI) calculated for $C_{25}H_{31}N_3NaO_8S^+$ $[M-CF_3COOH+Na]^+$ m/z: 556.1724, found: 556.1722.

UHPLC-MS analysis using column Zorbax 300 SB-C8 over 10 minutes (*method 1* in ***section 2***, $R_{t\ product}$ = 5.3 min):

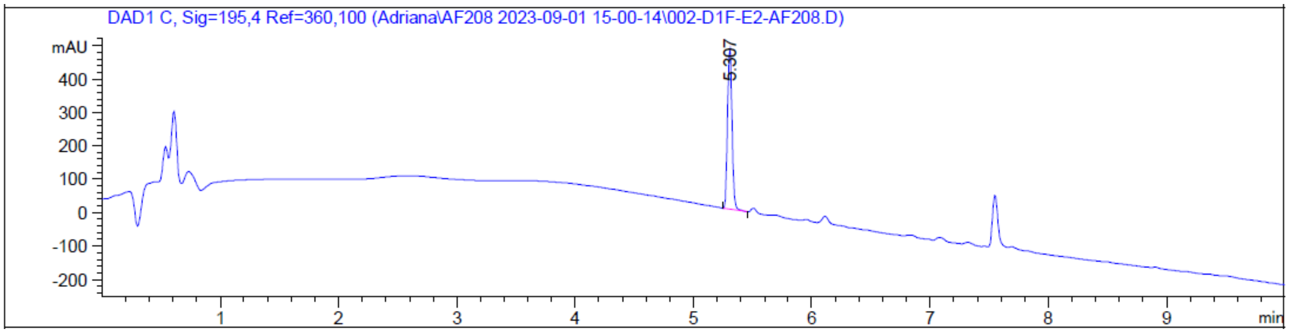

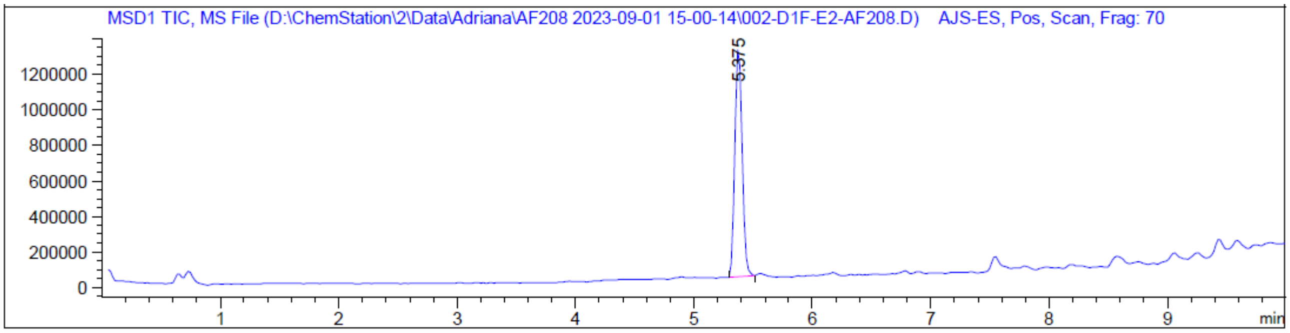

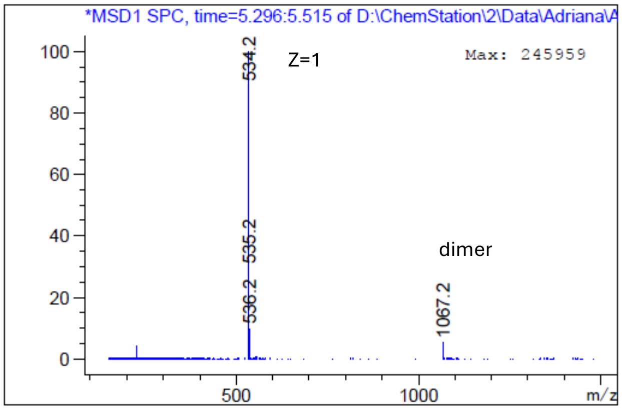

Peak #1 at 5.375 min (5.296 to 5.514 min)

S-[1-(Ethoxycarbonyl)-2-(4-Tolyl)-3-cyclopropylcyclopropenyl]glutathione (trifluoroacetate salt of 8)

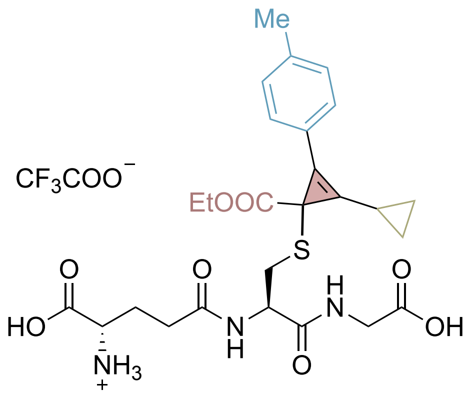

Prepared according to the ***general procedure B*** using GSH (60 mg, 0.2 mmol), PBS buffer 10 mM (2.31 mL) and **CPC 2f** (154 mg, 0.4 mmol, 2 equiv.) dissolved in 990 μL of MeCN as organic cosolvent (30% of total volume). The aqueous phase was subjected directly to flash column chromatography on C18 silica gel (*method 2* in ***section 3***) without performing organic solvent washes. The purification provided the title compound **8** as an off-white solid (80 mg, 61% yield, dr 1:1).

^{1}H NMR (400 MHz, D_2O, *diast1+diast2*) δ 7.38 – 7.33 (m, 2H), 7.28 – 7.22 (m, 2H), 4.25 (ddd, *J* = 25.3, 8.4, 5.2 Hz, 1H), 4.16 – 4.04 (m, 2H), 3.83 – 3.71 (m, 3H), 2.95 – 2.81 (m, 1H), 2.80 – 2.66 (m, 1H), 2.37 – 2.29 (m, 2H), 2.27 (s, 3H), 2.10 – 1.95 (m, 3H), 2.14 – 1.00 (m, 5H), 0.93 – 0.83 (m, 1H), 0.75– 0.67 (m, 1H).

^{13}C NMR (101 MHz, D_2O, *diast1+diast2*) δ 174.6, 174.6, 174.1, 174.1, 172.8, 172.5, 172.2, 172.2, 140.3, 129.9, 128.9, 128.9, 122.0, 121.8, 117.5, 116.4, 116.3, 115.2, 107.0, 106.8, 62.8, 55.6, 53.8, 53.8 52.9, 52.8, 41.1, 37.1, 32.4, 32.1, 31.1, 31.0, 25.8, 25.6, 25.3, 20.7, 13.5, 13.4, 7.9, 7.7, 5.6, 5.6. (4C missing due to overlap).

^{19}F NMR (376 MHz, D_2O, *diast1+diast2*) δ -75.62.

HRMS (ESI) calculated for $C_{26}H_{33}N_3O_8S^+$ $[M\text{-}CF_3COO]^+$ m/z: 547.2054, found: 547.2073.

UHPLC-MS analysis using column Zorbax 300 SB-C8 over 10 minutes (*method 1* in ***section 2***, $R_{t\ product}$ = 5.63 min):

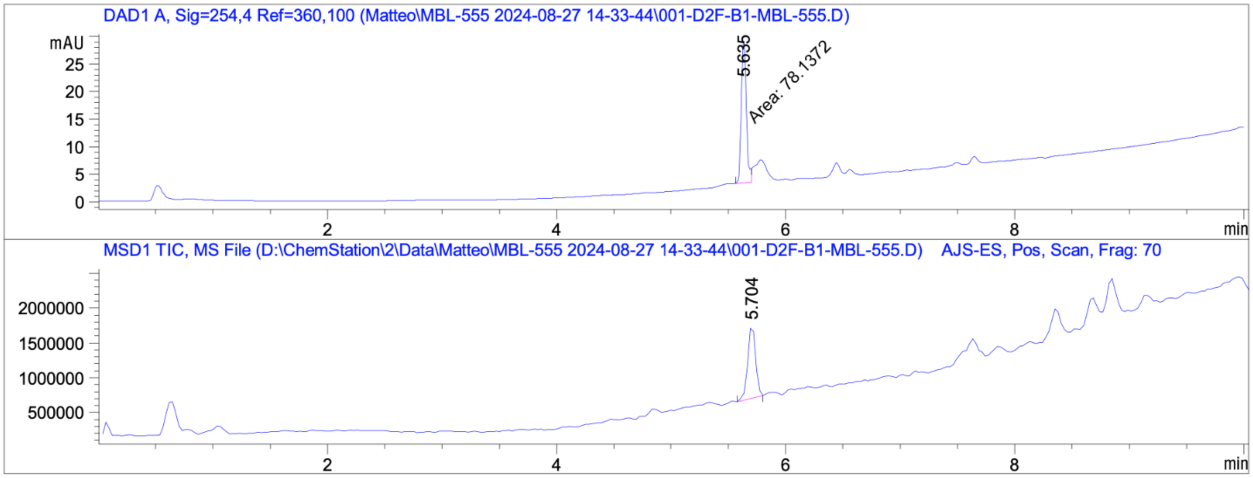

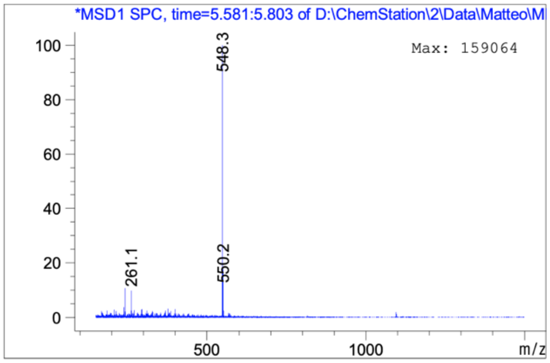

Peak #1 at 5.704 min (5.581 to 5.803 min)

S-[1-(Ethoxycarbonyl)-2-(4-Bromophenyl)-3-cyclopropylcyclopropenyl]glutathione (trifluoroacetate salt of 9)

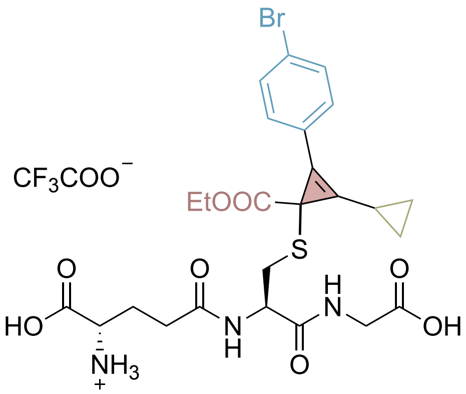

Prepared according to the ***general procedure B*** using GSH (101 mg, 0.33 mmol), PBS buffer 10 mM:MeCN=1:1 (3,5 mL) and **CPC 2e** (451 mg, 1.02 mmol, 3 equiv.). The aqueous phase was

subjected directly to flash column chromatography on C18 silica gel (*method 2* in ***section 3***) without performing organic solvent washes. The purification provided the title compound **9** as an off-white solid (160 mg, 67% yield, dr 1:1).

^{1}H NMR (500 MHz, D2O, *diast1+diast2*) δ 7.40 (d, *J* = 8.1 Hz, 2H), 7.23 (dd, *J* = 8.4, 1.6 Hz, 2H), 4.37 – 4.28 (m, 1H), 4.08 – 3.98 (m, 2H), 3.95 (m, 1H), 3.91 – 3.75 (m, 2H), 2.90 (m, 1H), 2.75 (m 1H), 2.42 (q, *J* = 7.6 Hz, 1H), 2.18 – 1.94 (m, 3H), 1.02 (m, 5H), 0.91 – 0.75 (m, 2H).

^{13}C NMR (101 MHz, D_2O, *diast1+diast2*) δ 173.1, 173.0, 173.0, 172.9, 171.6, 171.5, 171.2, 170.1, 132.0, 130.3, 130.2, 124.9, 124.8, 122.9, 119.2, 119.0, 107.4, 107.3, 61.6, 61.6, 53.6, 53.6, 52.2, 40.4, 40.4, 37.0, 36.9, 33.1, 33.0, 31.0, 25.6, 13.1, 7.6, 7.5, 7.2, 7.2, 5.6, 5.6. (6C missing due to overlap).

^{19}F NMR (376 MHz, D_2O, *diast1+diast2*) δ -76.8.

HRMS (ESI) calculated for $C_{25}H_{31}BrN_3O_8S^+$ [M-CF_3COO]$^+$ m/z: 612.1010, found: 612.1017.

UHPLC-MS analysis using column Zorbax 300 SB-C8 over 10 minutes (*method 1* in ***section 2***, $R_{t\ product}$ = 6.85 min):

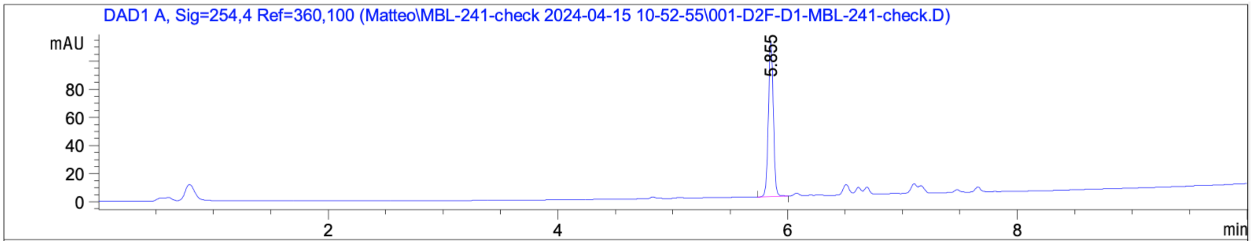

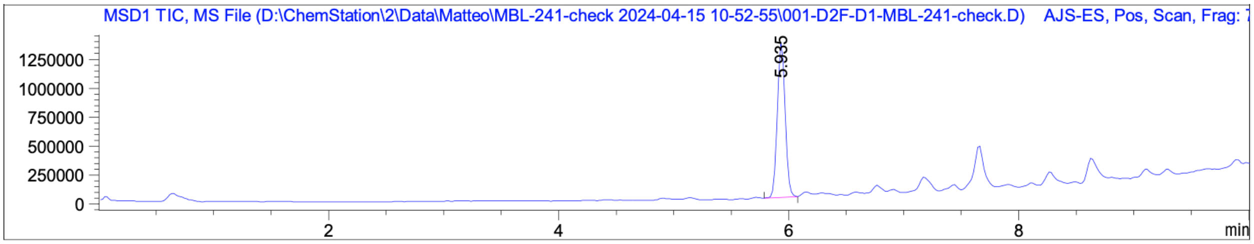

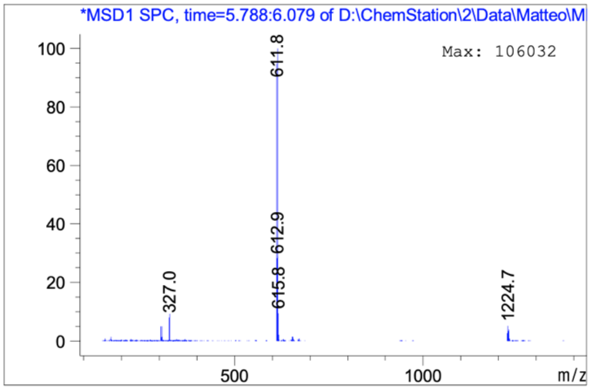

Peak #1 at 5.935 min (5.788 to 6.078 min)

<u>*Procedure for the isolation of the trifluoroacetate salt of compound*</u> **10**

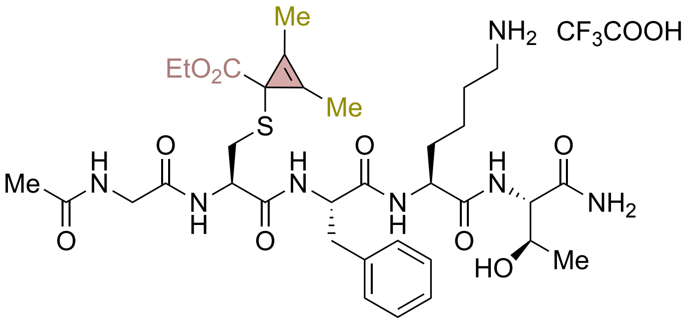

Prepared according to the ***general procedure B*** using commercial peptide Ac-GCFKT-NH_2 (2.5 mg, 0.004 mmol), PBS buffer 10 mM (419 µL for 10 mM peptide concentration) and **CPC 2a** (23.8 mg, 0.08 mmol, 20 equiv.) over 30 minutes of stirring at 37 ºC. Purification of the crude mixture by flash column chromatography on C18 silica gel (*method 1* in ***section 3***) provided the trifluoroacetate salt of the title compound **10** as a white solid (0.6 mg, 20% yield).

^{1}H NMR (500 MHz, D_2O) δ 7.43 – 7.23 (m, 5H), 4.70 (dd, *J* = 9.0, 5.8 Hz, 1H), 4.39 (ddd, *J* = 7.6, 6.0, 3.3 Hz, 2H), 4.31 (d, *J* = 4.0 Hz, 1H), 4.29 – 4.22 (m, 1H), 4.17 (q, *J* = 7.2 Hz, 2H), 3.91 (s, 2H), 3.23 (dd, *J* = 14.0, 5.8 Hz, 1H), 3.04 (dd, *J* = 14.0, 9.1 Hz, 1H), 3.00 (app t, *J* = 7.7 Hz, 2H), 2.87 (dd, *J* = 13.8, 6.3 Hz, 1H), 2.75 (dd, *J* = 13.7, 7.7 Hz, 1H), 2.10 – 2.04 (m, 9H), 1.91 – 1.80 (m, 1H), 1.79 – 1.63 (m, 3H), 1.47 – 1.33 (m, 2H), 1.27 – 1.20 (m, 6H).

^{13}C NMR (126 MHz, D_2O) δ 176.0, 174.9, 174.2, 173.6, 172.7, 171.8, 171.6, 136.1, 129.2, 128.7, 127.2, 108.8, 108.6, 67.0, 63.0, 58.6, 54.7, 53.7, 53.5, 42.6, 39.1, 37.6, 36.6, 32.1, 30.3, 26.2, 21.9, 21.7, 18.8, 13.5, 7.7, 7.6.

^{19}**F NMR** (376 MHz, D_2O) δ -75.7.

HRMS (ESI) calculated for $C_{34}H_{52}N_7O_9S^+$ [M-CF_3COO]$^+$ m/z: 734.3542, found: 734.3541.

TOCSY spectroscopy allowed to unequivocally assign the protons of the cysteine and lysine side chains as depicted below (**Figure S2**).

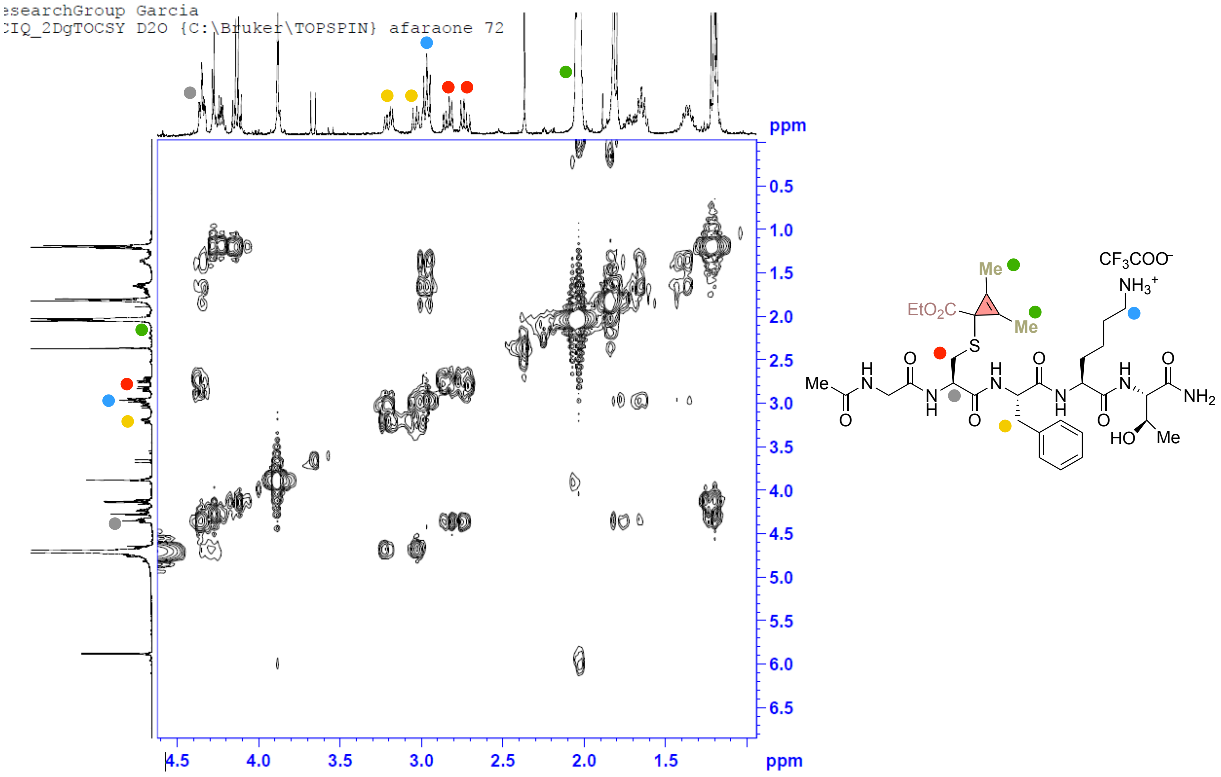

Figure S2. TOCSY spectroscopy of sample **10** (trifluoroacetate salt) in D_2O at 400 MHz.

ROESY spectroscopy showed a weak nuclear Overhauser effect between the protons of the cysteine side chain and the methyl substituents of the cyclopropene core (**Figure S3**). The absence of the same observation for the protons of the lysine side chain suggested that the introduction of the cyclopropene ring occurred selectively on the cysteine residue in the presence of lysine.

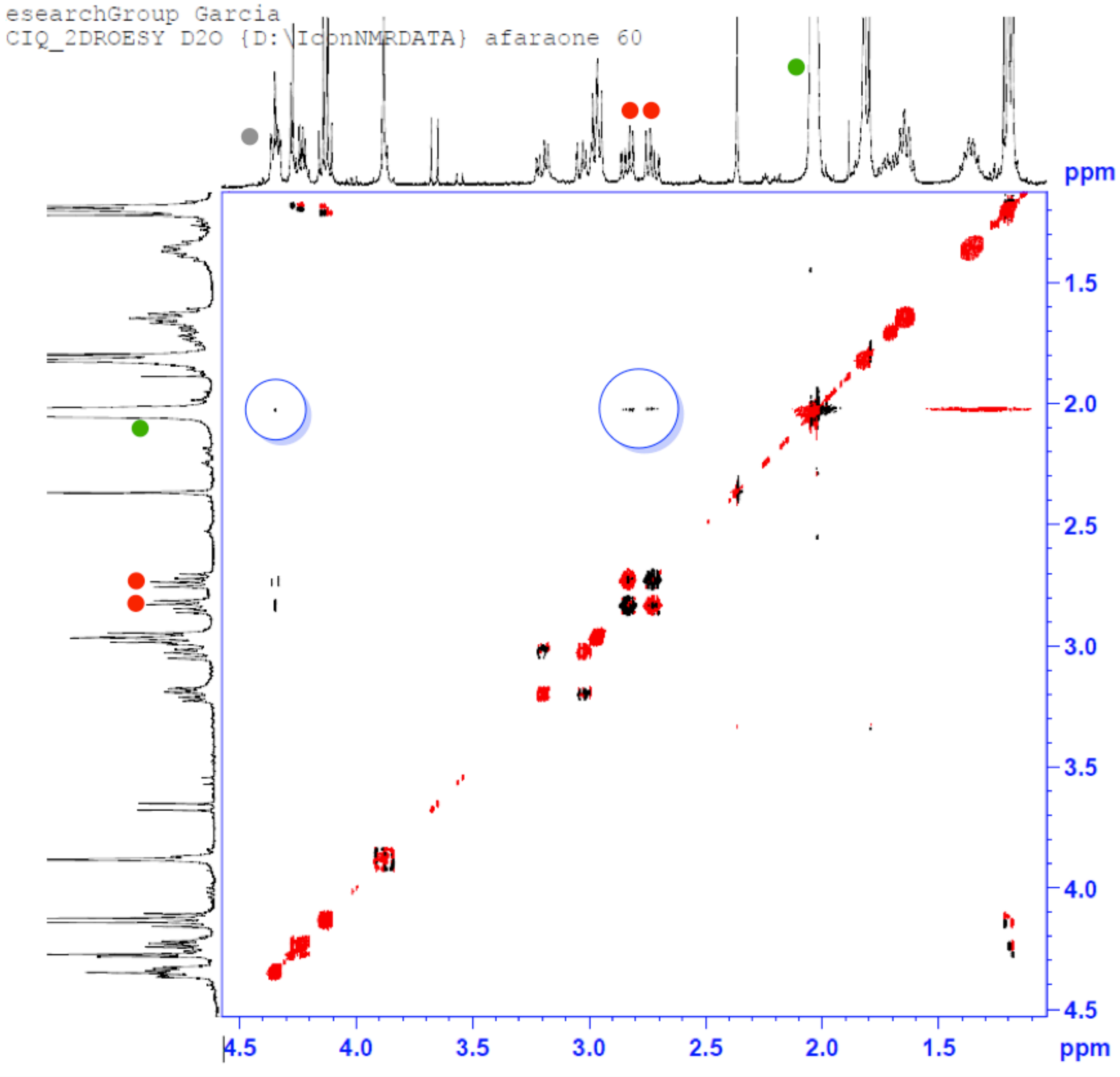

Figure S3. ROESY spectroscopy of sample **10** (trifluoroacetate salt) in D_2O at 400 MHz.

General procedure C: bioconjugation reaction under diluted conditions (GSH):

To a 1.5 mL Eppendorf tube were added the amount of PBS buffer 10 mM needed to reach a final volume of 100 µL, 10 µL of 10 or 50 mM glutathione (GSH) stock solution in water and the corresponding **CPC** neat or dissolved in the proper amount of organic solvent as additive to improve the solubilization of the **CPC** compound in water. The tube was vortexed 10 seconds and stirred in an Eppendorf thermomixer at 37 ºC for the corresponding time. After that, the aqueous phase was washed twice with 200 µL of cold diethyl ether. The organic phase was discarded and a known amount of sulisobenzone aqueous solution was added to the reaction crude as the internal standard. The mixture was filtered with PVDF Spheros Filters and analyzed by reverse phase UHPLC-MS. The analytical yields reported for each entry were determined from a calibration curve obtained using previously isolated trifluoroacetate salts of the bioconjugate versus sulisobenzone as the internal standard.

S-[1-(Ethoxycarbonyl)-2-phenyl-3-cyclopropylcyclopropenyl]glutathione (hexafluorophosphate salt of 7)

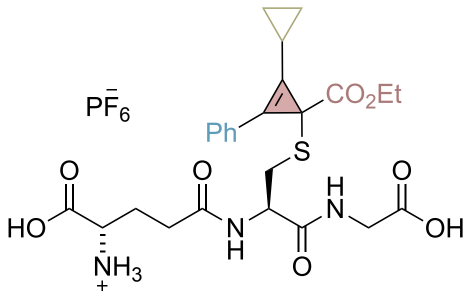

Obtained according to the ***general procedure C*** at 5 mM concentration using **CPC 2c** (3.7 mg, 20 equiv.) dissolved in 10 µL of trifluoroethanol as organic cosolvent (10% of total volume). The crude mixture was filtered and analyzed by reverse phase UHPLC-MS after 45 minutes of stirring and the title compound was detected as a 1:1 mixture of diastereomers in 70% analytical yield (R_t = 5.0 min), according to the calibration curve given below.

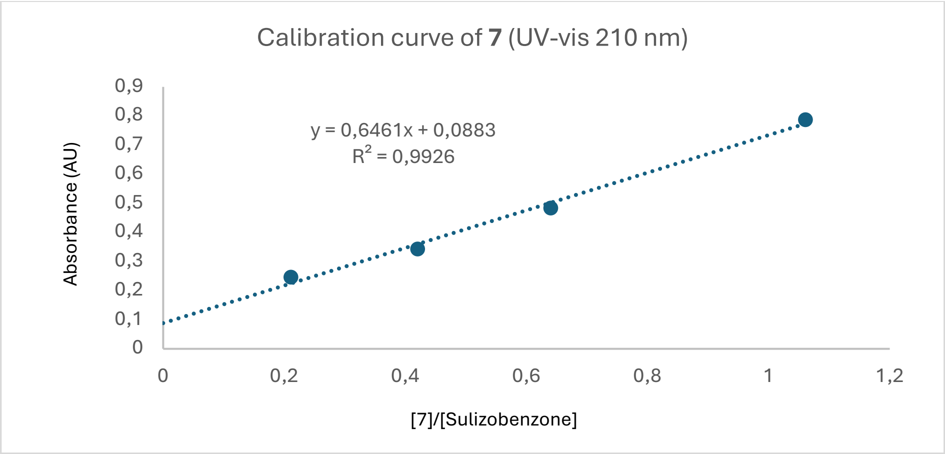

UHPLC calibration curve obtained on column Zorbax 300 SB-C8 over 10 minutes (*method 1* in ***section 2***).

UHPLC-MS analysis using column Zorbax 300 SB-C8 over 10 minutes (*method 1* in ***section 2***, $R_{t\ product}$ = 5.0 min, $R_{t\ IS}$ = 4.2 min):

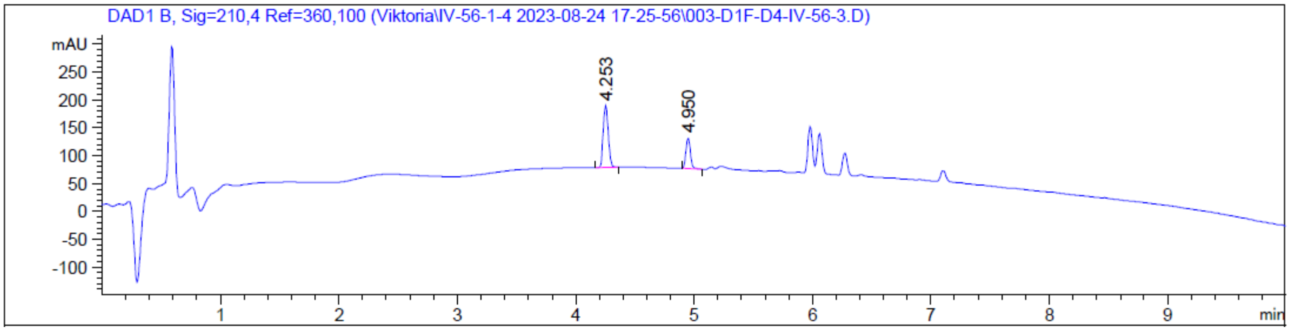

Signal 1: DAD1 B, Sig=210,4 Ref=360,100

Peak #	RetTime [min]	Type	Width [min]	Area [mAU*s]	Height [mAU]	Area %
1	4.253	BB	0.0466	328.56564	110.93745	69.2330
2	4.950	BB	0.0432	146.01416	54.52545	30.7670
Totals :				474.57980	165.46290	

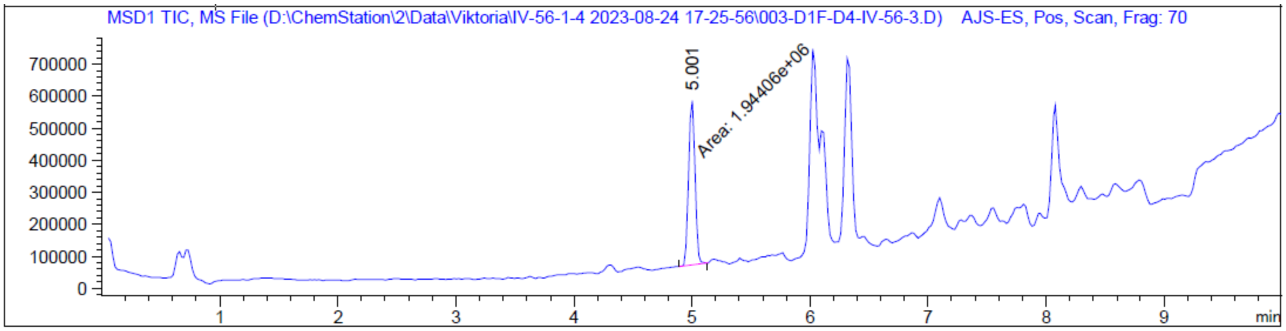

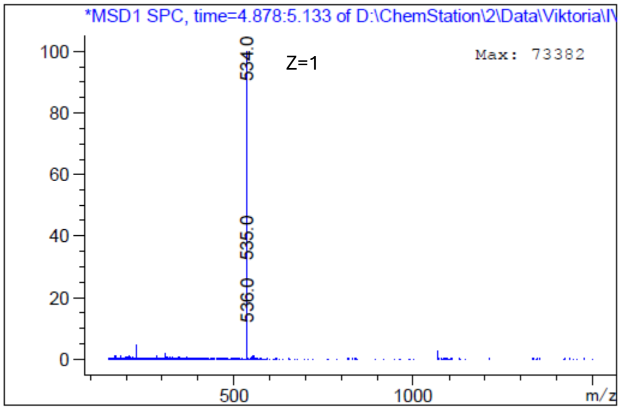

Peak #1 at 5.001 min (4.883 to 5.131 min)

S-[1-(Ethoxycarbonyl)-2-(4-bromobutyl)-3-cyclopropylcyclopropenyl]glutathione (hexafluorophosphate salt of 5)

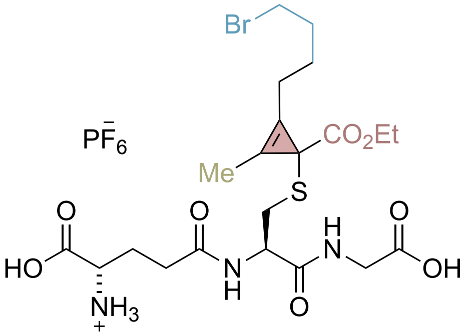

Obtained according to the ***general procedure C*** at 5 mM concentration using **CPC 2d** (4.3 mg, 20 equiv.) dissolved in 30 µL of trifluoroethanol as organic cosolvent (30% of total volume). In this case, the aqueous phase washes with diethyl ether were not performed. The crude mixture was filtered and analyzed by reverse phase UHPLC-MS after 2.5 hours of stirring and the title compound was detected as a 1:1 mixture of diastereomers in 87% analytical yield (R_t = 9.3 min),

according to the calibration curve given below. The product was obtained in 60% analytical yield when only 10 equivalents of **CPC 2d** were used.

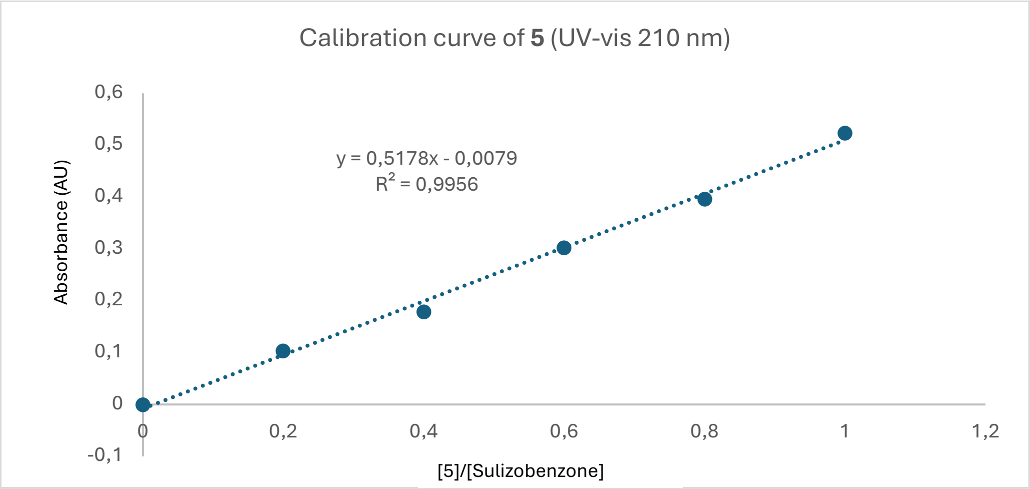

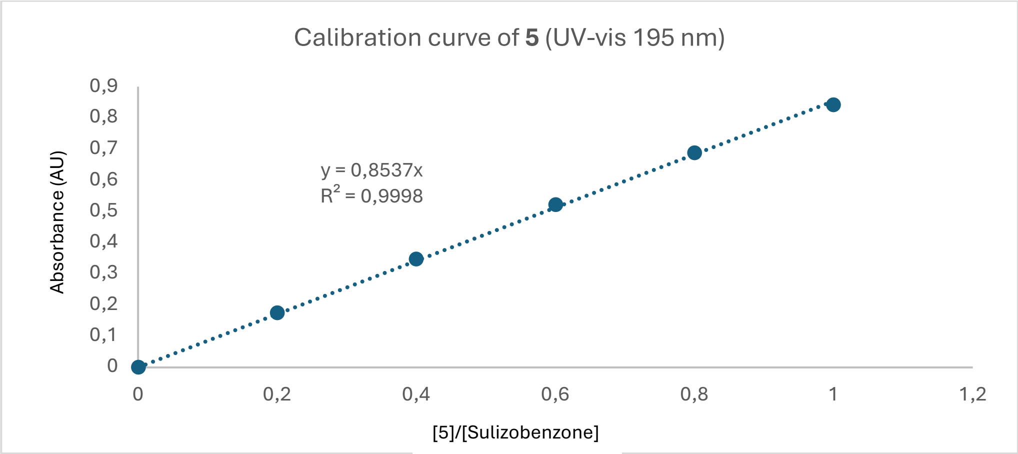

UHPLC calibration curve obtained on column Zorbax 300 SB-C8 over 30 minutes (*method 2* in ***section 2***).

UHPLC-MS analysis using column Zorbax 300 SB-C8 over 30 minutes (*method 2* in ***section 2***, $R_{t\ product}$ = 9.3 min, $R_{t\ IS}$ = 5.4 min):

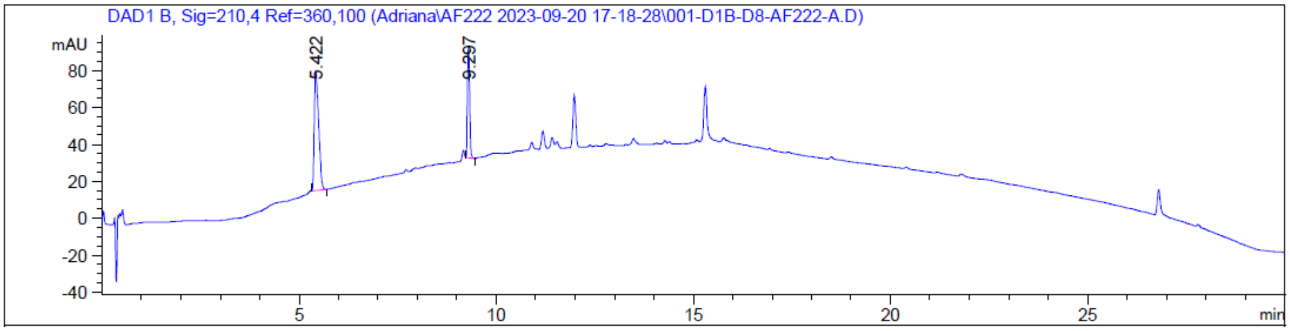

Signal 1: DAD1 B, Sig=210,4 Ref=360,100

Peak #	RetTime [min]	Type	Width [min]	Area [mAU*s]	Height [mAU]	Area %
1	5.422	BB	0.1177	488.84518	65.03772	68.1662
2	9.297	BB	0.0583	228.29192	60.10806	31.8338

Totals : 717.13710 125.14578

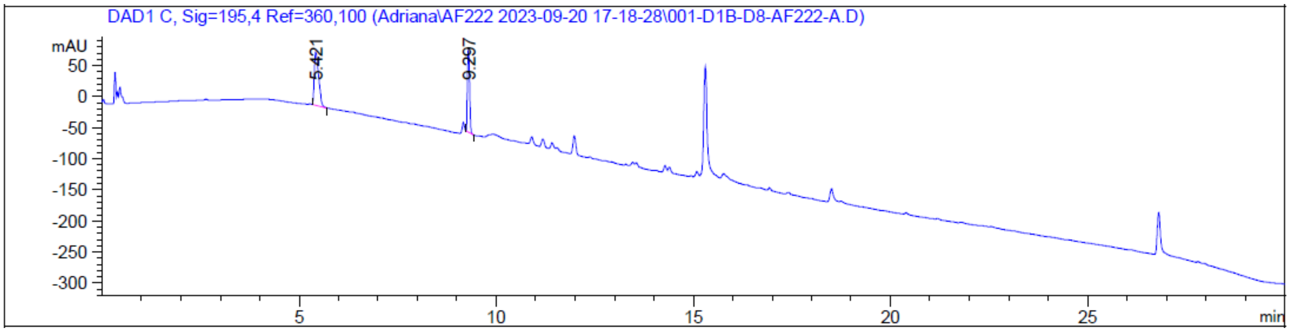

Signal 2: DAD1 C, Sig=195,4 Ref=360,100

Peak #	RetTime [min]	Type	Width [min]	Area [mAU*s]	Height [mAU]	Area %
1	5.421	BB	0.1158	648.02765	86.15023	56.2232
2	9.297	BB	0.0575	504.57028	135.31917	43.7768

Totals : 1152.59793 221.46940

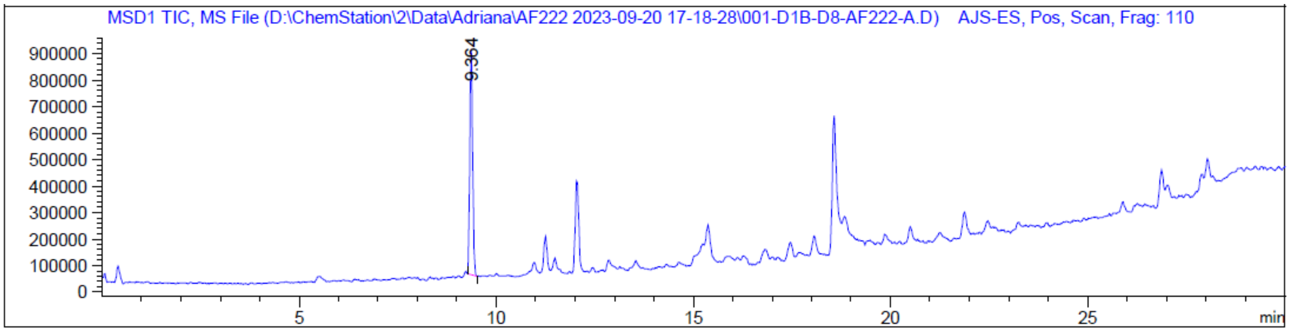

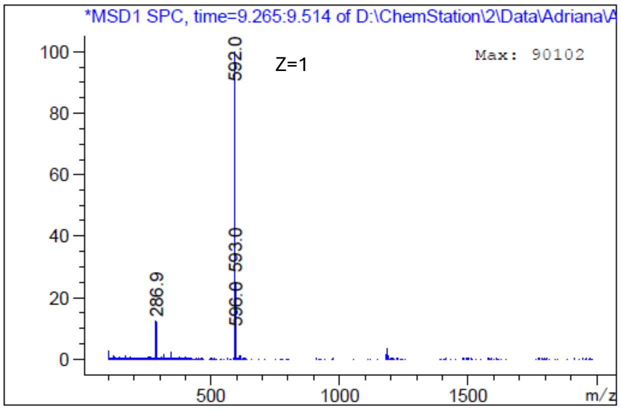

Peak #1 at 9.364 min (9.265 to 9.514 min)

General procedure D: bioconjugation of peptides at cysteine residues

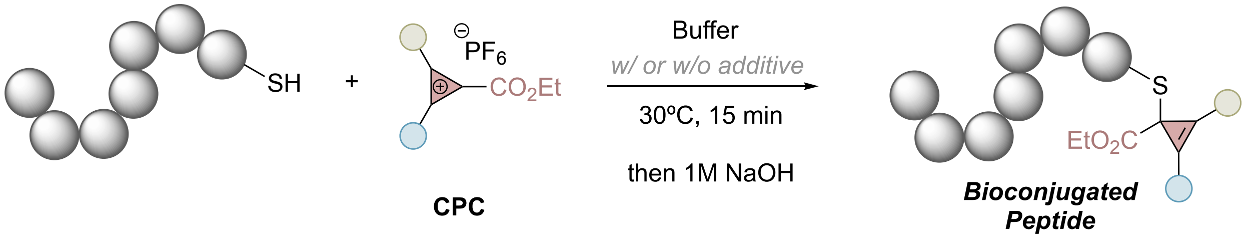

To a 1.5 mL Eppendorf tube were added the amount of PBS buffer 10 mM needed to reach a final volume of 100 μL, 10 μL of 10 or 50 mM stock solution of the peptide in PBS buffer and the corresponding **CPC** neat or dissolved in the proper amount of organic solvent as additive to improve the solubilization of the CPC compound in water. To those peptides that contained two cysteines connected in a S-S bridge, tris(2-carboxyethyl)phosphine hydrochloride (TCEP, 10 μL of a 11 or 20 mM stock solution in water, 1.1-2 equiv.) was added as the reducing agent and the mixture was stirred for 5 minutes at 37 ºC before the addition of the **CPC** reagent. The tube was

vortexed few seconds and stirred in an Eppendorf thermomixer at 37 ºC for the corresponding time. After that, the aqueous phase was washed twice with 200 µL of ethyl acetate or cold diethyl ether. The organic phase was discarded, and the mixture was filtered with PVDF Spheros Filters and analyzed by reverse phase UHPLC-MS.

Conversions were determined by integration of all peptide-containing peaks in the TIC spectra according to the following equation:

$$\% \text{ yield} = \frac{I_{pp}}{I_{sp} + I_{pp} + \sum I_{psp}} \qquad \textit{eq. (1)}$$

where I_{pp}, I_{sp} and I_{psp} are the average TIC integral areas for the peaks corresponding to the peptide product, the starting peptide and the peptide-based side products if present, respectively.

S-[1-(Ethoxycarbonyl)-2,3-dimethylcyclopropenyl] derivative of peptide Ac-GCFKT-NH_2 (10)

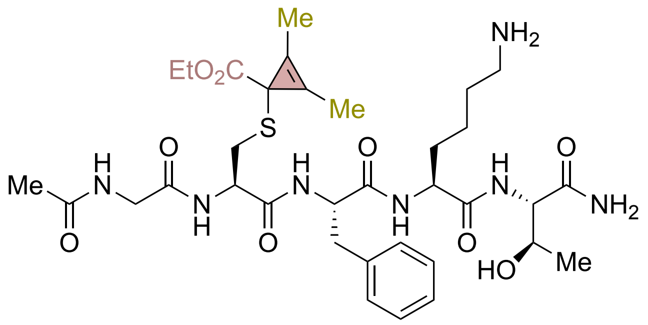

Obtained according to the ***general procedure D*** using commercial peptide Ac-GCFKT-NH_2 at 1 mM concentration and **CPC 2a** (2.8 mg, 100 equiv.) neat. In this case, the pH of the aqueous phase was adjusted to neutral with 1 M NaOH after 15 minutes of stirring and the mixture was washed with ethyl acetate (2 x 200 µL). The crude mixture was filtered and separated by reverse phase UHPLC-MS analysis on column Zorbax 300 SB-C8 over 10 minutes (*method 1* in ***section 2***). The title compound was detected in 97% analytical yield (R_t = 4.8 min), obtained according to the *equation (1)* above.

UHPLC-MS analysis (TIC and MS spectra) of starting peptide Ac-GCFKT-NH_2 using column Zorbax 300 SB-C8 over 10 minutes (*method 1* in ***section 2***, $R_{t\ sp}$ = 3.7 min):

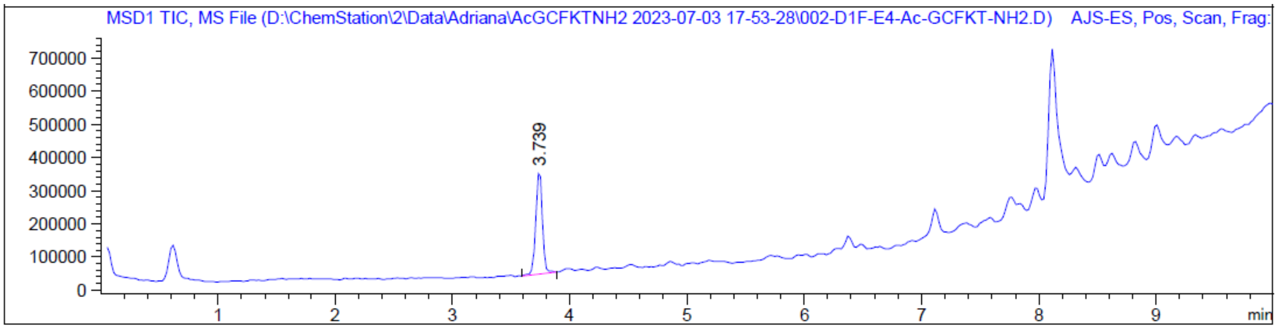

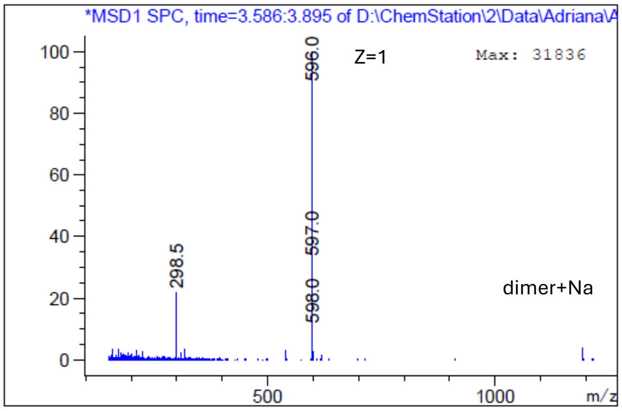

Peak #1 at 3.739 min (3.586 to 3.895 min)

UHPLC-MS analysis (TIC and MS spectra) of crude mixture using column Zorbax 300 SB-C8 over 10 minutes (*method 1* in ***section 2***, $R_{t\ product}$ = 4.8 min):

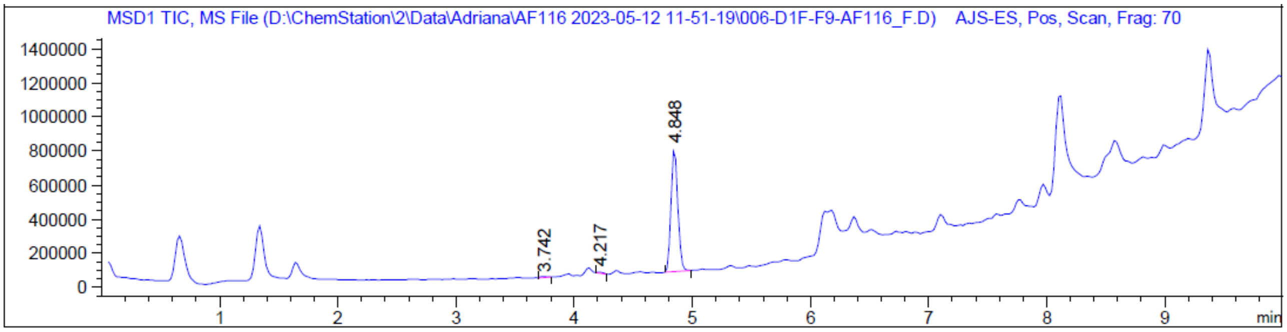

```
Signal 3: MSD1 TIC, MS File
```

Peak #	RetTime [min]	Type	Width [min]	Area	Height	Area %
1	3.742	BB	0.0546	1.67526e4	5185.13525	0.5675
2	4.217	BB	0.0530	2.74763e4	8894.33008	0.9308
3	4.848	BB	0.0638	2.90778e6	7.24184e5	98.5017
Totals :				2.95201e6	7.38263e5	

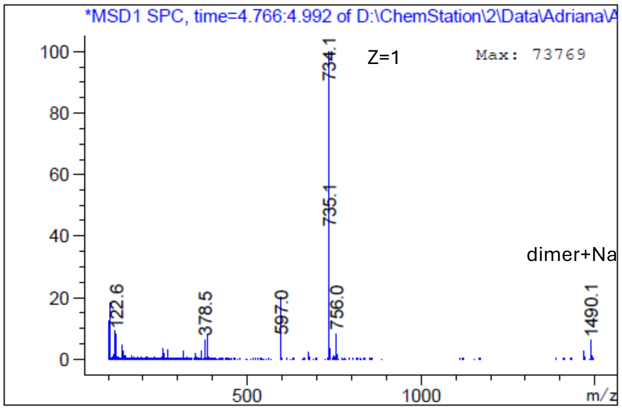

Peak #3 at 4.848 min (4.766 to 4.992 min)

S-[1-(Ethoxycarbonyl)-2,3-dimethylcyclopropenyl] derivative of peptide H-IAIAC-OH (11)

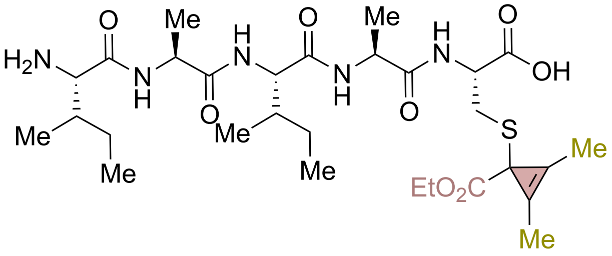

Obtained according to the ***general procedure D*** using the in house-prepared crude peptide H-IAIAC-OH at 5 mM concentration in 9:1 PBS buffer: DMF as organic cosolvent and **CPC 2a** (2.8 mg, 20 equiv.) neat. In this case, the pH of the aqueous phase was adjusted to neutral with 1 M NaOH after 15 minutes of stirring and the mixture was washed with ethyl acetate (2 x 200 µL). The crude mixture was filtered and separated by reverse phase UHPLC-MS analysis on column

Poroshell 120 EC-C18 over 10 minutes (*method 4* in ***section 2***). The title compound was detected in 78% analytical yield (R_t = 3.8 min), obtained according to the *equation (1)* above.

UHPLC-MS analysis (TIC and MS spectra) of starting peptide H-IAIAC-OH using column Poroshell 120 EC-C18 over 10 minutes (*method 4* in ***section 2***, $R_{t\ sp}$ = 3.0 min):

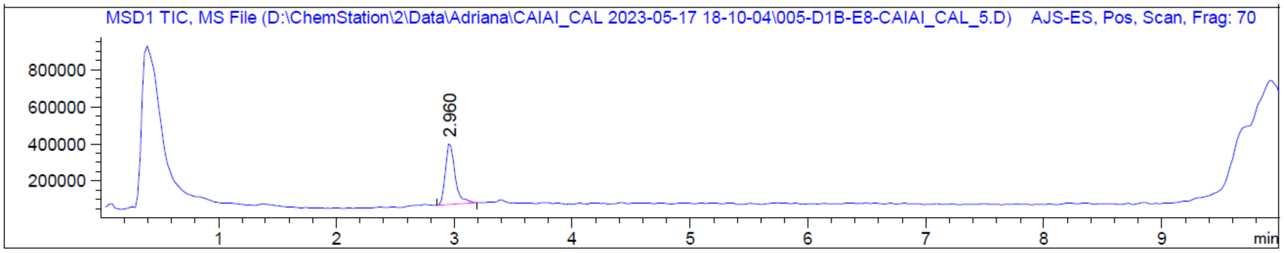

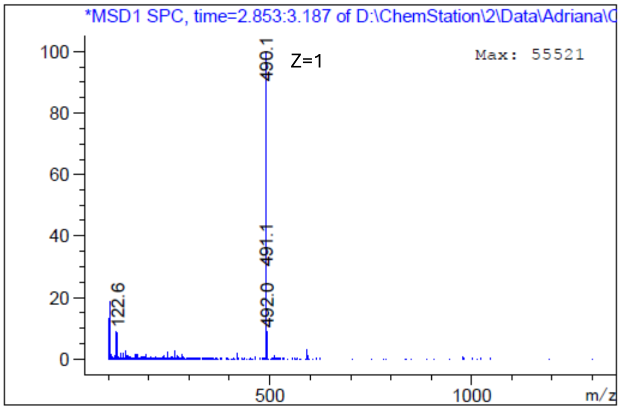

Peak #1 at 2.960 min (2.853 to 3.192 min)

UHPLC-MS analysis (TIC and MS spectra) of crude mixture using column Poroshell 120 EC-C18 over 10 minutes (*method 4* in ***section 2***, $R_{t\ product}$ = 3.8 min):

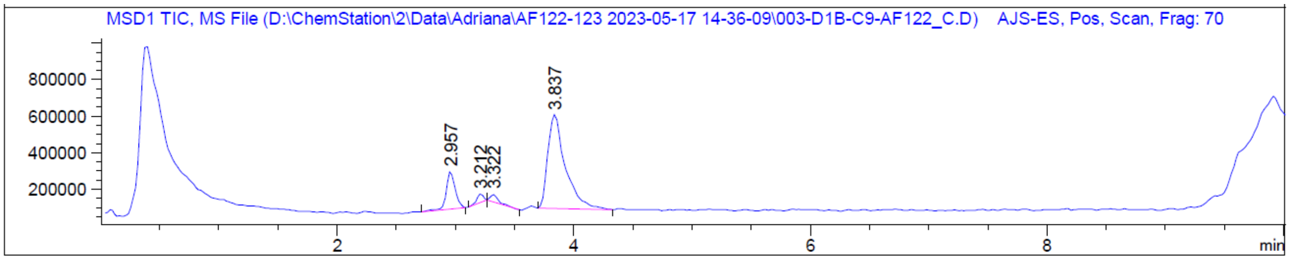

Signal 2: MSD1 TIC, MS File

Peak #	RetTime [min]	Type	Width [min]	Area	Height	Area %
1	2.957	BB	0.0792	1.09225e6	2.07253e5	16.1369
2	3.212	BB	0.0596	1.68615e5	4.70089e4	2.4911
3	3.322	BB	0.0726	1.97084e5	4.18509e4	2.9117
4	3.837	BB	0.1557	5.31068e6	5.15239e5	78.4602
Totals :				6.76863e6	8.11352e5	

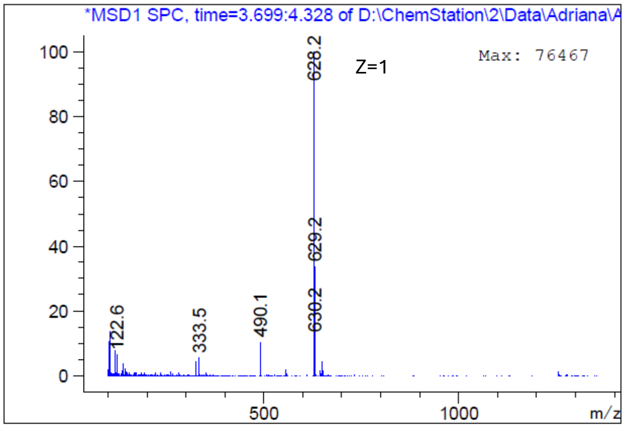

Peak #4 at 3.837 min (3.699 to 4.328 min)

S-[1-(Ethoxycarbonyl)-2,3-dimethylcyclopropenyl] derivative of GTP-binding protein fragment, G alpha (12)

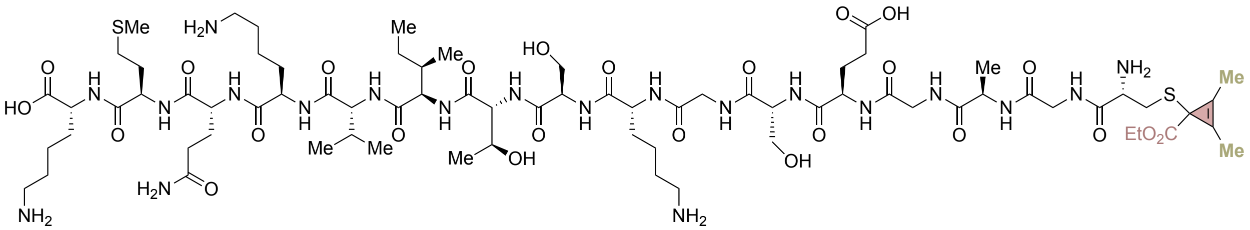

Obtained according to the ***general procedure D*** using the commercially available GTP-binding protein fragment, G alpha (> 74% HPLC purity) at 5 mM concentration and **CPC 2a** (2.8 mg, 20 equiv.) neat. After 15 minutes of stirring the mixture was washed with ethyl acetate (2 x 200 µL). The crude mixture was filtered and separated by reverse phase UHPLC-MS analysis on column

Zorbax 300 SB-C8 over 30 minutes (*method 2* in ***section 2***). The title compound **12** was detected in 22% analytical yield (R_t = 5.7 min), obtained according to the *equation (1)* above.

UHPLC-MS analysis (TIC and MS spectra) of crude mixture using column Zorbax 300 SB-C8 over 30 minutes (*method 2* in ***section 2***, $R_{t\ sp}$ = 2.6 min, $R_{t\ product}$ = 5.7 min):

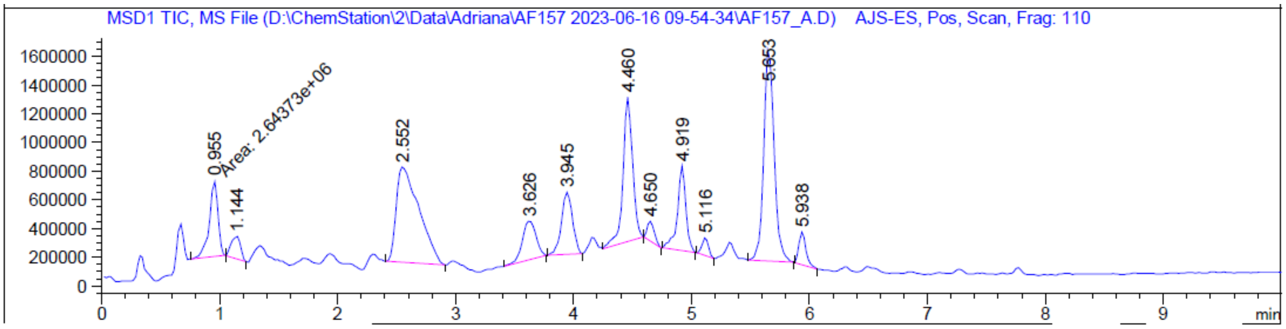

Signal 4: MSD1 TIC, MS File

Peak #	RetTime [min]	Type	Width [min]	Area	Height	Area %
1	0.955	MM	0.0826	2.64373e6	5.33575e5	7.1090
2	1.144	BB	0.0929	8.64968e5	1.57287e5	2.3259
3	2.552	BB	0.1854	8.92982e6	6.65021e5	24.0123
4	3.626	BB	0.1297	2.20908e6	2.70470e5	5.9402
5	3.945	BB	0.0991	2.82522e6	4.33191e5	7.5970
6	4.460	BB	0.0829	5.66628e6	1.00652e6	15.2366
7	4.650	BB	0.0659	5.78771e5	1.38586e5	1.5563
8	4.919	BB	0.0771	3.03415e6	5.91302e5	8.1588
9	5.116	BB	0.0687	4.76005e5	1.21555e5	1.2800
10	5.653	BB	0.0935	9.02959e6	1.49348e6	24.2806
11	5.938	BB	0.0650	9.30930e5	2.27177e5	2.5033
Totals :				3.71886e7	5.63816e6	

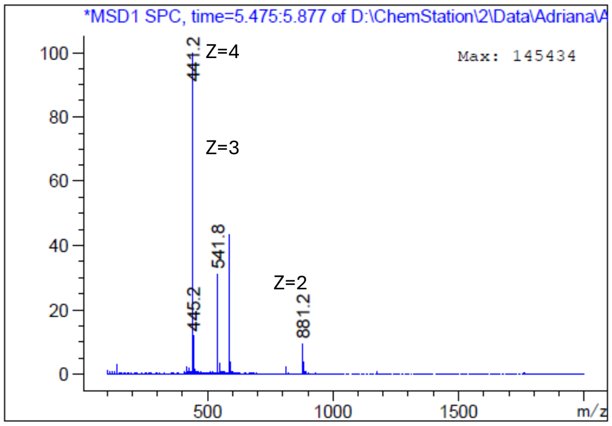

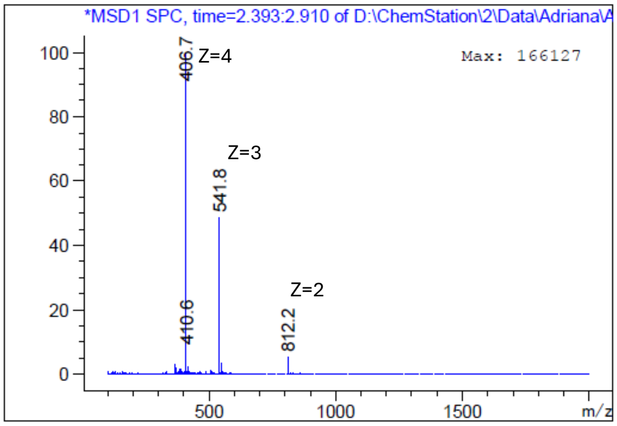

Peak #10 at 5.653 min (5.478 to 5.869 min)   Peak #3 at 2.552 min (2.402 to 2.910 min)

S-[1-(Ethoxycarbonyl)-2,3-dimethylcyclopropenyl] derivative of reduced oxytocin (13)

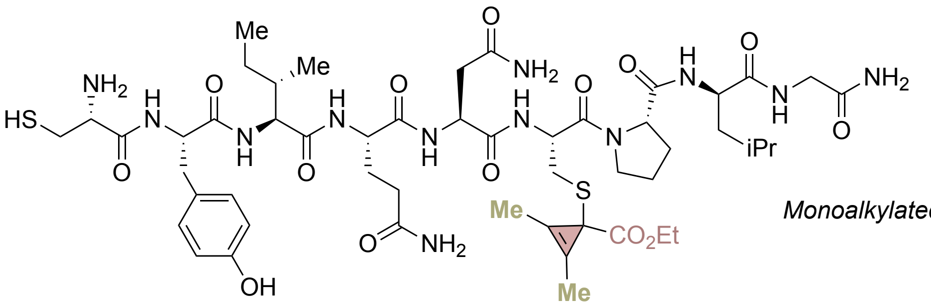

Monoalkylated **13**

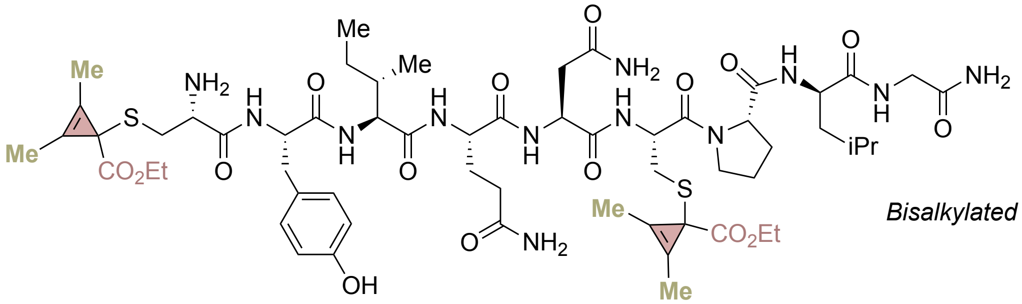

Bisalkylated **13***

Product **13** and **13*** were obtained from the same reaction mixture according to the ***general procedure D*** using commercially available oxytocin acetate hydrate at 1 mM concentration, 1.2 equiv. of TCEP from a 12 mM stock solution in water and **CPC 2a** (2.8 mg, 100 equiv.) neat. The mixture was washed with cold diethyl ether (2 x 200 μL) after stirring at 37 ºC for 15 minutes, then filtered and separated by reverse phase UHPLC-MS analysis on column Zorbax 300 SB-C8

over 30 minutes (*method 2* in ***section 2***). The title compound **13** was detected in 58% analytical yield (R_t = 9.3 min) and 42% analytical yield (R_t = 11.3 min), respectively, according to the *equation (1)* above.

UHPLC-MS analysis (TIC and MS spectra) of reduced oxytocin using column Zorbax 300 SB-C8 over 30 minutes (*method 2* in ***section 2***, $R_{t\ sp}$ = 6.7 min):

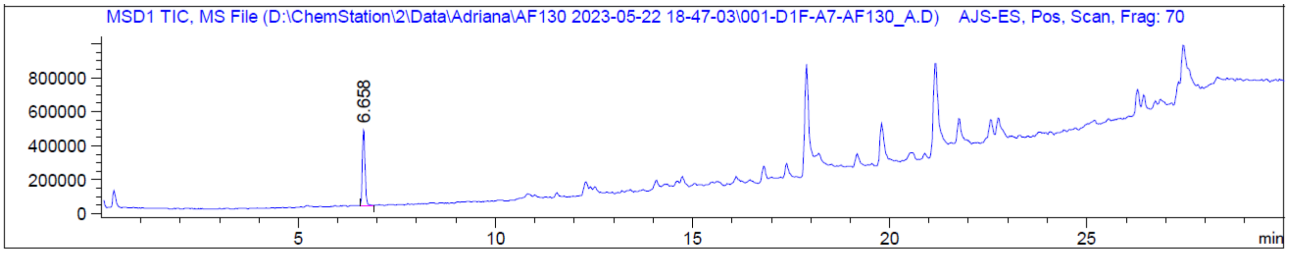

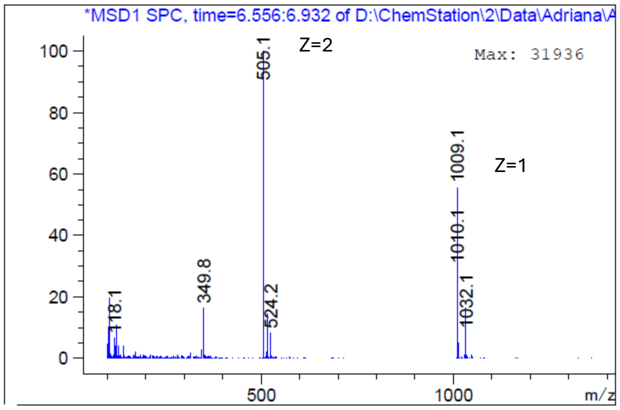

Peak #1 at 6.658 min (6.556 to 6.929 min)

UHPLC-MS analysis (TIC and MS spectra) of crude mixture using column Zorbax 300 SB-C8 over 30 minutes (*method 2* in ***section 2***, $R_{t\ monoalkylated}$ = 9.3 min, $R_{t\ bisalkyated}$ = 11.3 min):

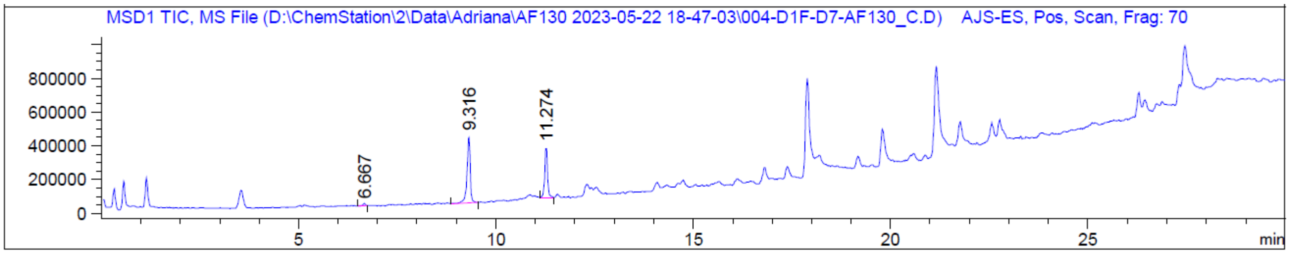

Signal 2: MSD1 TIC, MS File

Peak #	RetTime [min]	Type	Width [min]	Area	Height	Area %
1	6.667	BB	0.0766	7.25638e4	1.42032e4	1.8687
2	9.316	BB	0.0903	2.31165e6	3.98166e5	59.5301
3	11.274	BB	0.0808	1.49895e6	3.00437e5	38.6012

Totals : 3.88316e6 7.12806e5

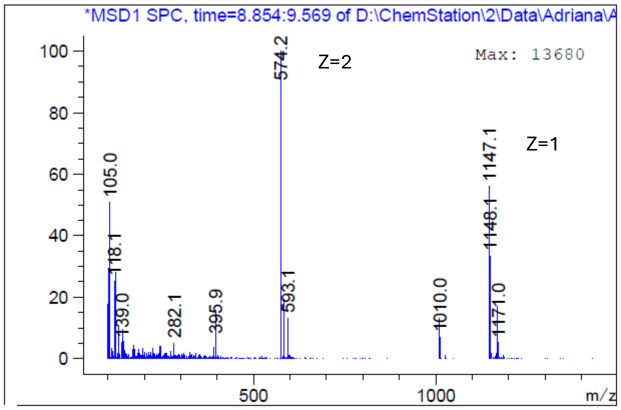

Peak #2 at 9.316 min (8.854 to 9.569 min)

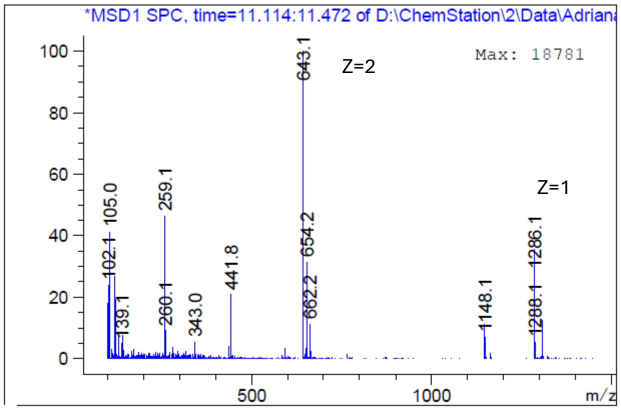

Peak #3 at 11.274 min (11.114 to 11.472 min)

S-[1-(Ethoxycarbonyl)-2,3-dimethylcyclopropenyl] derivative of reduced salmon calcitonin (14)

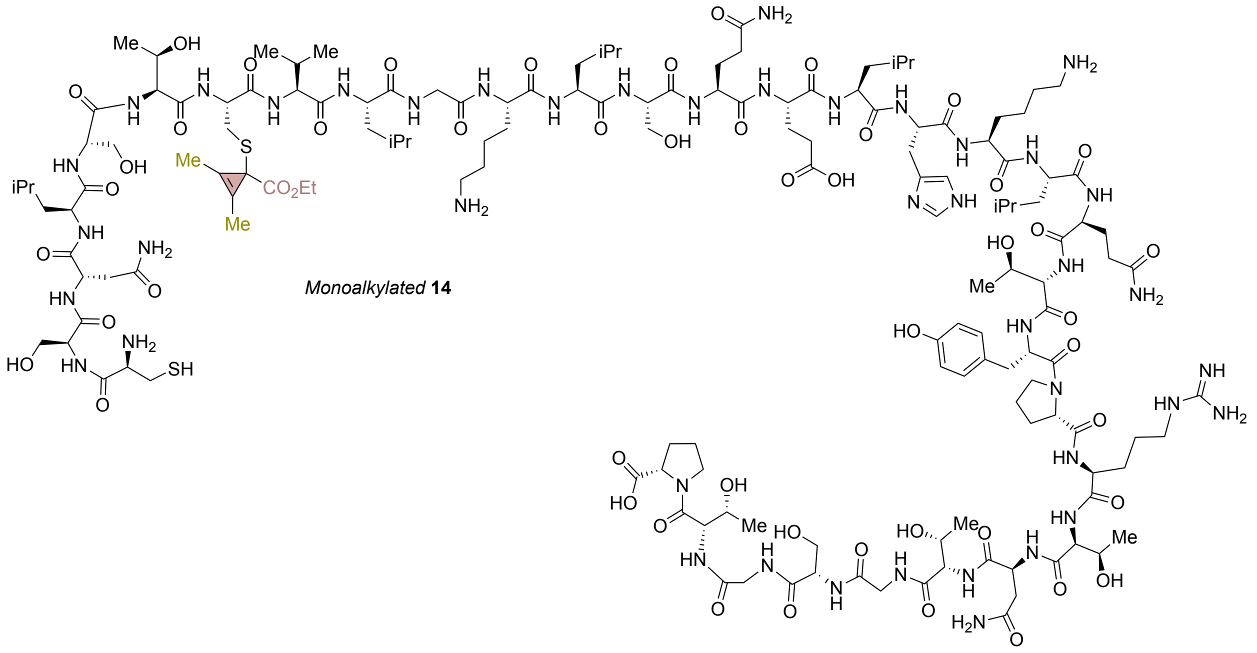

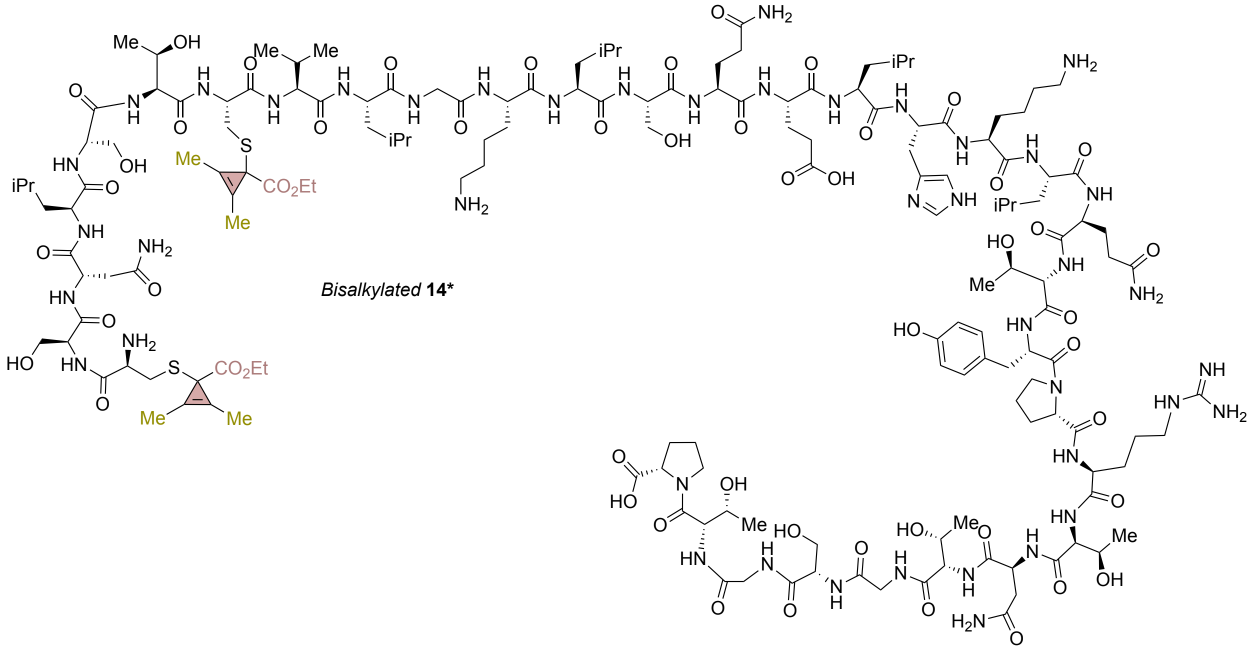

Products **14** and **14*** (mono- and bis-alkylated) were obtained from the same reaction mixture according to the ***general procedure D*** using commercially available Salmon Calcitonin at 1 mM concentration, 2 equiv. of TCEP from a 20 mM stock solution in water and **CPC 2a** (2.8 mg, 100 equiv.) neat. The mixture was washed with cold diethyl ether (2 x 200 μL) after stirring at 37 ºC

for 15 minutes, then filtered and separated by reverse phase UHPLC-MS analysis on column Zorbax 300 SB-C8 over 30 minutes (*method 2* in ***section 2***). The title compounds **14** were detected in 48% conversion of the **mono-alkylated** products (R_t = 10.5 and 10.7 min) and 44% analytical yield of **bis-alkylated** (R_t = 11.2 min), obtained according to the *equation (1)* above.

UHPLC-MS analysis (TIC and MS spectra) of reduced salmon calcitonin using column Zorbax 300 SB-C8 over 30 minutes (*method 2* in ***section 2***, $R_{t\ sp}$ = 9.8 min):

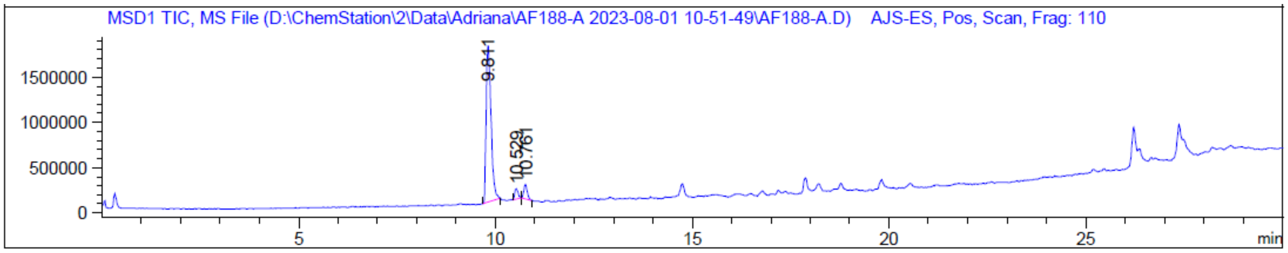

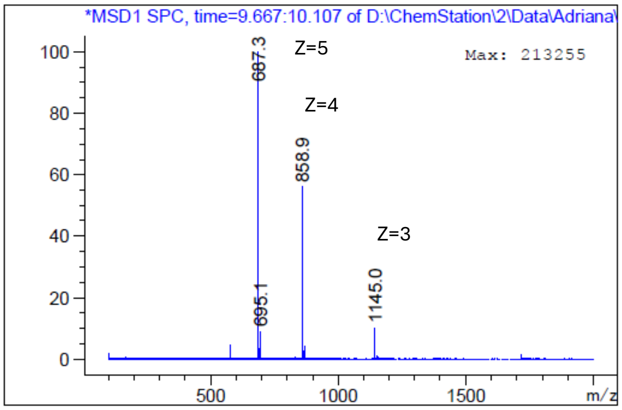

Peak #1 at 9.811 min (9.667 to 10.107 min)

UHPLC-MS analysis (TIC and MS spectra) of crude mixture using column Zorbax 300 SB-C8 over 30 minutes (*method 2* in ***section 2***, $R_{t\ monoalkylated\ product\ external}$ = 10.5 min, $R_{t\ monoalkylated\ product\ internal}$ = 10.7 min, $R_{t\ bisalkylated\ product}$ = 11.2 min):

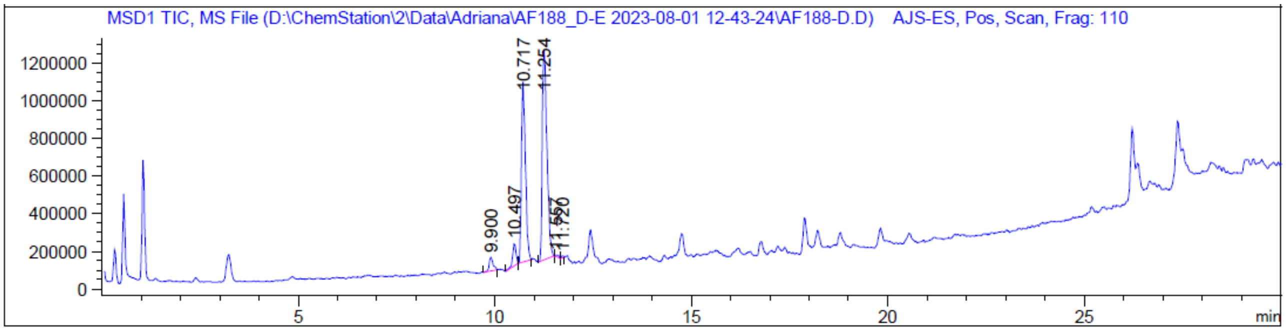

Signal 3: MSD1 TIC, MS File

Peak #	RetTime [min]	Type	Width [min]	Area	Height	Area %
1	9.900	BB	0.1055	5.06152e5	7.15591e4	2.9391
2	10.497	BB	0.0982	7.10861e5	1.19423e5	4.1278
3	10.717	BB	0.1109	6.77064e6	9.61196e5	39.3154
4	11.254	BB	0.1239	9.13490e6	1.11946e6	53.0440
5	11.557	BB	0.1072	6.09891e4	7435.88232	0.3541
6	11.720	BB	0.0554	3.78157e4	1.15703e4	0.2196
Totals :				1.72214e7	2.29064e6	

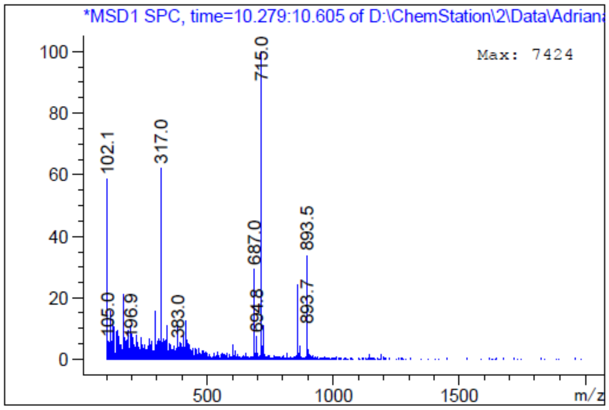

Peak #2 at 10.497 min (10.279 to 10.602 min)

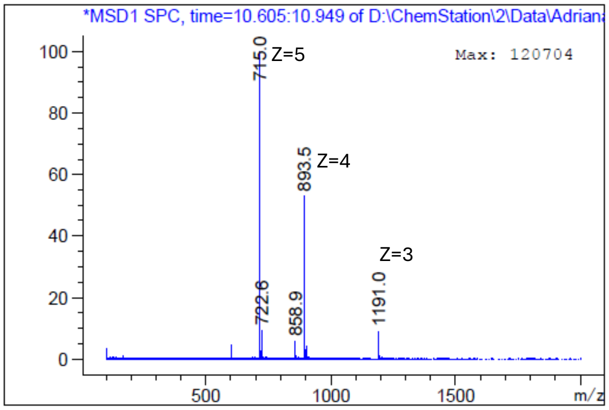

Peak #3 at 10.717 min (10.605 to 10.942 min)

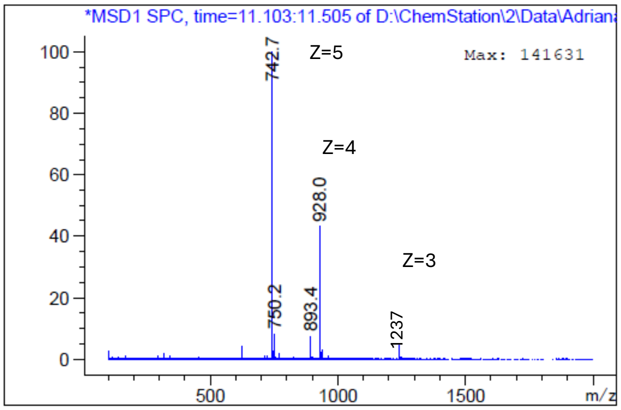

Peak #4 at 11.254 min (11.103 to 11.514 min)

S-[1-(ethoxycarbonyl)-2-cyclopropyl-3-tolyl] derivative of Coenzime A (15)

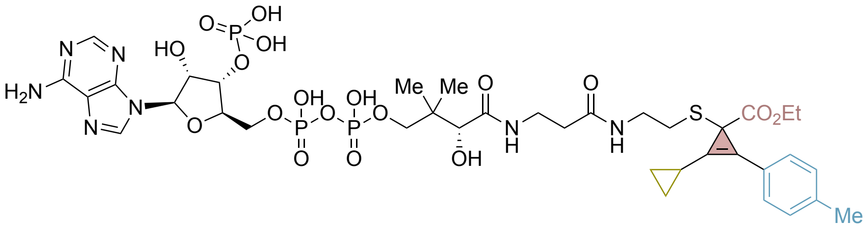

Obtained according to the ***general procedure D*** using the commercially available Coenzime A Hydrate at 10 mM concentration and **CPC 2f** (4 mg, 10 equiv.) neat. After 15 minutes of stirring the mixture was washed with ethyl acetate (2 x 200 μL). The crude mixture was filtered and separated by reverse phase UHPLC-MS analysis on column Zorbax 300 SB-C8 over 10 minutes (*method 1* in ***section 2***). The title compound **15** was detected in 55% conversion (R_t = 5.17 min), obtained according to the *equation (1)* above.

UHPLC-MS analysis (TIC and MS spectra) of CoA using column Zorbax 300 SB-C8 over 30 minutes (*method 2* in ***section 2***, $R_{t\ sm}$ = 9.8 min):

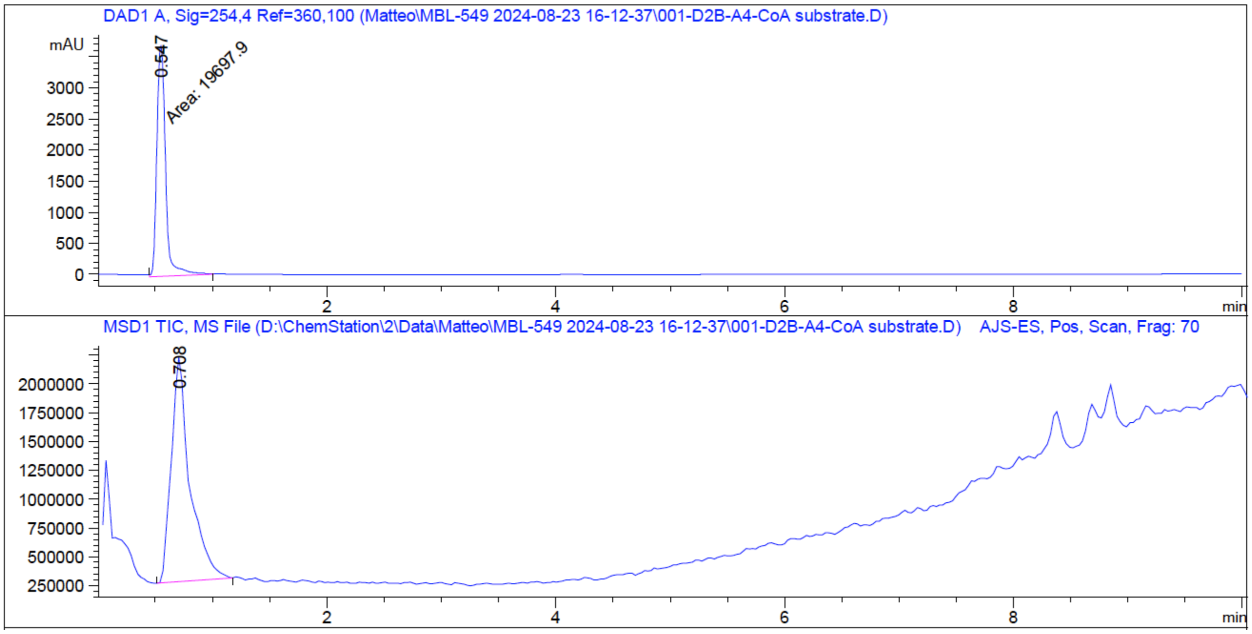

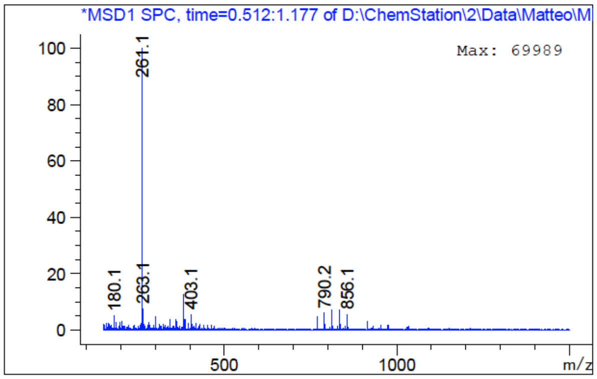

Peak #1 at 0.708 min (0.512 to 1.174 min)

UHPLC-MS analysis (TIC and MS spectra) of crude mixture using column Zorbax 300 SB-C8 over 10 minutes (*method 1* in ***section 2***, $R_{t\ sm}$ = 0.7 min, $R_{t\ product}$ = 5.2 min):

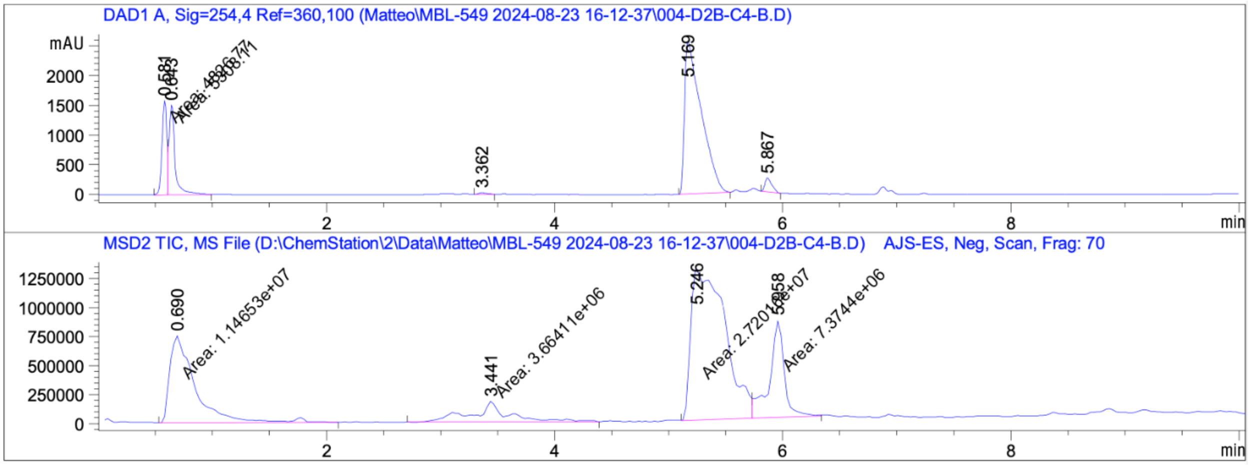

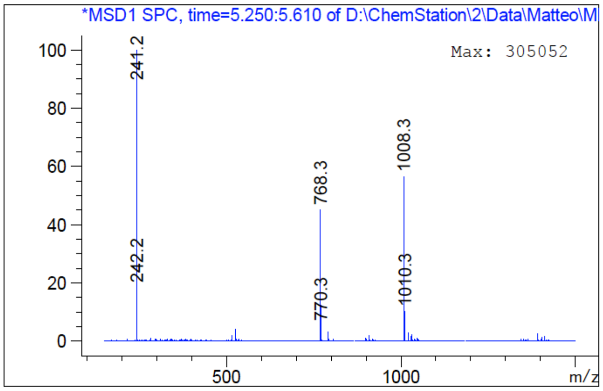

Signal 2: MSD2 TIC, MS File

Peak #	RetTime [min]	Type	Width [min]	Area	Height	Area %
1	0.690	MM	0.2549	1.14653e7	7.49589e5	23.0665
2	3.441	MM	0.3423	3.66411e6	1.78393e5	7.3716
3	5.246	MF	0.3413	2.72016e7	1.32834e6	54.7256
4	5.958	FM	0.1461	7.37440e6	8.41371e5	14.8362

Totals : 4.97054e7 3.09769e6

Peak #1 at 5.369 min (5.250 to 5.613 min)

7. Cyclopropenylation of Proteins

Proteinogenic bioconjugates were obtanied following the ***general procedure D*** described before. Cystein containing proteins were purchased from CRG (Center for Genomic Regulation, Barcelona). After reaction the crude was purified by centrifugation using Amicon® Ultra centrifugal filter (3kDa). MALDI-TOF-MS analysis of the filtered sample was performed on a Bruker AutoFlex mass spectrometer using sinapinic acid (SA) in TA (ACN:TFA 0.1% in H_2O) 1:2. The sample was previously desalted using Pierce™ C18 Spin Columns with 70% ACN in H_2O + 0.1% HCOOH as the elution buffer.

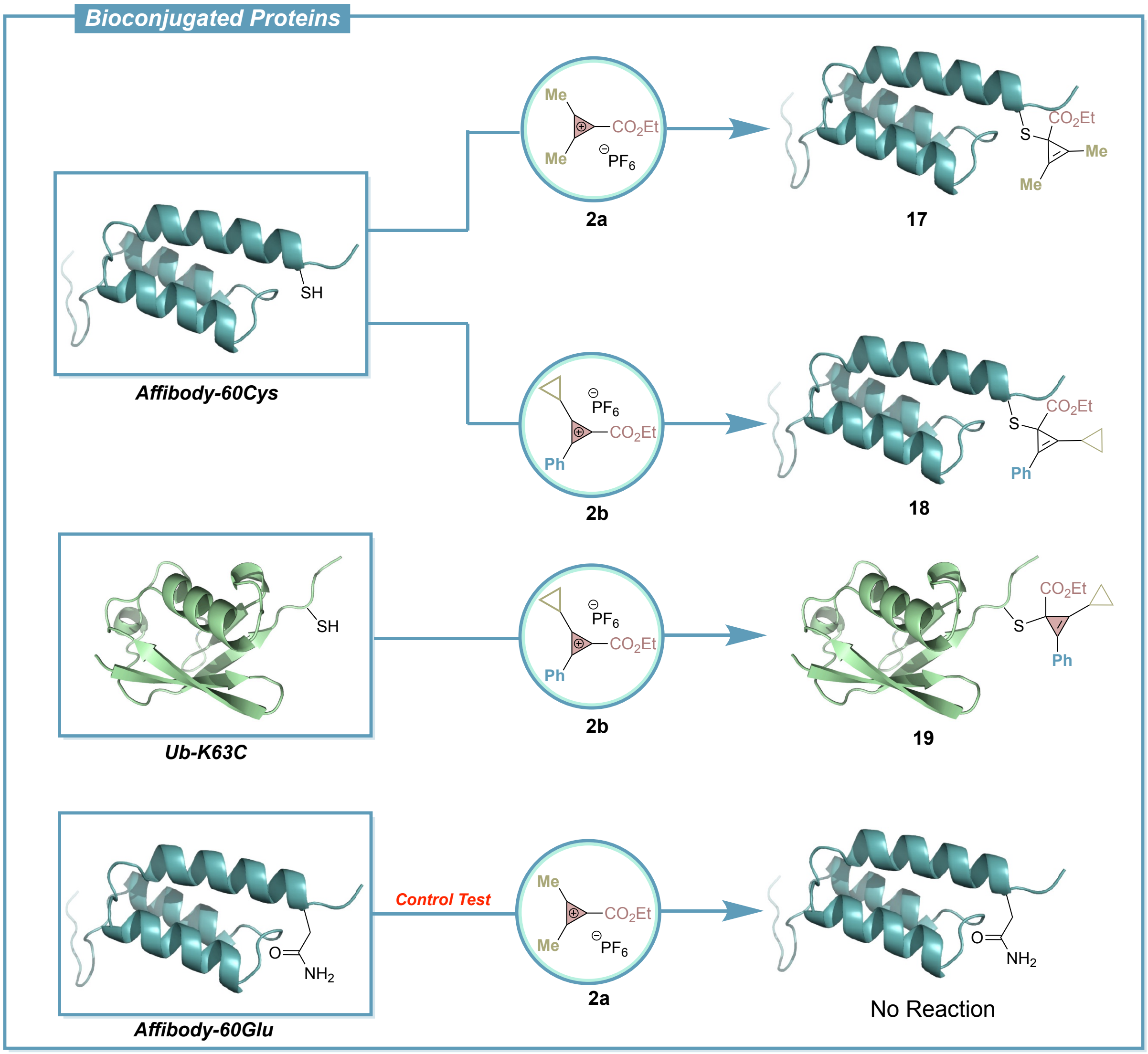

Figure S4: Protein tested in this work

Affibody-60Cys

Sequence:
GGGGGVDNKFNKEQQNAFYEILHLPNLNEEQRNAFIQSLKDDPSQSANLLAEAKKLN
DA**C**APK
Lenght: 63 aa
Calculated Mass: 6900.6 Da

Ub-K63C [3]

Sequence:
SAQIFVKTLTGKTITLEVEPSDTIENVKAKIQDKEGIPPDQQRLIFAGKQLEDGRTLSD
YNIQ**C**ESTLHLVLRLRGG
Lenght: 77 aa
Calculated Mass: 8566.8 Da

Affibody-60Glu

Sequence:
GGGGGVDNKFNKEQQNAFYEILHLPNLNEEQRNAFIQSLKDDPSQSANLLAEAKKLN
DA**Q**APK
Lenght: 63 aa
Calculated Mass: 6925.6 Da

Procedure for the selective cyclopropenylation of Affibody-60Cys: **17**

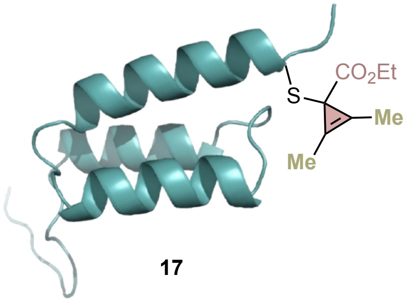

17

To a 1.5 mL Eppendorf tube were added 100 μL of the 0.720 mg/mL protein solution in PBS buffer (104 μM) and **CPC 2a** neat (2.8 mg, 960 equiv.). The tube was vortexed few seconds and the mixture was stirred for 15 minutes at 37 ºC in an Eppendorf thermomixer. The aqueous phase was filtered and analyzed by reverse phase UHPLC-MS using Agilent AdvanceBio RP-mAb SB-C8 column over 30 minutes (*method 2* in ***section 2***) to afford the modified protein **17** in 90% conversion based on TIC integration as described in ***general procedure D***.

UHPLC-MS analysis (TIC and MS spectra + deconvoluted spectrum) of Affibody-60Cys (Starting Material) using Agilent AdvanceBio RP-mAb SB-C8 column over 30 minutes (*method 2* in ***section 2***, R_t = 9.4 min):

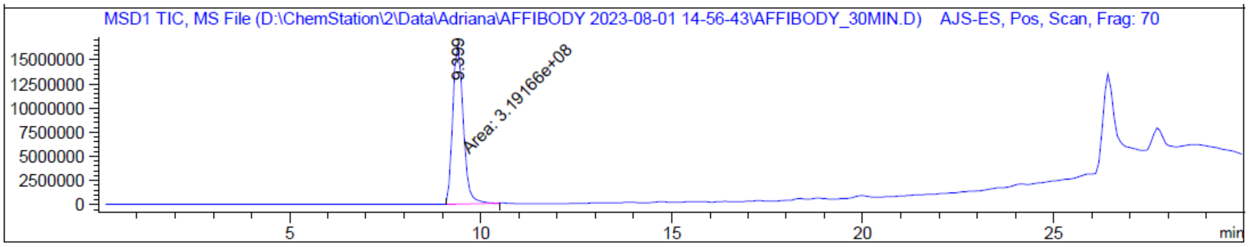

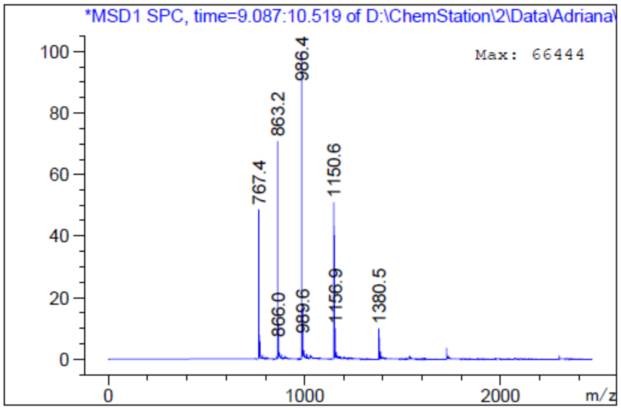

Peak #1 at 9.399 min (9.082 to 10.493 min)

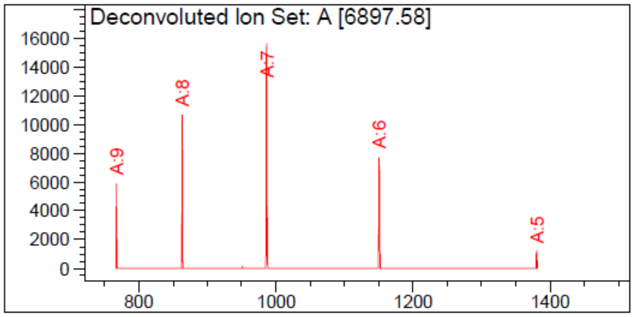

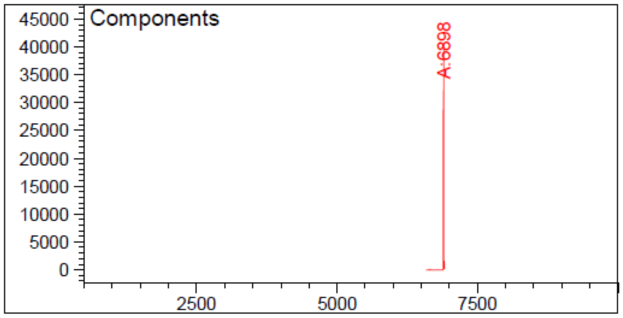

Component	Molecular Weight	Absolute Abundance	Relative Abundance
A	6897.58	40448	100.00

UHPLC-MS analysis (TIC and MS spectra + deconvoluted spectrum) of crude mixture using column Zorbax 300 SB-C8 over 30 minutes (*method 2* in ***section 2***, $R_{t\ product}$= 10.0 min):

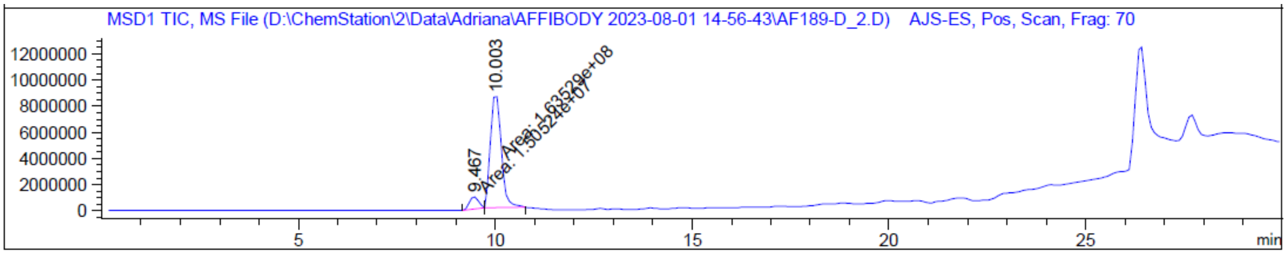

Signal 2: MSD1 TIC, MS File

Peak #	RetTime [min]	Type	Width [min]	Area	Height	Area %
1	9.467	MM	0.2559	1.50524e7	9.80402e5	8.4289
2	10.003	MM	0.3049	1.63529e8	8.93865e6	91.5711
Totals :				1.78582e8	9.91905e6	

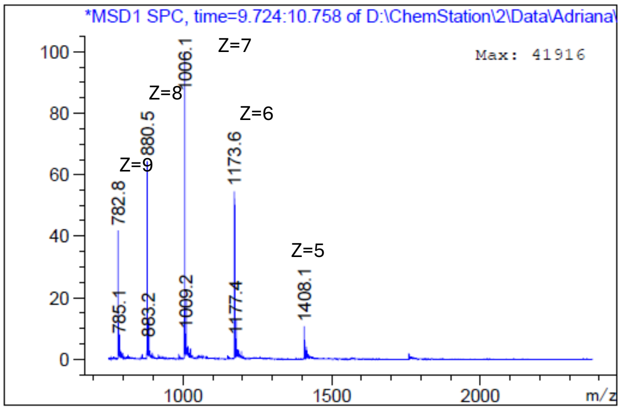

Peak #2 at 10.003 min (9.723 to 10.753 min)

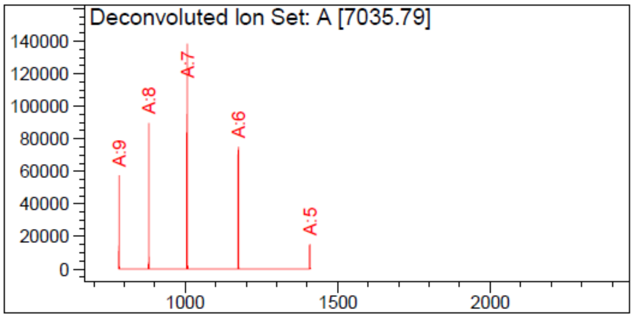

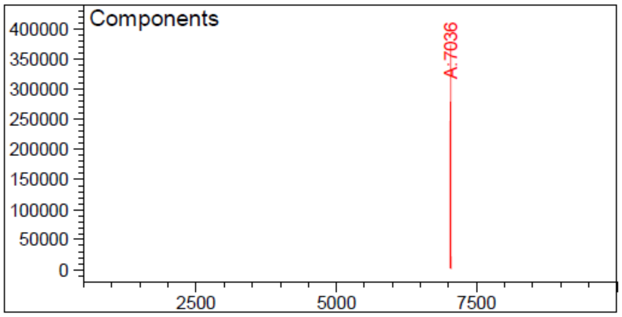

Component	Molecular Weight	Absolute Abundance	Relative Abundance
A	7035.79	374011	100.00

MALDI-TOF-MS analysis of **17**

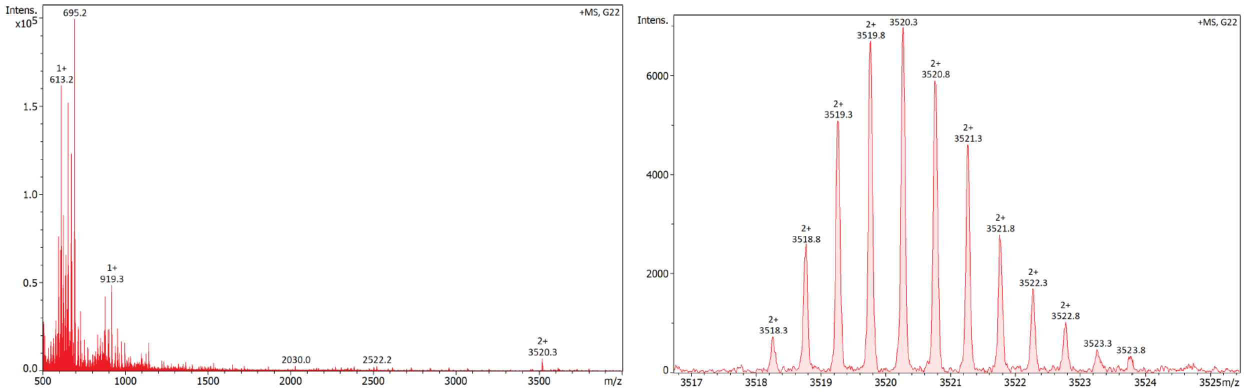

Procedure for the selective cyclopropenylation of Affibody-60Cys: **18**

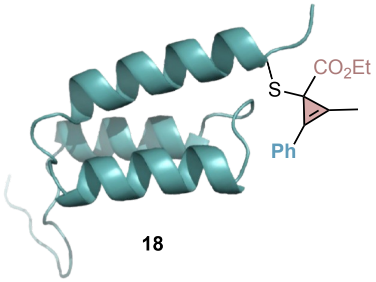

18

To a 1.5 mL Eppendorf tube were added 100 µL of the 0.720 mg/mL protein solution in PBS buffer (104 µM) followed by 50 µL of MeCN (UHPLC Grade) and **CPC 2c** neat (8 mg, 2000 equiv.). The tube was vortexed few seconds and the mixture was stirred for 15 minutes at 37 ºC in an Eppendorf thermomixer. The aqueous phase was filtered and analyzed by reverse phase UHPLC-MS using Agilent AdvanceBio RP-mAb SB-C8 column over 30 minutes (*method 2* in ***section 2***) to afford the modified protein **18** in 78% conversion based on TIC integration as described in ***general procedure D***.

UHPLC-MS analysis (TIC and MS spectra + deconvoluted spectrum) of Affibody-60Cys (Starting Material) using Agilent AdvanceBio RP-mAb SB-C8 column over 30 minutes (*method 2* in ***section 2***, R_t = 9.4 min):

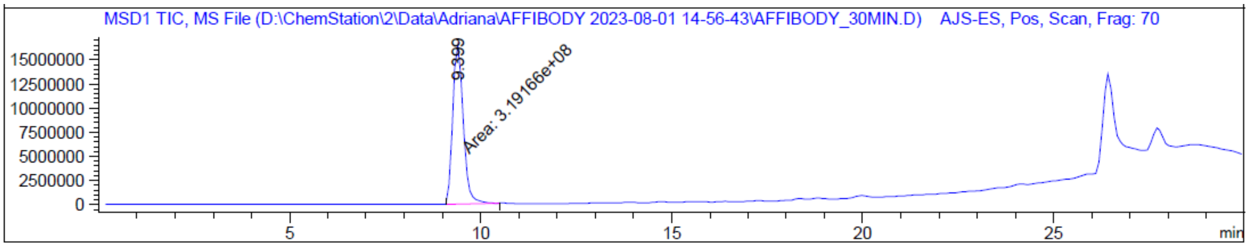

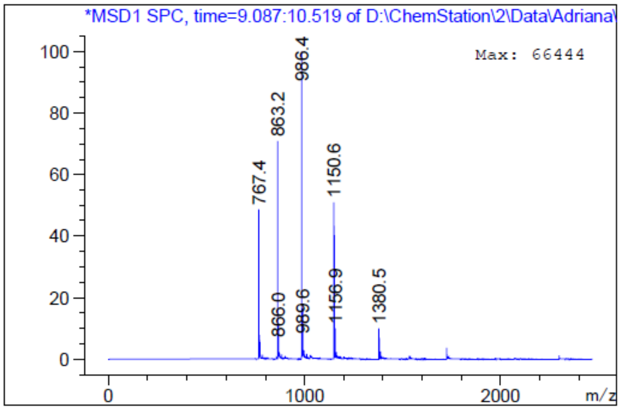

Peak #1 at 9.399 min (9.082 to 10.493 min)

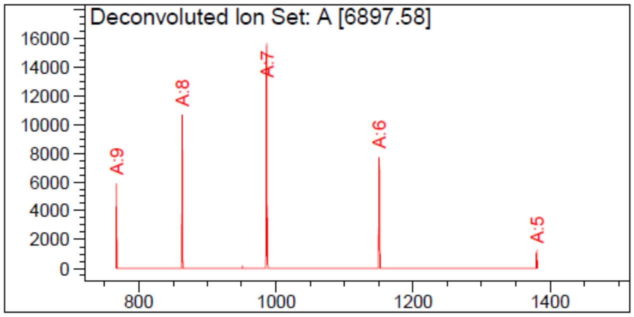

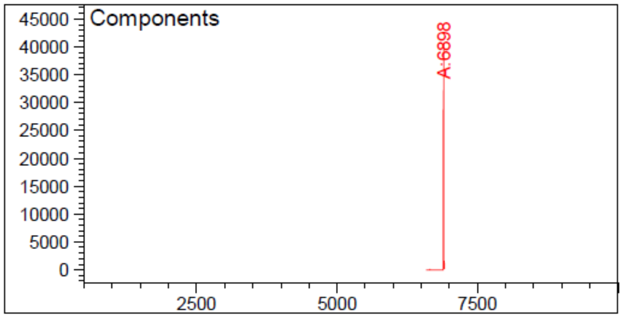

Component	Molecular Weight	Absolute Abundance	Relative Abundance
A	6897.58	40448	100.00

UHPLC-MS analysis (TIC and MS spectra + deconvoluted spectrum) of crude mixture using Agilent AdvanceBio RP-mAb SB-C8 column over 30 minutes (*method 2* in ***section 2***, R_t = 11.1 min):

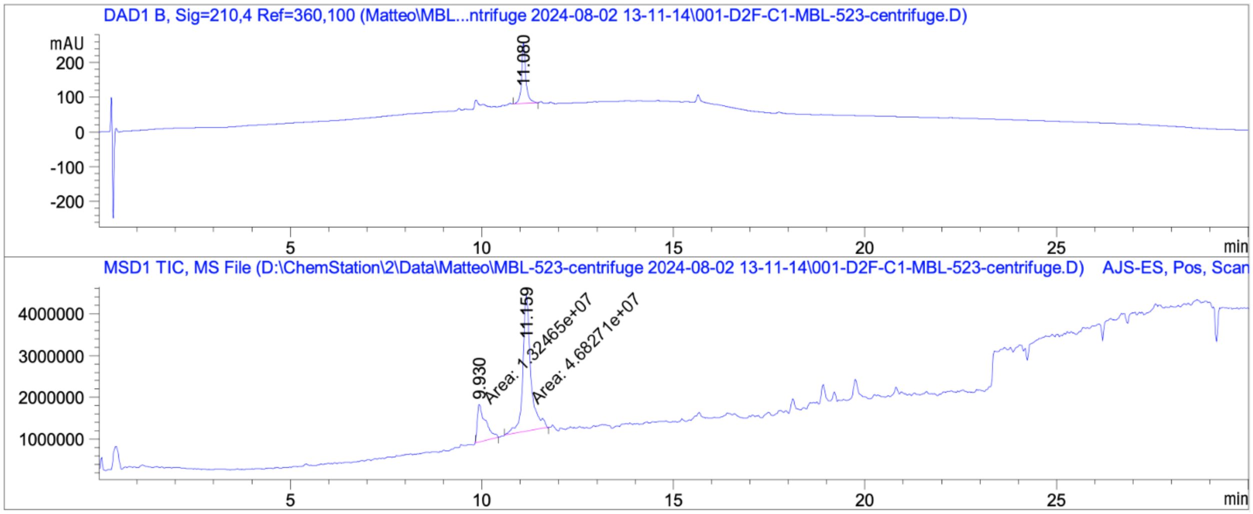

Signal 2: MSD1 TIC, MS File

Peak #	RetTime [min]	Type	Width [min]	Area	Height	Area %
1	9.930	MM	0.2436	1.32465e7	9.06143e5	22.0505
2	11.159	MM	0.2403	4.68271e7	3.24728e6	77.9495

Deconvolution of Spectrum # 1 @ 11.076 min

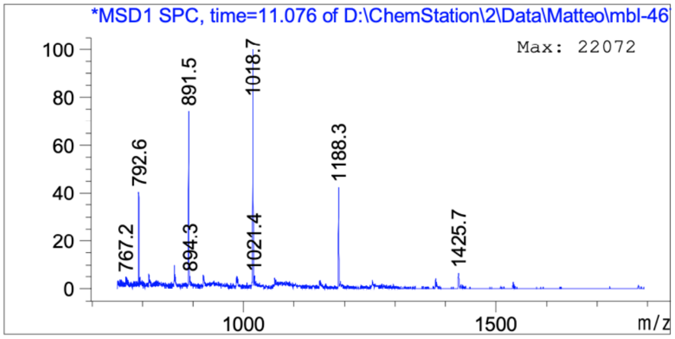

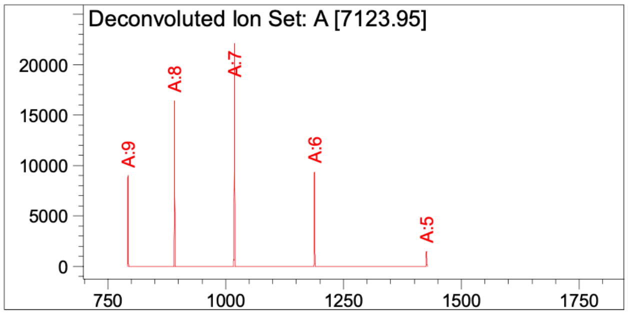

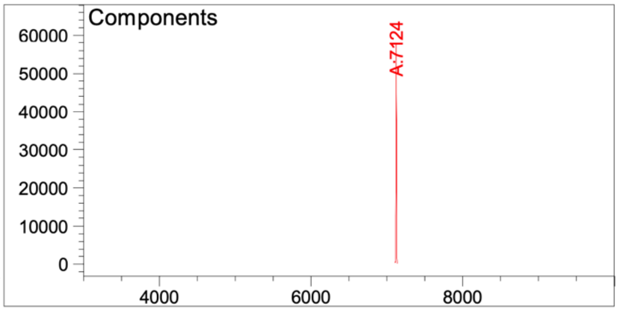

MALDI-TOF-MS analysis of **18**

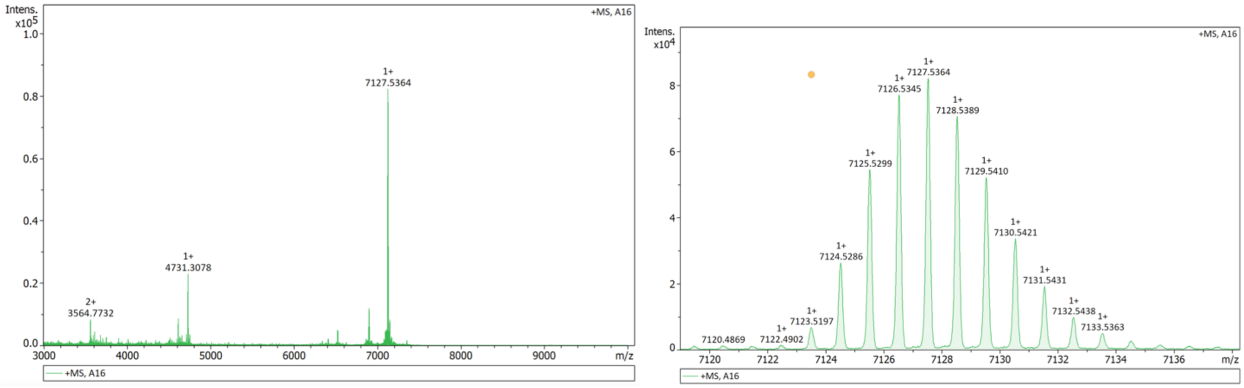

<u>*Procedure for the selective cyclopropenylation of Ub-K63C:*</u> **19**

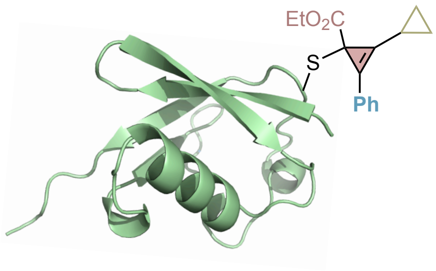

19

To a 1.5 mL Eppendorf tube were added 100 μL of the 0.91 mg/mL protein solution in PBS buffer (100 μM) and **CPC 2c** neat (2.0 mg, 500 equiv.). The tube was vortexed few seconds and the mixture was stirred for 15 minutes at 37 ºC in an Eppendorf thermomixer. The aqueous phase was filtered and analyzed by reverse phase UHPLC-MS using using Agilent AdvanceBio RP-mAb SB-C8 column over 10 minutes (*method 1* in ***section 2***) to afford the modified protein **19** in 84% conversion as an average of three runs based on TIC integration as described in ***general procedure D***.

UHPLC-MS analysis (TIC and MS spectra + deconvoluted spectrum) of Ub-K63C (Starting Material) using Agilent AdvanceBio RP-mAb SB-C8 column over 10 minutes (*method 1* in ***section 2***, R_t = 5.4 min):

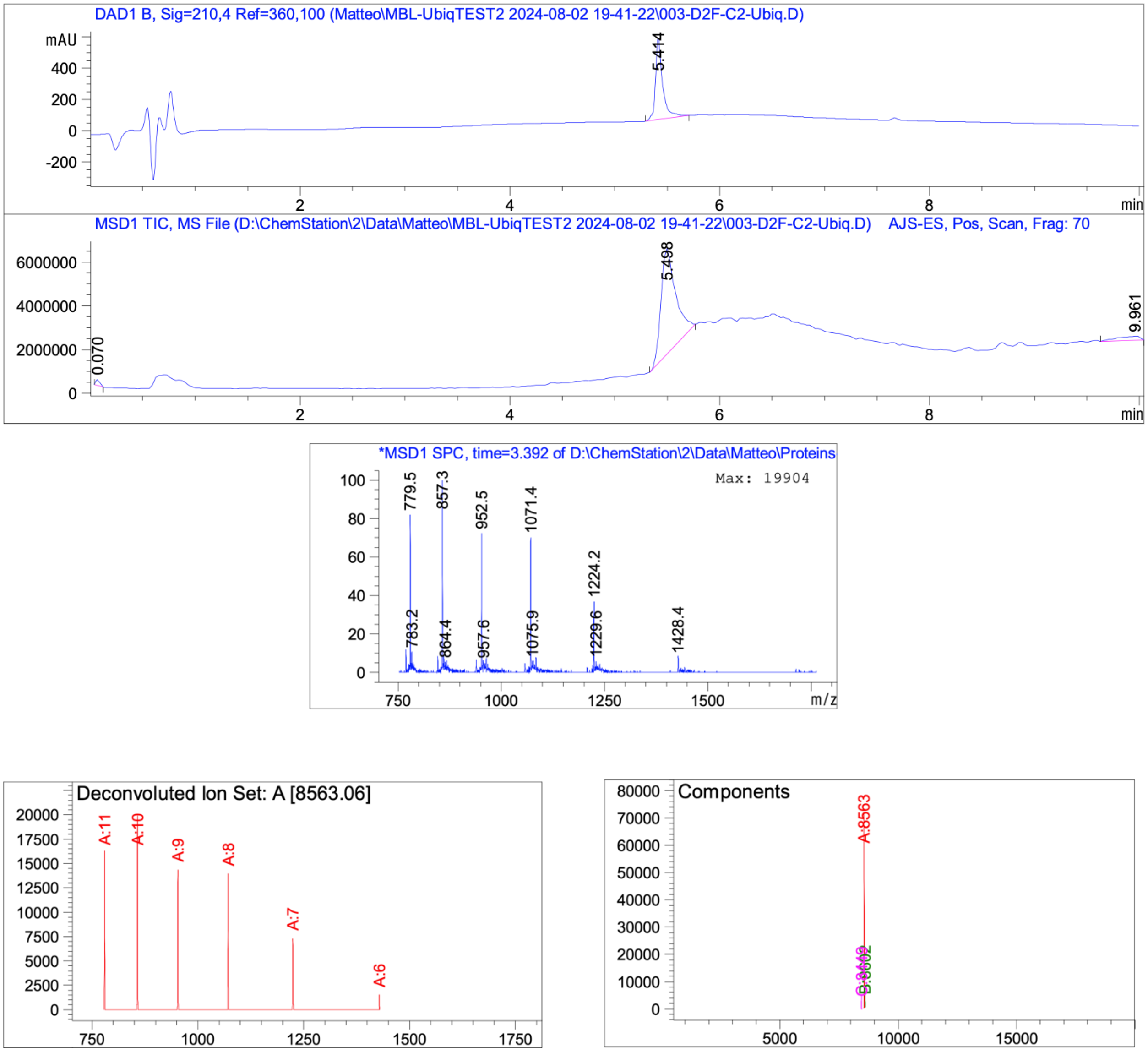

UHPLC-MS analysis (TIC and MS spectra + deconvoluted spectrum) of crude mixture using Agilent AdvanceBio RP-mAb SB-C8 column over 10 minutes (*method 1* in ***section 2***, R_t = 5.8 min):

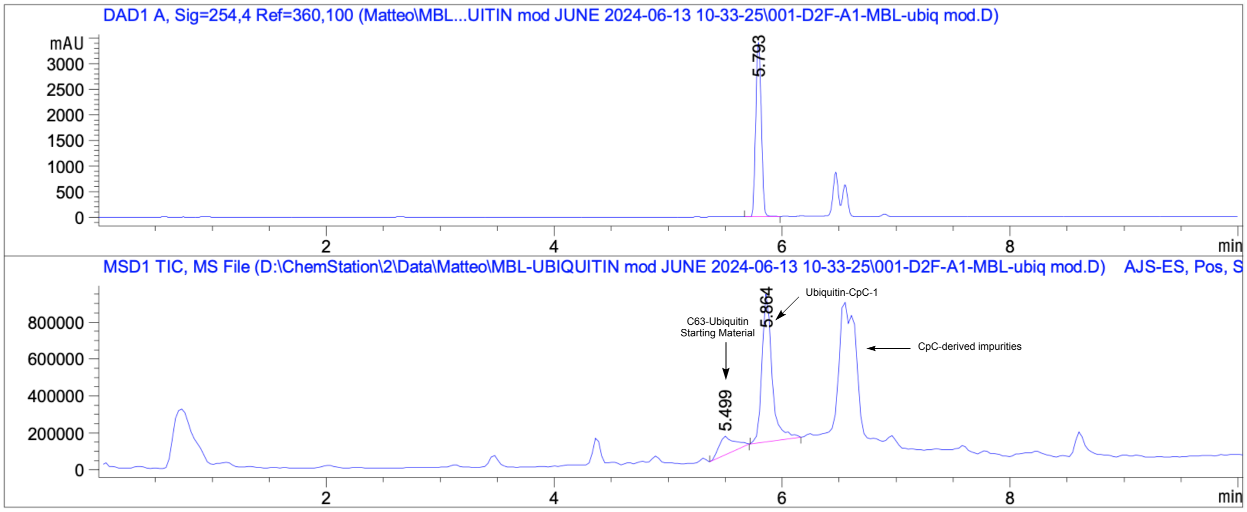


```
Signal 2: MSD1 TIC, MS File

Peak RetTime Type  Width     Area      Height     Area
  #   [min]        [min]                          %
----|-------|----|-------|----------|----------|--------|
   1   5.499 BB    0.1235 9.15800e5  9.99852e4  15.6769
   2   5.864 BB    0.0962 4.92590e6  8.06480e5  84.3231
```

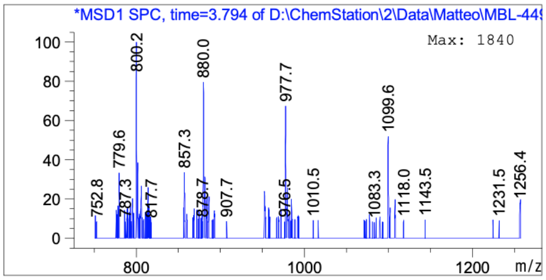

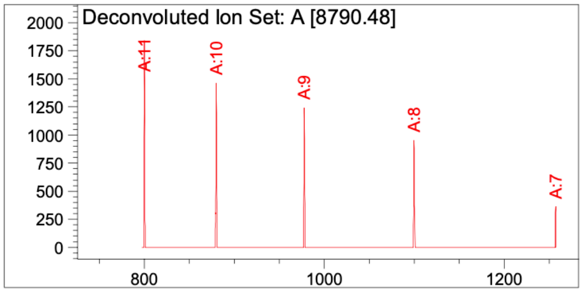

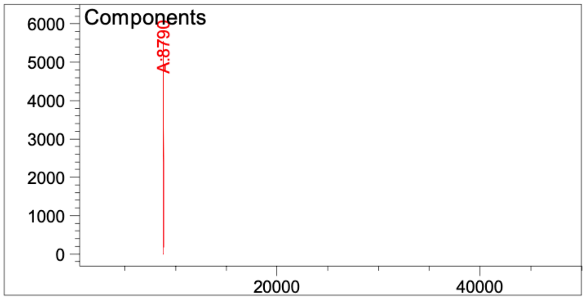

MALDI-TOF-MS analysis of **19**

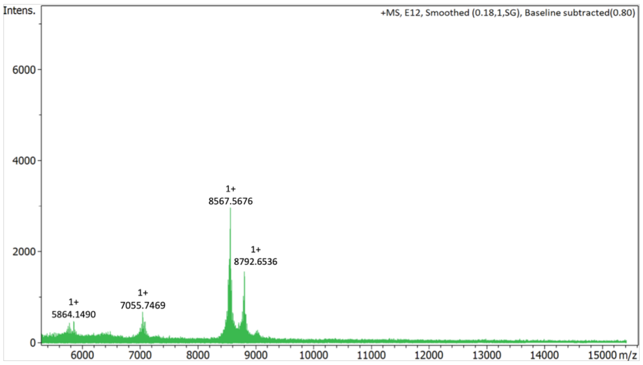

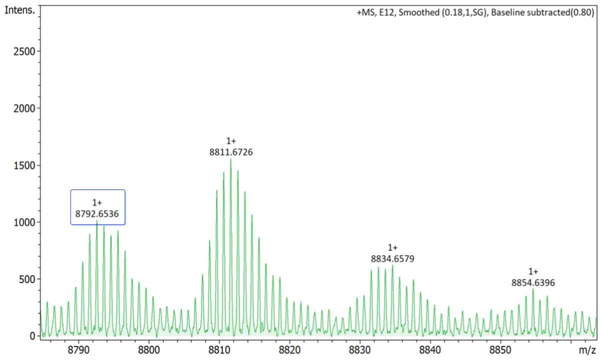

Control test: cyclopropenylation of Affibody-60Glu:

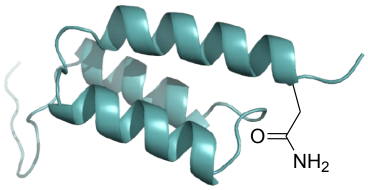

To a 1.5 mL Eppendorf tube were added 100 μL of the 1 mg/mL protein solution in PBS buffer (100 μM) and **CPC 2a** neat (10 mg, 500 equiv.). The tube was vortexed few seconds and the mixture was stirred for 15 minutes at 37 ºC in an Eppendorf thermomixer. The aqueous phase was filtered and analyzed by reverse phase UHPLC-MS using Agilent AdvanceBio RP-mAb SB-C8 column over 10 minutes (*method 1* in ***section 2***). Analysis of the starting protein and the mixture after reaction confirmed that Affibody-60Glu is not reactive.

UHPLC-MS analysis (TIC and MS spectra + deconvoluted spectrum) of *Affibody-60Glu* (Starting Material) using Agilent AdvanceBio RP-mAb SB-C8 column over 10 minutes (*method 1* in ***section 2***, R_t = 2.9 min):

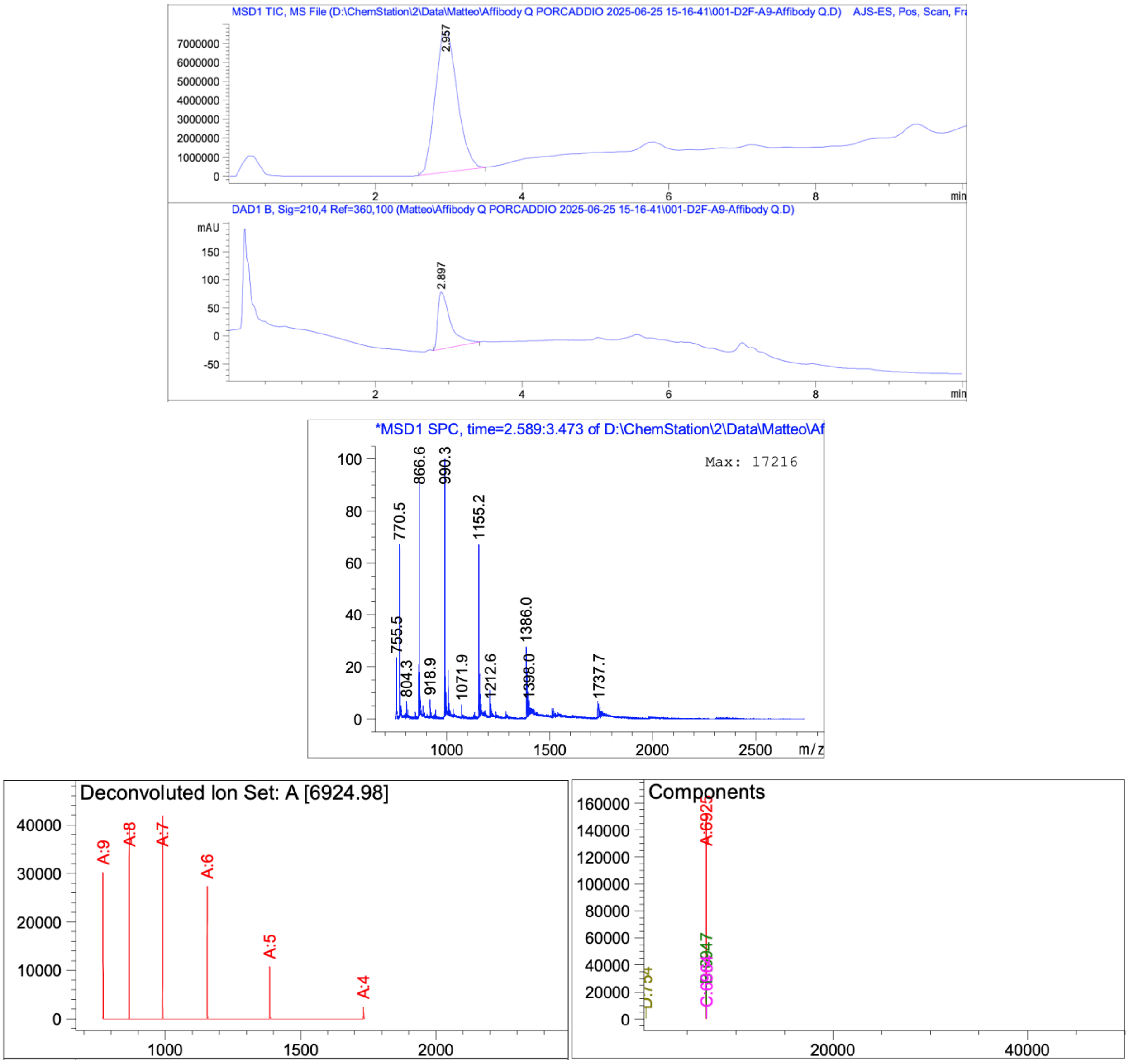

UHPLC-MS analysis (TIC and MS spectra + deconvoluted spectrum) of crude mixture using Agilent AdvanceBio RP-mAb SB-C8 column over 10 minutes (*method 1* in ***section 2***, R_t = 5.8 min):

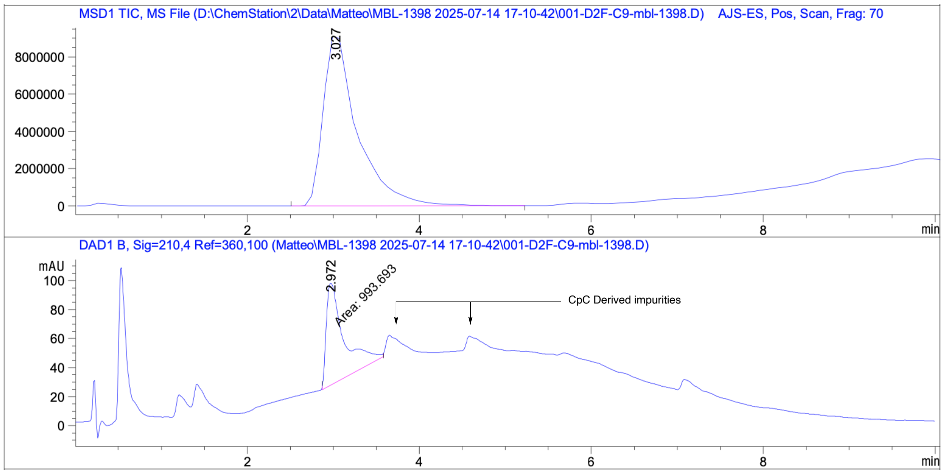

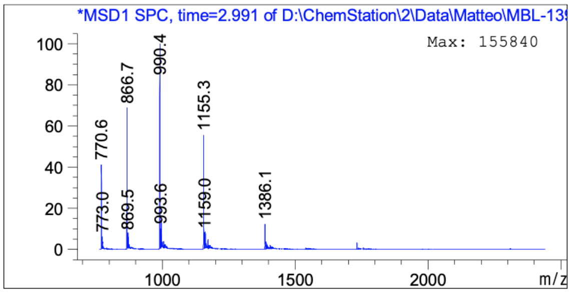

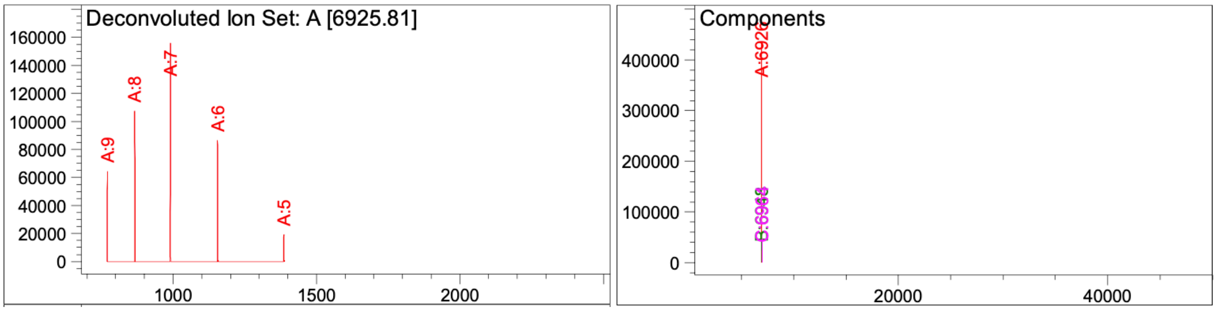

Circular Dichroism of Proteins Bioconjugates

Circular Dichroism of the proteins was measured using a Chirascan CD Spectrometer. Spectra were taken using a 0.1 mM diluted solution of the protein in water. After bioconjugation reaction PBS buffer was exchanged to water using Amicon® Pro Purification System for Dialysis.

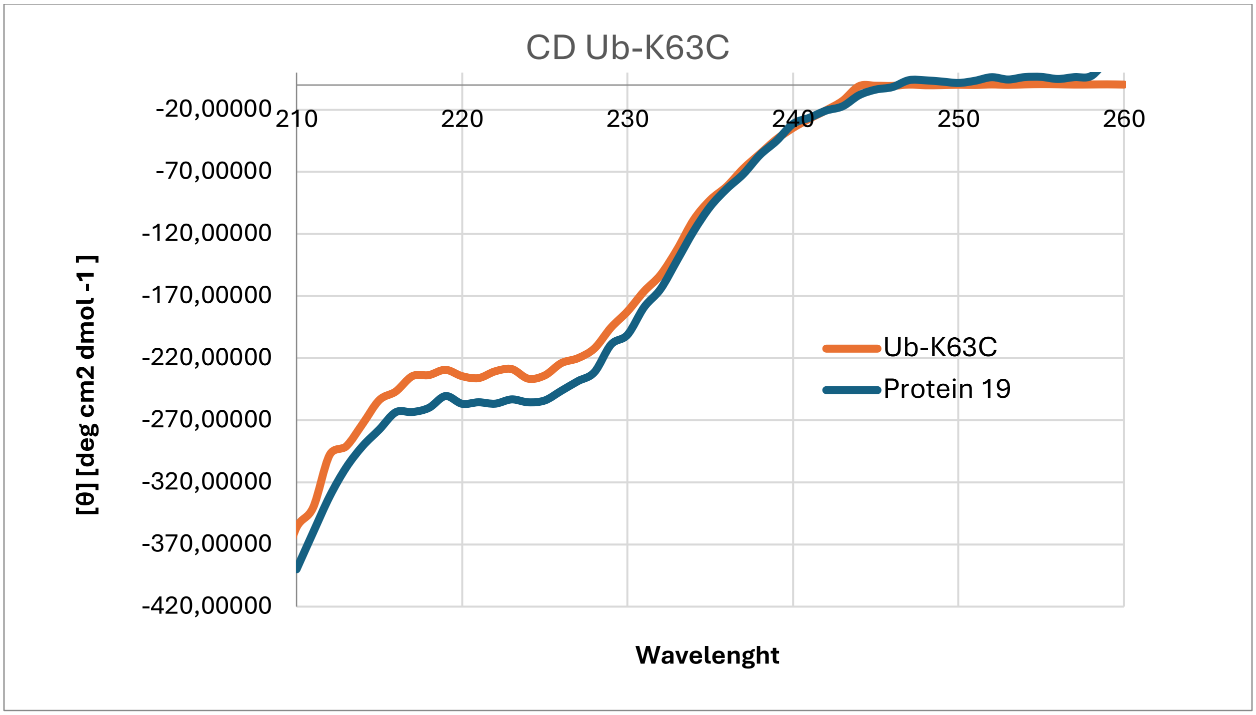

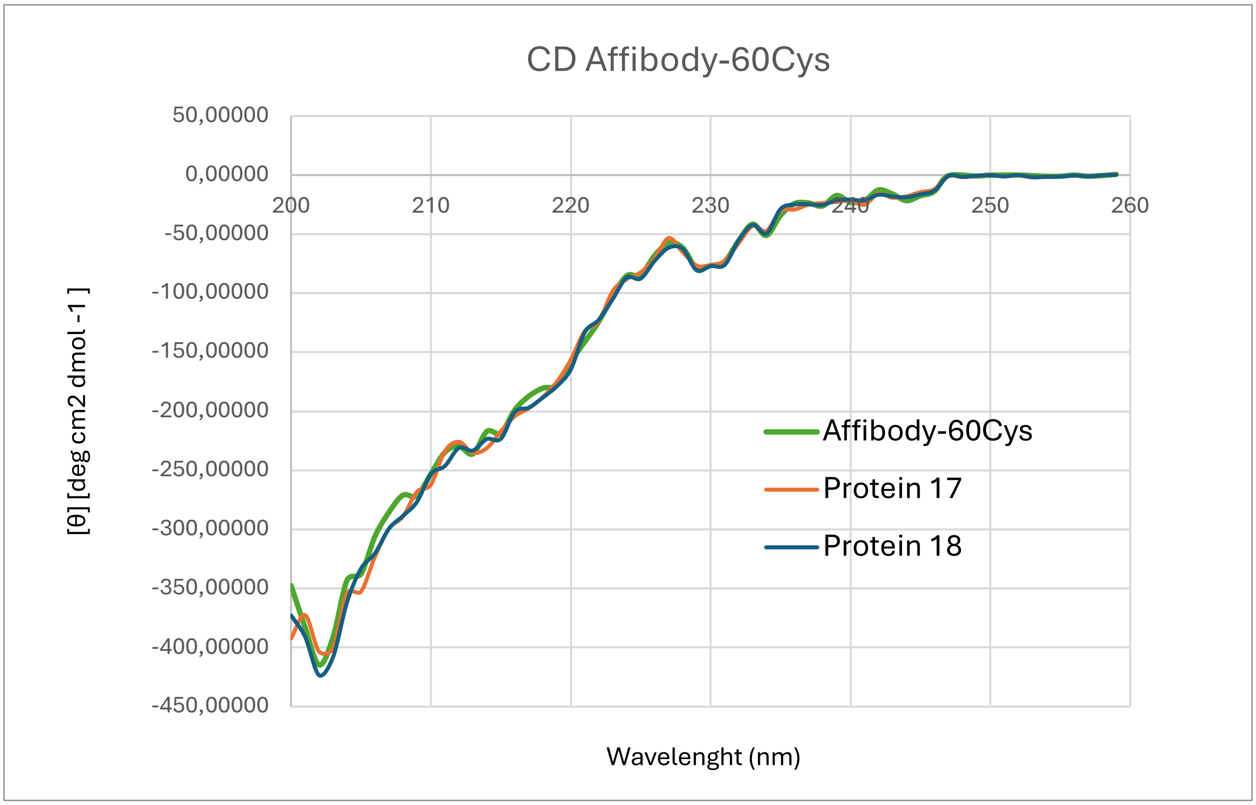

Figure S5: Circul Dichroism Analysis of Proteins

Digestion of cyclopropenylated Affibody-Cys and MS analysis of peptide fragments

The tryptic digestion of modified Affibody-Cys and subsequent MS analysis of the peptide mixture were conducted in the facilities of the Centre for Genomic Regulation (CGR) in Barcelona (Spain). To the solution of modified Affibody-Cys solid urea was first added to help denaturation. The mixture was digested with trypsin and purified by off-line reverse phase. The resulting peptide mixture was analyzed by LCMS in an Orbitrap Fusion Lumos, with a chromatographic gradient of 60 min. Full scan MS were acquired at a resolution of 120K, peptides were fragmented using HCD and collision energy 28%, and fragmentation spectra were acquired in the Orbitrap at a resolution of 30K.

Predicted peptide fragments:

Fragment	*Location*	*MW*	*Sequence*
1	A(1-9)	759.4	GGGGGVDNK
2	A(10-12)	407.2	FNK
3	A(13-32)	2485.2	EQQNAFYEILHLPNLNEEQR
4	A(33-40)	919.5	NAFIQSLK
5	A(41-54)	1457.7	DDPSQSANLLAEAK
6	A(55-55)	146.1	K
7	A(56-63)	830.4 + 139 (modif.)	LNDACAPK

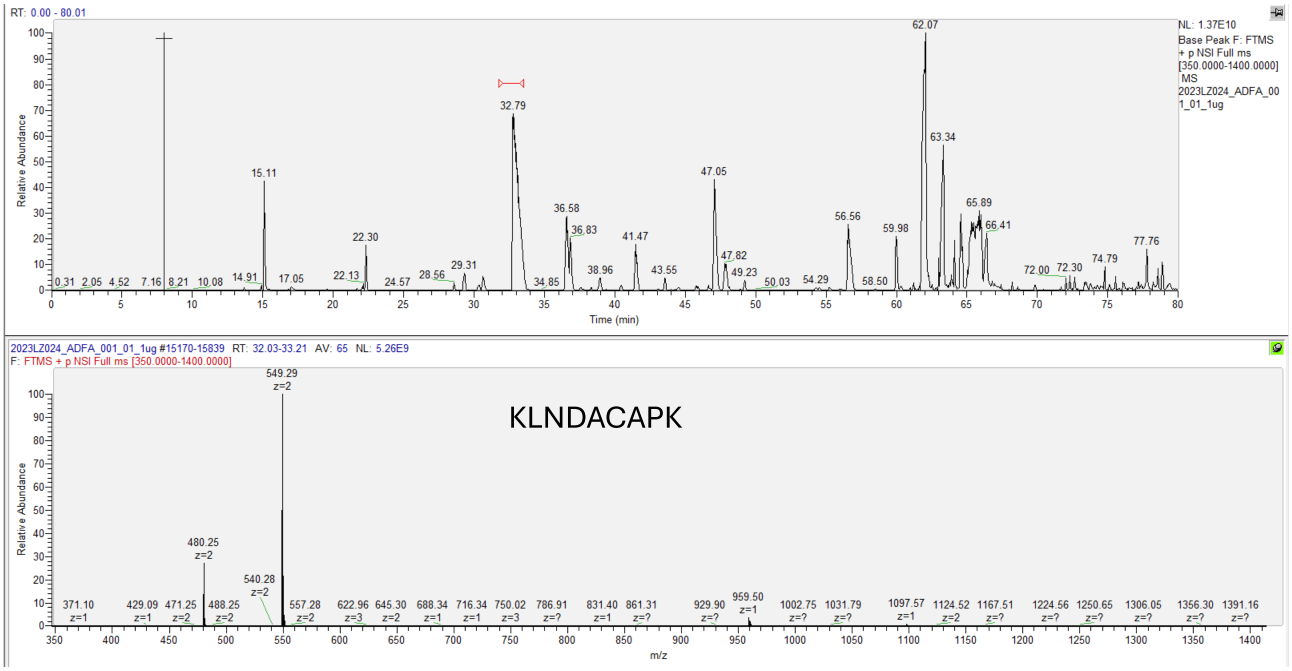

At a retention time of 32.79 min, m/z=480.25 corresponds to peptide KLNDACAPK (Z=2) and m/z=549.29 corresponds to the modification of interest in peptide KLNDACAPK (Z=2). The modification was lost in the gas phase when the molecule was ionized before fragmentation took place. Therefore, MS/MS analysis of m/z=480.25 only shows unmodified peptide fragments, whereas MS/MS analysis of m/z=549.29 only shows the loss of the modification.

Fragmentation spectrum of m/z=480.25

Theoretical fragments:

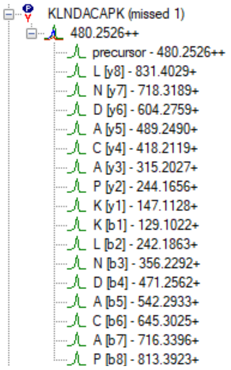

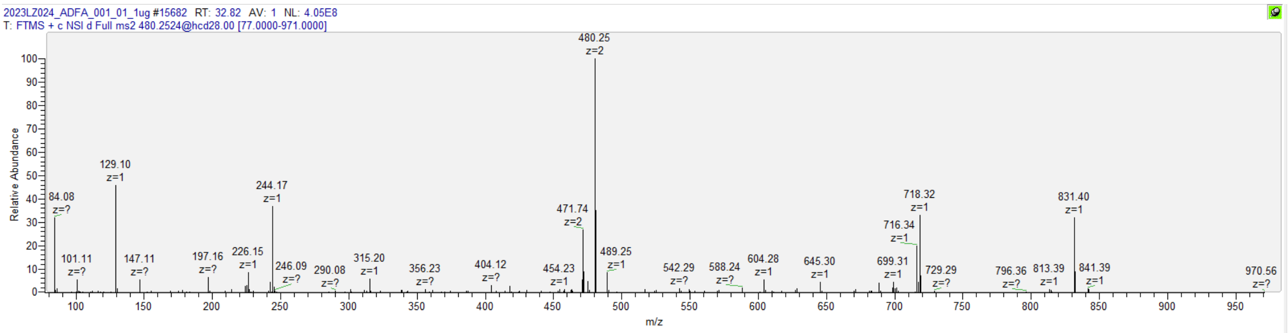

Fragmentation spectrum of m/z=549.29

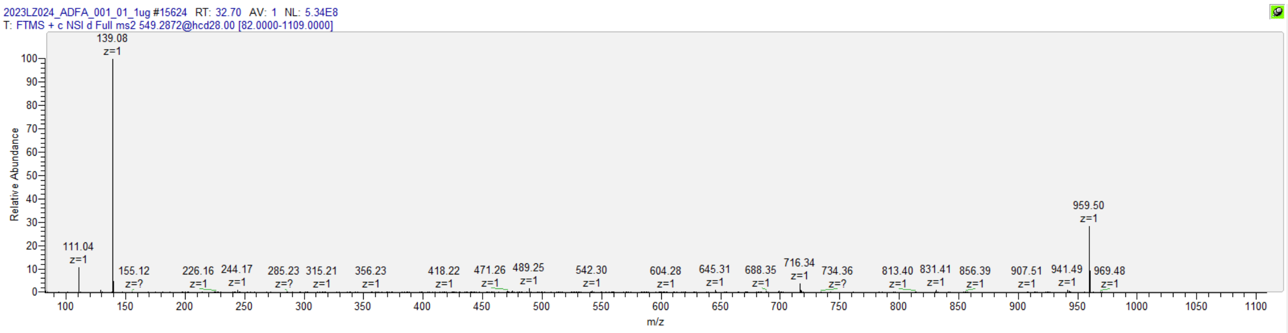

At a retention time of 41.47 min, m/z=969.47 and m/z=485.24 correspond to peptide LNDACAPK with the modification of interest (Z=1 and Z=2, respectively) and m/z=831.40 corresponds to unmodified peptide LNDACAPK (Z=1). The modification was lost, again, in the gas phase when the molecule was ionized before fragmentation took place. Therefore, MS/MS analysis of m/z=485.24 only shows the loss of the modification.

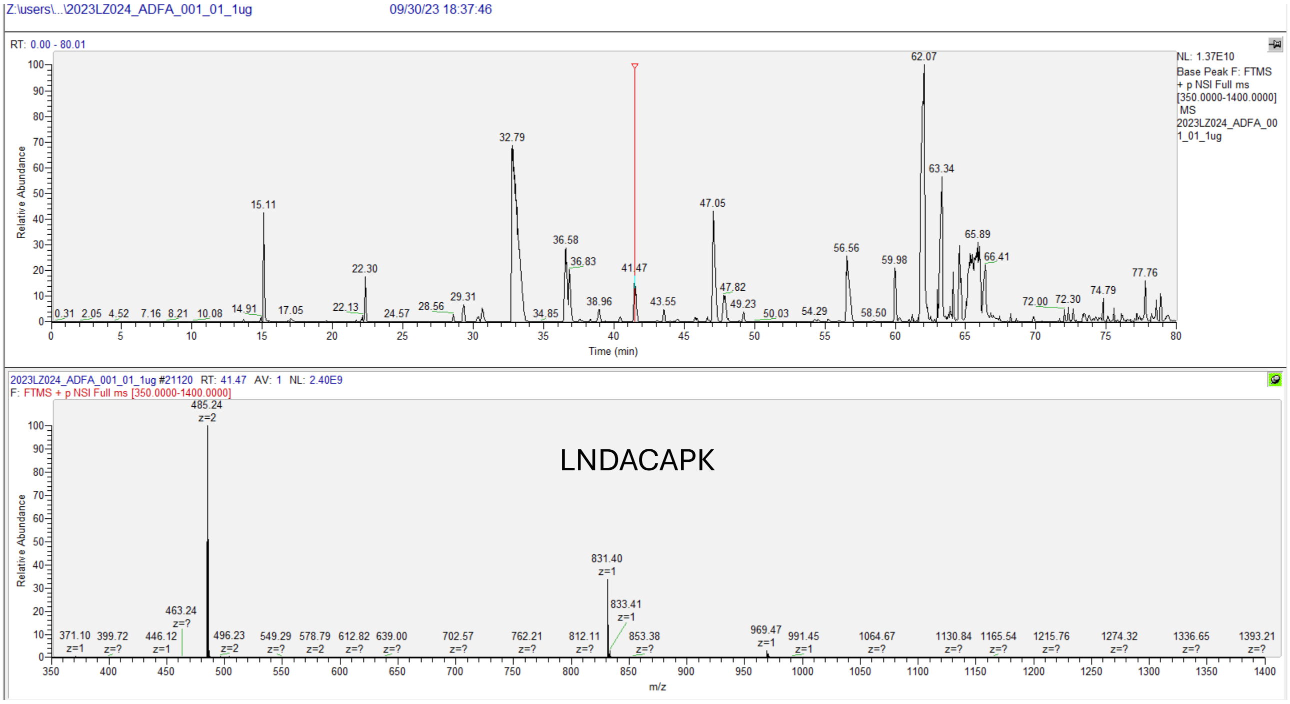

Fragmentation spectrum of m/z=485.24

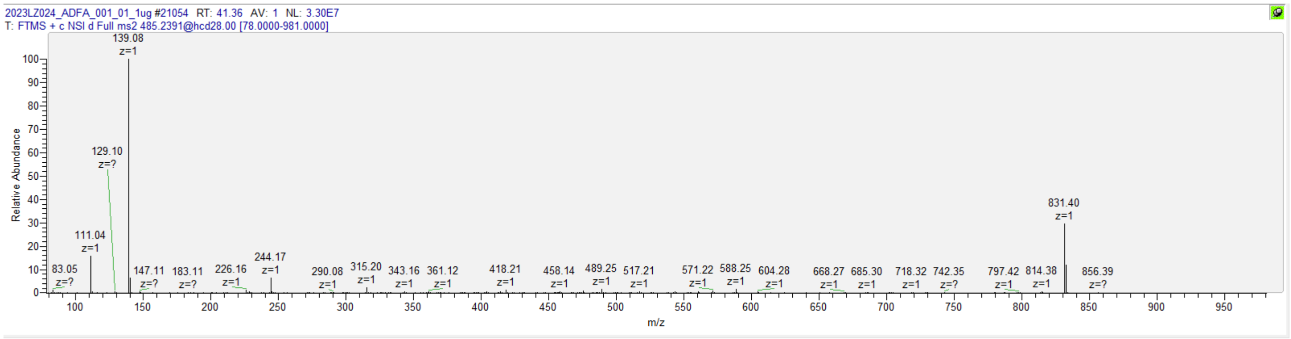

8. Thiol-ene Reaction

General Procedure for the Thiol-ene reaction using the bioconjugate mimic:

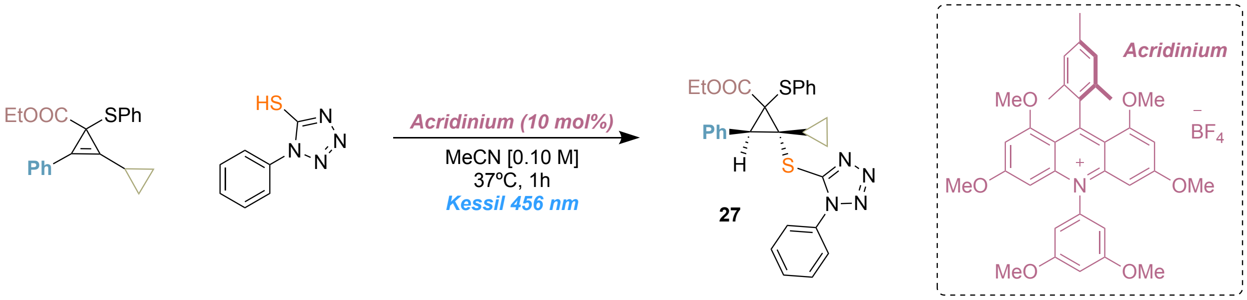

To a 10 mL Schlenk tube equipped with a magnetic stirring bar, cyclopropene was added (1 eq, 0.2 mmol, 66 mg) followed by MeCN (2mL, 0.1M previously degassed) and 1-Phenyl-1H-tetrazole-5-thiol (1.5 eq, 0.3 mmol, 53 mg). Finally acridinium (5 mol%, 6mg) was added to the mixture. The tube was sealed and irradiated under 456nm Kessil Lamp for 18h, maintaining the temperature at 37ºC using a cooling fan. After the indicated time the solvent was removed under reduced pressure and the mixture analized by ^{1}H-NMR. Finally, purification of the crude mixture by silica-gel chromatography delivered product **27** as an inseparable mixture of diastereoisomers (3:1 d.r.) in 78% yield.

^{1}H NMR (400 MHz, $CDCl_3$, *diast1+diast2*) δ. 7.61 – 7.16 (m, 15H *diast1+diast2*); 5.54 (s, 1H, *diast1*); 5.52 (s, 1H, *diast2*); 4.10 (q, 2H, *diast1*); 3.97 (qd, 7.1, 4.4 Hz, 2H, *diast2*); 1.77 – 1.65 (m, 2H *diast1+diast2*); 1.18 (t, 7.1 Hz, 3H, *diast1*); 1.08 (t, 7.1 Hz, 3H, *diast2*) 0.88 – 0.39 (m, 8H, *diast1+diast2*).

^{13}C NMR (101 MHz, $CDCl_3$, *diast1+diast2*) δ 168.9, 168.7, 152.9, 152.8, 145.7, 145.6, 138.6, 137.5, 133.8, 133.6, 133.5, 133.3, 130.3, 130.2, 129.8, 129.7, 129.6, 129.3, 129.2, 129.2, 129.1, 128.9, 128.2, 128.1, 128.2, 127.9, 124.2, 123.7, 62.1, 58.6, 56.7, 31.0, 16.7, 14.8, 13.9, 13.86, 9.4, 8.8, 8.6, 8.2.

*GOESY Spectra of **27a** for the relative stereochemical assignment:*

As seen by the above GOESY Spectra, there's a strong spacial interaction between the benzilic proton and the tertiary proton of the cyclopropane unit in the minor diasteroisomer. Another important interaction can be seen between the CH_2 protons of the ester moiety and the benzylic proton of the major diasteroisomer.

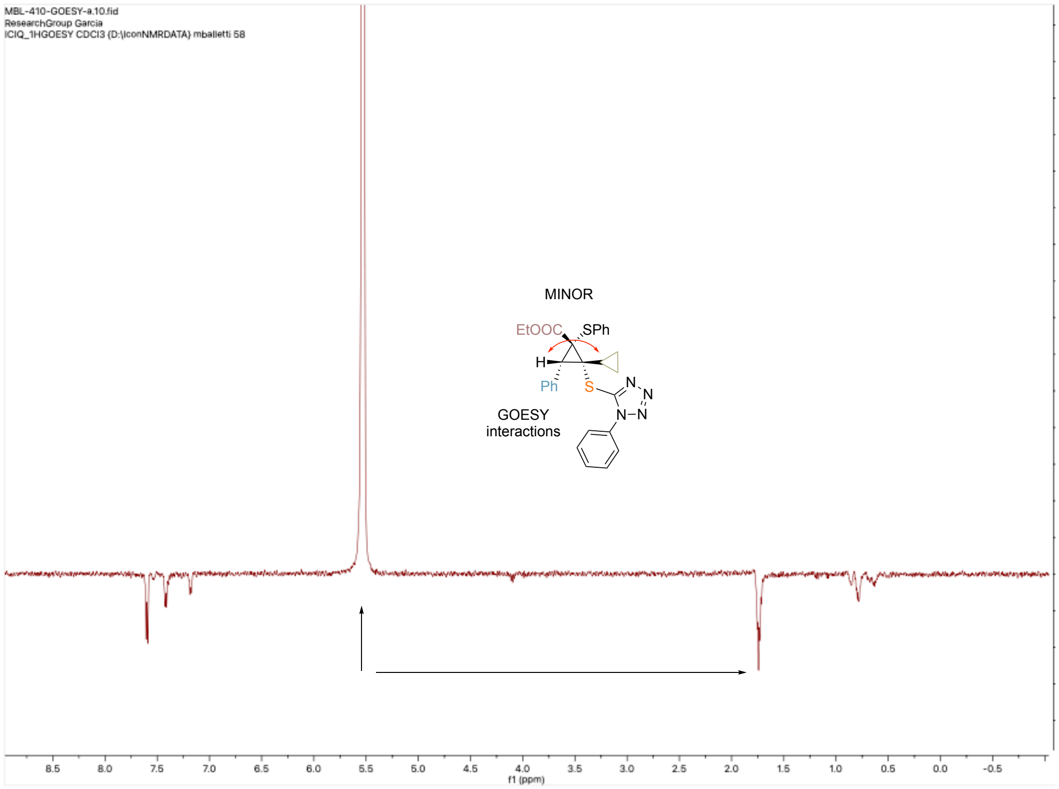
MBL-410-GOESY-a.10.fid
ResearchGroup Garcia
ICIQ_1HGOESY CDCl3 {D:\IconNMRDATA} mballetti 58
MINOR
EtOOC
SPh
H
Ph
S
N
N
N
N
GOESY
interactions
f1 (ppm)

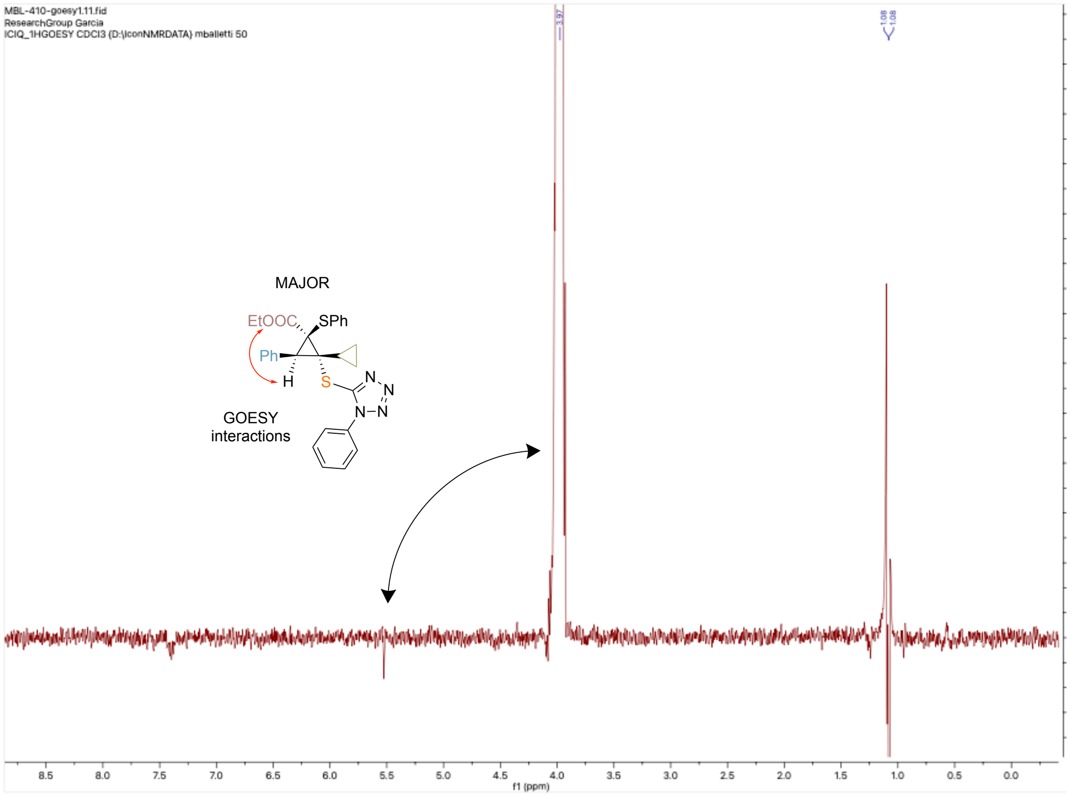
MBL-410-goesy1.11.fid
ResearchGroup Garcia
ICIQ_1HGOESY CDCl3 {D:\IconNMRDATA} mballetti 50
MAJOR
EtOOC
SPh
Ph
H
S
N
N
N
N
GOESY
interactions
f1 (ppm)

General Procedure for the bio-orthogonal Thiol-ene reaction:

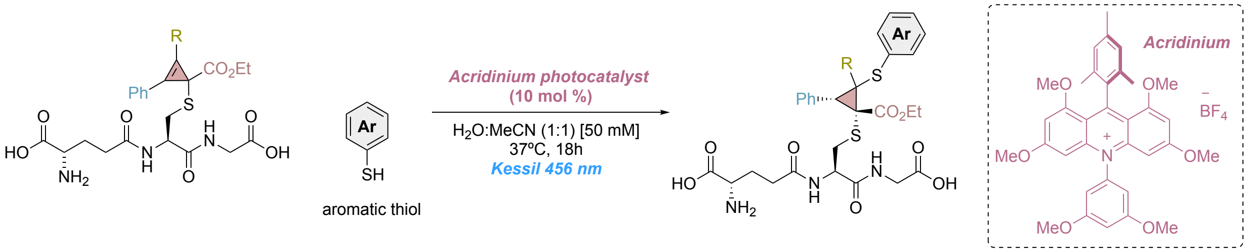

In a 1.5 mL vial equipped with a stirring bar, was added a 50 mM solution (H_2O:MeCN=1:1) of bioconjugate, followed by the (hetero)aromatic thiol (10 eq.) and the Acridinium catalyst 10% (using a 80 mM stock solution of acridinium in H_2O:MeCN=1:1). The vial was sealed and irradiated for 18 hours with a 465 Kessil Lamp. The crude mixture was diluted (1 mL H_2O:MeCN=1:1) and analyzed by UHPLC-MS using column Zorbax 300 SB-C8 over 10 minutes (*method 1* in ***section 2***). **Note**: all the solvents were previously degassed by N_2 bubbling

UHPLC-MS Traces of the products

20

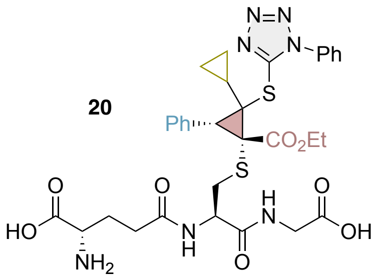

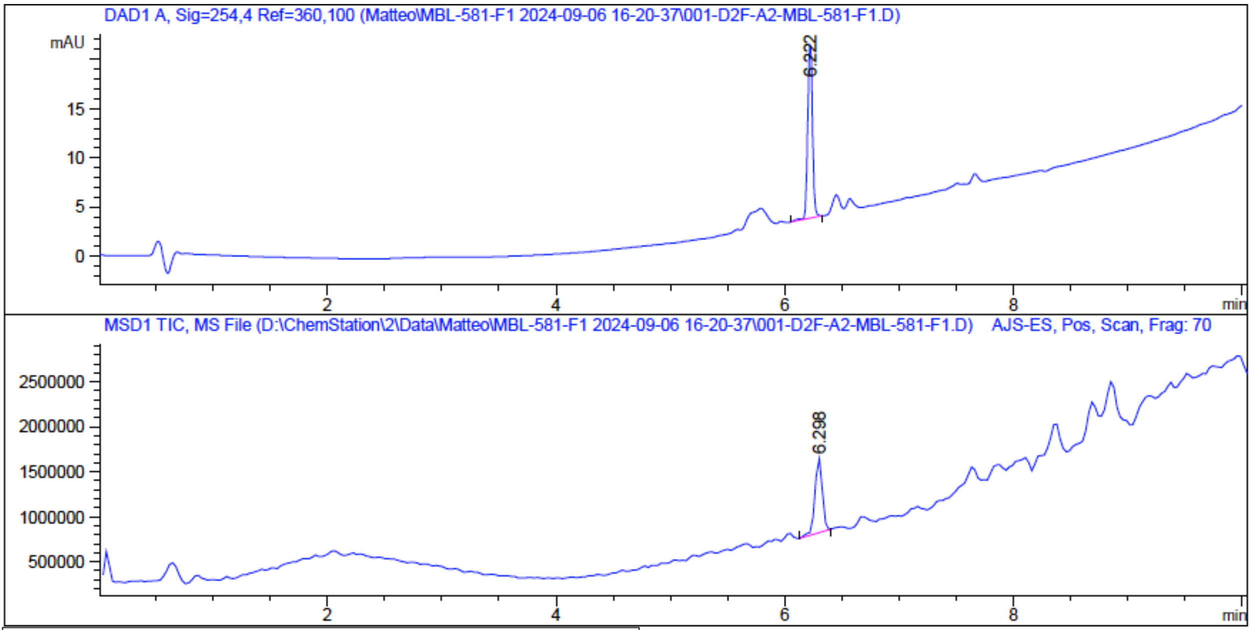

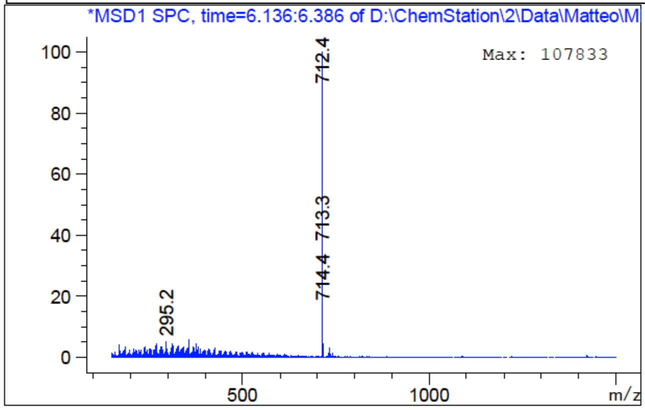

Signal 2: MSD1 TIC, MS File

Peak #	RetTime [min]	Type	Width [min]	Area	Height	Area %
1	6.298	BB	0.0692	3.73377e6	8.29059e5	100.0000

Peak #1 at 6.298 min (6.136 to 6.397 min)

21

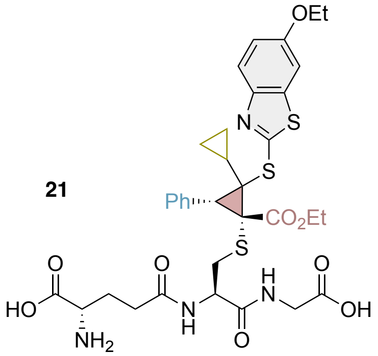

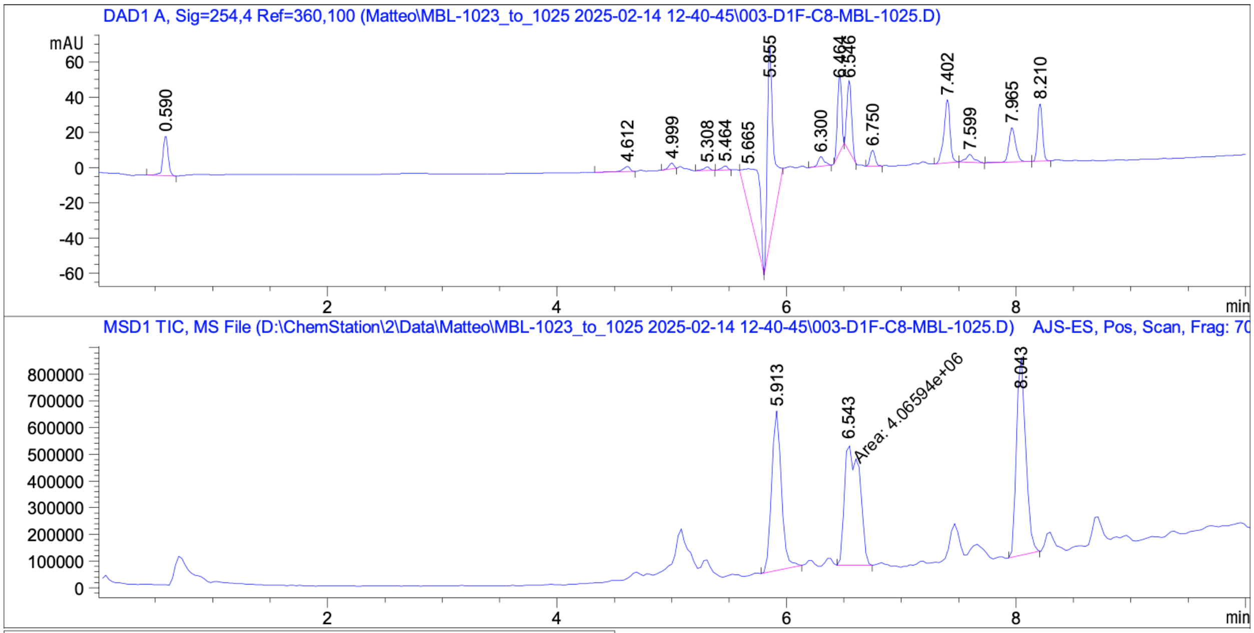

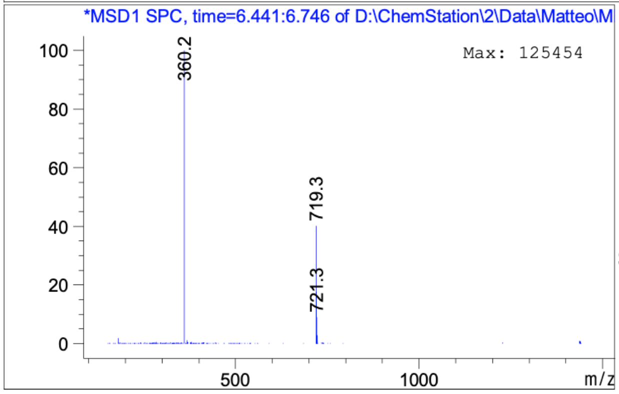

Signal 2: MSD1 TIC, MS File

Peak #	RetTime [min]	Type	Width [min]	Area	Height	Area %
1	5.913	BB	0.0938	3.53844e6	5.99026e5	30.2340
2	6.543	MM	0.1484	4.06594e6	4.56574e5	34.7413
3	8.043	BB	0.0889	4.09911e6	7.47127e5	35.0247

Peak #2 at 6.543 min (6.441 to 6.749 min)

22

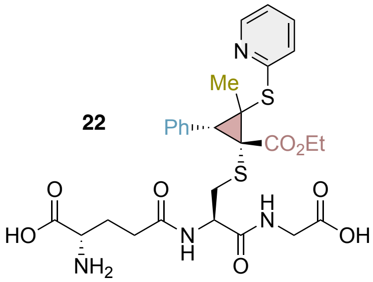

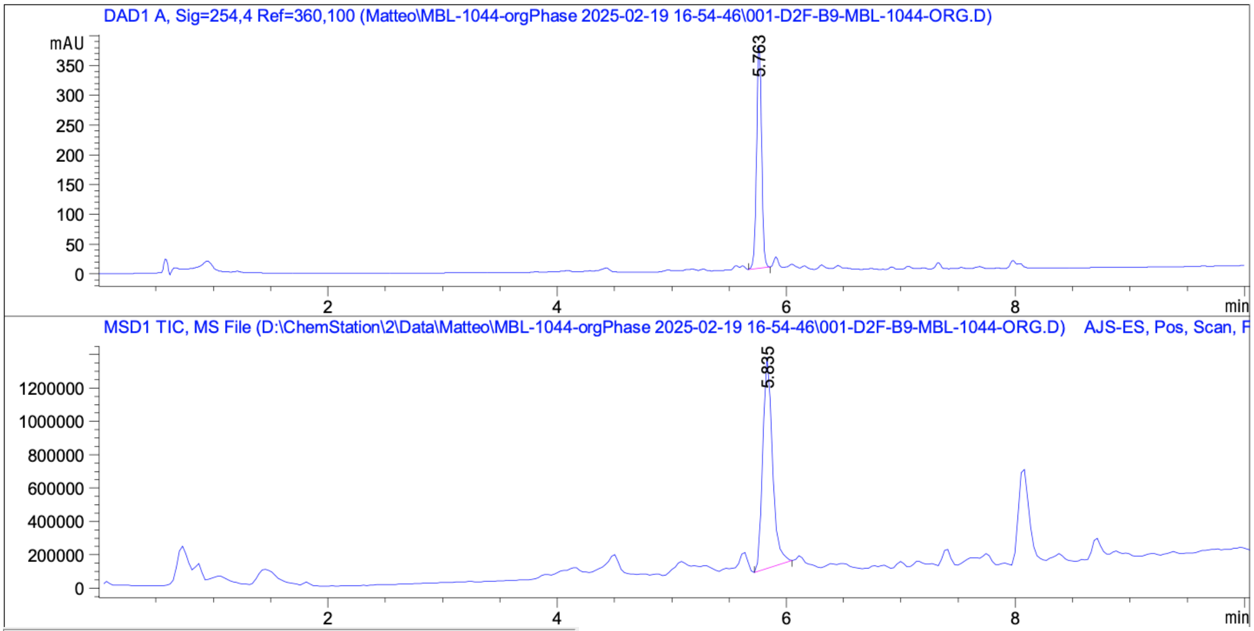

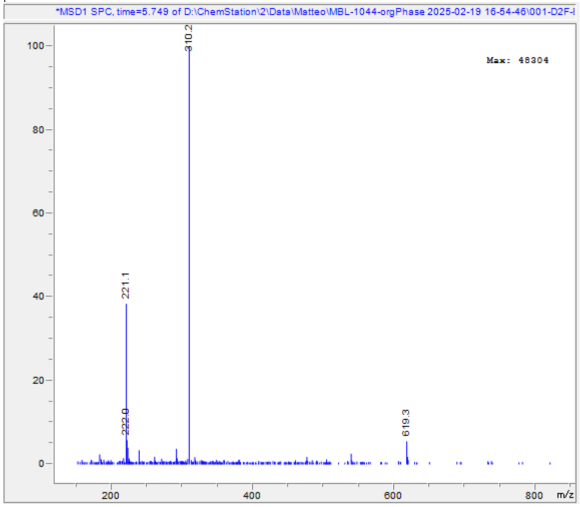

Signal 2: MSD1 TIC, MS File

Peak #	RetTime [min]	Type	Width [min]	Area	Height	Area %
1	5.835	BB	0.0936	7.46941e6	1.26904e6	100.0000

23

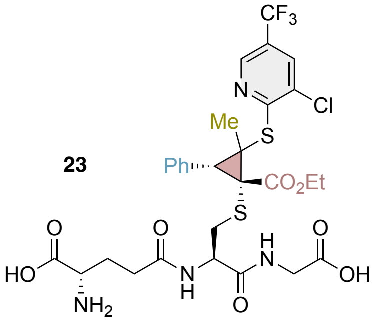

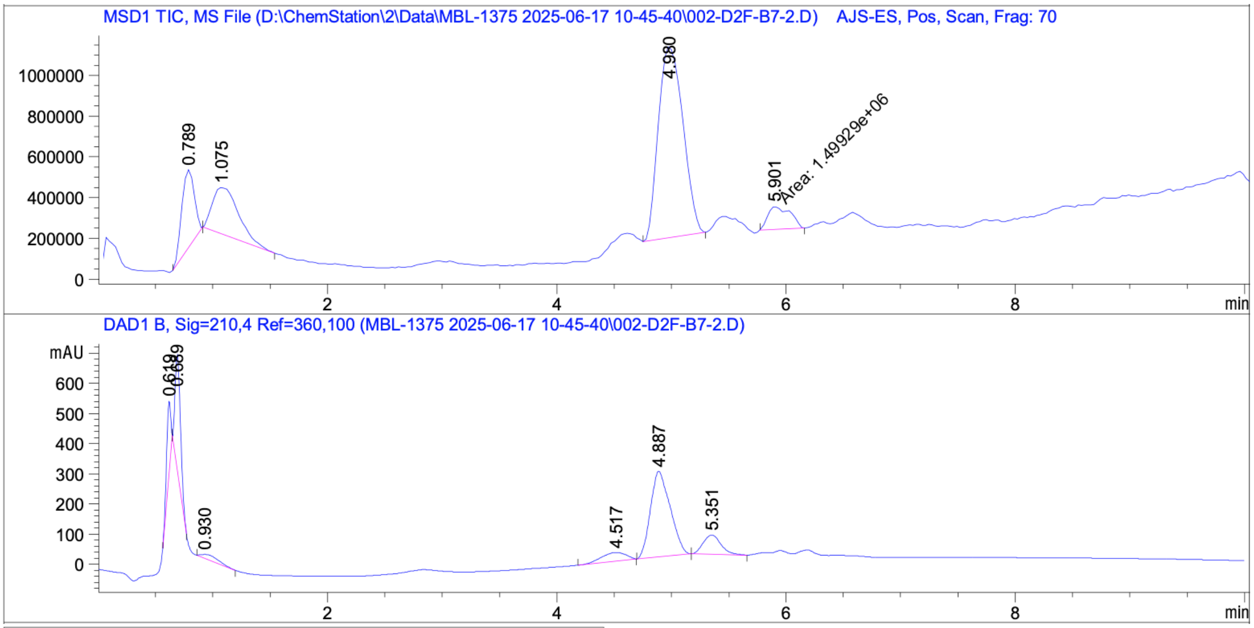

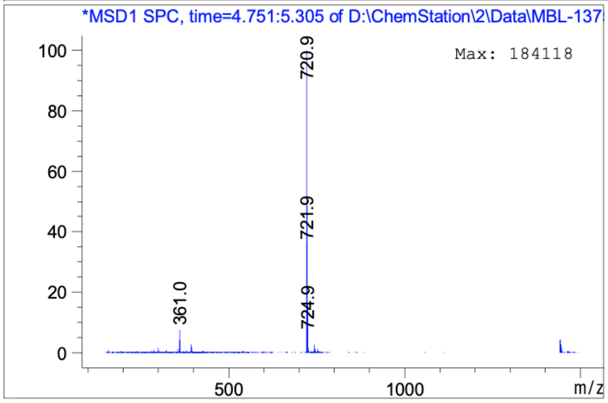

Peak #3 at 4.980 min (4.751 to 5.296 min)

Signal 2: MSD1 TIC, MS File

Peak #	RetTime [min]	Type	Width [min]	Area	Height	Area %
1	0.789	BB	0.1231	2.91916e6	3.79266e5	13.2782
2	1.075	BB	0.2477	3.69389e6	2.27407e5	16.8022
3	4.980	BB	0.2470	1.38722e7	9.39782e5	63.0999
4	5.901	MM	0.2237	1.49929e6	1.11693e5	6.8198

24

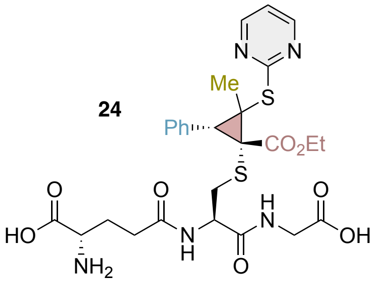

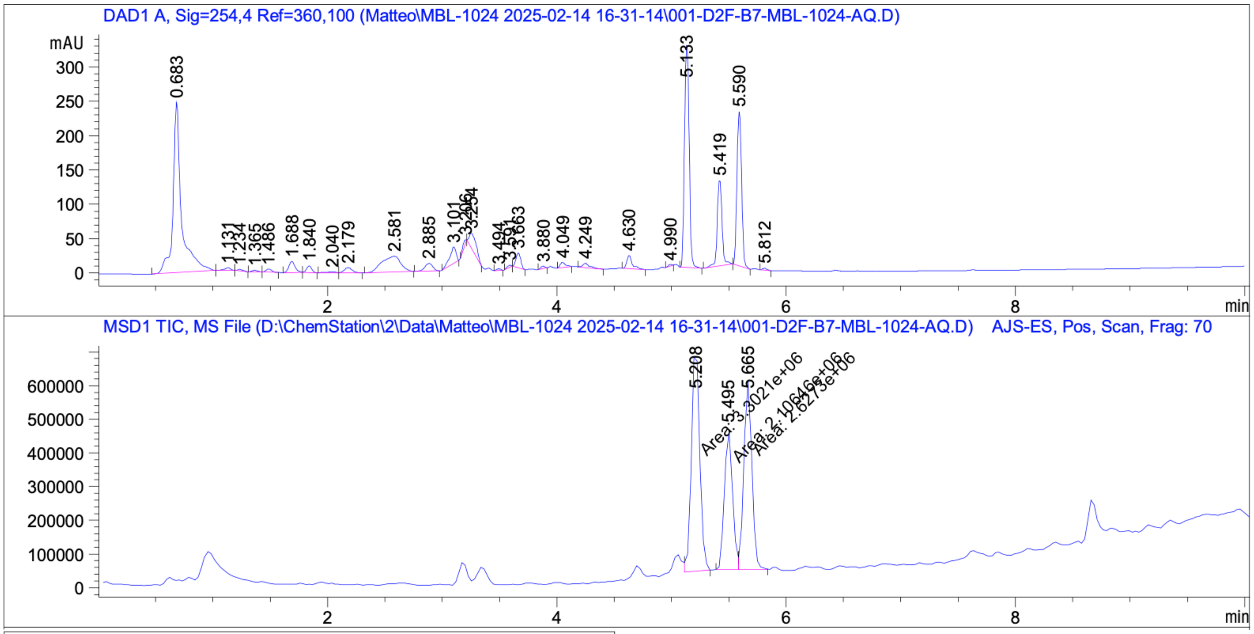

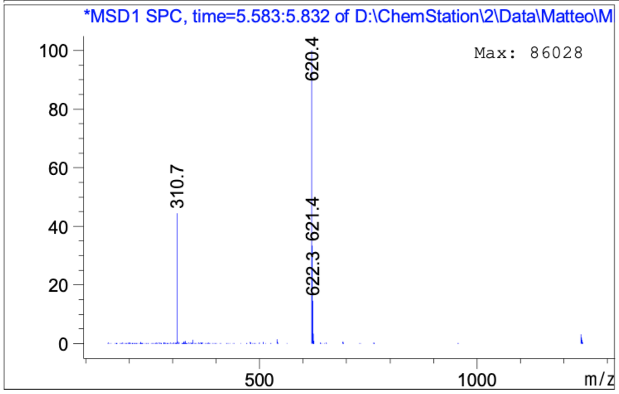

Peak #3 at 5.665 min (5.584 to 5.842 min)

Signal 2: MSD1 TIC, MS File

Peak #	RetTime [min]	Type	Width [min]	Area	Height	Area %
1	5.208	FM	0.0795	3.30210e6	6.91911e5	41.0920
2	5.495	MF	0.0828	2.10646e6	4.23974e5	26.2133
3	5.665	FM	0.0766	2.62730e6	5.72018e5	32.6947

25

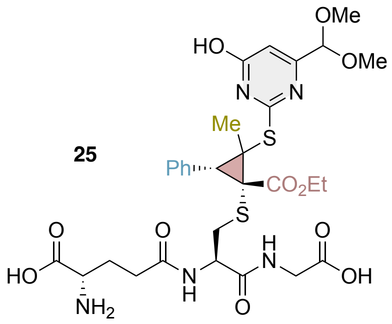

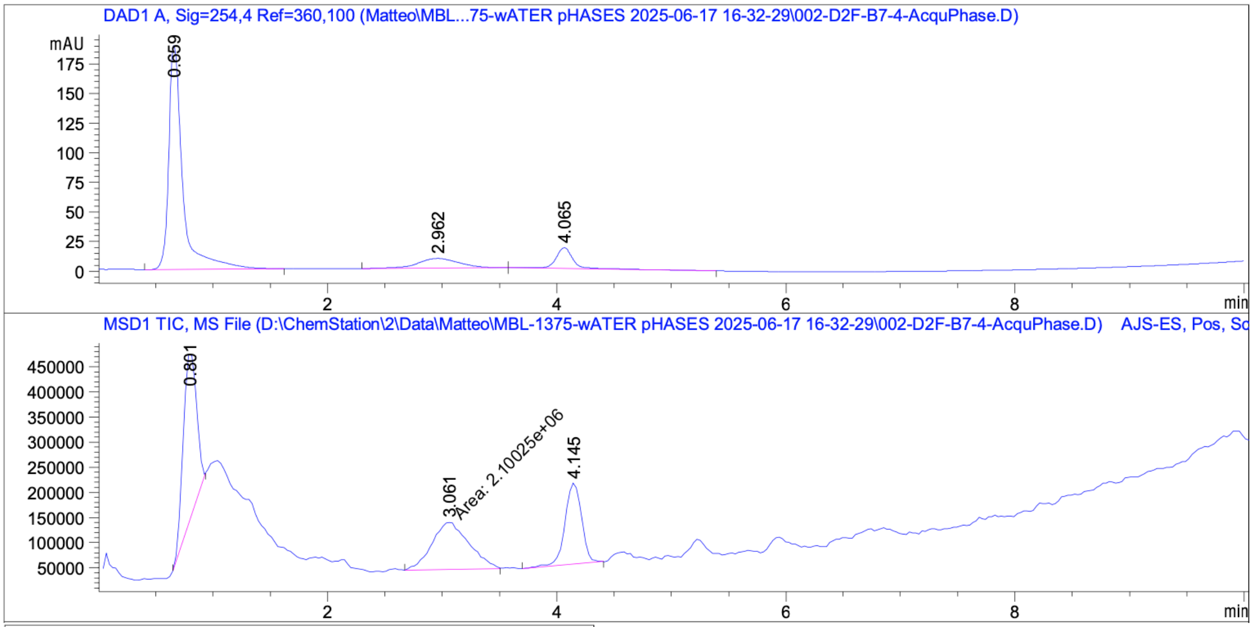

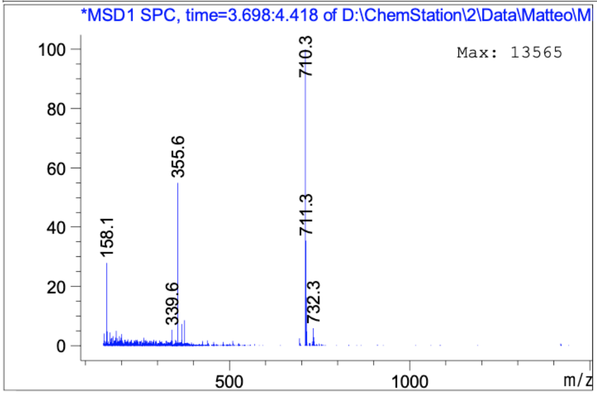

Peak #3 at 4.145 min (3.698 to 4.410 min)

Signal 2: MSD1 TIC, MS File

Peak #	RetTime [min]	Type	Width [min]	Area	Height	Area %
1	0.801	BB	0.1262	2.67572e6	3.35899e5	41.7766
2	3.061	MM	0.3715	2.10025e6	9.42150e4	32.7917
3	4.145	BB	0.1679	1.62886e6	1.61071e5	25.4317

26

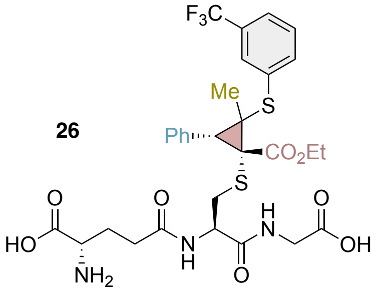

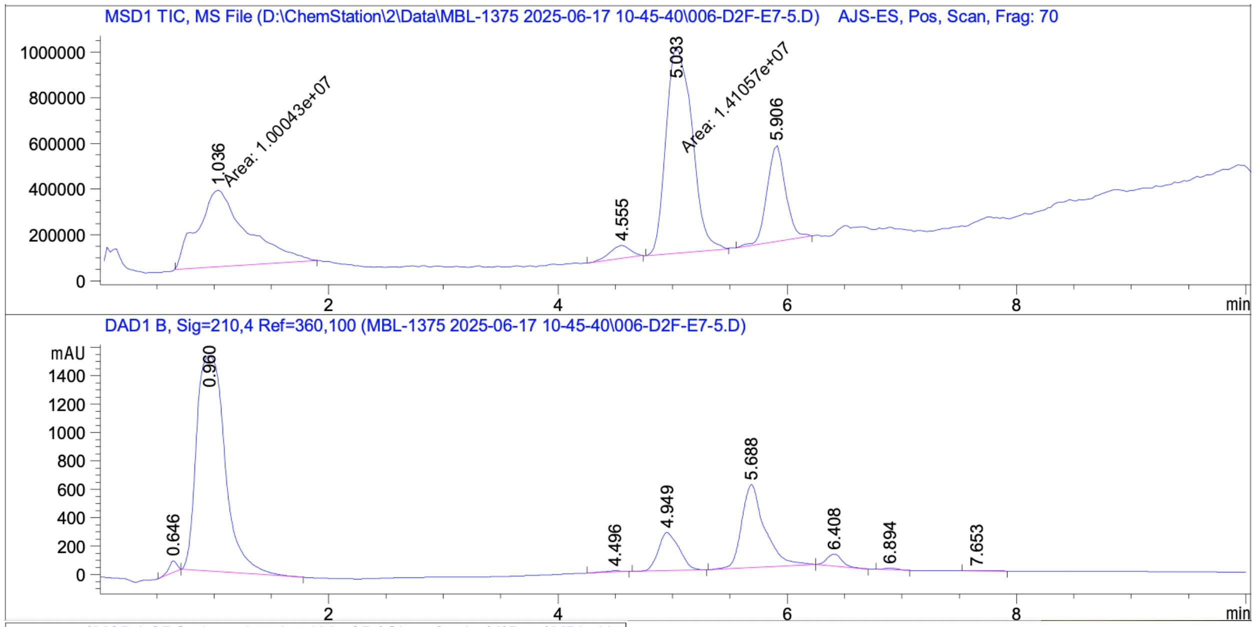

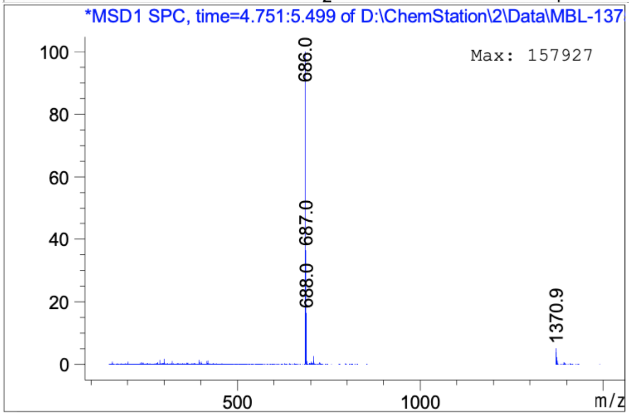

Peak #3 at 5.033 min (4.765 to 5.489 min)

Signal 2: MSD1 TIC, MS File

Peak #	RetTime [min]	Type	Width [min]	Area	Height	Area %
1	1.036	MM	0.4962	1.00043e7	3.36020e5	33.8972
2	4.555	BB	0.1793	7.17661e5	5.73369e4	2.4316
3	5.033	MM	0.2594	1.41057e7	9.06142e5	47.7939
4	5.906	BB	0.1713	4.68596e6	4.21240e5	15.8773

Stern-Volmer Quenching Studies

Fluorescence measurements were carried out on an Aminco-Bowman Series 2 Luminescence spectrofluorimeter equipped with a high voltage PMT detector and continuum Xe light source. A $2x10^{-5}$ M stock solution of acridinium Photocatalyst in degassed MeCN:H2O (1:1) was prepared. A 0.5 M solution of 1-Phenyl-1H-tetrazole-5-thiol substrate (quencher) in degassed MeCN:H2O (1:1) was prepared. In a quartz cuvette 2 mL of acridinium stock solution was added followed by

20 μL of quencher solution. The addition of the substrate solution (quencher) was repeated for five/six times. After each addition, the solution was mixed and the emission spectra of the excited catalyst was acquired from 380 nm to 500 nm (the excitation wavelength was fixed at 370 nm). A solvent blank was subtracted from all the measurements. The excitation wavelength was chosen in order to avoid saturation of the emission detector. The results shown in Figure S7 and S8 indicates that the Thiol quenched the excited state emission of the Acridinium catalyst better than the bioconjugate K_{SV} Thiol = 0.0187 and K_{SV} Bioconjugate = $6x10^{-5}$. The Stern-Volmer plot shows a linear correlation between the amounts of substrates and the ratio I_0/I, following the relationship: $I_0/I = 1 + KSV[Q]$ (Q = Quencher).

We therefore consider that the reaction occurs through photoexcitation of the acridinium photocatalyst followed by reductive quenching of the acridinium excited state. Deprotonation or the radical cation generates the thiyl radical which attacks the electron rich cyclopropene, delivering a stabilized benzylic radical. Further atom abstraction from the solvent or another thiol molecule would deliver the product (Figure S6)

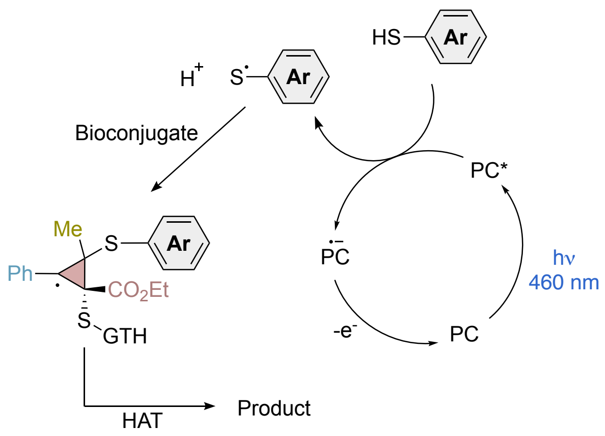

Figure S6: Postulated mechanism of the thiol-ene photocatalyzed reaction

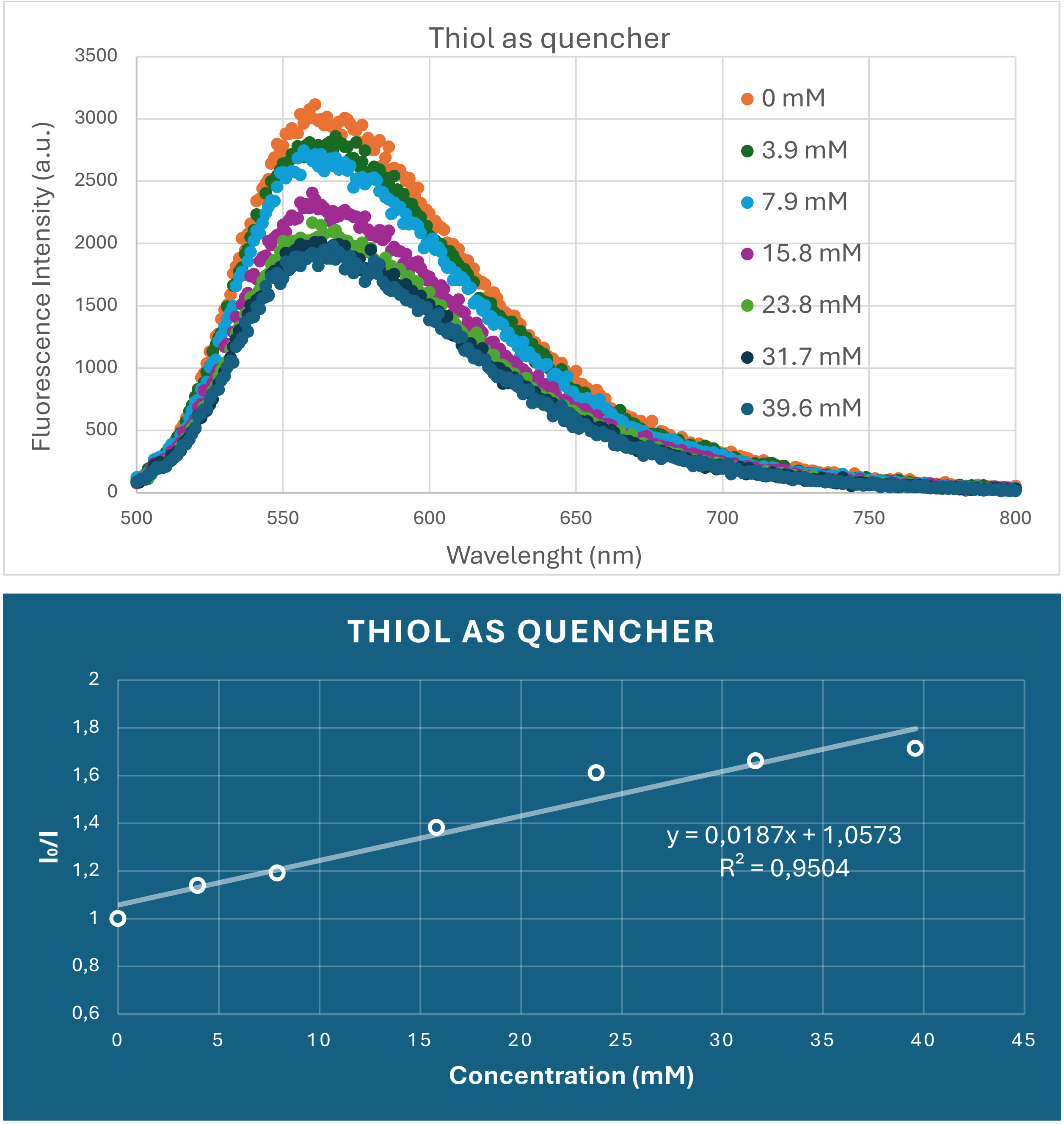

Figure S7: Stern Volmer Quenching Study of 1-Phenyl-1H-tetrazole-5-thiol as quencher

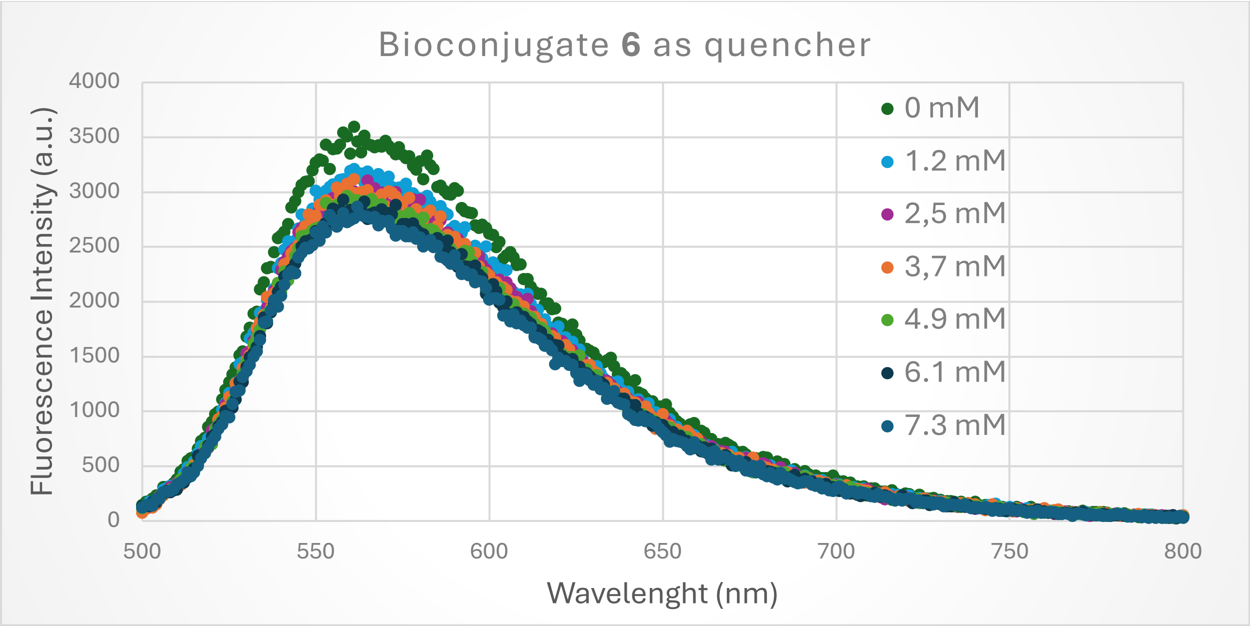

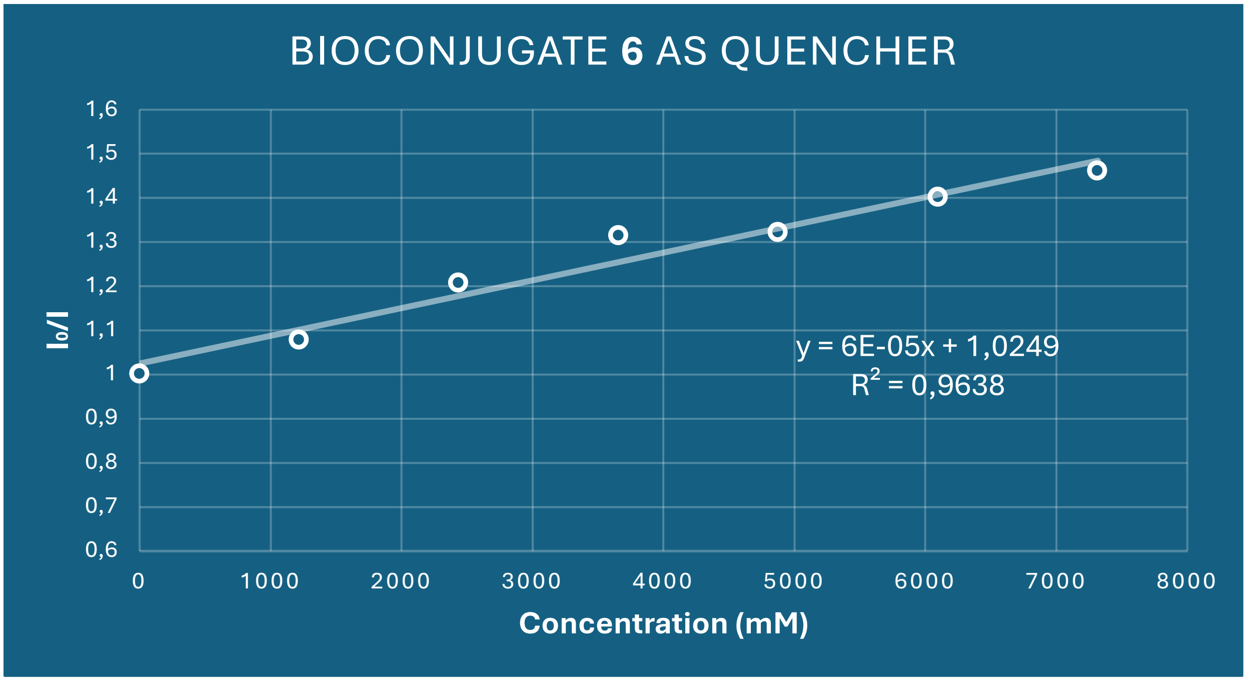

Figure S8: Stern Volmer Quenching Study of bioconjugate **6** as quencher

Unsuccessful Substrates for Thiol-Ene reactivity

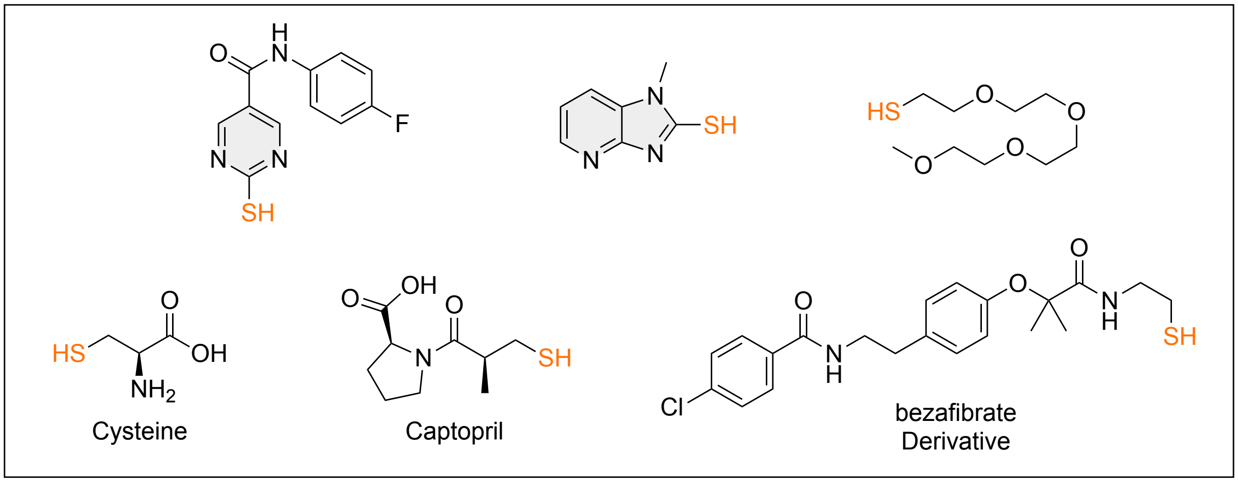

Figure S9: Table of unsuccesfull substrates in the thiol-ene reactivity

9. Unsuccessful Reactivities

<u>*Unsuccessful Substrates for the bioconjugation with CPCs*</u>

During our investigation different cations, peptide and proteins were tested showing no reactivity in the bioconjugation reaction. Figure **S10A** highlights the structure of the cations which were found to be unreactive under standard condition using GTH as model peptide for the bioconjugation (test performed following *general procedure B*). Figure **S10B** show the structure of the peptides and proteins which didn't react in the bioconjugation using CPC **2a** or **2c** as alkylating reagent (test performed following *general procedure B* in the case of peptides).

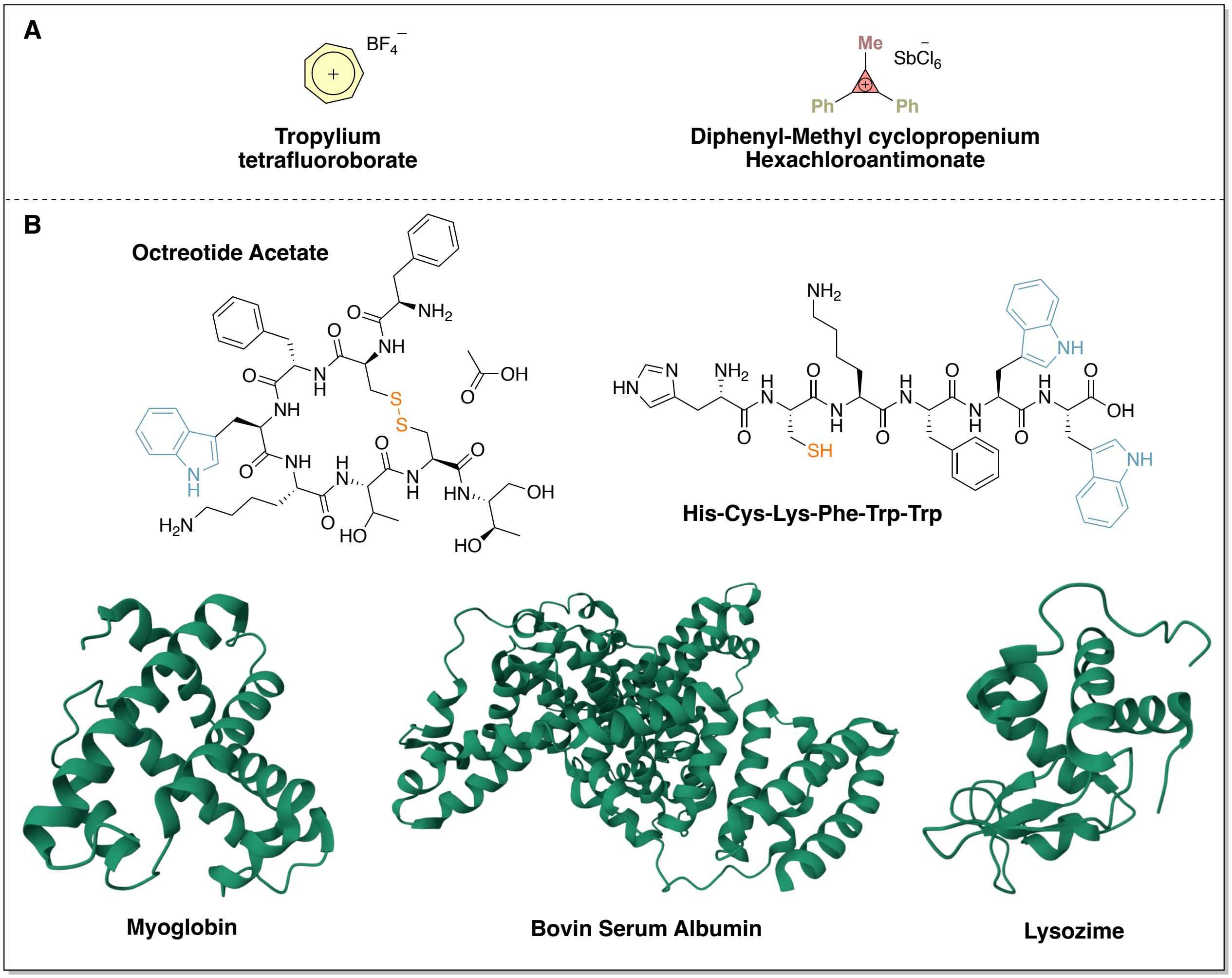

Figure S10. Table of unsuccessful peptides and proteins

The bioconjugation using CPC **2a-c** was shown to be ineffective using tryptophan-containing substrates such as commercially available Octreotide Acetate (Sigma-Aldrich) or Pentapeptide H-

HCKFWW-OH (SPPS). Furthermore, the reaction didn't work on commercially available Myoglobin, Bovin Serum Albumin and Lysozime (Sigma-Aldrich) probably due to inner position of cysteine.

<u>*Unsuccessful reactions for the functionalization of the cyclopropene moiety*</u>

The possibility to use the cyclopropene moiety as bio-orthogonal handle was assessed by testing different well-known metal catalyzed reactions. Figure **S11** schematically highlight our attempts to react the cyclopropene on the bioconjugates.

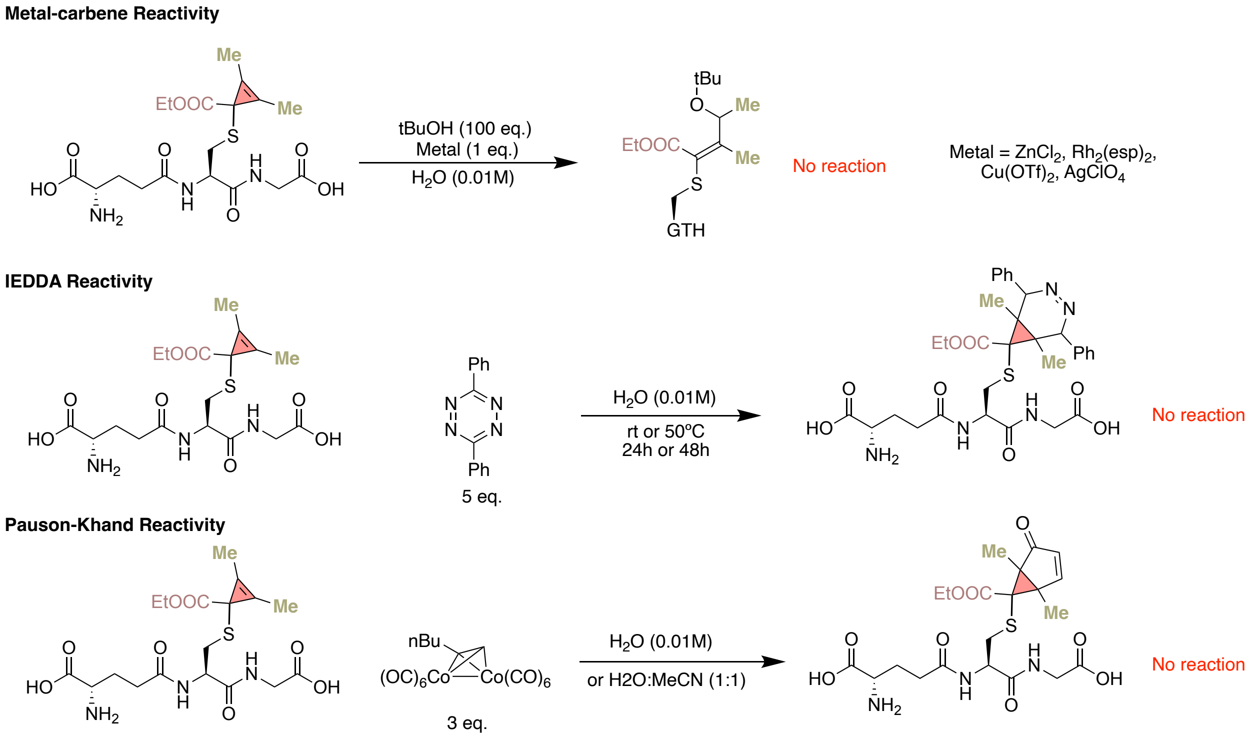

Figure S11. Table of unsuccessful peptides and proteins

10. Oxytocin Selectivity

To prove the selectivity of the cyclopropenylation reaction toward the internal or external cysteine we designed an experiment based on the selective conversion of terminal cysteine to thiazolidine (See figure **S12** Below) reported in literature.[4] If the cyclopropenylation proceed to the internal cysteine, then we should observe its conversion to the thiazolidine derivative upon treating the crude with (2-formylphenyl)boronic acid, on the other hand if the alkylation undergoes to the N-terminal cysteine, no reaction should be observed after treating the product with the same reagent (See figure **S12** below).

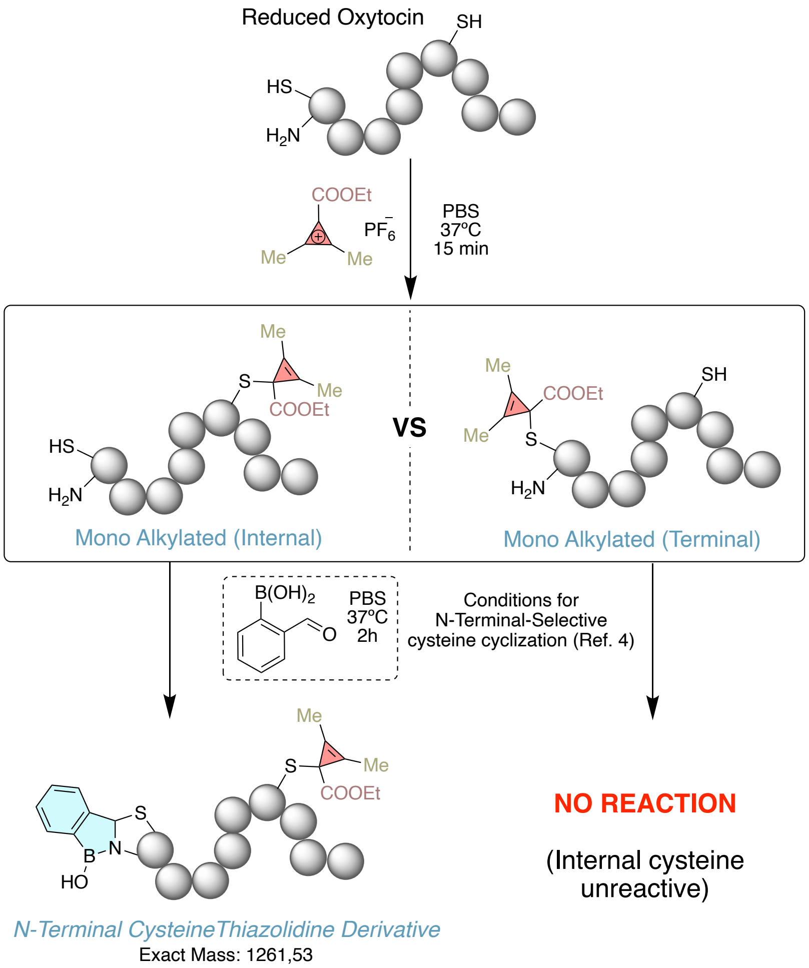

Figure S12. Strategy for the elucidation of the cyclopropenylation's regioselectivity.

After a first functionalization of oxytocin with CPC **2a** (Figure **S13A**), 20 equivalents of (2-formylphenyl)boronic acid were added to the same eppendorf and the reaction was stirred at 37ºC using ThermoMixer C. Analysis of the reaction after 2 hours from the addition of the boronic acid

showed 60% conversion of the monoakylated oxytocin **13** to the expected thiazolidine product (Figure **S13B**).The incomplete conversion of monoakylated oxytocin **13** to the thiazolidine product is due to the innate poor reactivity of the oxytocin substrate toward the (2-formylphenyl)boronic acid reagent. This was corroborated with a control experiment performing the selective N-Terminal selective bioconjugation without previous alkylation with CPC **2a**: even in the presence of 100 eq. of boronic acid reagent the oxytocin is not completely transformed to the thiazolidine product (Figure **S13C**).

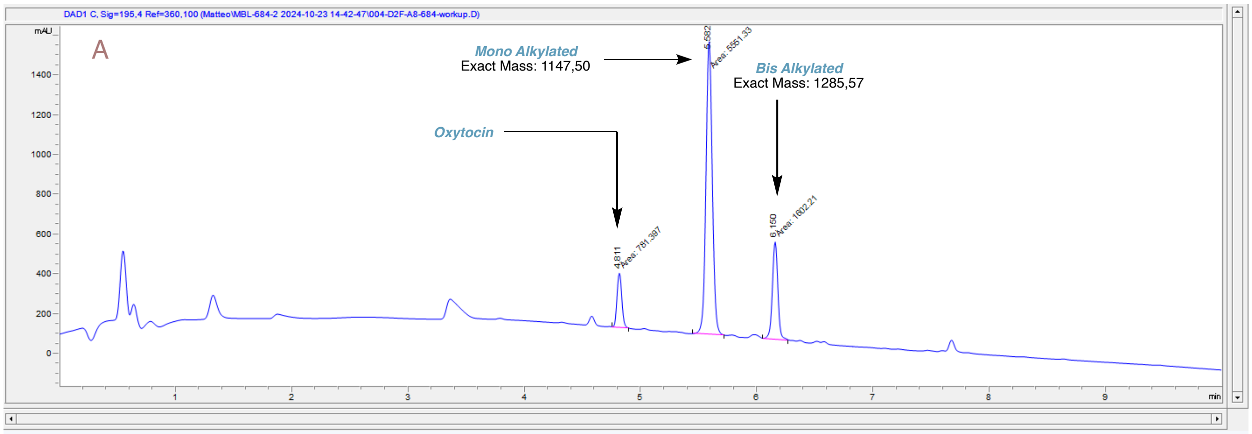

File Information	
LC-File	004-D2F-A8-684-workup.D
File Path	D:\ChemStation\2\Data\Matteo\MBL-684-2 2024-10-23 14-42-47
Date	23-Oct-24, 15:26:10
Sample	684-workup
Sample Info	

#	Time	Type	Area	Height	Width	Area%	Symmetry
1	4.811	MM	781.4	273.2	0.0477	9.848	0.921
2	5.582	MM	5551.3	1476	0.0627	69.961	0.945
3	6.15	MM	1602.2	490.3	0.0545	20.192	0.987

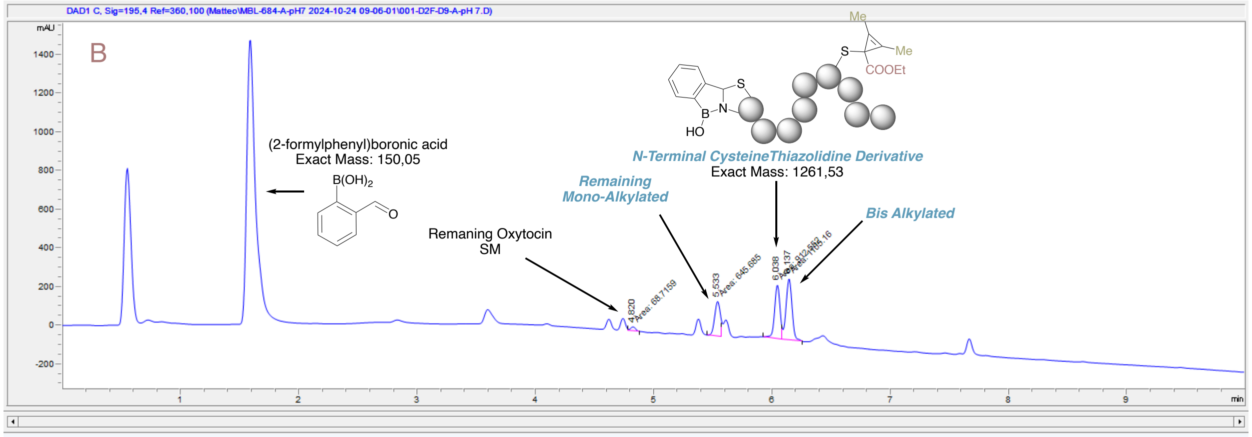

File Information	
LC-File	001-D2F-D9-A-pH 7.D
File Path	D:\ChemStation\2\Data\Matteo\MBL-684-A-pH7 2024-10-24 09-06-
Date	24-Oct-24, 09:10:45
Sample	A-pH 7
Sample Info	

#	Time	Type	Area	Height	Width	Area%	Symmetry
1	4.82	FM	68.7	22.2	0.0515	2.515	0.799
2	5.533	MF	645.7	180.7	0.0596	23.633	0.955
3	6.038	MF	912.6	279	0.0545	33.401	0.991
4	6.137	FM	1105.2	318.3	0.0579	40.451	0.938

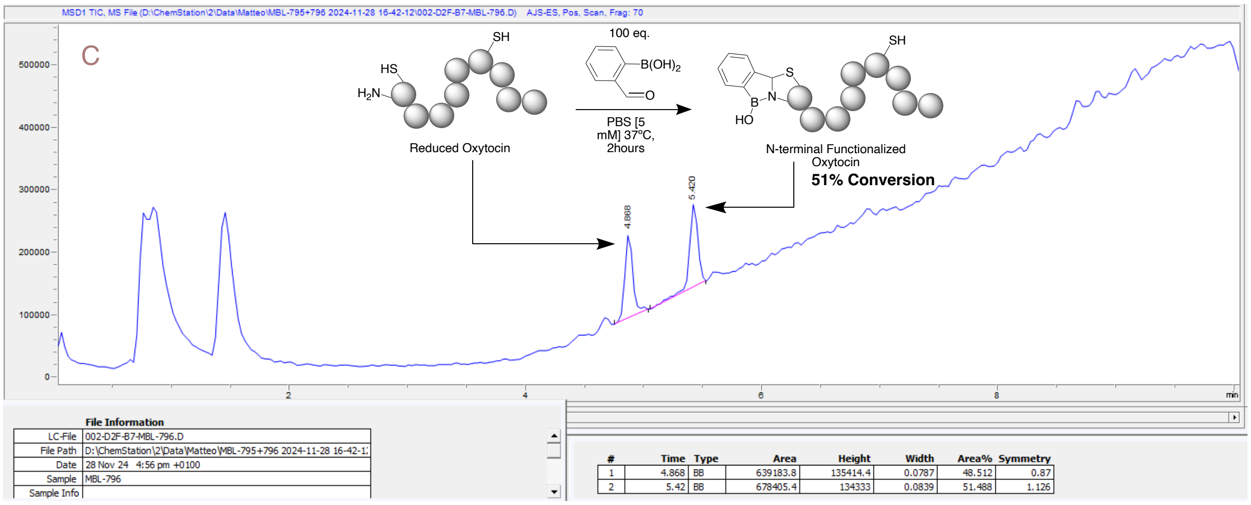

Figure S13. Chromatograms of the experiment regarding the selectivity of the cyclopropenylation reaction: A) Chromatogram of the reaction after the cyclopropenylation B) Chromatogram of the reaction after a second bioconjugation selective for the N-Terminal Cysteine residue. C) Control experiment showing the poor reactivity of Oxytocin toward the boronic acid reagent.

11. Decaging of bioconjugate & bio-orthogonal functionalization with NMM

Decaging of Bioconjugate

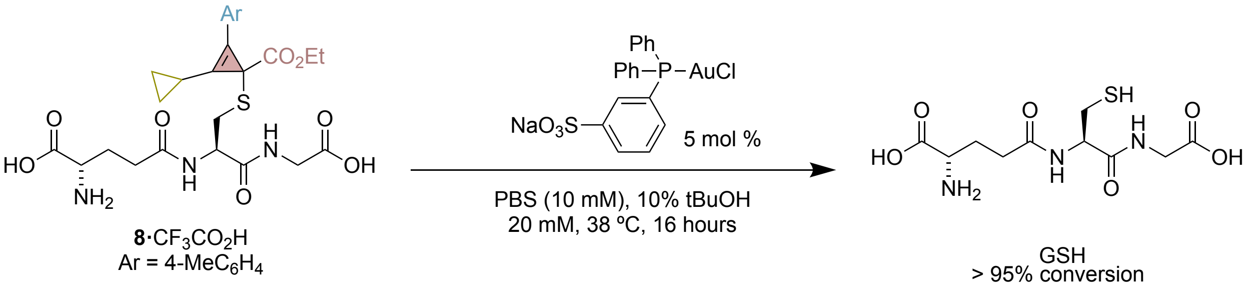

In an Eppendorf tube was prepared a 20 mM solution of bioconjugate **8** (0.02 mmol, 13 mg) in 1mL of PBS (10 mM, pH 7.4) and tBuOH (10%). In another Eppendorf a 0.1M stock solution of gold complex[5] (30 mg in 500 μL) in PBS (10mM, pH 7.4) was prepared. Finally, 1μL of gold complex stock solution was added to the solution of **8**. The mixture was stirred at 38ºC for 16 hours using Eppendorf thermomixer. The aqueous phase was filtered and analyzed by reverse phase UHPLC-MS using column Zorbax 300 SB-C8 over 10 minutes (*method 5* in ***section 2***). Analysis revealed 37% conversion after 5 hours and >95% conversion after 16 hours.

Analysis after 5 hours

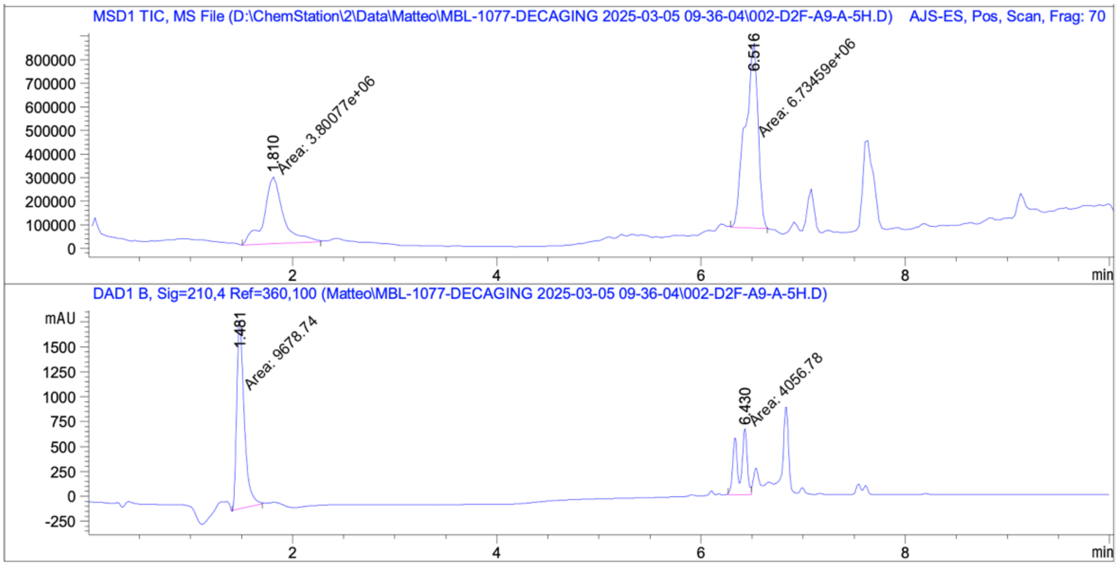

Analysis after 18 hours

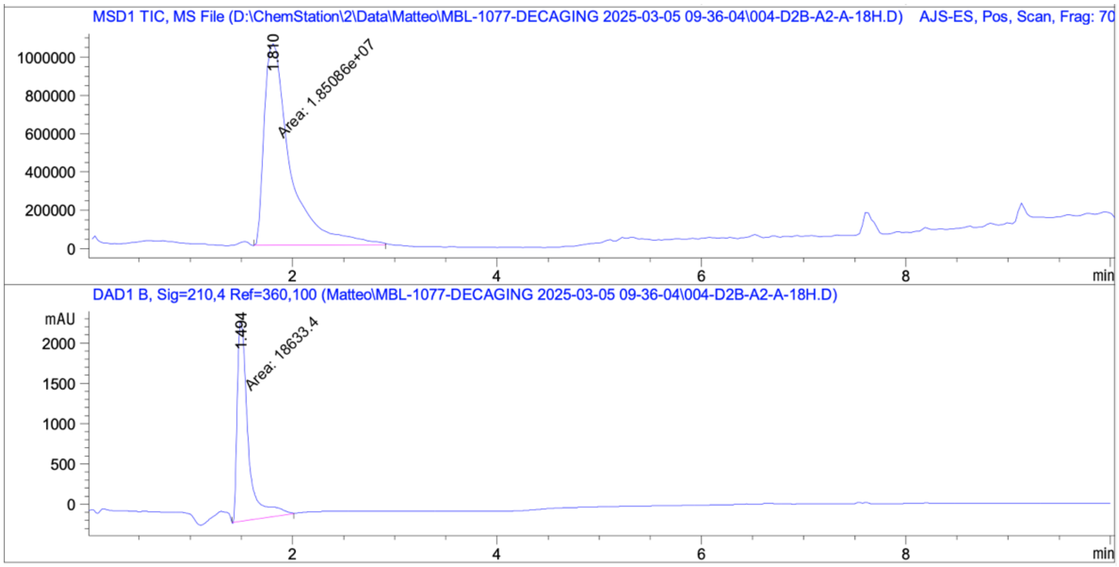

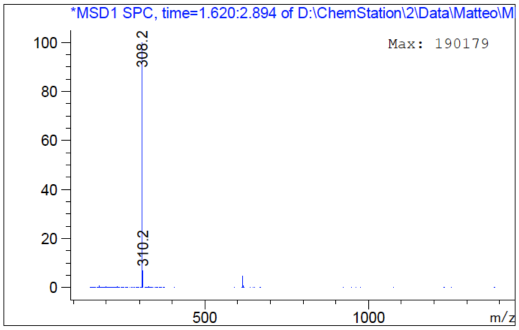

Peak #1 at 1.810 min (1.625 to 2.908 min)

Bio-orthogonal functionalization with NMM

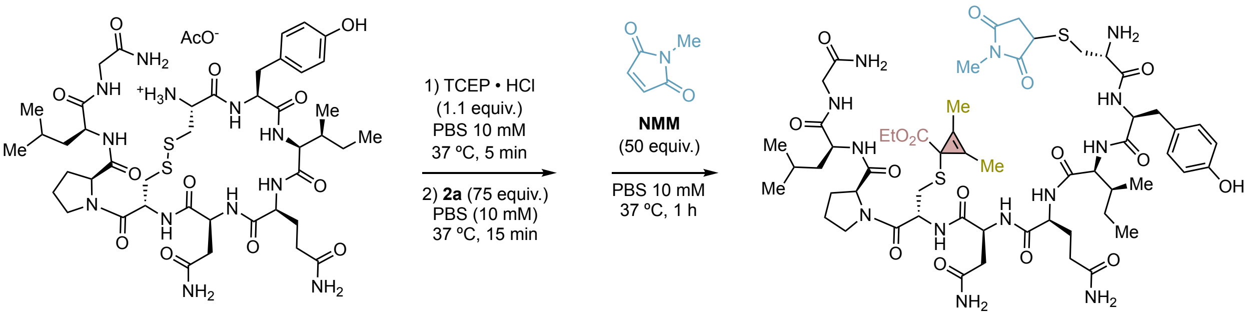

Oxytocin Acetate **16** 61%, dr 1:1

In an Eppendorf tube was prepared a 1.25 mM solution of Oxytocin Acetate in PBS (10 mM, pH 7.4). To this solution 11 µL of TCEP 10 mM stock solution in H_2O were added, and the mixture stirred at 37ºC. After 10 minutes, CpC **2a** was added neat (2.1 mg 75 eq.) to the solution, and the mixture was kept 15 minutes under stirring at 37ºC before adding 10 µL of N-Methyl Maleimide 0.5 M stock solution in MeCN. After 1 hour, the excess of organic material was extracted from the aqueous solution using Et2O (2x200 µL). The mixture was then filtered and analyzed by reverse phase UHPLC-MS (*method 2* in ***section 2***).

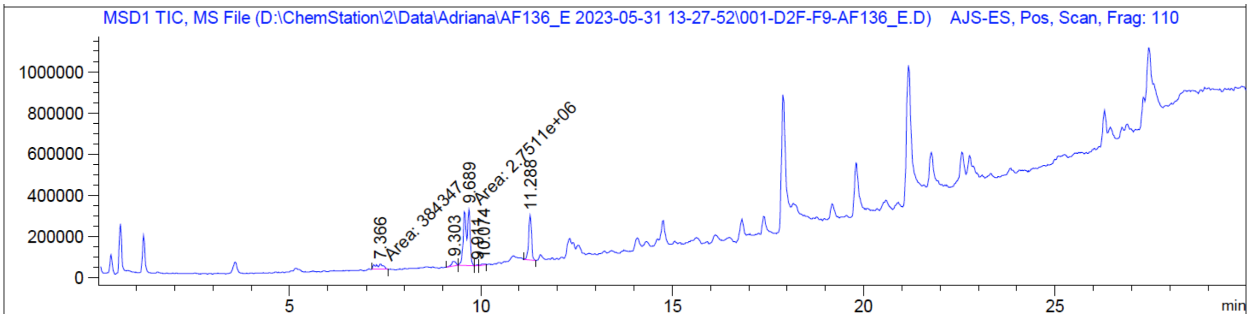

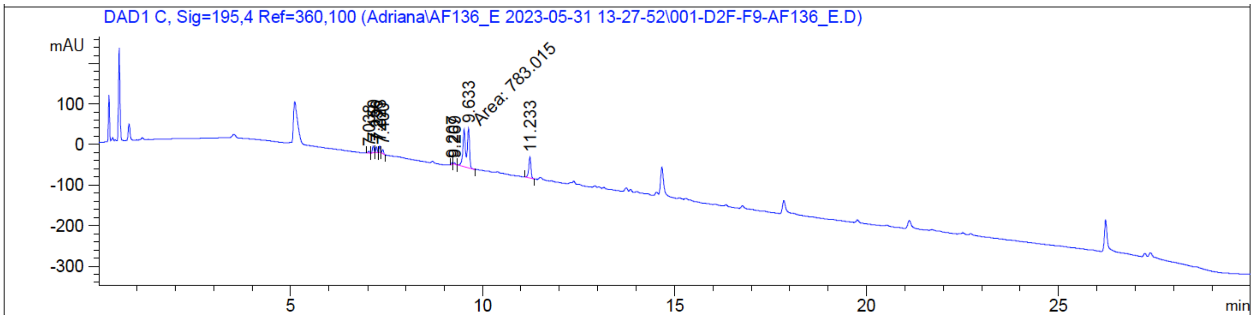

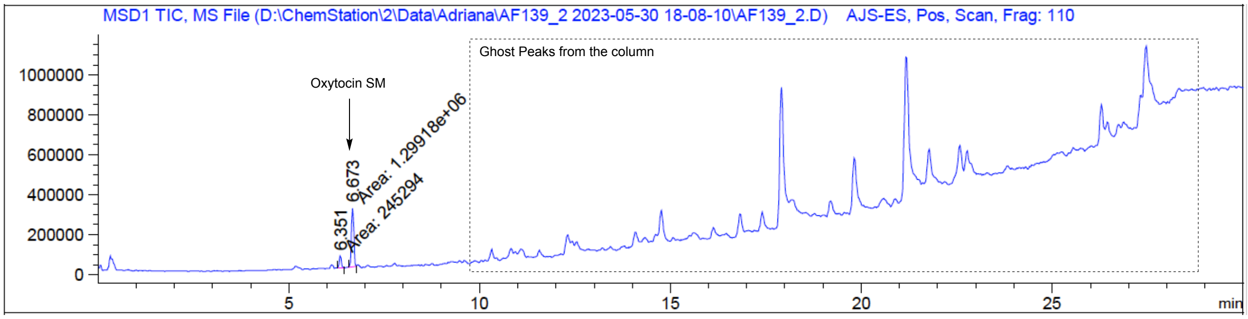

ZOOM & Description

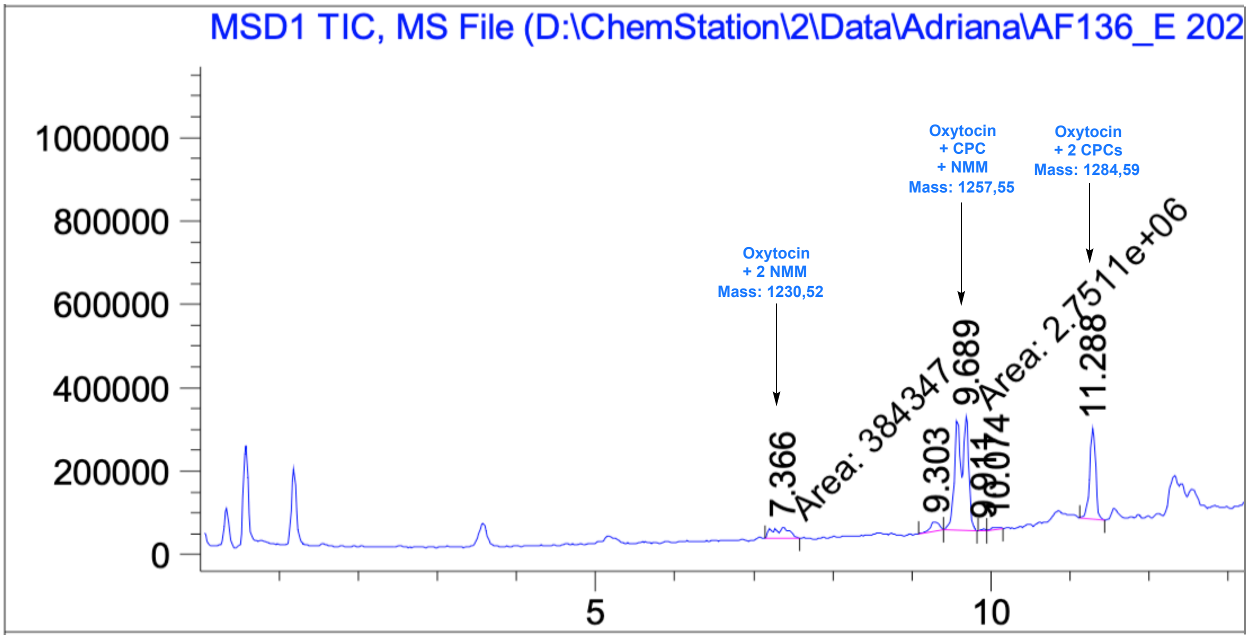

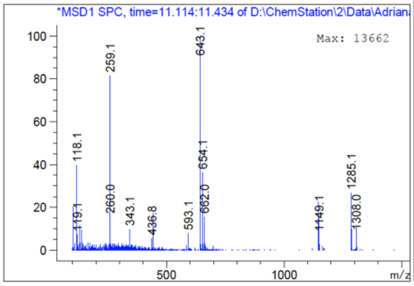

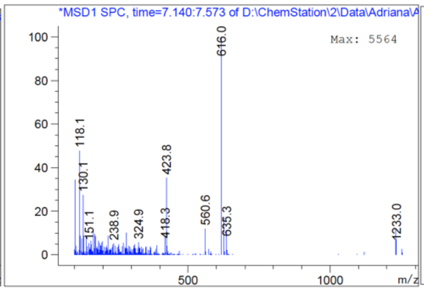

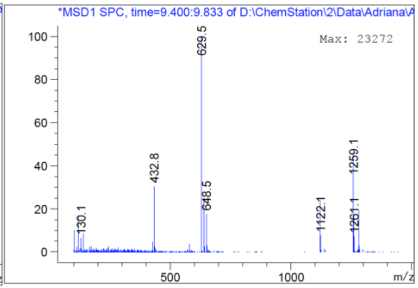

Peak #6 at 11.288 min (11.117 to 11.434 min)   Peak #1 at 7.366 min (7.140 to 7.575 min)   Peak #3 at 9.689 min (9.403 to 9.824 min)

12. Stability of Bioconjugates

The stability of bioconjugate **6** was tested with the following stability test: two solutions of bioconjugate were prepared [0.01M] in H_2O. The pH of the two solutions was corrected to pH 2 and pH 10 using 1M solution of HCl and NaOH respectively. After 12 hours, 5 days and 3 weeks

an aliquot of each solution was taken and diluted for the HPLC analysis. The chromatograms below show that the biconjugate is stable at room temperature up to 3 weeks.

	pH: 2 (HCl corrected)	pH: 10 (NaOH corrected)
12 hours	Stable (Chromatogram A)	Stable (Chromatogram B)
5 days	Stable (Chromatogram C)	Stable (Chromatogram D)
3 weeks	Stable (Chromatogram E)	Stable (Chromatogram F)

Chromatogram A (12 hours pH 2)

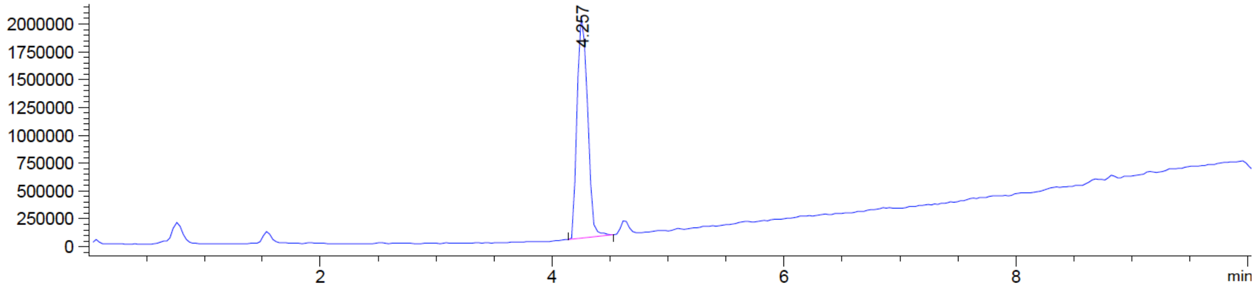

Chromatogram B (12 hours pH 10)

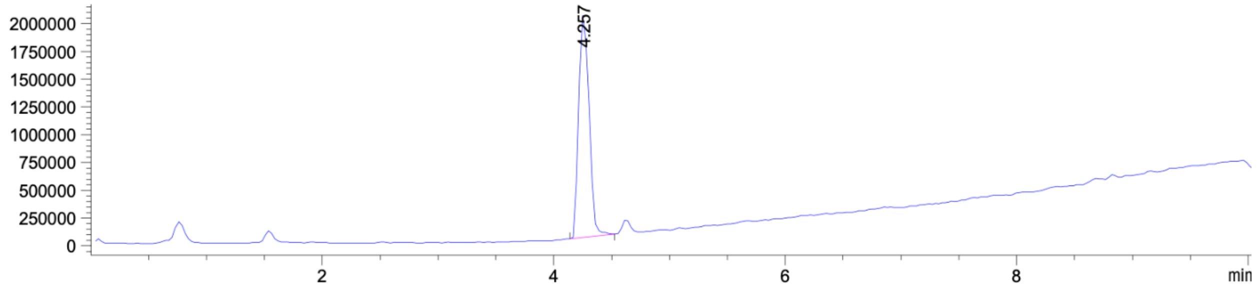

Chromatogram C (5 days pH 2)

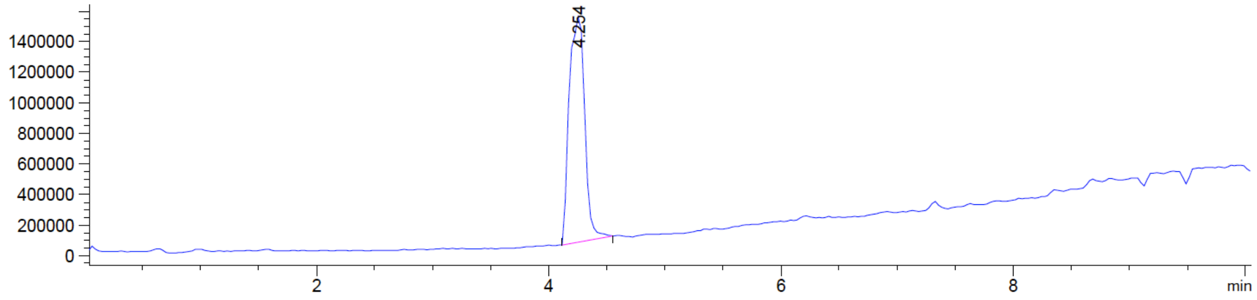

Chromatogram D (5 days pH 10)

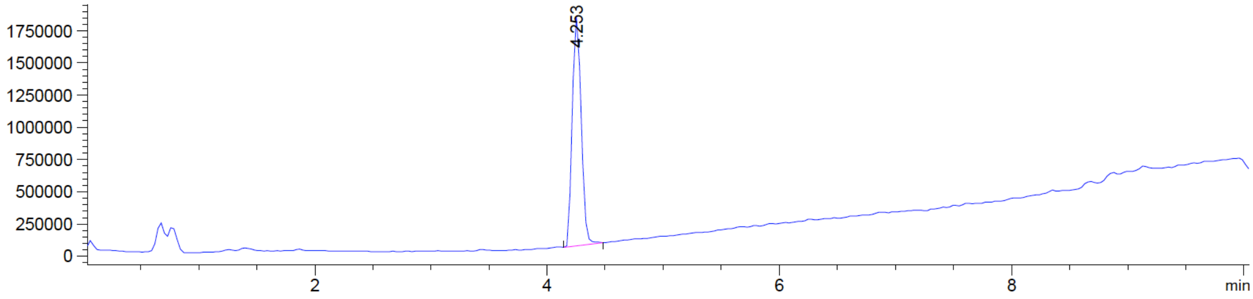

Chromatogram E (3 weeks pH 2)

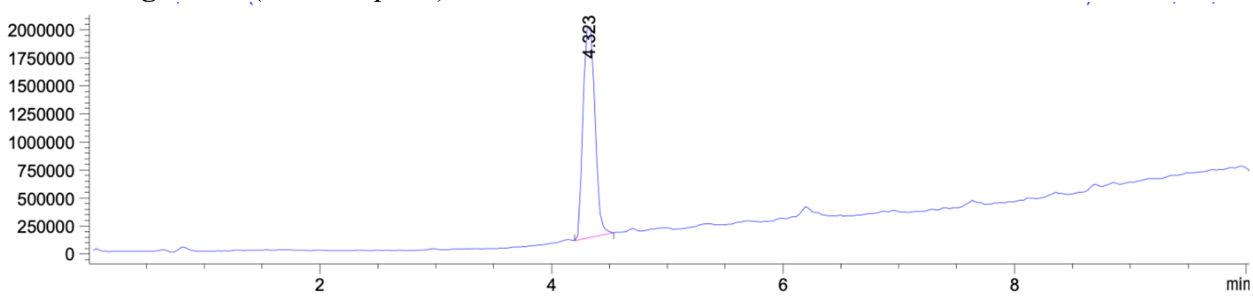

Chromatogram F (3 weeks pH 10)

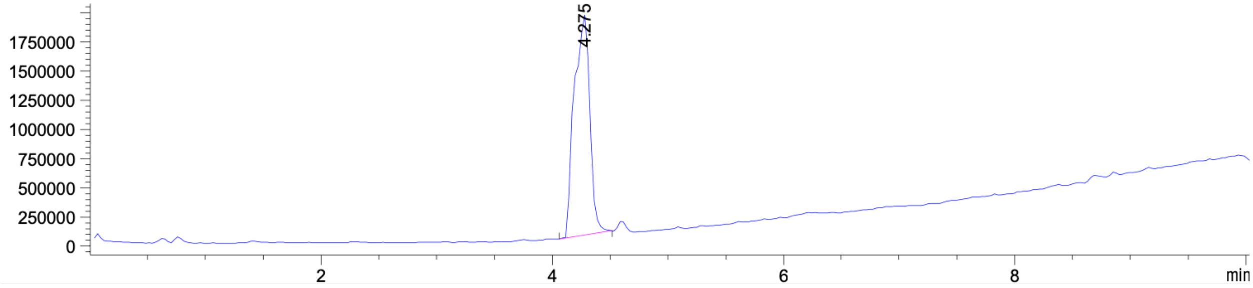

^{1}H-NMR bioconjugate 6 (3 weeks pH 10)

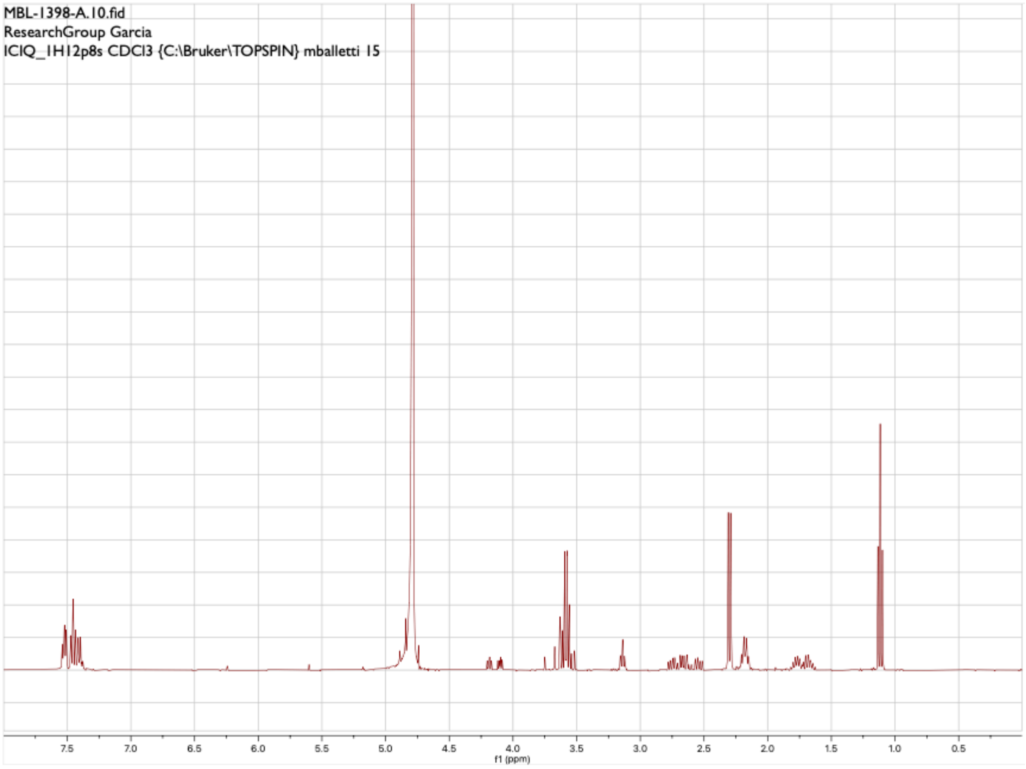

^{1}H-NMR bioconjugate 6 (3 weeks pH 2)

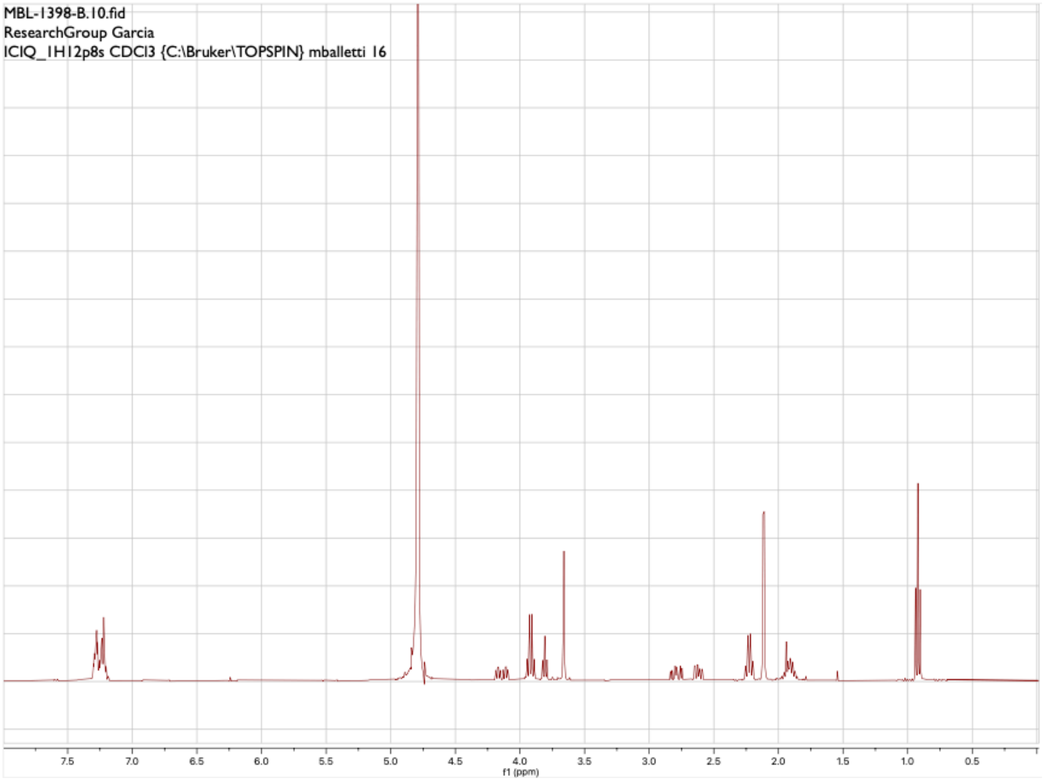

13. References

1 – Tu, H.; Jeandin, A.; Suero, M. G. *J. Am. Chem. Soc.* **2022**, *144*, 16737–16743.

2 – Kanai, M. J. *Am. Chem. Soc.* **2021**, *143*, 19844–19855

3 – Lee, B.; Sun, S.; Jiménez-Moreno, E.; Neves, A. A.; Bernardes, G. J. L. Site-selective installation of an electrophilic handle on proteins for bioconjugation. *Bioorg. & Med. Chem.* **2018**, *26*, 3060–3064.

4 – (a) Bandyopadhyay, A.; Cambraya, S.; Gao, J. *Chem. Sci.* **2016**, *7*, 4589–4593. (b) Faustino, H.; Silva, M. J. S. A.; Veiros, L. F.; Bernardes, G. J. L.; Gois; P. M. P. *Chem. Sci.*, **2016**, *7*, 5052–5058.

5 – Vidal, C.; Tomás-Gamasa, M.; Destito, P.; López, F.; Mascareñas, J. L. Concurrent and Orthogonal Gold(I) and Ruthenium(II) Catalysis inside Living Cells. *Nat. Commun.* **2018**, *9*, 1913

14. Copies of NMR spectra.

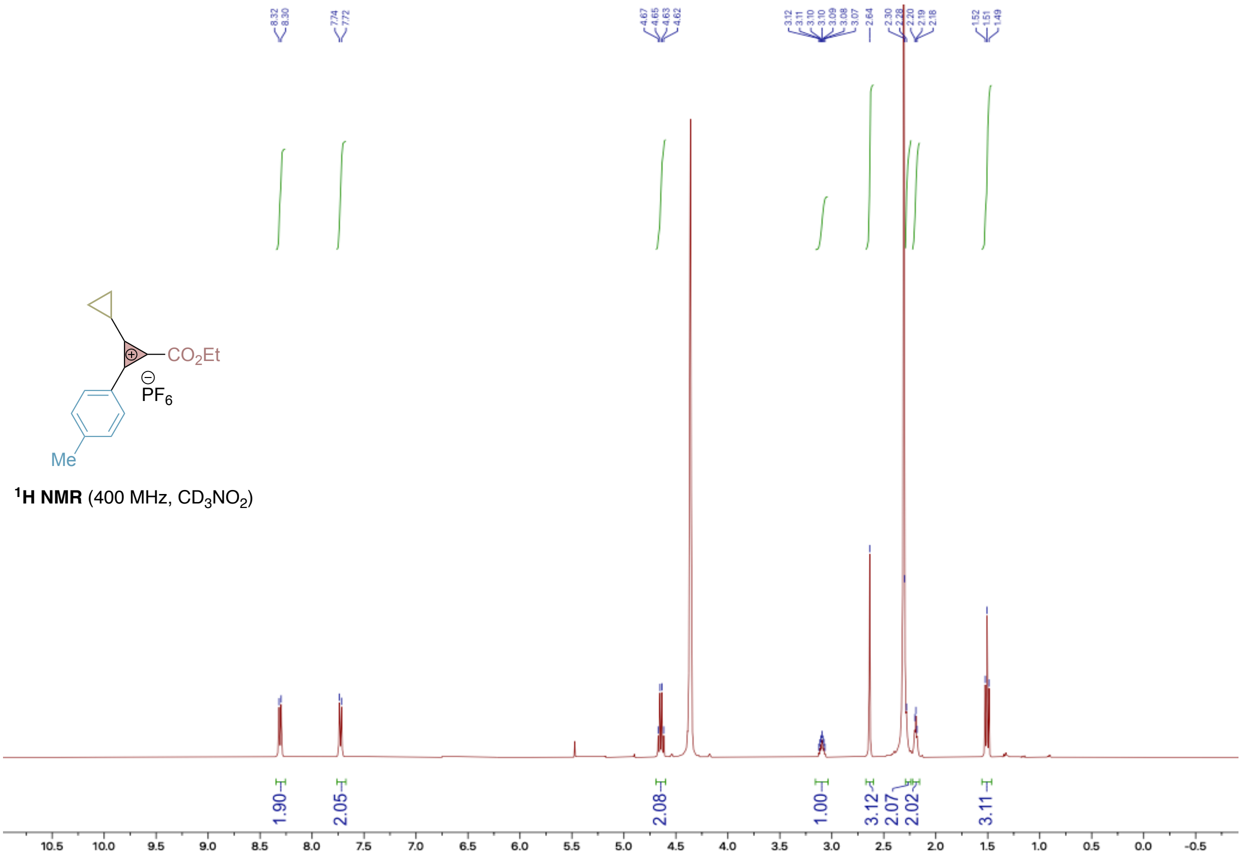

^{1}**H NMR** (400 MHz, CD_3NO_2)

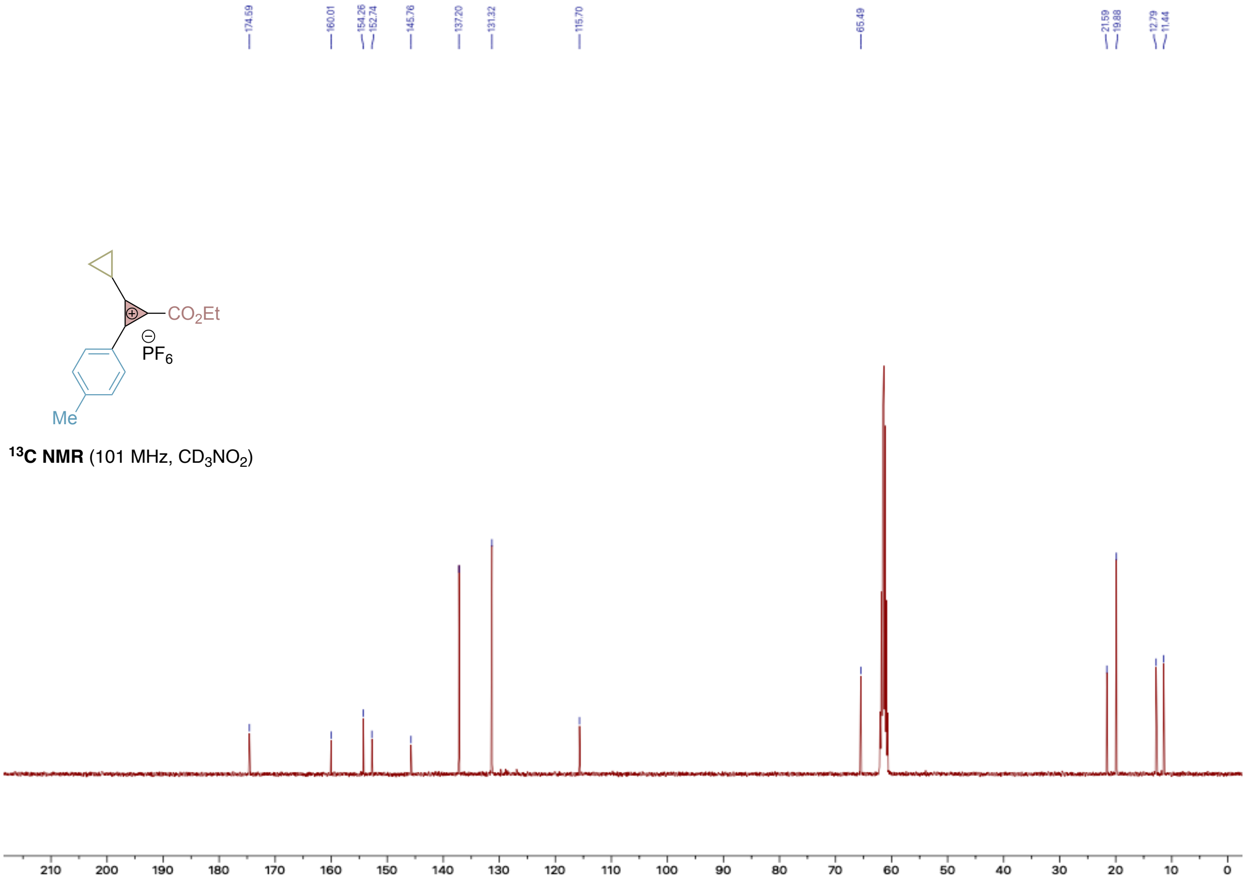

^{13}**C NMR** (101 MHz, CD_3NO_2)

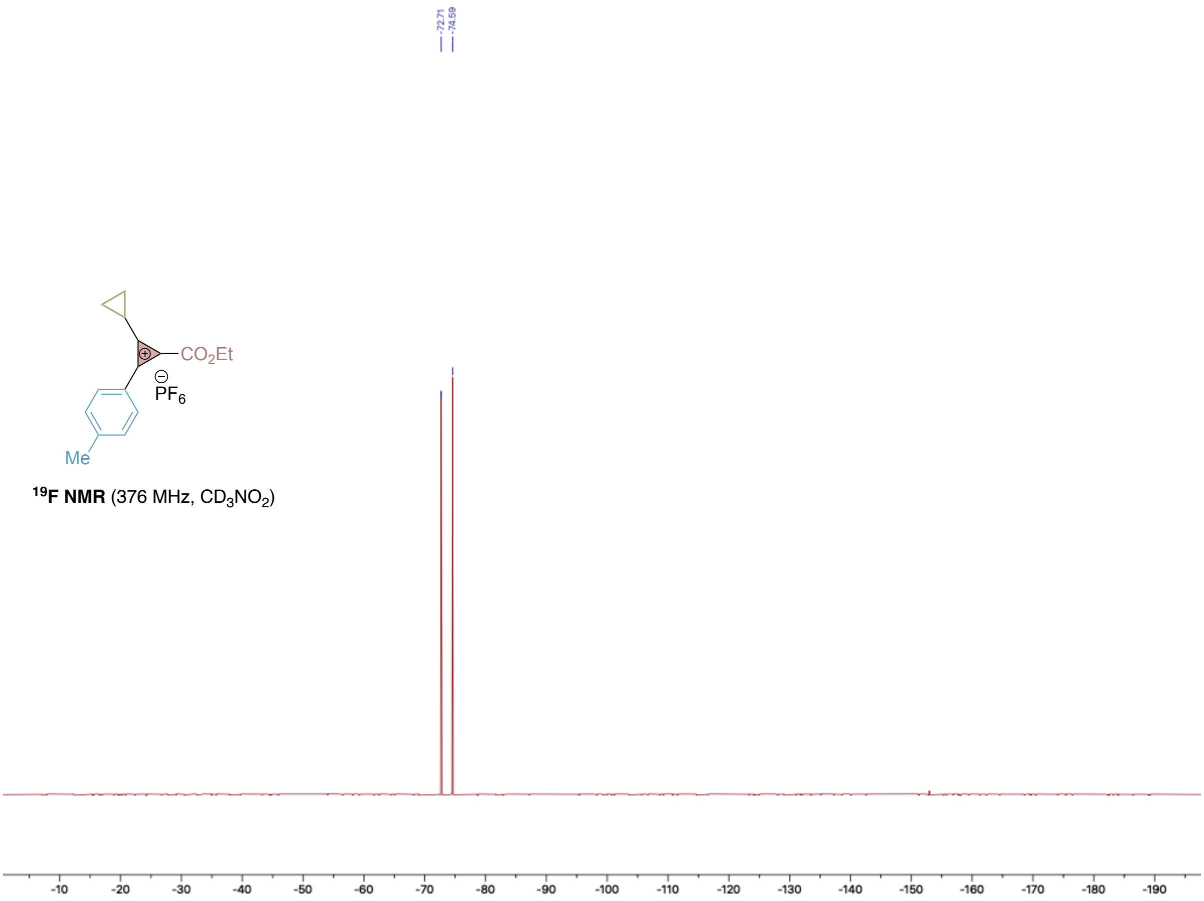
-72.71
-74.59
CO2Et
PF6
Me
19F NMR (376 MHz, CD3NO2)
-10
-20
-30
-40
-50
-60
-70
-80
-90
-100
-110
-120
-130
-140
-150
-160
-170
-180
-190

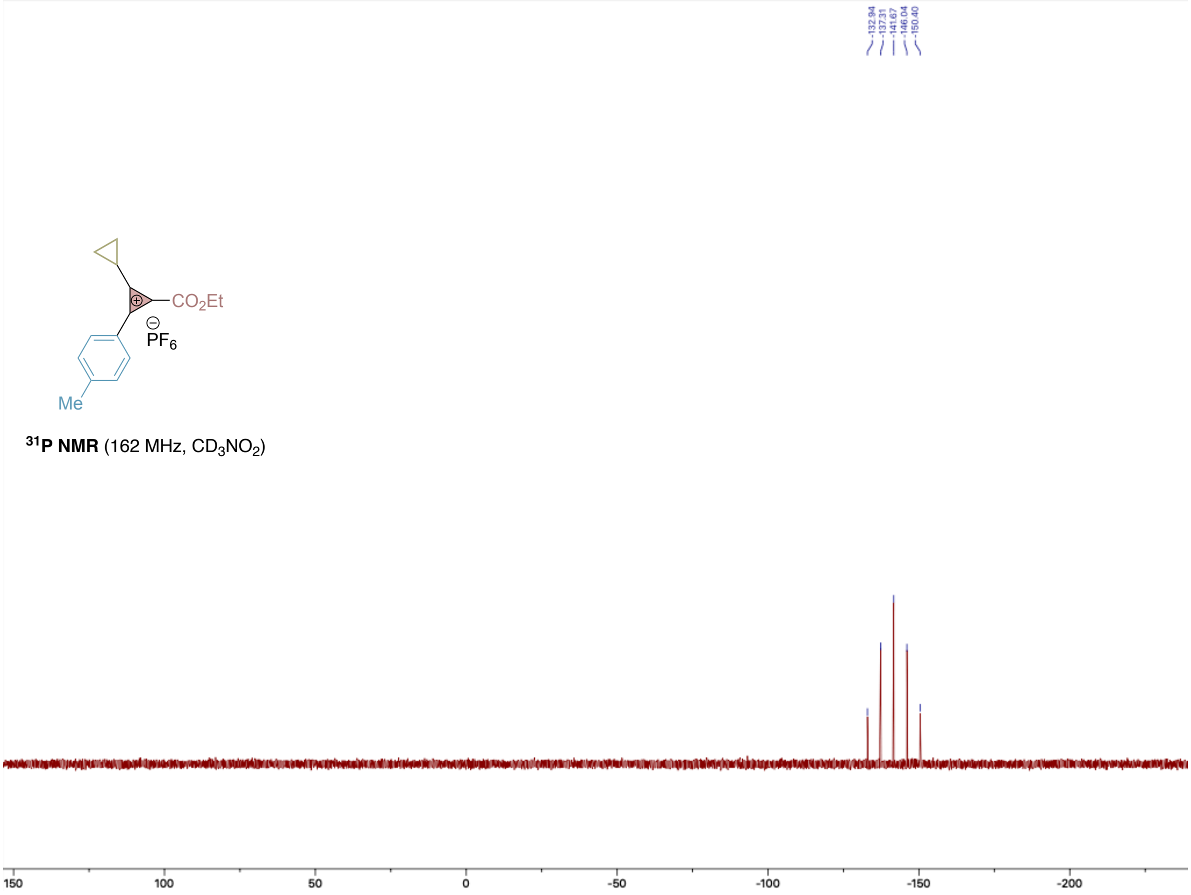
-132.94
-137.31
-141.67
-146.04
-150.40
CO2Et
PF6
Me
31P NMR (162 MHz, CD3NO2)
150
100
50
0
-50
-100
-150
-200

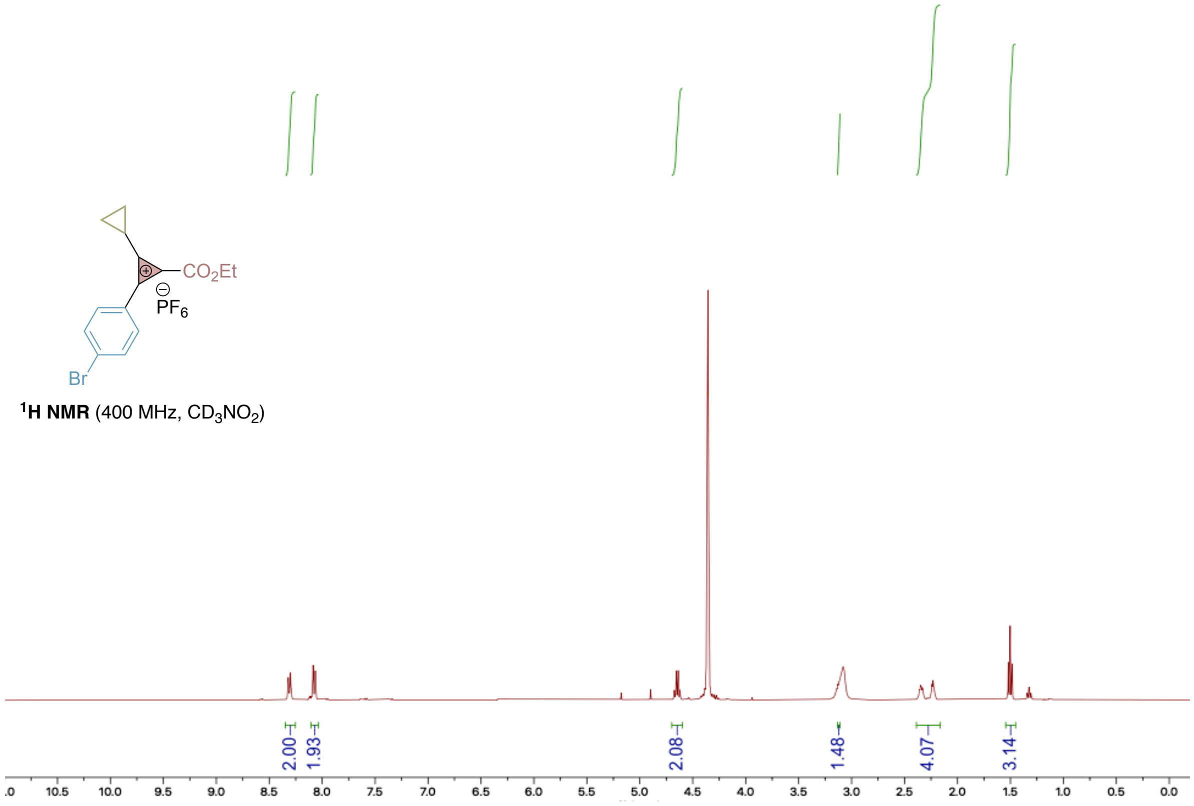

1H NMR (400 MHz, CD_3NO_2)

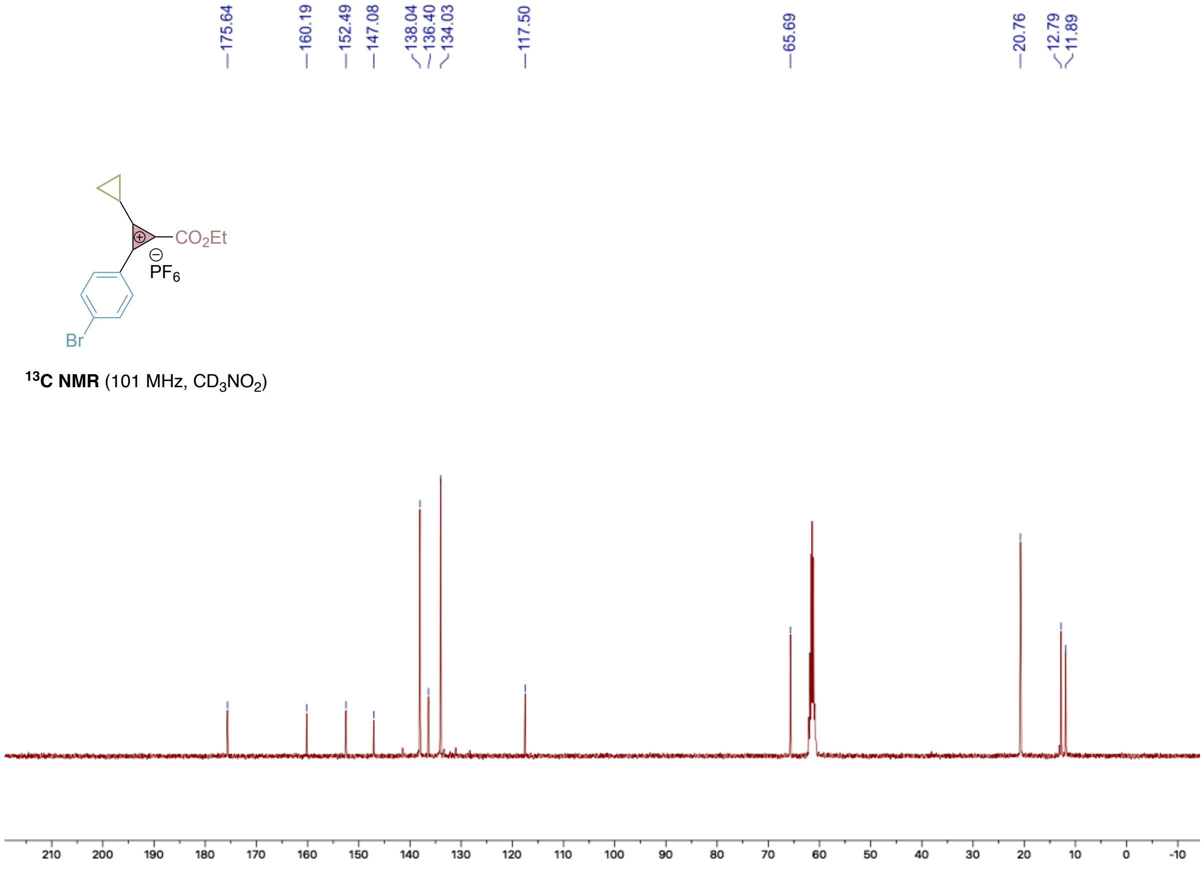

13C NMR (101 MHz, CD_3NO_2)

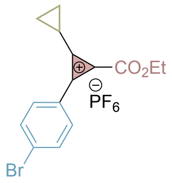

^{19}F NMR (376 MHz, CD_3NO_2)

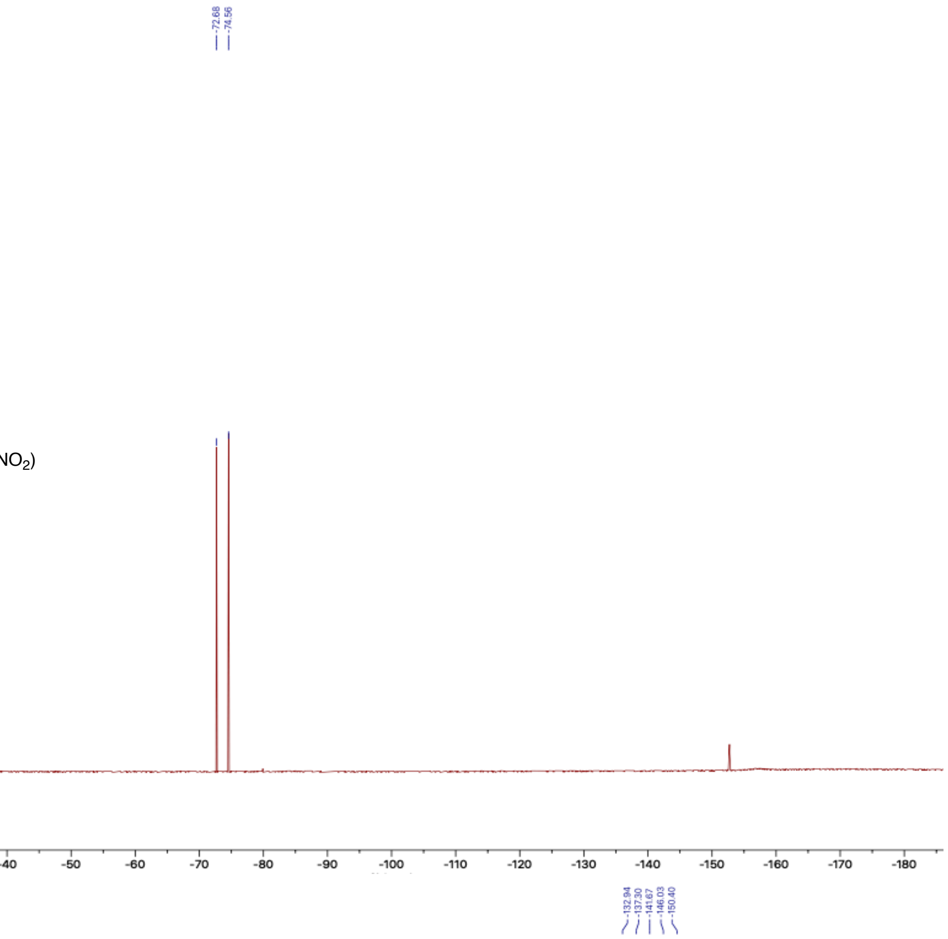

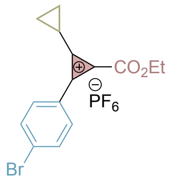

^{31}P NMR (162 MHz, CD_3NO_2)

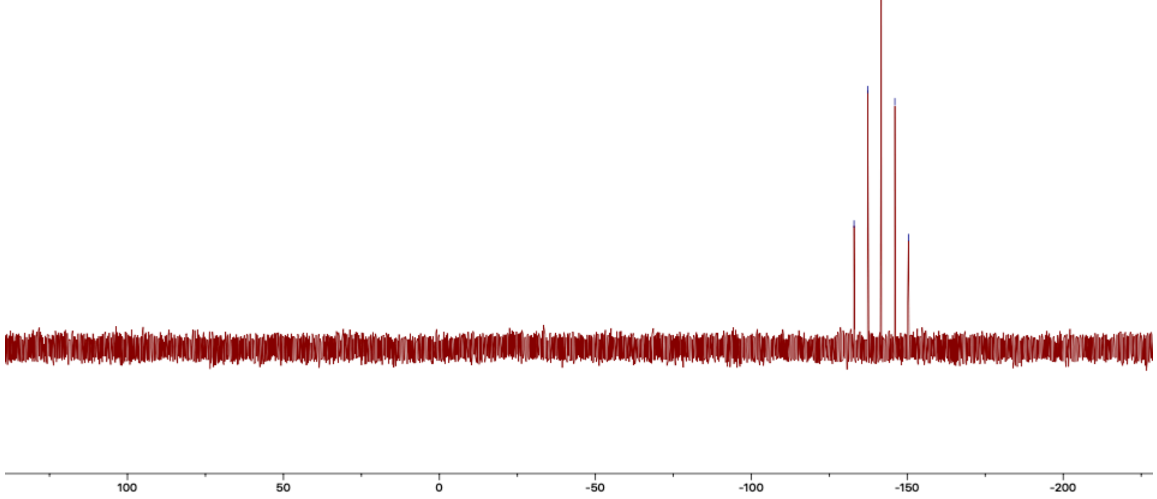

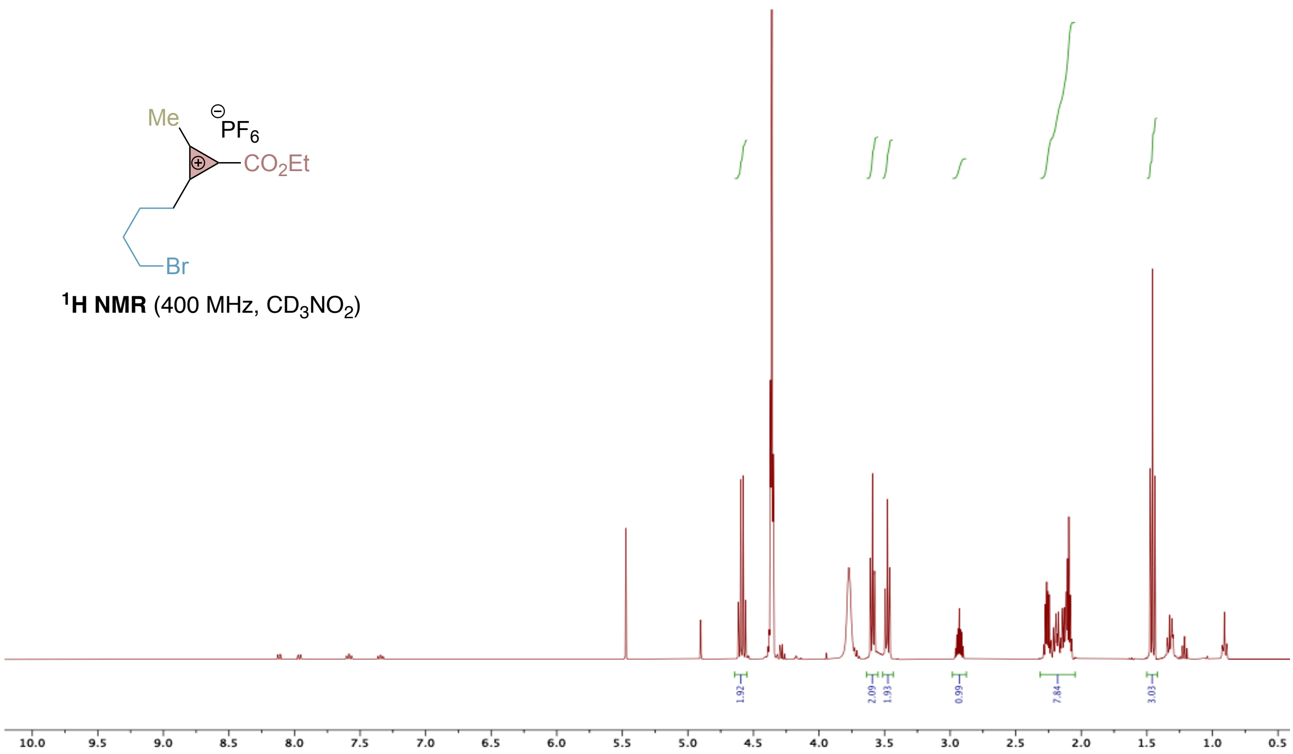
Me
PF6
CO2Et
Br
1H NMR (400 MHz, CD3NO2)
1.92
2.09
1.93
0.99
7.84
3.03

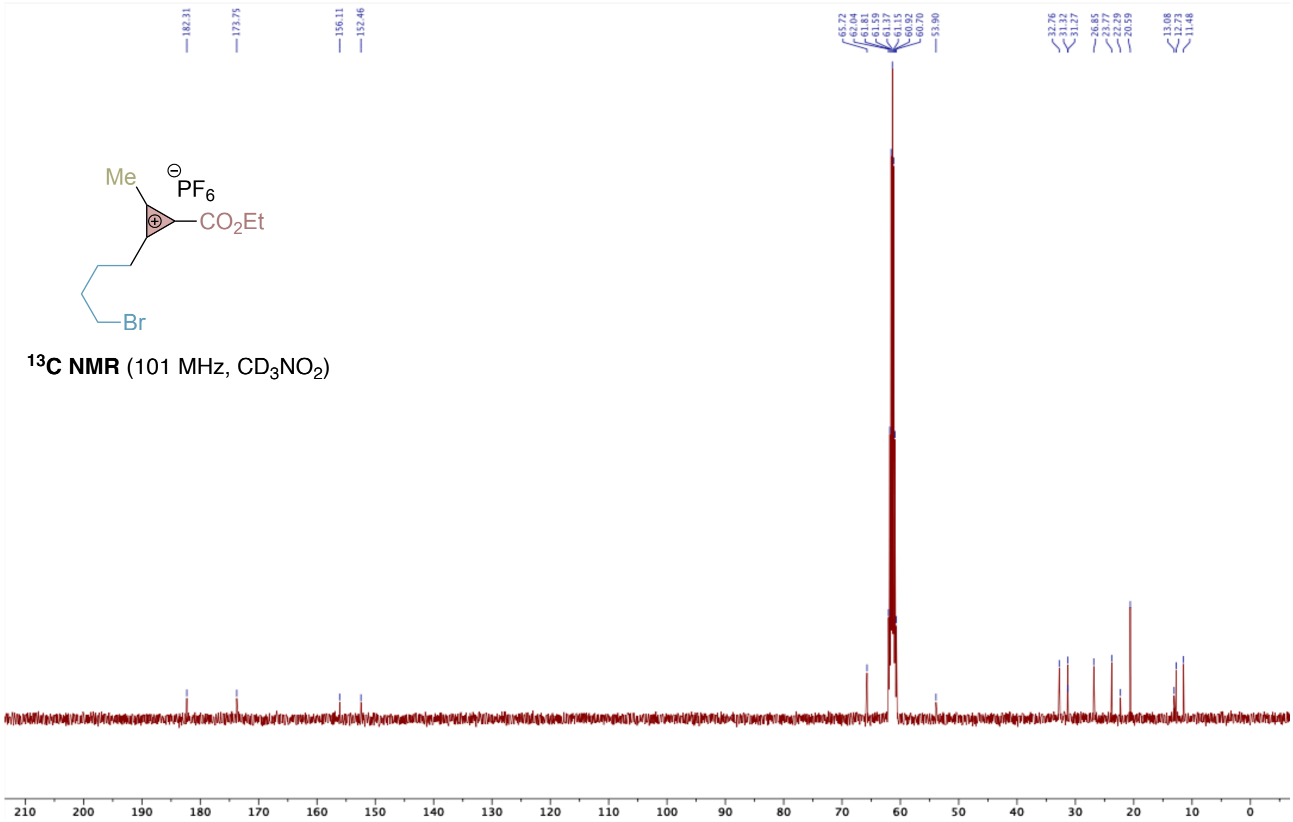
182.31
173.75
156.11
152.46
65.72
62.04
61.81
61.59
61.37
61.15
60.92
60.70
53.90
32.76
31.32
31.27
26.85
23.77
22.29
20.59
13.08
12.73
11.48
Me
PF6
CO2Et
Br
13C NMR (101 MHz, CD3NO2)

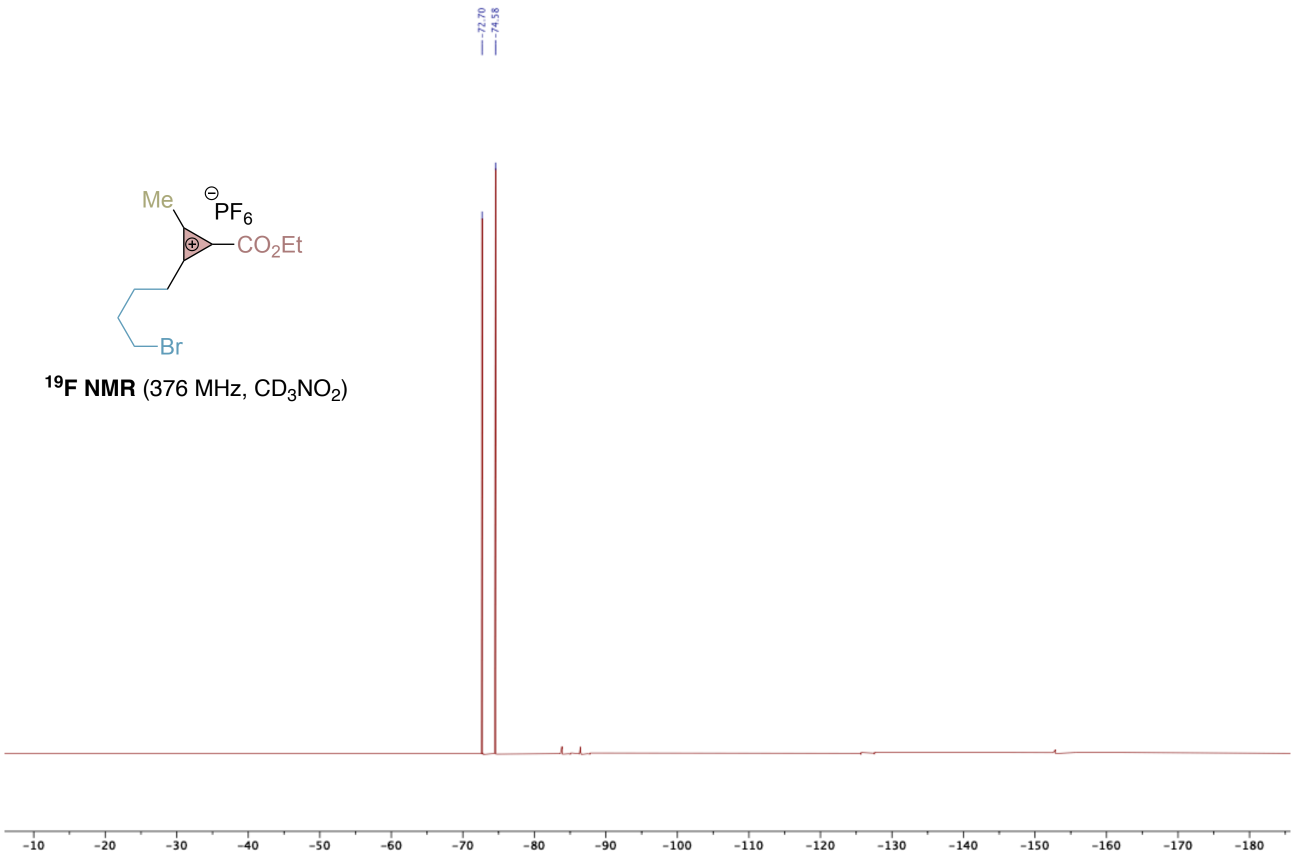

^{19}**F NMR** (376 MHz, CD_3NO_2)

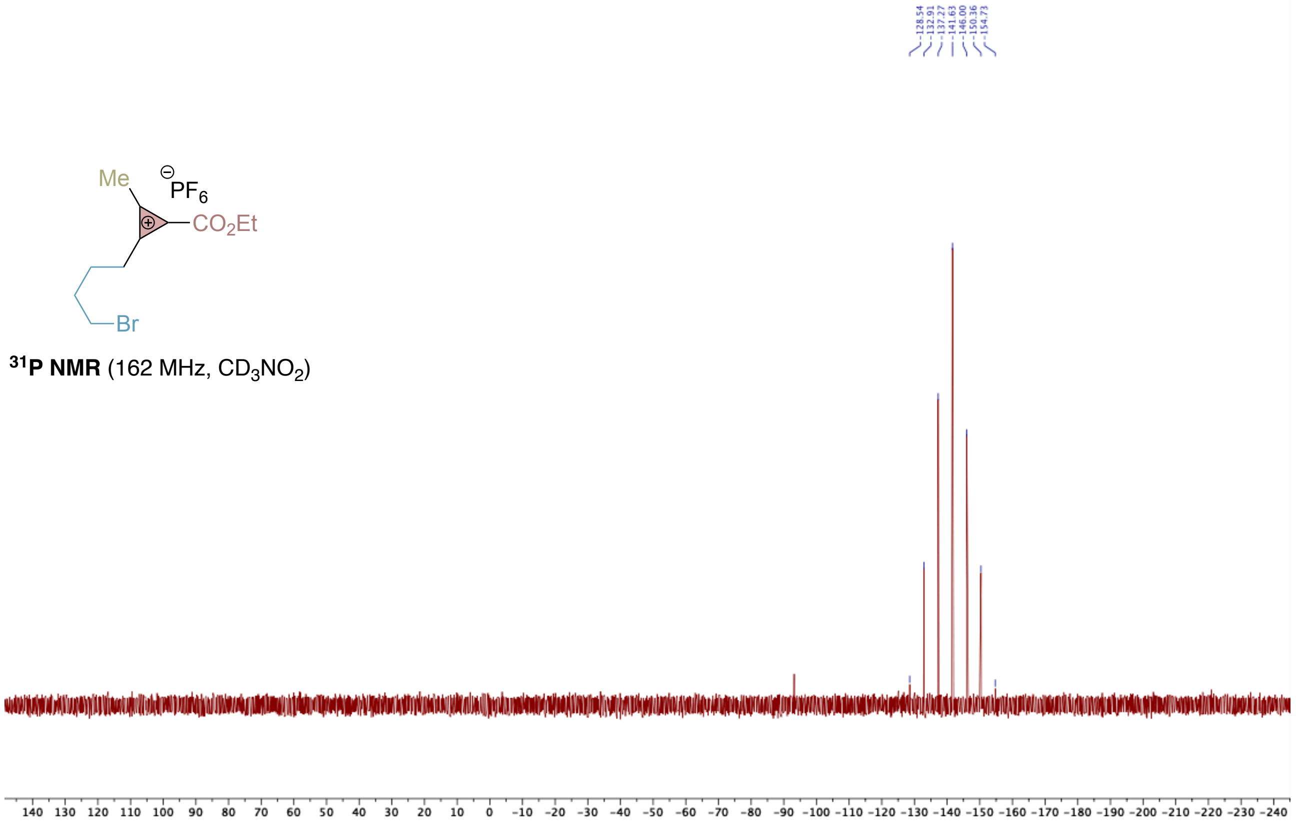

^{31}**P NMR** (162 MHz, CD_3NO_2)

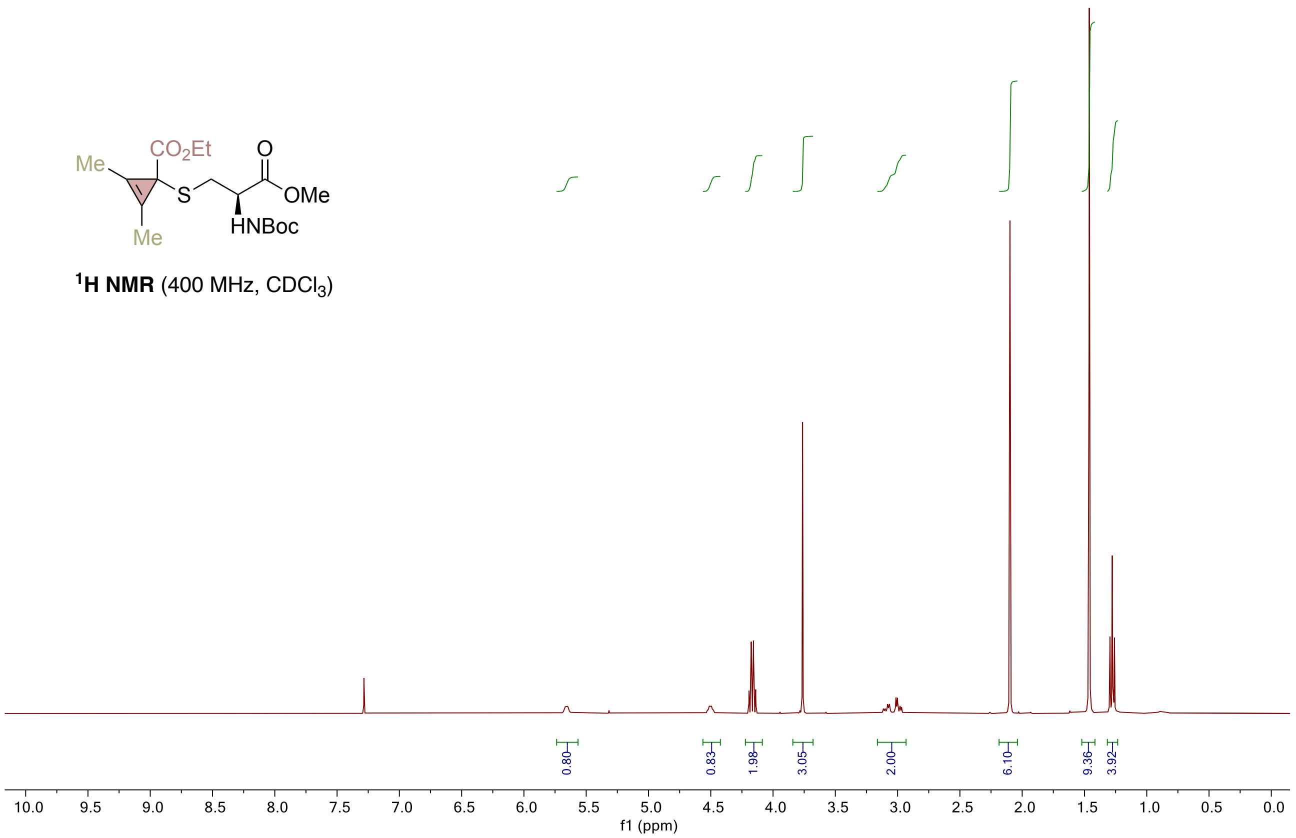

^{1}H NMR (400 MHz, $CDCl_3$)

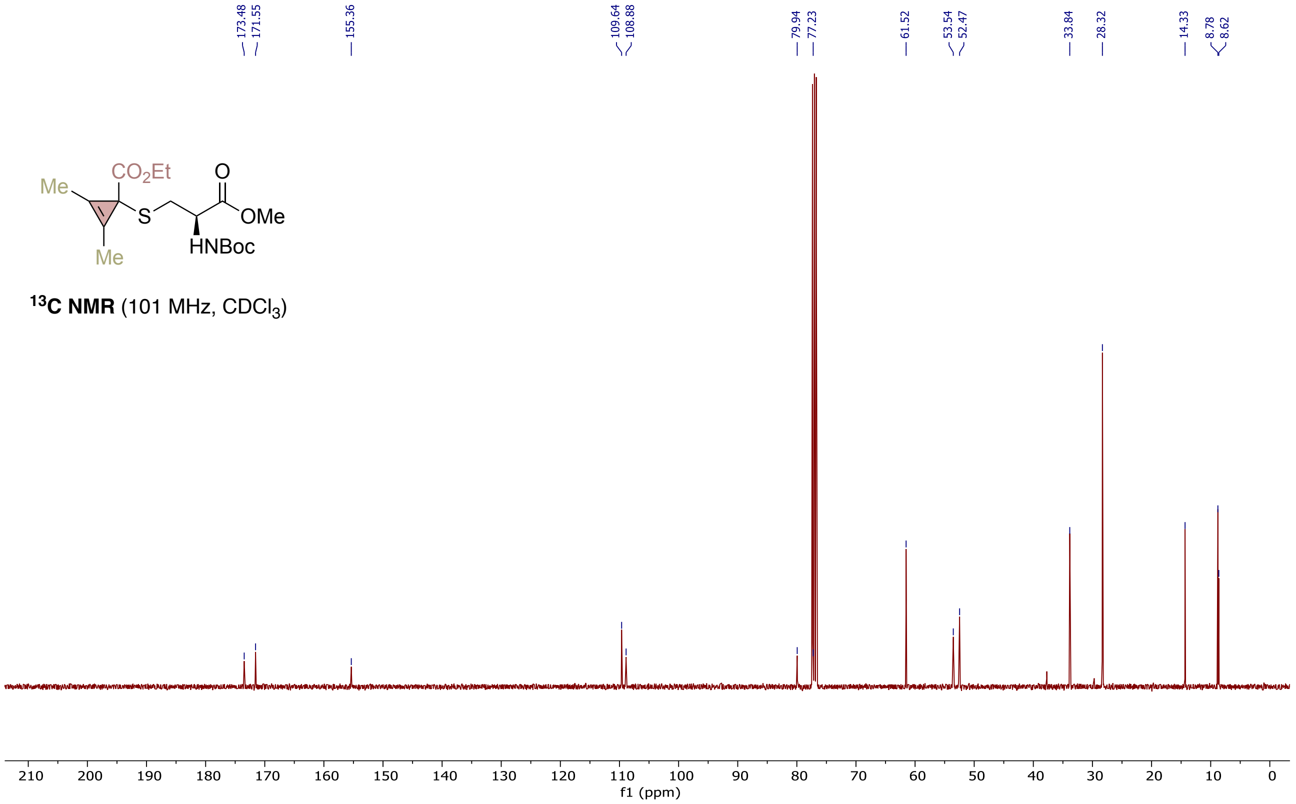

^{13}C NMR (101 MHz, $CDCl_3$)

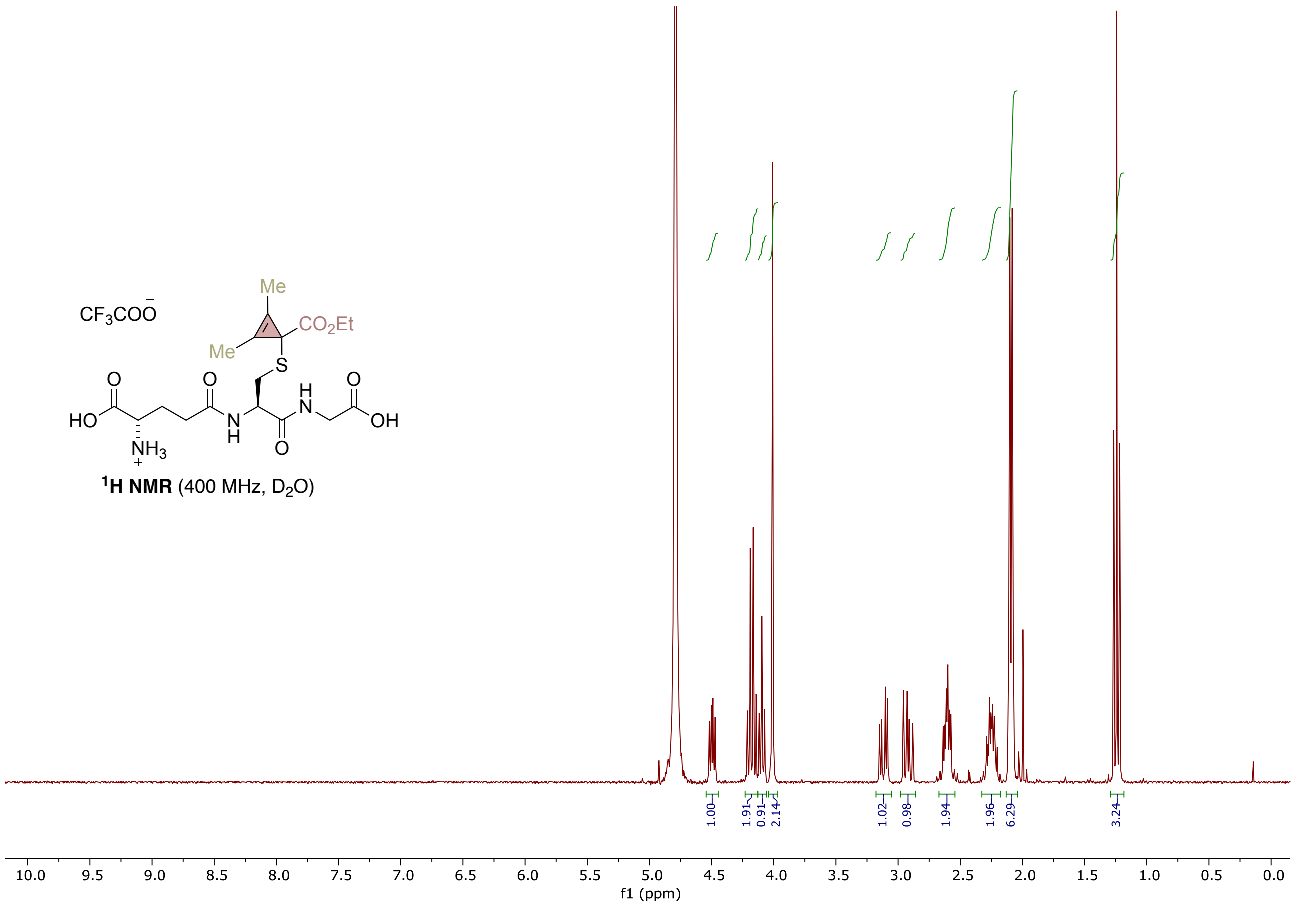

CF3COO
Me
CO2Et
Me
S
HO
NH3
N
H
H
N
OH
1H NMR (400 MHz, D2O)
1.00
1.91
0.91
2.14
1.02
0.98
1.94
1.96
6.29
3.24
10.0 9.5 9.0 8.5 8.0 7.5 7.0 6.5 6.0 5.5 5.0 4.5 4.0 3.5 3.0 2.5 2.0 1.5 1.0 0.5 0.0
f1 (ppm)

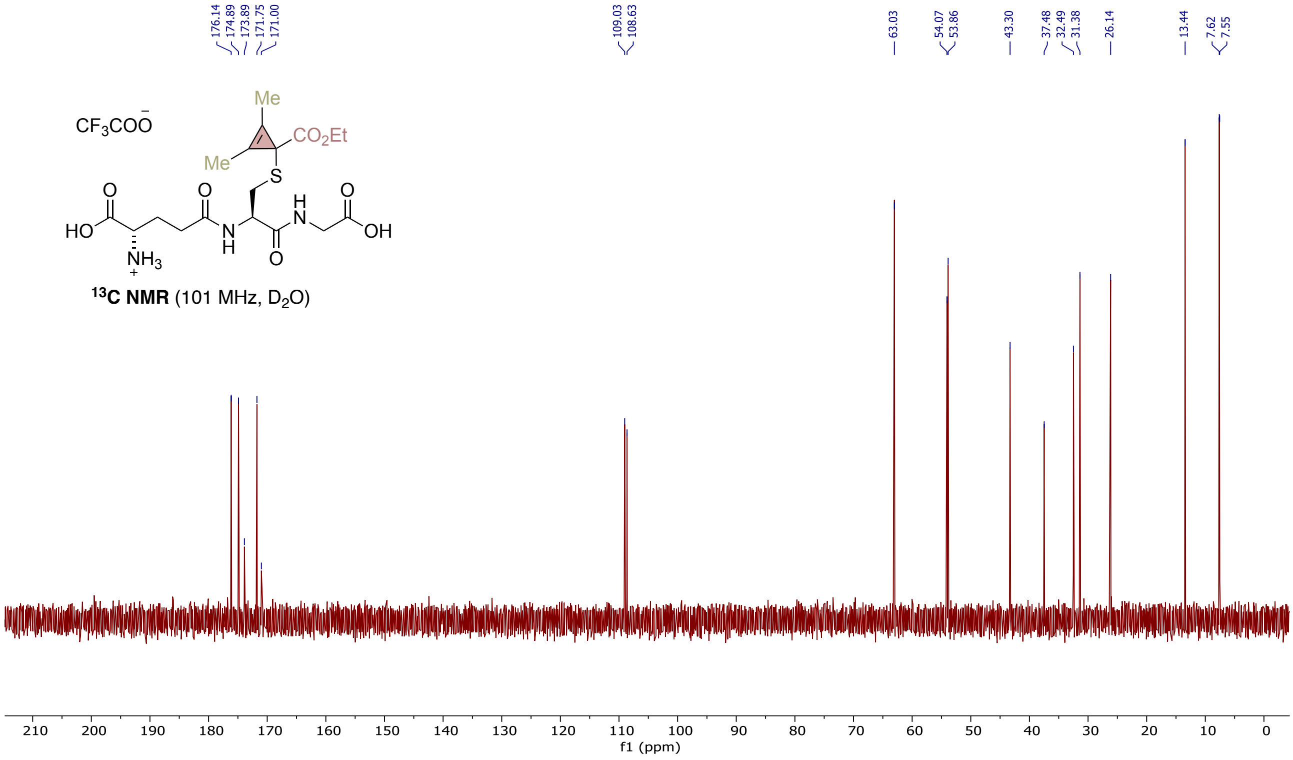

176.14
174.89
173.89
171.75
171.00
109.03
108.63
63.03
54.07
53.86
43.30
37.48
32.49
31.38
26.14
13.44
7.62
7.55
CF3COO
Me
CO2Et
Me
S
HO
NH3
N
H
H
N
OH
13C NMR (101 MHz, D2O)
210 200 190 180 170 160 150 140 130 120 110 100 90 80 70 60 50 40 30 20 10 0
f1 (ppm)

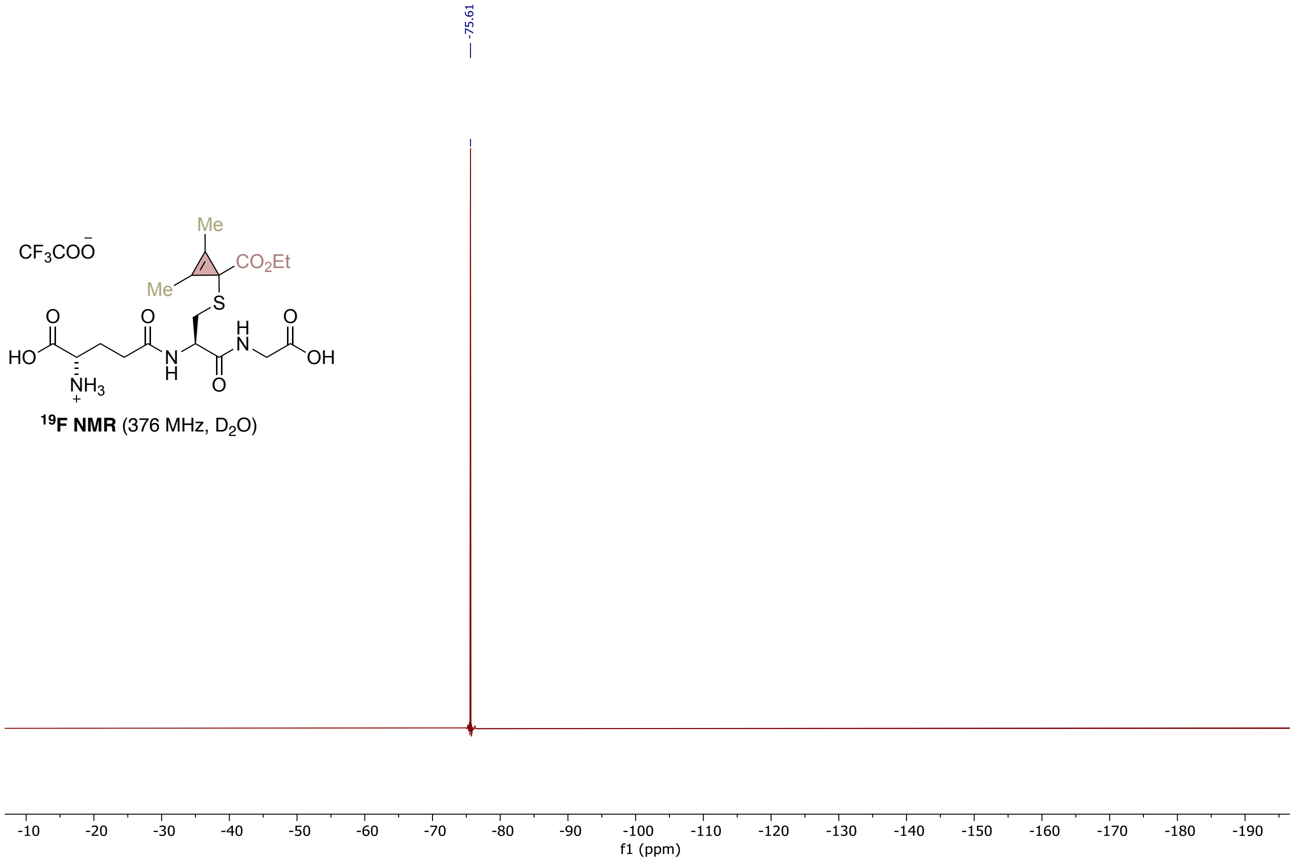

^{19}F NMR (376 MHz, D_2O)

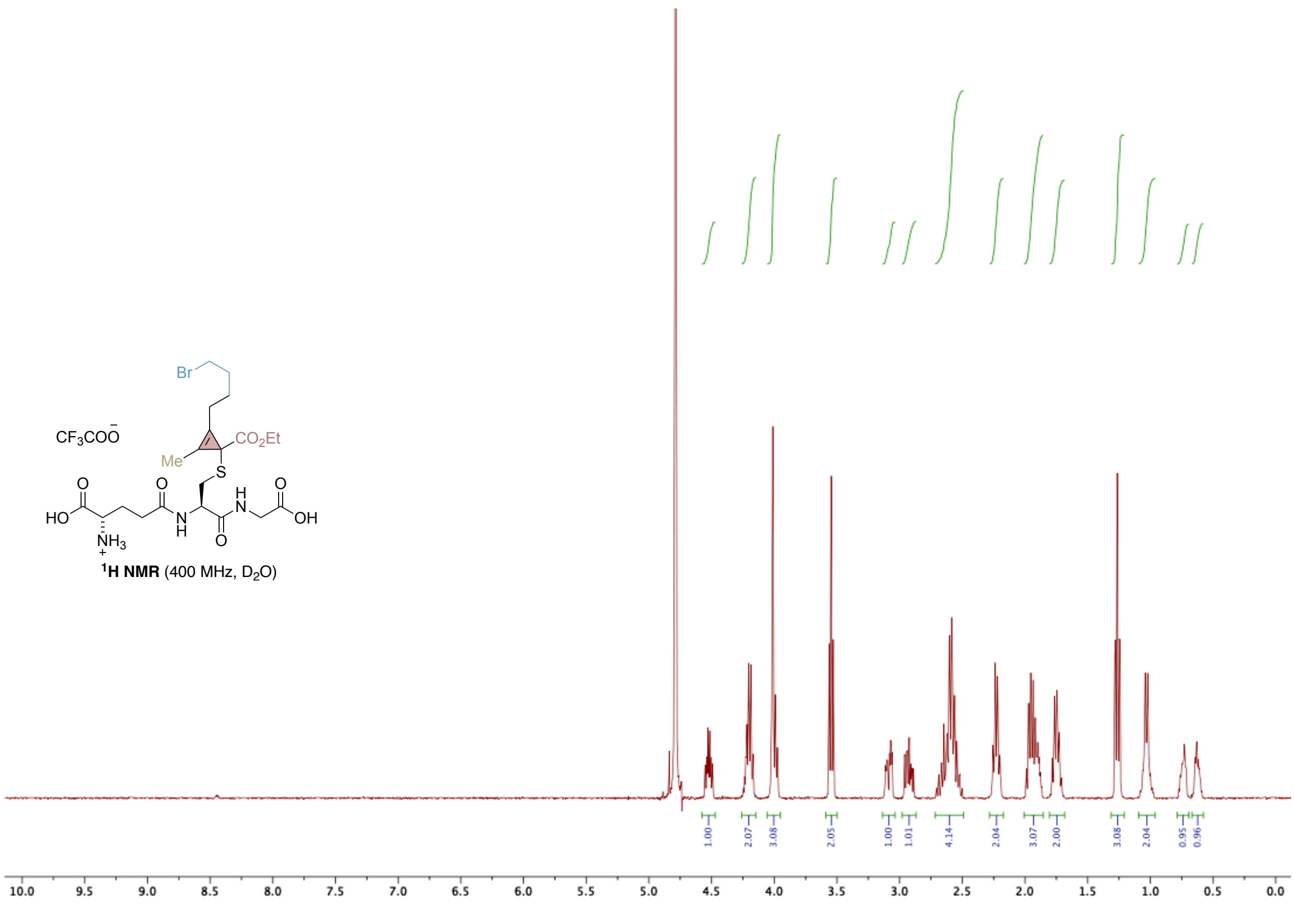
CF3COO
Br
CO2Et
Me
S
HO
NH3
N
H
OH
1H NMR (400 MHz, D2O)
1.00
2.07
3.08
2.05
1.00
1.01
4.14
2.04
3.07
2.00
3.08
2.04
0.95
0.96

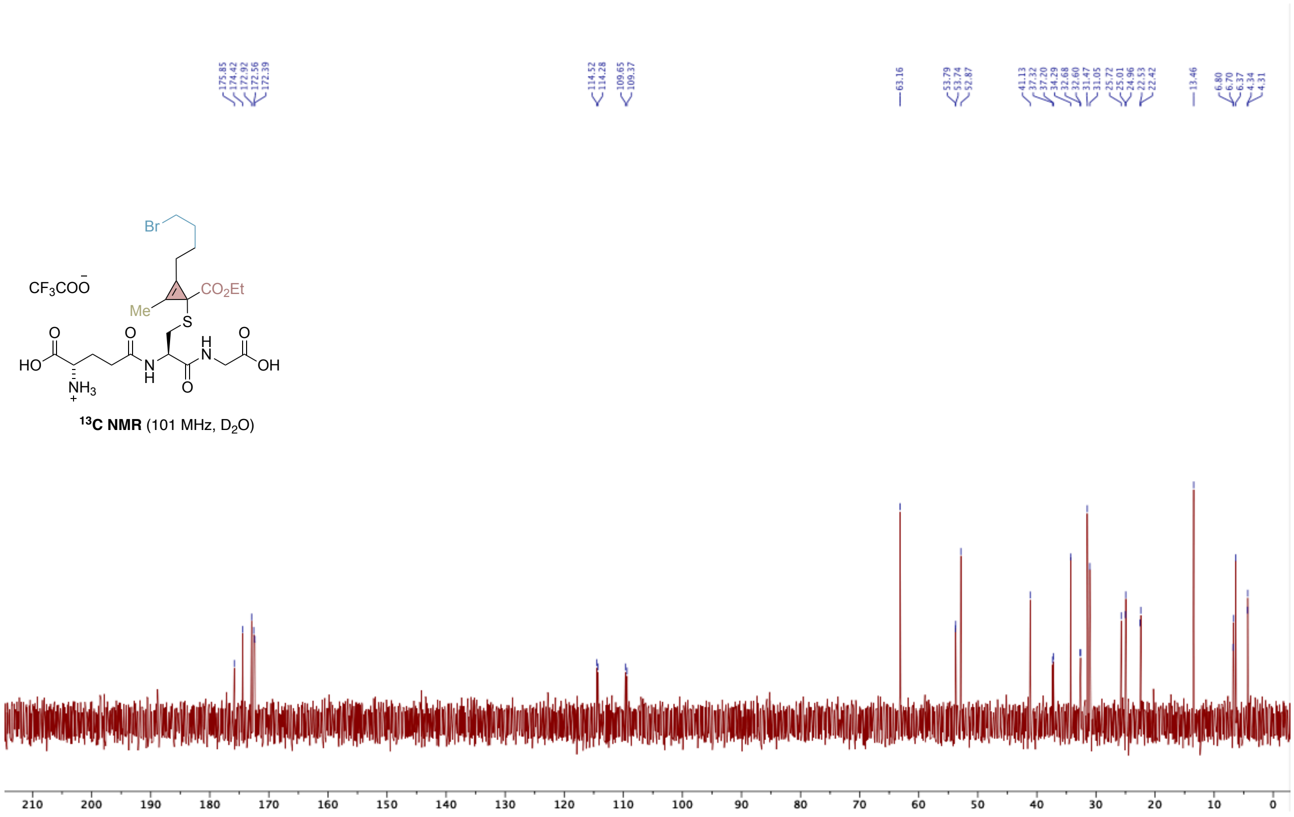
175.85
174.42
172.92
172.56
172.39
114.52
114.28
109.65
109.37
63.16
53.79
53.74
52.87
41.13
37.32
37.20
34.29
32.68
32.60
31.47
31.05
25.72
25.01
24.96
22.53
22.42
13.46
6.80
6.70
6.37
4.34
4.31
CF3COO
Br
CO2Et
Me
S
HO
NH3
N
H
OH
13C NMR (101 MHz, D2O)

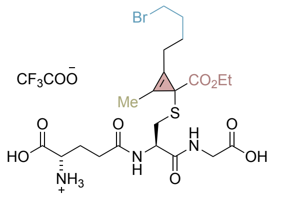

^{13}C NMR (101 MHz, D_2O)

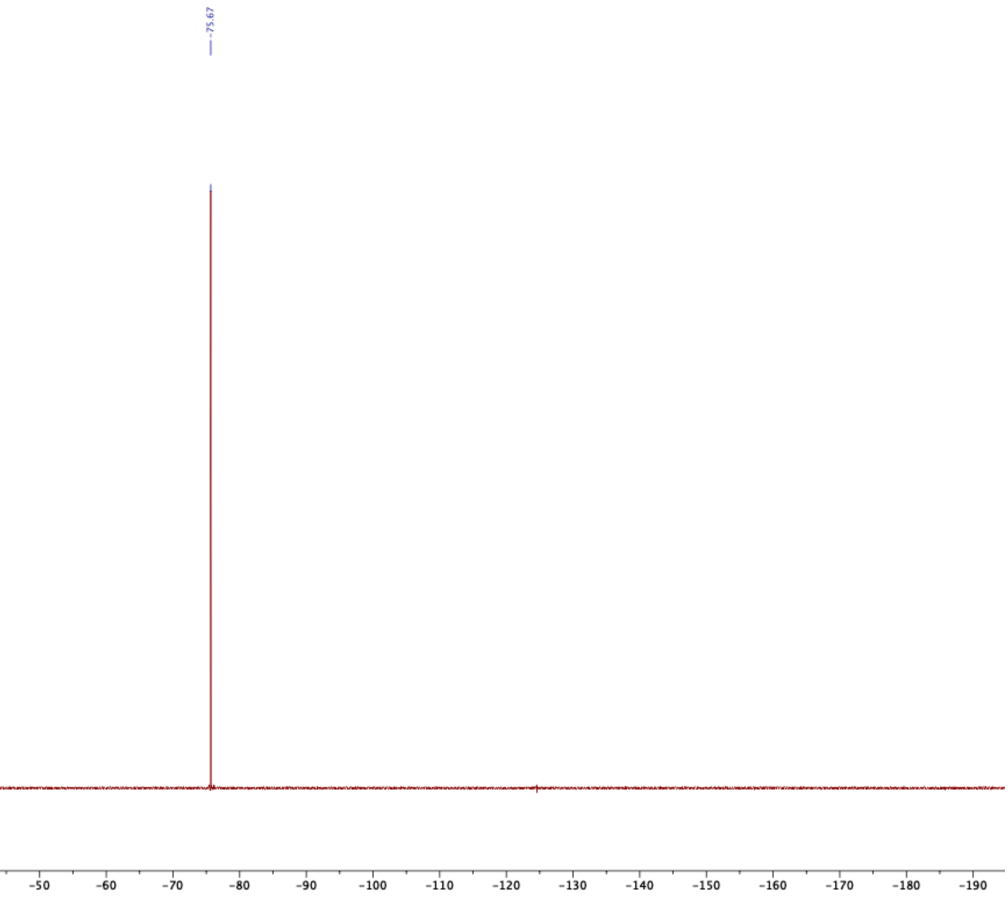

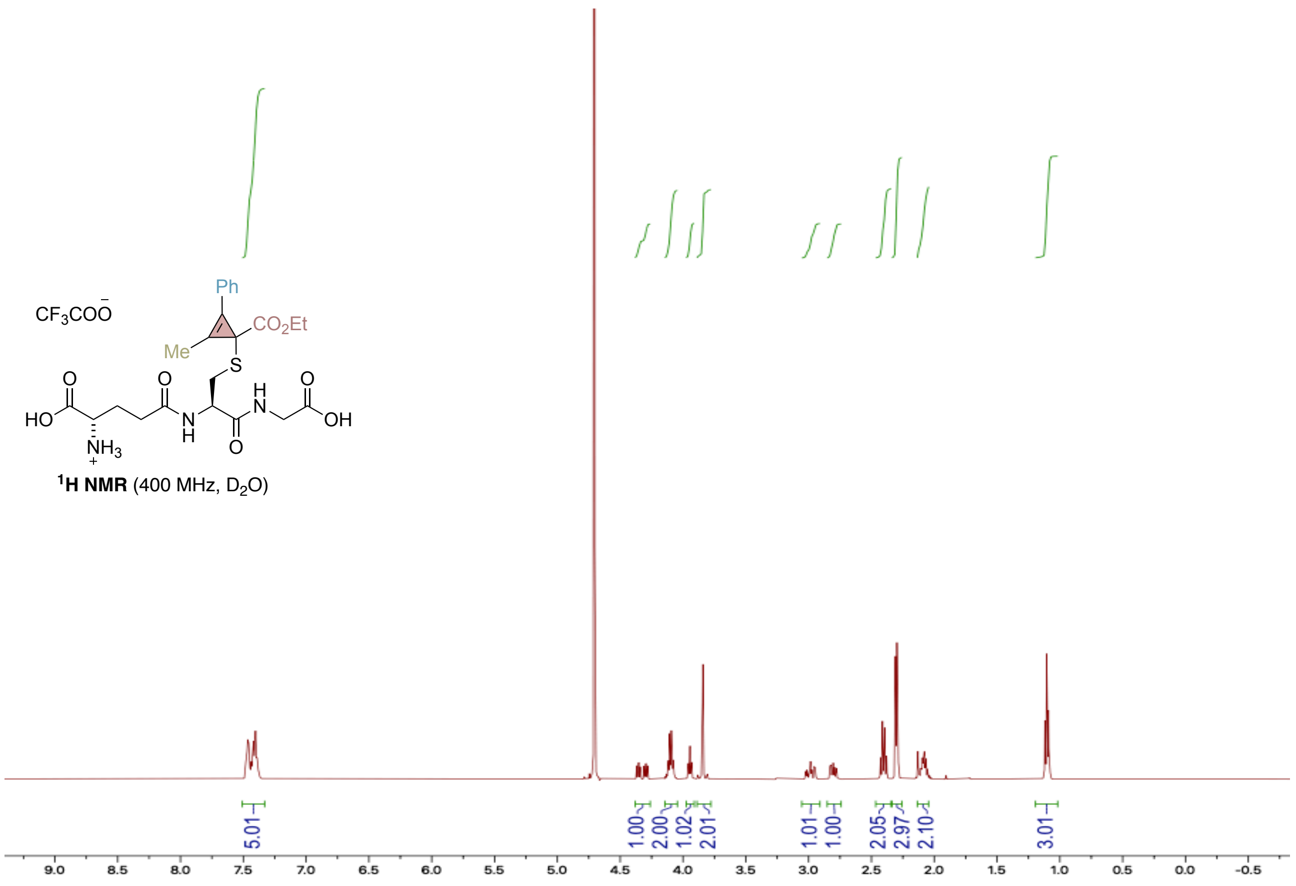
CF3COO
Ph
CO2Et
Me
S
HO
NH3
N
H
O
OH
1H NMR (400 MHz, D2O)
5.01
1.00
2.00
1.02
2.01
1.01
1.00
2.05
2.97
2.10
3.01

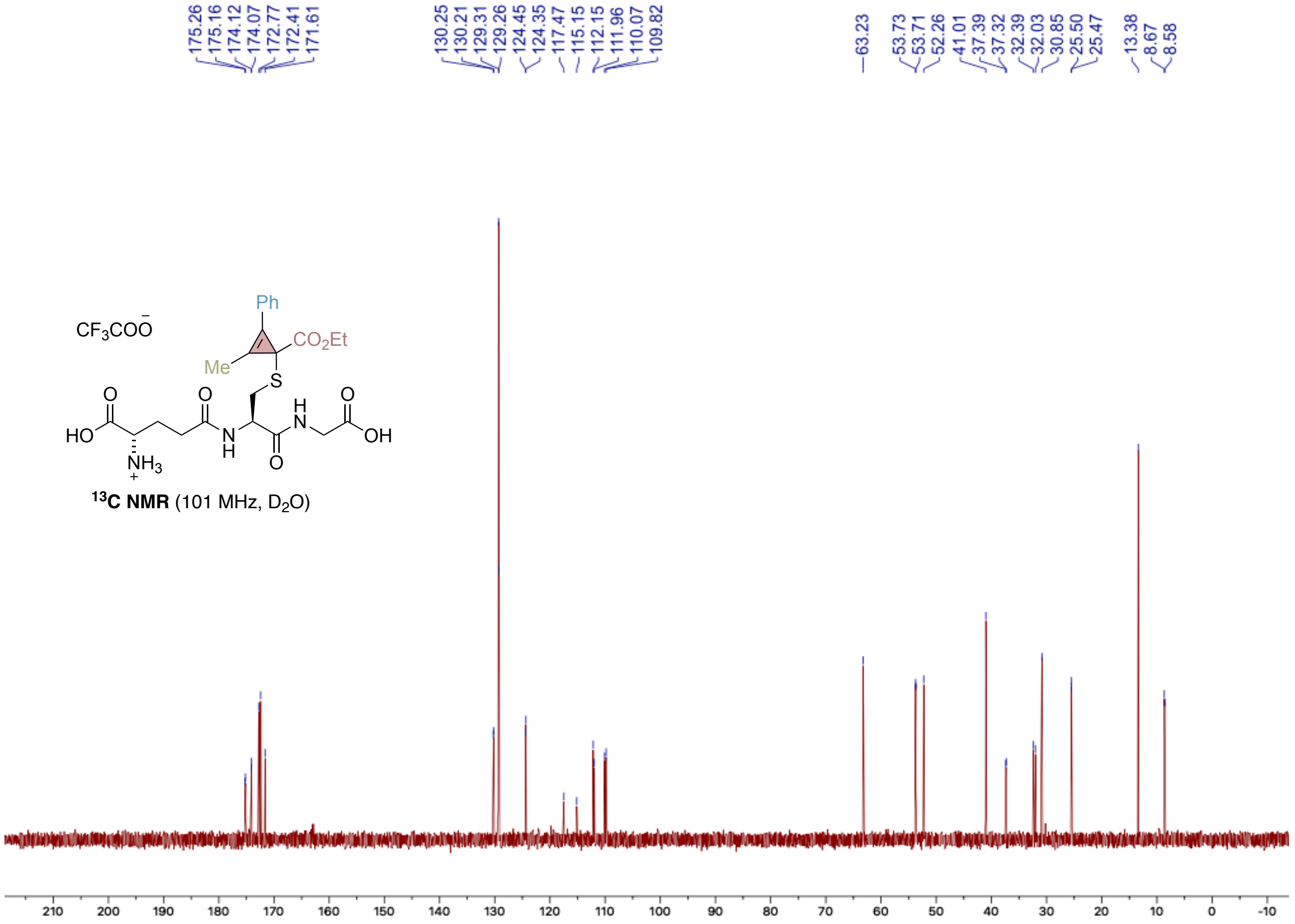
175.26
175.16
174.12
174.07
172.77
172.41
171.61
130.25
130.21
129.31
129.26
124.45
124.35
117.47
115.15
112.15
111.96
110.07
109.82
63.23
53.73
53.71
52.26
41.01
37.39
37.32
32.39
32.03
30.85
25.50
25.47
13.38
8.67
8.58
CF3COO
Ph
CO2Et
Me
S
HO
NH3
N
H
O
OH
13C NMR (101 MHz, D2O)

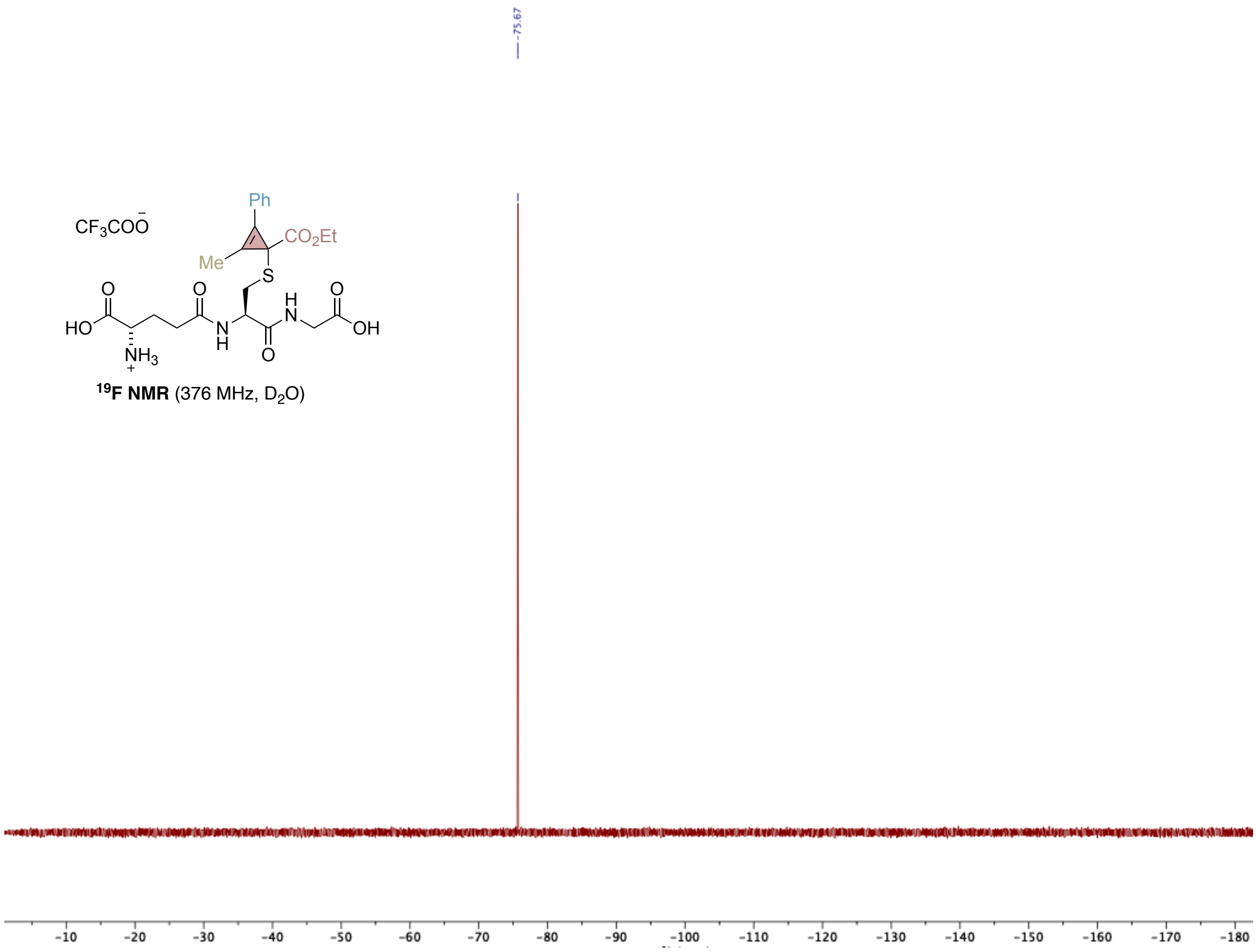

^{19}F NMR (376 MHz, D_2O)

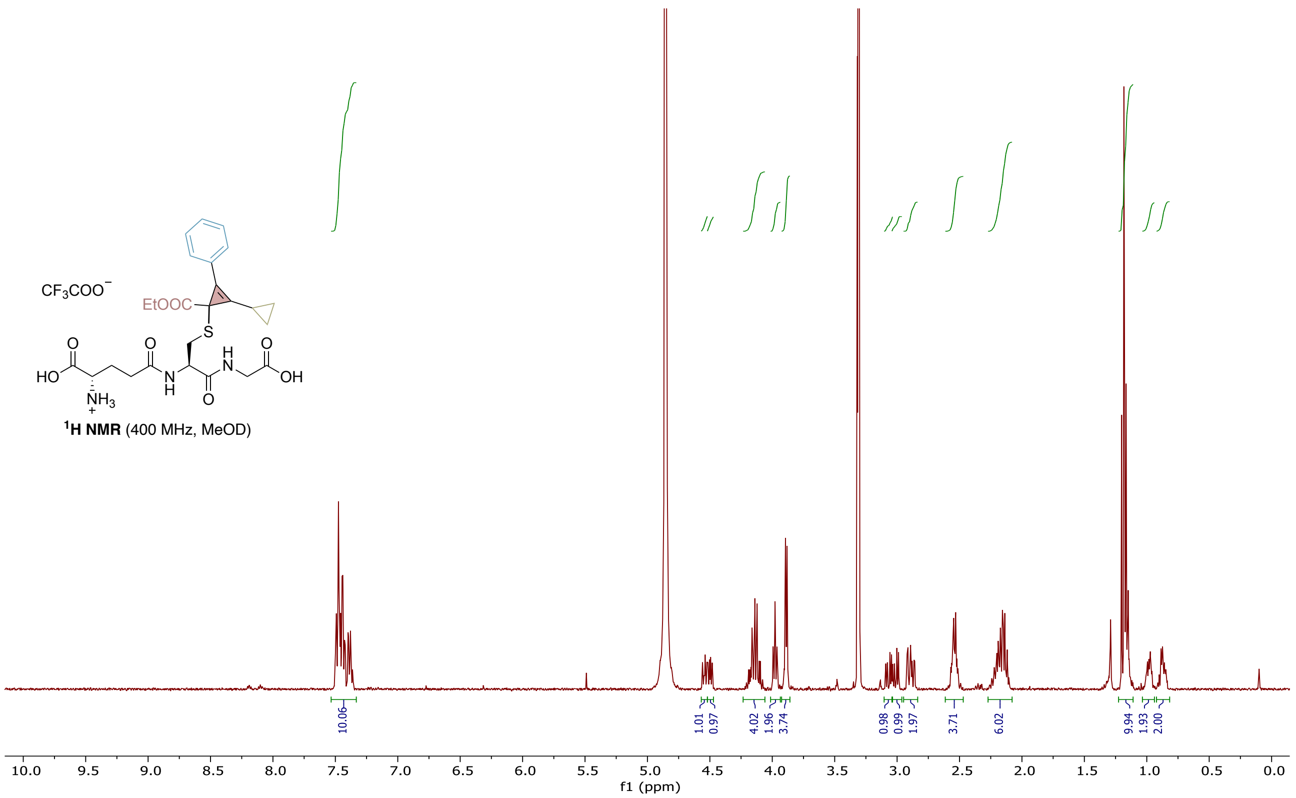
CF3COO−
EtOOC
S
HO
NH3
OH
1H NMR (400 MHz, MeOD)
f1 (ppm)

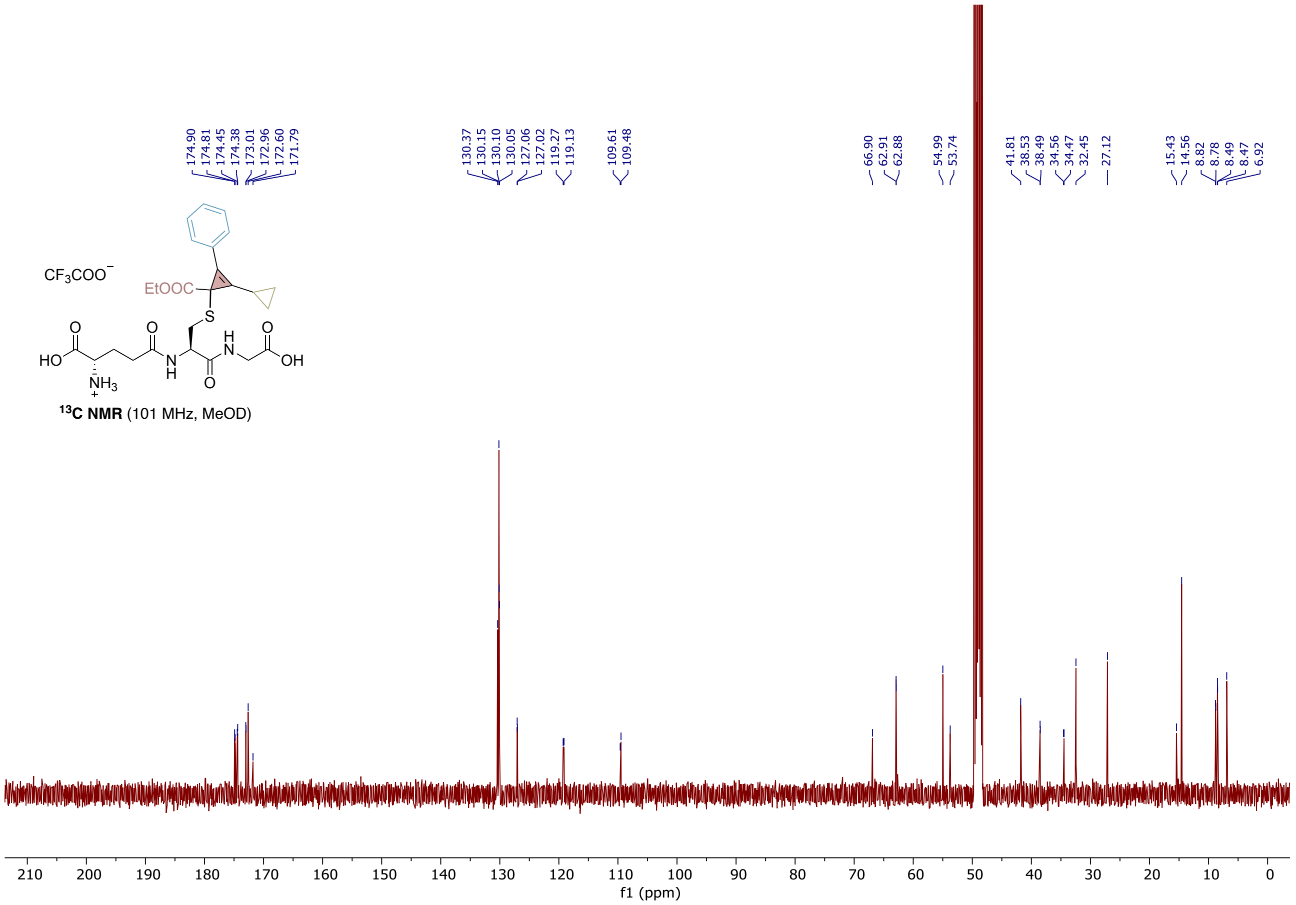
174.90
174.81
174.45
174.38
173.01
172.96
172.60
171.79
130.37
130.15
130.10
130.05
127.06
127.02
119.27
119.13
109.61
109.48
66.90
62.91
62.88
54.99
53.74
41.81
38.53
38.49
34.56
34.47
32.45
27.12
15.43
14.56
8.82
8.78
8.49
8.47
6.92
CF3COO−
EtOOC
13C NMR (101 MHz, MeOD)
f1 (ppm)

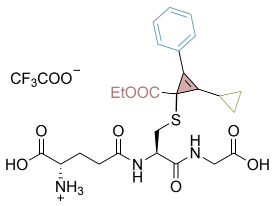

19F NMR (376 MHz, MeOD)

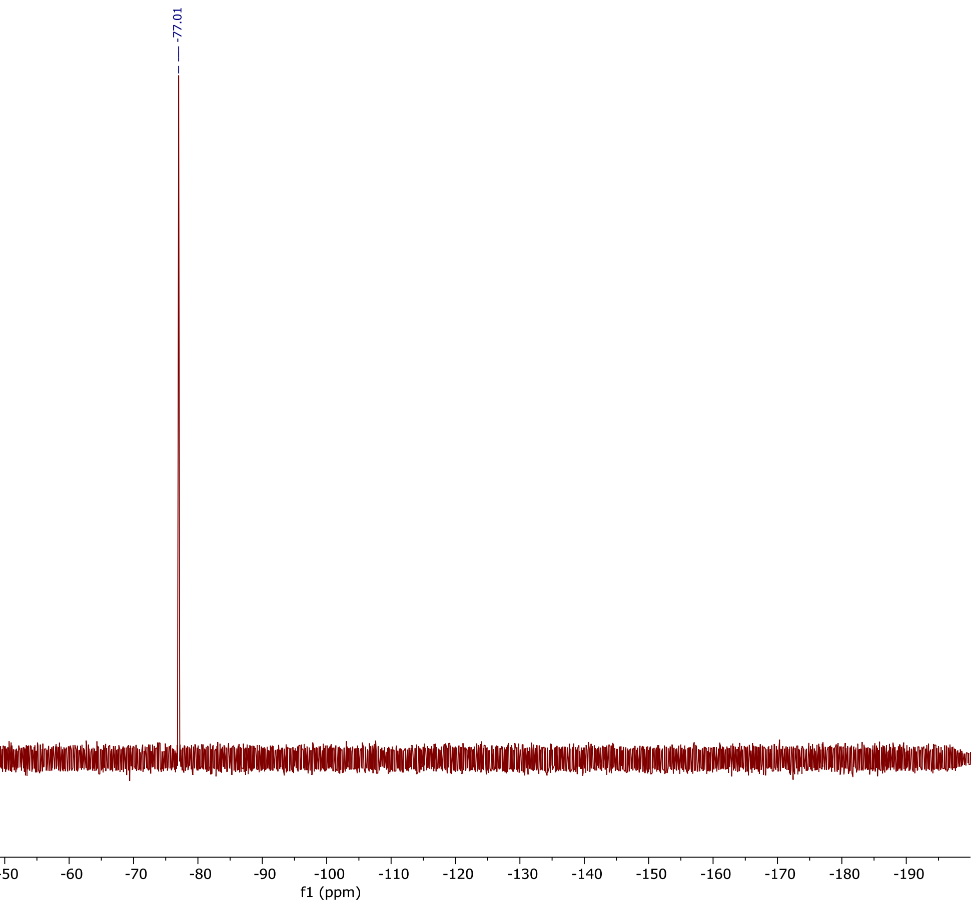

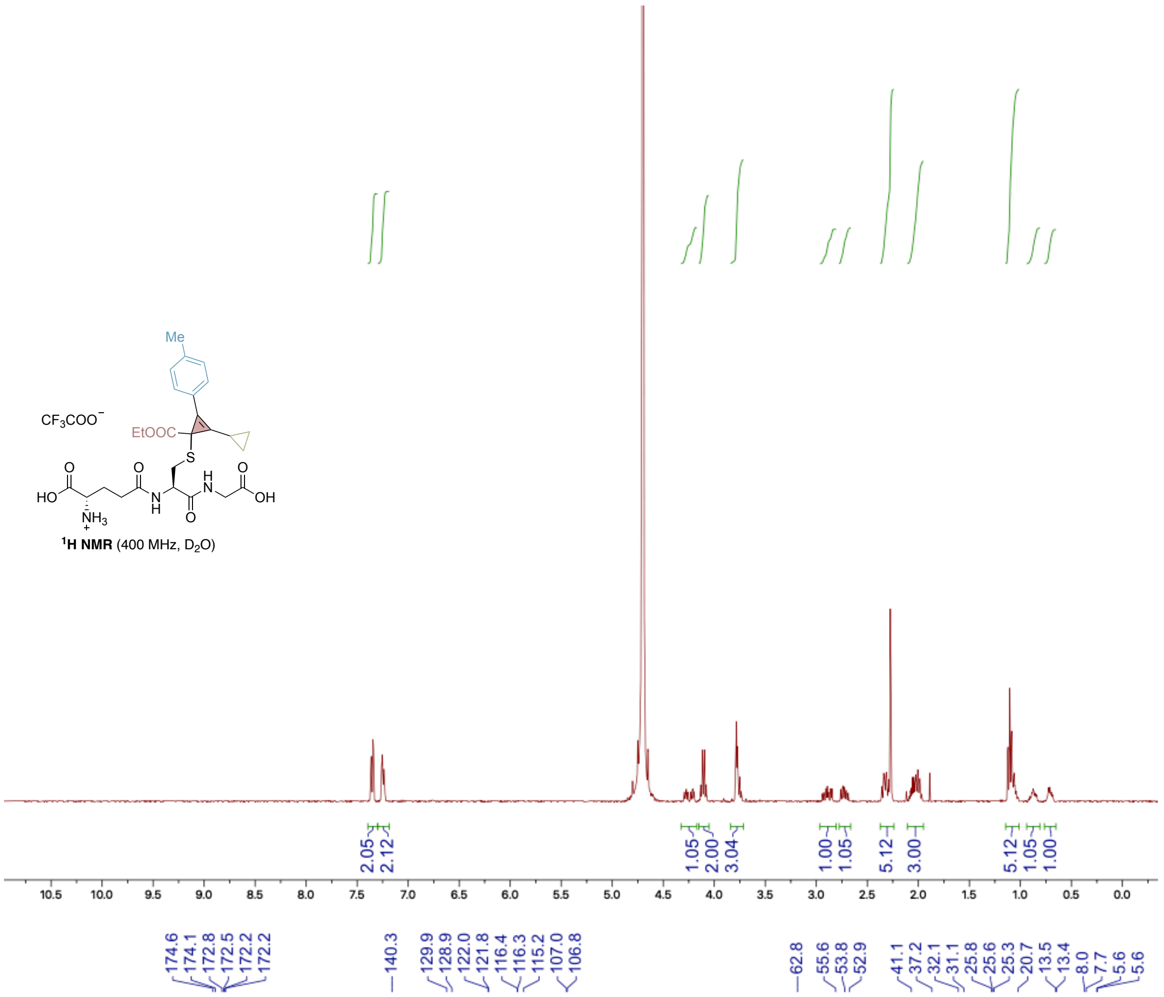

CF3COO−
EtOOC
Me
S
HO
OH
NH3
1H NMR (400 MHz, D2O)

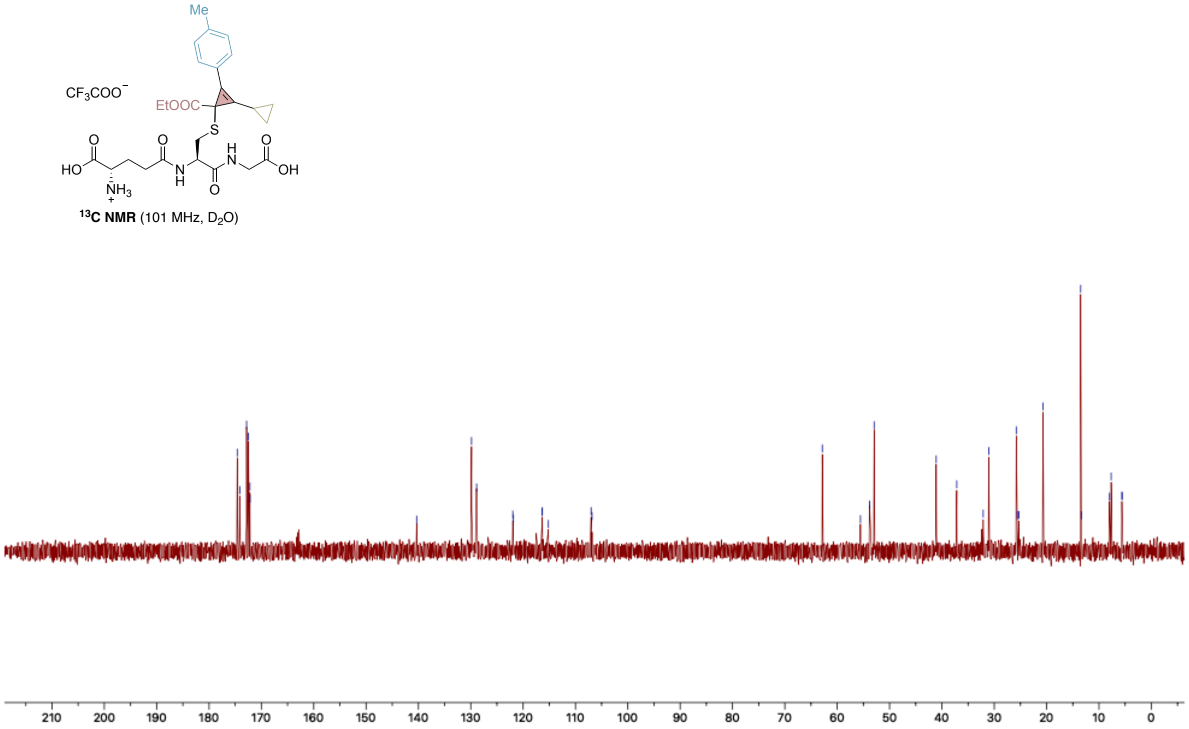

CF3COO−
EtOOC
Me
13C NMR (101 MHz, D2O)

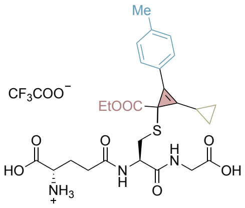

^{19}**F NMR** (376 MHz, D_2O)

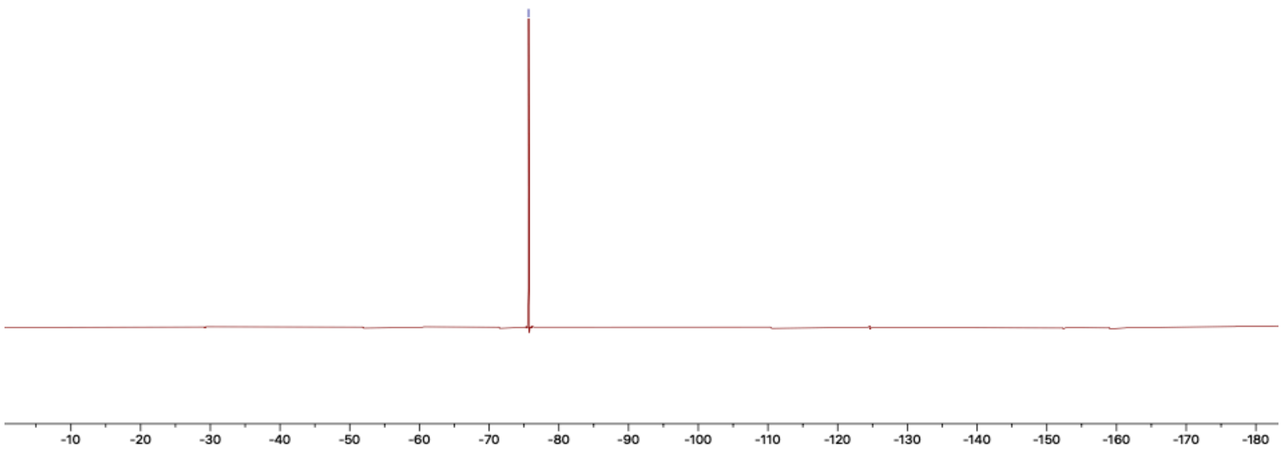

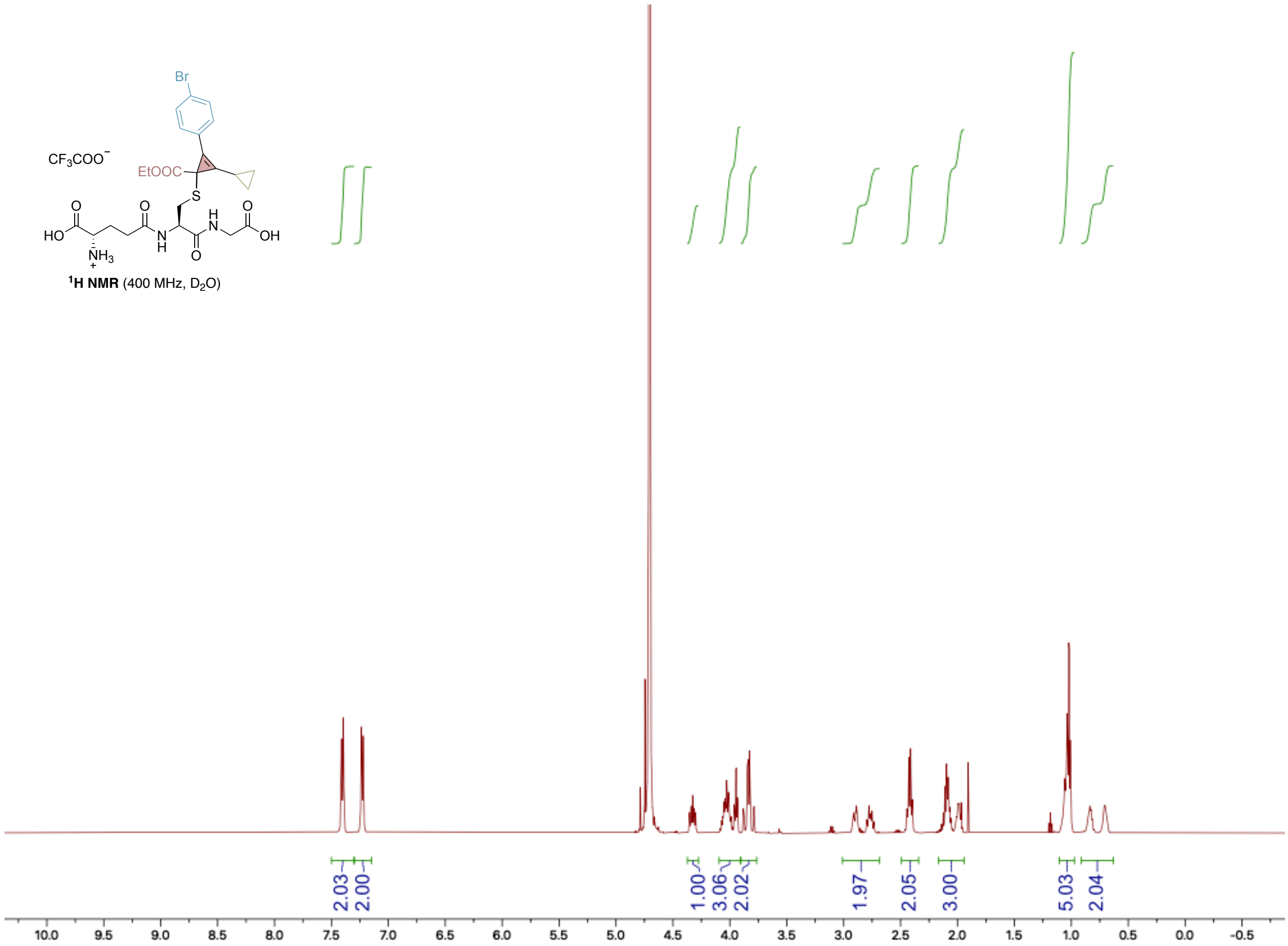
CF3COO−
EtOOC
Br
S
HO
NH3
OH
¹H NMR (400 MHz, D2O)
2.03
2.00
1.00
3.06
2.02
1.97
2.05
3.00
5.03
2.04

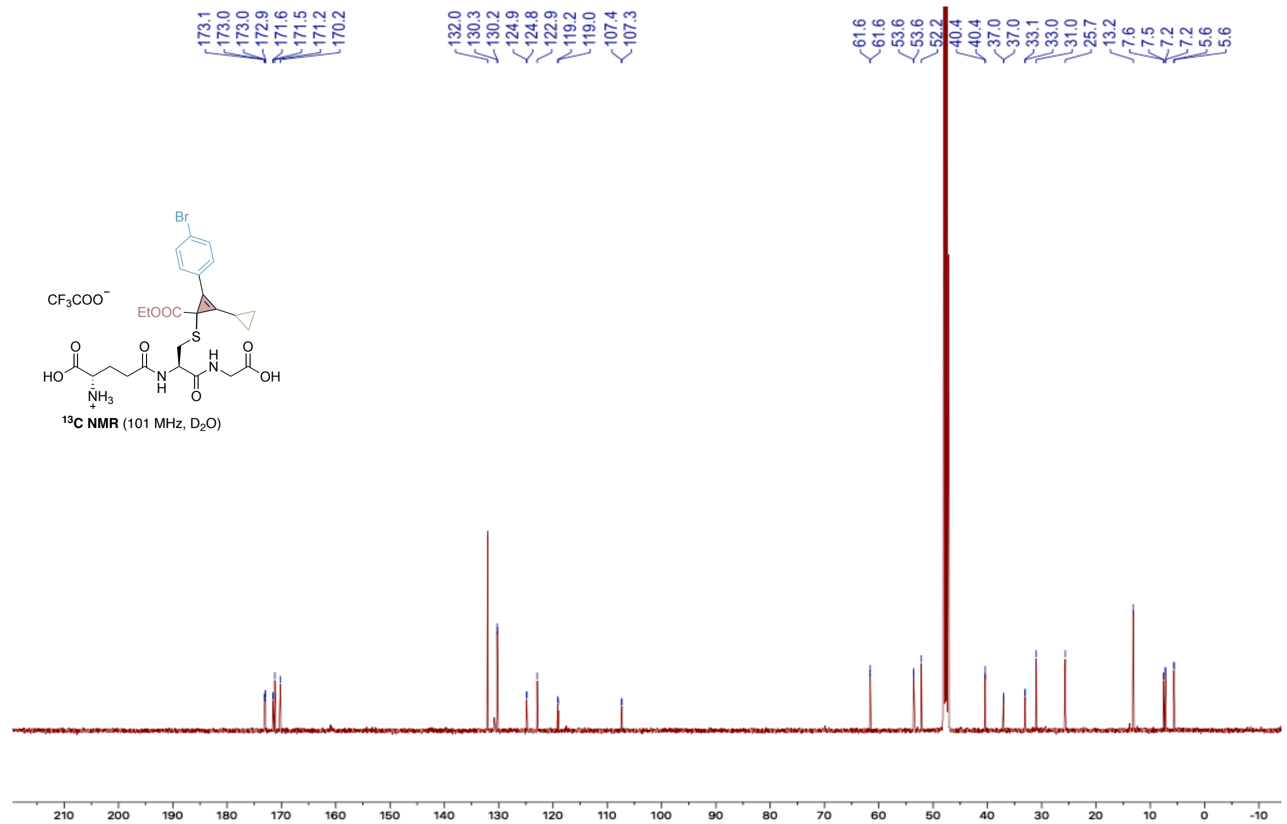
173.1
173.0
173.0
172.9
171.6
171.5
171.2
170.2
132.0
130.3
130.2
124.9
124.8
122.9
119.2
119.0
107.4
107.3
61.6
61.6
53.6
53.6
52.2
40.4
40.4
37.0
37.0
33.1
33.0
31.0
25.7
13.2
7.6
7.5
7.2
7.2
5.6
5.6
CF3COO−
EtOOC
Br
¹³C NMR (101 MHz, D2O)

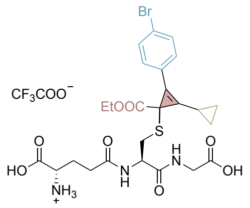

^{19}F NMR (376 MHz, D_2O)

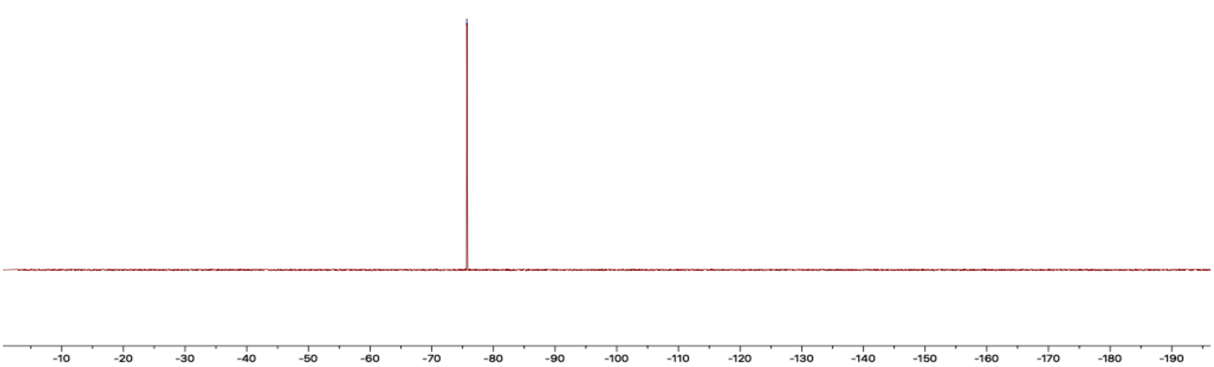

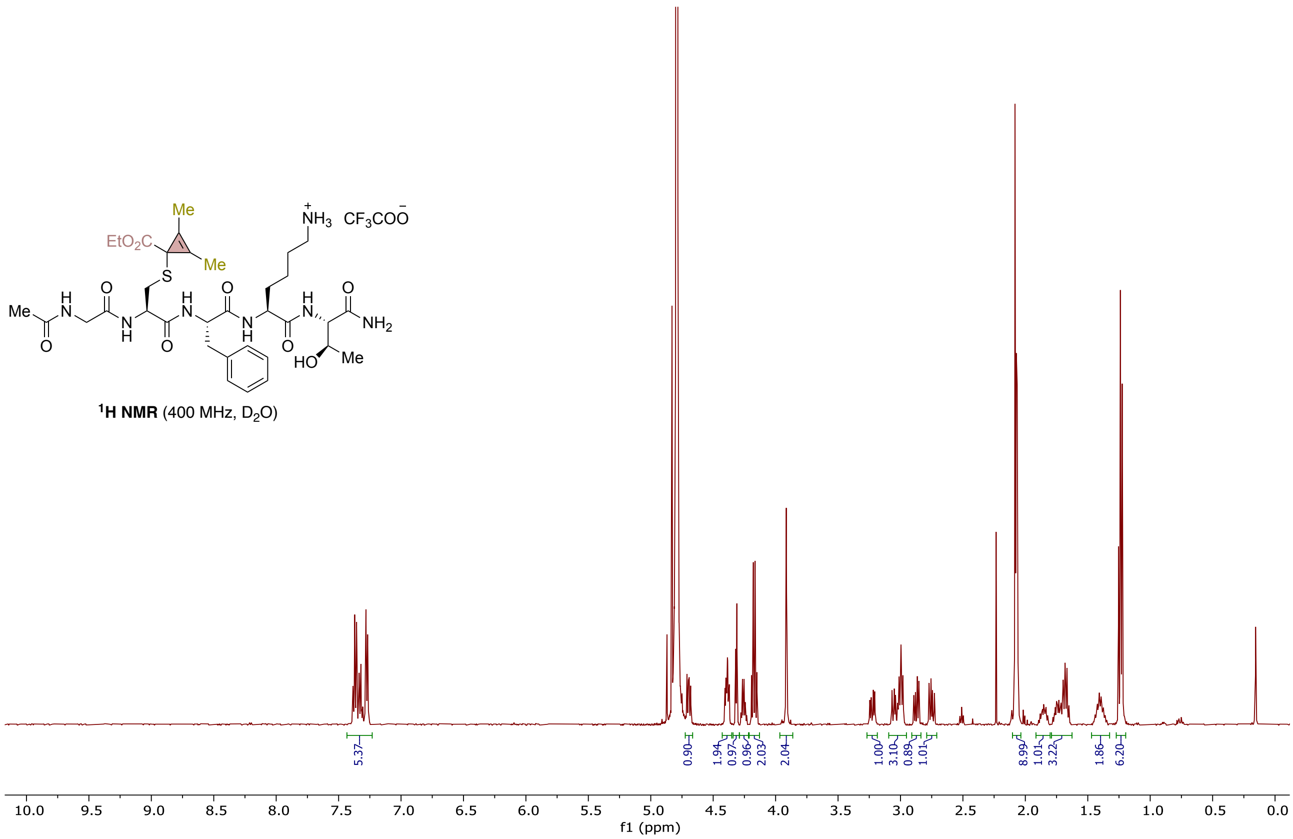

^{1}H NMR (400 MHz, D_2O)

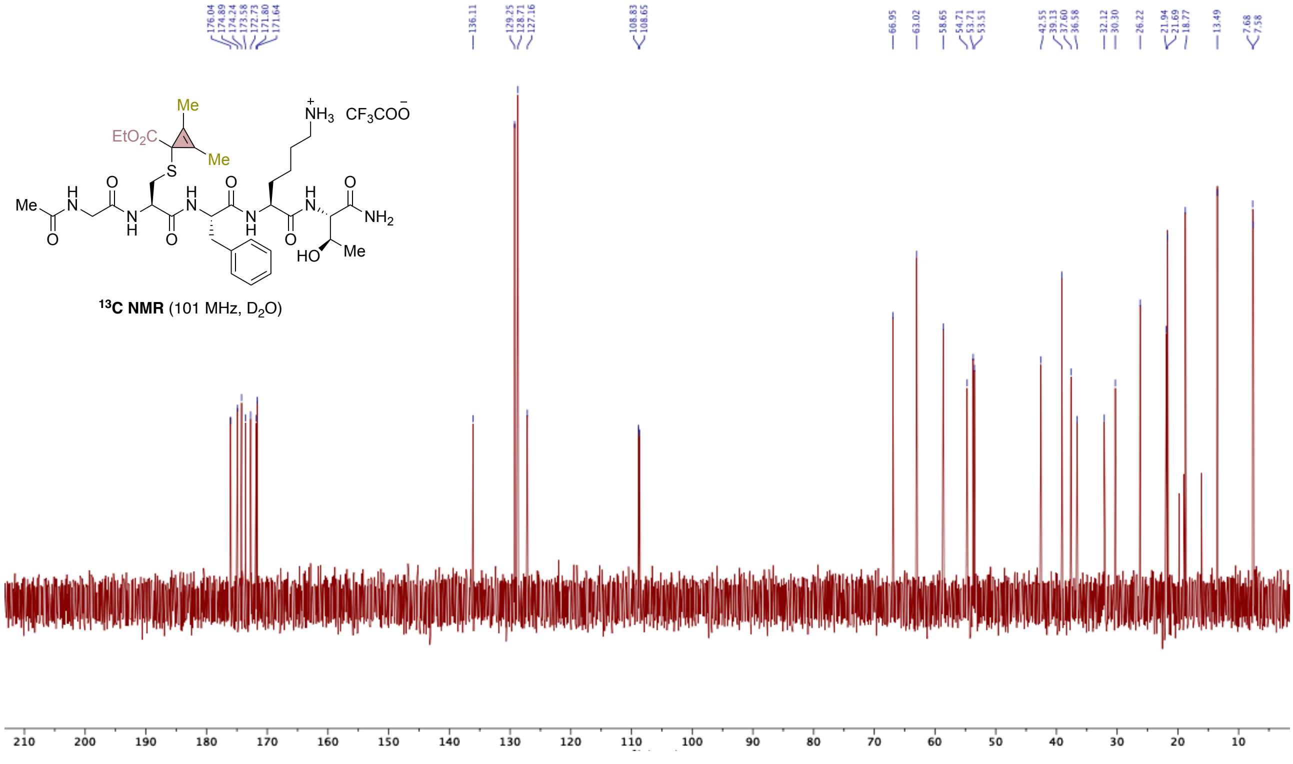

^{13}C NMR (101 MHz, D_2O)

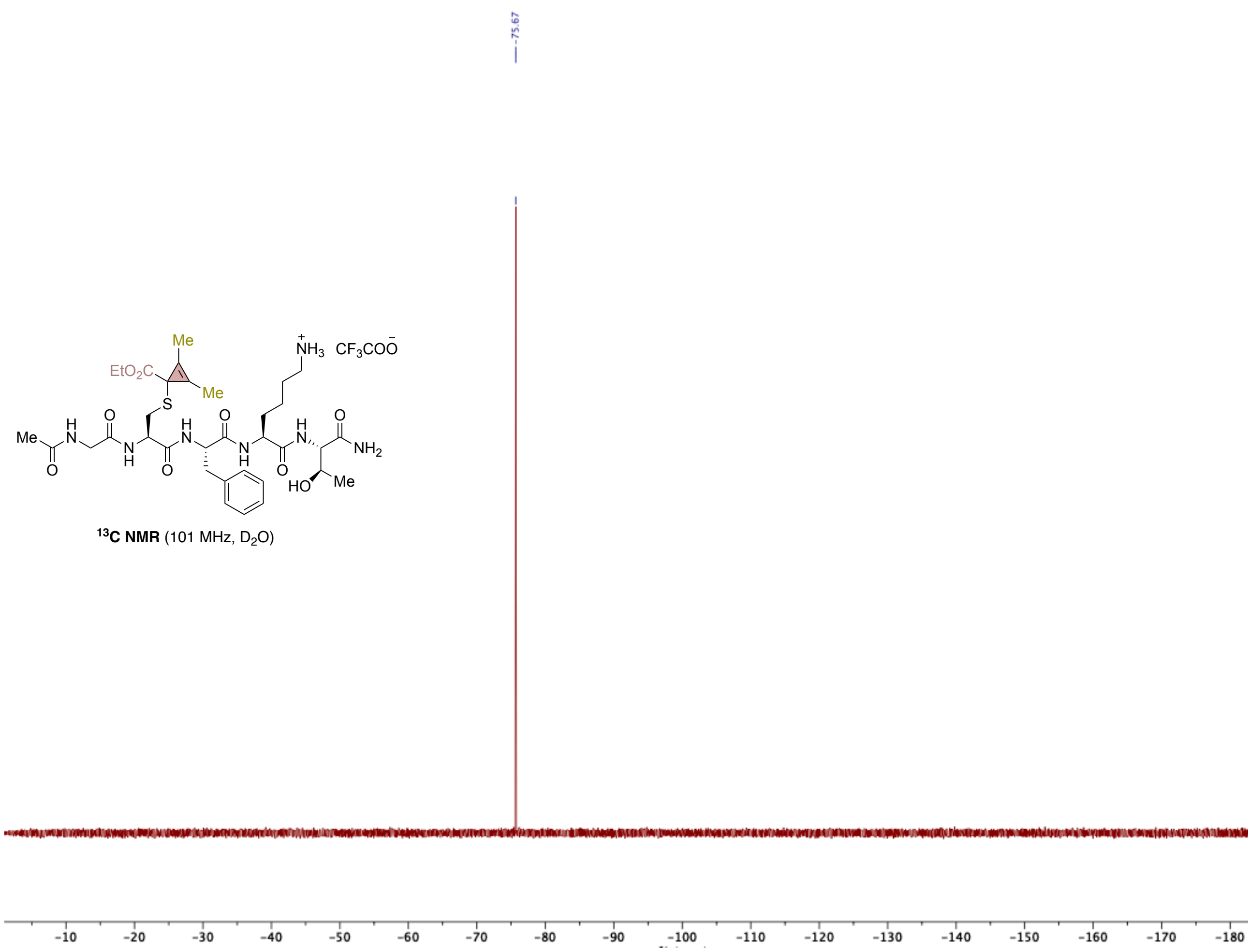

^{13}C NMR (101 MHz, D_2O)

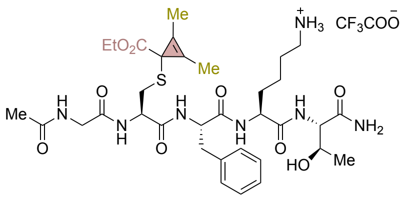

TOCSY (400 MHz, D_2O)

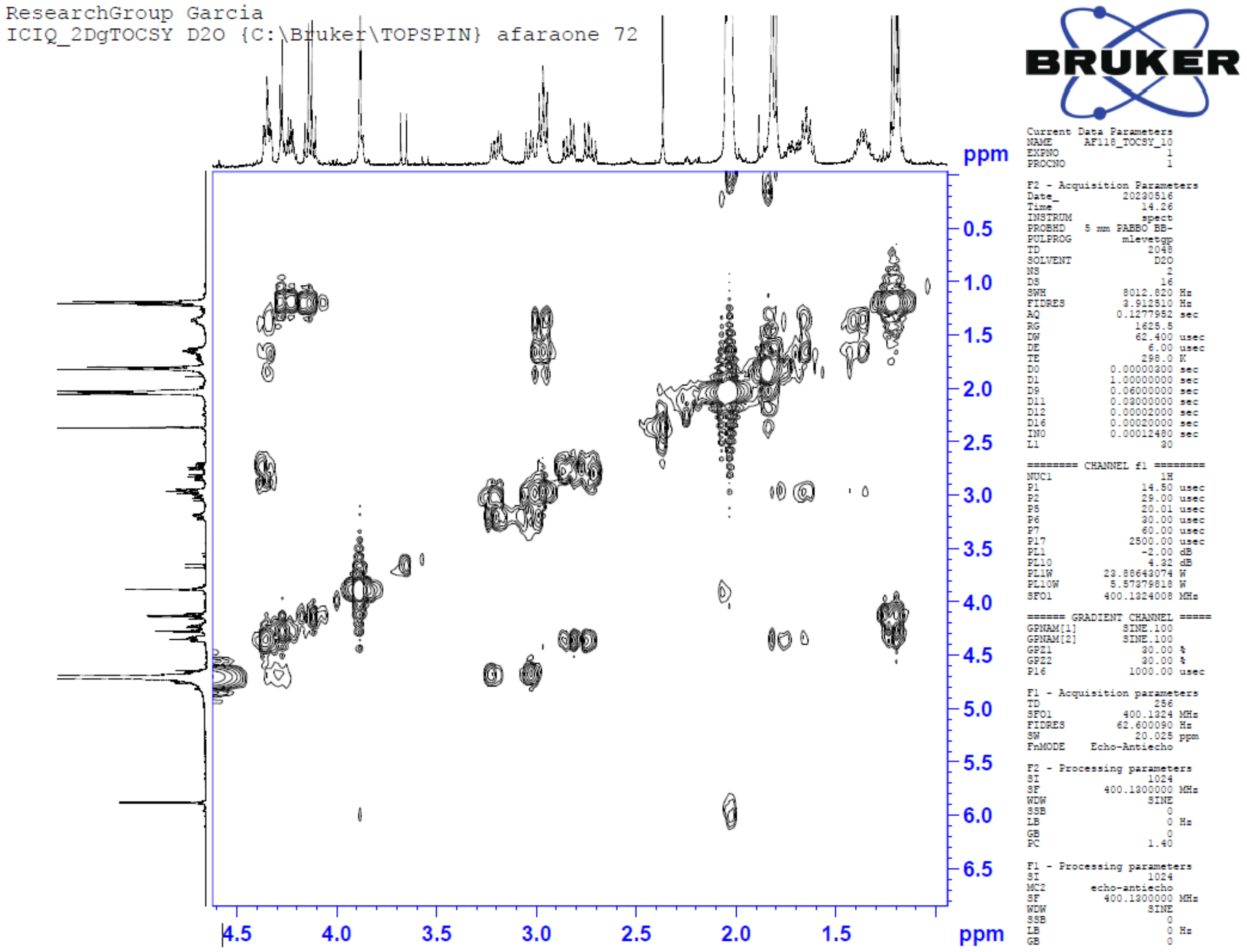

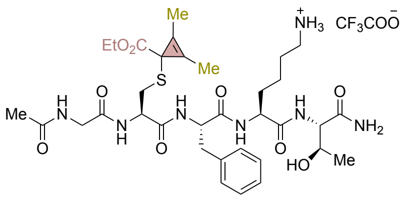

ROESY (400 MHz, D_2O)

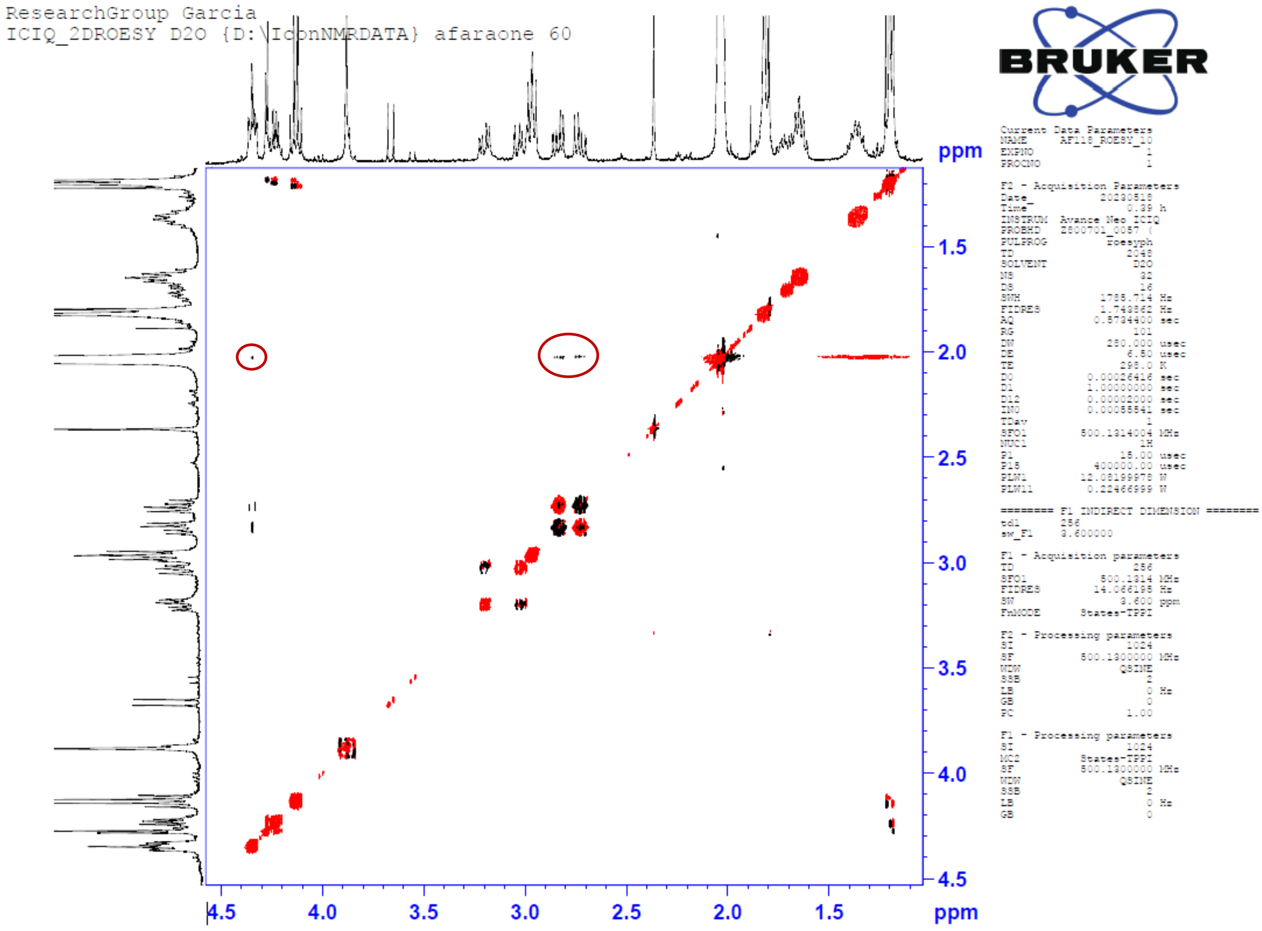

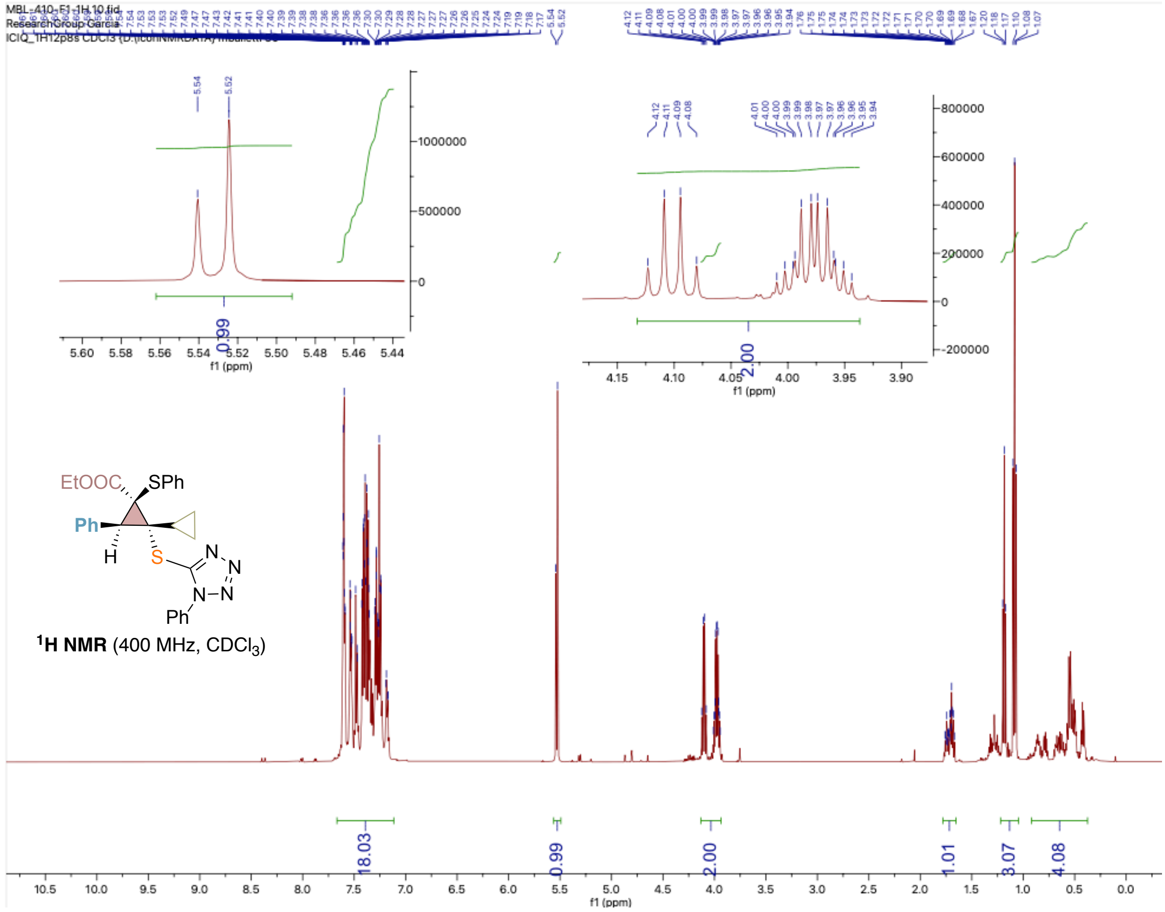
EtOOC
SPh
Ph
H
S
N
N
N
N
Ph
1H NMR (400 MHz, CDCl3)
18.03
0.99
2.00
1.01
3.07
4.08
f1 (ppm)

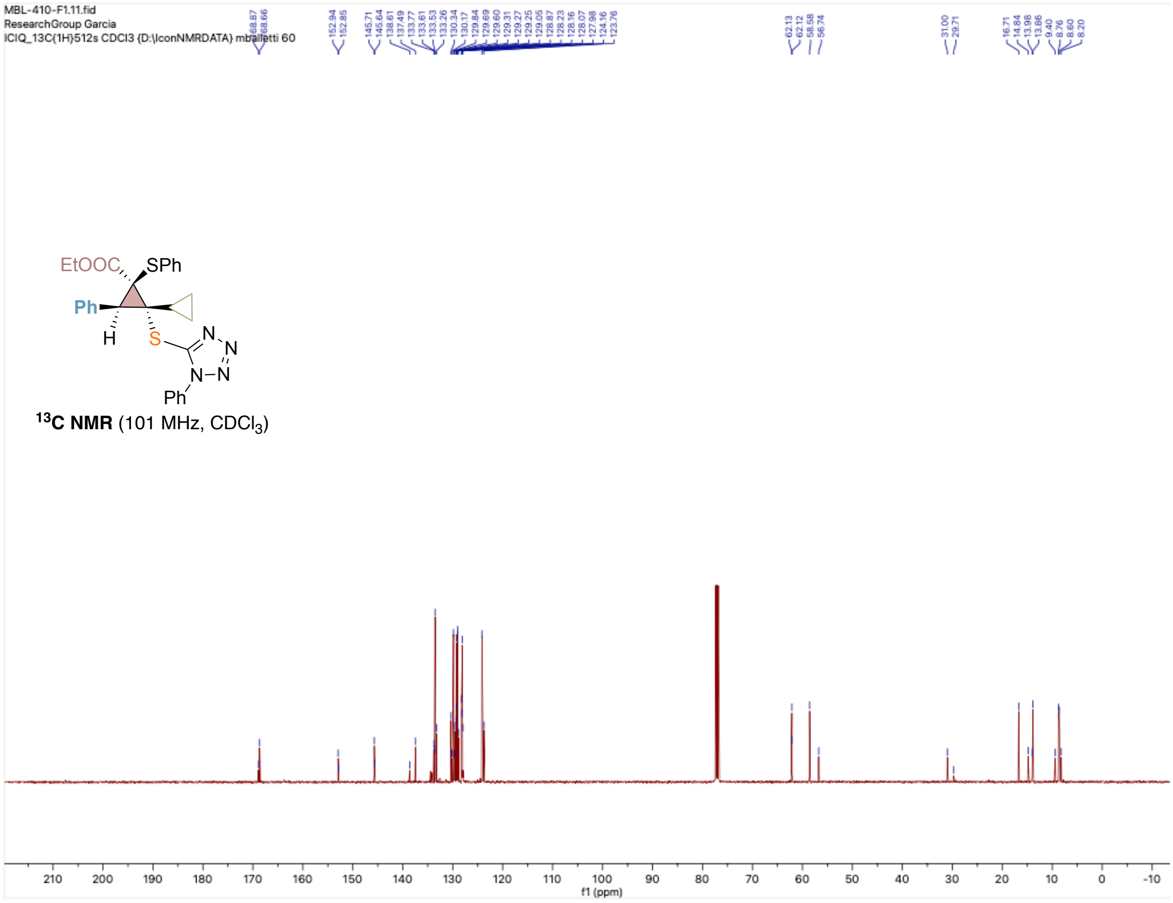
MBL-410-F1.11.fid
ResearchGroup Garcia
EtOOC
SPh
Ph
H
S
N
N
N
N
Ph
13C NMR (101 MHz, CDCl3)
f1 (ppm)

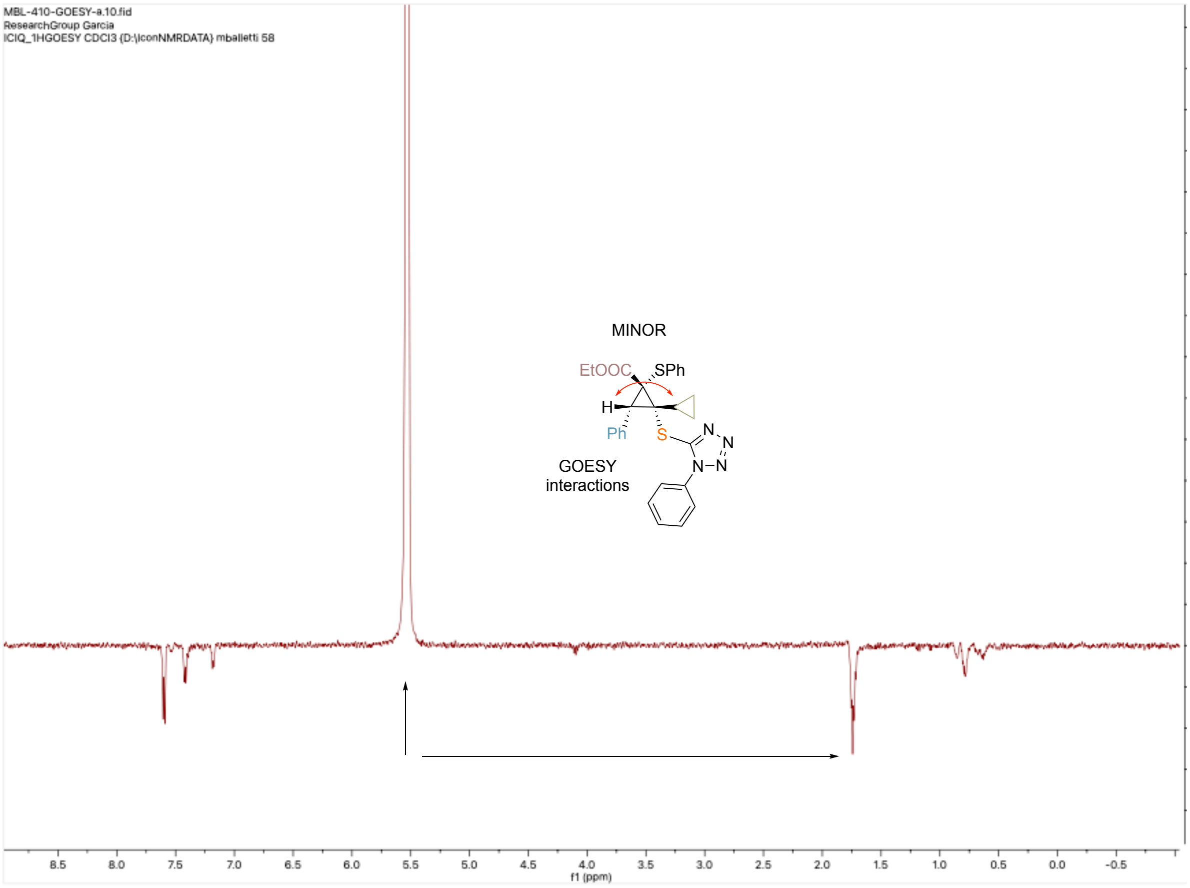
MBL-410-GOESY-a.10.fid
ResearchGroup Garcia
ICIQ_1HGOESY CDCl3 (D:\IconNMRDATA) mballetti 58
MINOR
EtOOC
SPh
H
Ph
S
N
N
N
N
GOESY
interactions
8.5
8.0
7.5
7.0
6.5
6.0
5.5
5.0
4.5
4.0
3.5
3.0
2.5
2.0
1.5
1.0
0.5
0.0
-0.5
f1 (ppm)

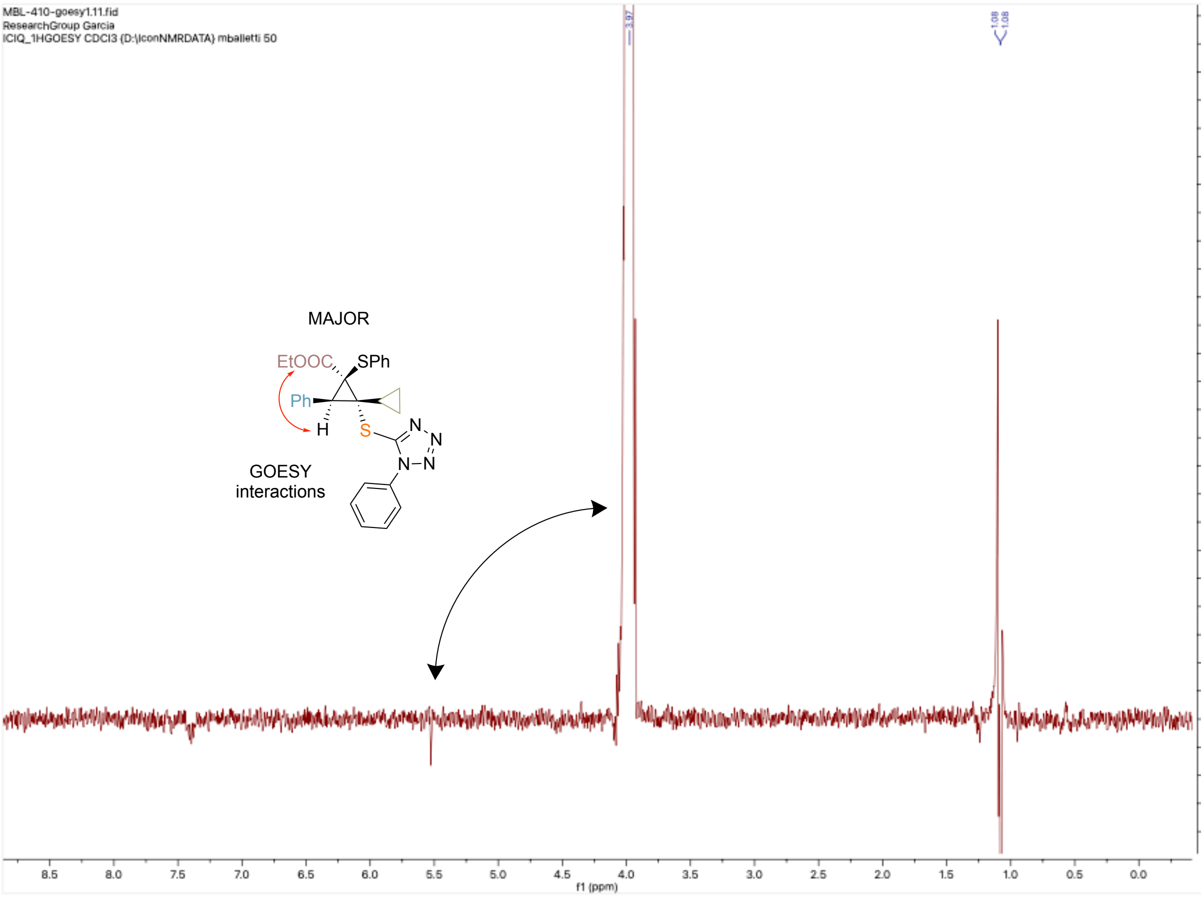
MBL-410-goesy1.11.fid
ResearchGroup Garcia
ICIQ_1HGOESY CDCl3 (D:\IconNMRDATA) mballetti 50
MAJOR
EtOOC
SPh
Ph
H
S
N
N
N
N
GOESY
interactions
8.5
8.0
7.5
7.0
6.5
6.0
5.5
5.0
4.5
4.0
3.5
3.0
2.5
2.0
1.5
1.0
0.5
0.0
f1 (ppm)
